# Multi-kingdom gut microbiota analyses identify biomarkers of different types of pediatric short bowel syndrome

**DOI:** 10.1128/msystems.00316-26

**Published:** 2026-09-01

**Authors:** Qing-Qing Wu, Cheng-Yong Li, Shan-Shan Chen, Juan Xu, Wei-Hui Yan, Li-Na Lu, Hai-Xia Feng, Jin-An Zhou, Lu Wang, Ning-Ning Liu, Lu Jiang, Ying Wang

**Affiliations:** 1 Division of Pediatric Gastroenterology and Nutrition, Xinhua Hospital, Shanghai Jiao Tong University School of Medicine 56694, Shanghai, China; 2 State Key Laboratory of Systems Medicine for Cancer, Center for Single-Cell Omics, School of Public Health, Shanghai Jiao Tong University School of Medicine 117884, Shanghai, China; 3 Shanghai Institute for Pediatric Research 674079 , Shanghai, China; 4 Shanghai Key Laboratory of Pediatric Gastroenterology and Nutrition 633081 , Shanghai, China; Boston College, Chestnut Hill, Massachusetts, USA

**Keywords:** short bowel syndrome, pediatric, multi-kingdom, functional signatures, microbiota

## Abstract

Despite recent advances in bacterial profiling across the three anatomical types of pediatric short bowel syndrome (SBS), the gut multi-kingdom remains unexplored. We characterized the four-kingdom gut microbiota using modified DNA extraction and deep shotgun metagenomic sequencing of fecal samples from 26 healthy controls and 34 pediatric SBS patients comprising three types (8 SBS I, 15 SBS II, and 11 SBS III). Overall, children with SBS exhibited a significant reduction in α-diversity compared with controls, with no difference observed among SBS types. Compared with controls, the proportion of archaea was significantly decreased in all SBS types, while bacteria, fungi, and viruses remained similar across types. Different types of SBS exhibited distinct microbial signatures: SBS I was enriched with pathogens (such as species from the *Streptococcus* and *Klebsiella* genera); SBS II was marked by a depletion of beneficial short-chain fatty acid-producing species (such as *Faecalibacterium prausnitzii*); and SBS III displayed loss of bile acid-metabolizing species, alongside a significant expansion of *Lactobacillus* species. Ecological networks were rewired in SBS, with disruption pronounced in SBS I. Functional analysis revealed that core metabolic pathways were markedly suppressed in SBS I compared with controls. Integrated correlation analyses revealed ileocecal valve loss as the primary determinant of SBS I microbial profiles, linking it to impaired secretory functions and hepatic injury. Our findings deciphered type-specific alterations in the multi-kingdom microbiota and their functional profiles, providing the basis for designing precision microbial therapies aimed at improving long-term outcomes for children with SBS.

**IMPORTANCE:**

Pediatric short bowel syndrome (SBS) is a primary cause of intestinal failure, yet prior research characterizing the gut microbiota has focused almost exclusively on bacteria. In this study, we characterized the multi-kingdom microbiome (including bacteria, fungi, archaea, and viruses) across the three anatomical types of pediatric SBS. We found that different SBS subtypes showed distinct microbial patterns: SBS I was enriched in pathogens, SBS II exhibited a depletion of beneficial short-chain fatty acid-producing species, and SBS III was characterized by a loss of bile acid-metabolizing microbes with an expansion of *Lactobacillus*. Functional analysis showed that SBS I had markedly suppressed core metabolic pathways, and integrated analyses identified the ileocecal valve as a key determinant of microbial gene profiles, with its loss linked to impaired secretion and liver injury. These findings provide a comprehensive multi-kingdom view of the pediatric SBS microbiome and highlight anatomical determinants shaping host–microbiome dysfunction.

## INTRODUCTION

Short bowel syndrome (SBS) is a severe clinicopathological condition characterized by a significant reduction in functional intestinal length, typically resulting from surgical resection or congenital malformations (1). While it can occur at any age, SBS is more frequent in infants and children, especially those with congenital intestinal malformations or severe gastrointestinal diseases (2, 3). In the United States, the prevalence of pediatric SBS is estimated to be 1.1% among extremely low birth weight infants, with necrotizing enterocolitis representing the leading cause (4). Patients with SBS often require long-term parenteral nutrition (PN) to support growth and development; however, extended PN dependence is associated with severe complications such as central line-associated bloodstream infections and intestinal failure-associated liver disease (3).

Based on the anatomical configuration of the remaining intestine, SBS can be classified into three types: SBS I (end-jejunostomy), SBS II (jejuno-colic), and SBS III (jejuno-ileal anastomosis) (2). Among SBS types, SBS III has the most favorable outcome with minimal PN dependence and maximal absorption, and SBS I has the poorest outcome with limited absorption and long-term PN dependence (2). SBS II shows an intermediate prognosis. While anatomical phenotype provides a baseline estimate of prognosis, it fails to capture the real-time status of intestinal functional recovery, and the duration of PN dependence varies greatly even among patients with the same type.

Emerging evidence suggests that the gut microbiota plays a crucial role in regulating intestinal adaptation, nutrient absorption, mucosal integrity, and immune homeostasis (5). Dysbiosis is closely linked to impaired intestinal function and long-term PN dependence, with microbial diversity being further shaped by factors such as medication use, nutritional support, infections, feeding status, and age (6, 7). In the pediatric population, Budinska et al. (8) compared the gut microbiota profile in children with PN-dependent SBS I and SBS II along with PN-independent SBS patients. They found that SBS I patients exhibited an enrichment of oxygen-tolerant microbes and a depletion of strict anaerobes. While current studies on gut microbiota in pediatric SBS have focused mainly on bacteria, the roles of fungi, archaea, and viruses remain largely unexplored. Furthermore, the distinct microbial signatures across the three anatomical types have not yet been reported.

In this study, we performed deep shotgun metagenomic sequencing to systematically characterize the gut bacteria, fungi, archaea, viruses, and their associated functional genes across different types of pediatric SBS. We further integrated multi-kingdom taxonomic data with functional gene and pathway profiles to evaluate their associations with key clinical parameters. Collectively, our findings provide a comprehensive landscape of multi-kingdom gut microbiota and functional alterations in different types of pediatric SBS.

## MATERIALS AND METHODS

### Patient cohorts

A cross-sectional study was conducted. A total of 34 pediatric patients diagnosed with SBS were recruited from the Division of Pediatric Gastroenterology and Nutrition at Xinhua Hospital Affiliated to Shanghai Jiao Tong University School of Medicine, including 8 with SBS I, 15 with SBS II, and 11 with SBS III. Inclusion criteria for SBS patients were (i) a confirmed diagnosis of SBS according to established clinical criteria (9); (ii) current status within the intestinal adaptation (compensatory) phase; and (iii) age between 0 and 12 years. Additionally, 26 age- and sex-matched healthy children were recruited from the local community as controls. Exclusion criteria for controls included (i) a history of gastrointestinal or systemic disorders, (ii) prior abdominal surgery, and (iii) use of antibiotics or probiotics within 3 months before fecal sample collection.

Complete medication and medical histories were collected at admission. Demographic, clinical, and nutritional parameters were systematically recorded. The data included birth weight, gestational age (GA), age in months, sex, prematurity status, use of antibiotics, probiotics, proton pump inhibitors (PPI), etiology of SBS, residual small intestine length, preservation of the ileum and ileocecal valve (ICV), duration of PN (defined as the time interval from the initiation of PN to the date of fecal sample collection in this study), height, weight, oral energy intake, and macronutrient intake. Laboratory parameters included white blood cell (WBC) count, C-reactive protein (CRP), and a biochemistry profile comprising albumin, liver function (total bile acid [TBA], alanine aminotransferase [ALT], aspartate aminotransferase [AST], alkaline phosphatase [AKP], gamma-glutamyl transferase [GGT], total bilirubin [TB], and direct bilirubin [DB]), renal function (blood urea nitrogen [BUN], serum creatinine [SCr], and uric acid [UA]), and nutrients (copper [Cu], zinc [Zn], calcium [Ca], magnesium [Mg], iron [Fe], and vitamin D [VD]). Complete blood count was performed using an automated hematology analyzer (LH750; Beckman Coulter Inc., Brea, CA, USA). CRP was determined by an immunoturbidimetric assay (QuikRead go; Orion Diagnostica, Espoo, Finland). Liver and renal functions were analyzed using a Hitachi 7600-120 automatic analyzer (Hitachi, Tokyo, Japan; reagents from Asahi Kasei Pharma Corporation). Nutrients (except VD) were measured by atomic absorption spectrometry (Bohui Co., Beijing, China), while VD levels were quantified using an enzyme-linked immunosorbent assay kit (Jining Co., Shanghai, China).

### Sample collection, transportation, and storage

To minimize sampling variability, parents and legal guardians received standardized instructions prior to sample collection. For healthy controls and patients with SBS II and SBS III, fresh spontaneously passed fecal samples were collected immediately after defecation using sterile spoons from clean diapers or sterile containers, strictly avoiding environmental contamination. For patients with SBS I, fresh stoma effluent was collected immediately after the replacement of sterile ostomy bags. All samples were immediately placed on dry ice for transportation and subsequently stored at −80°C with strict avoidance of freeze-thaw cycles prior to DNA extraction.

### DNA extraction and metagenomic sequencing

DNA was extracted from human stool samples using a stool genomic DNA kit (CW2092S, CWBIO, Jiangsu, China). To enhance cell lysis efficiency and maximize the release of DNA from microbial cells, we optimized the protocol as previously described (10). Briefly, 200 mg of fecal sample was transferred into a grinding tube, followed by the addition of 1 mL of release buffer SW. The mixture was vortexed and subsequently heated. After centrifugation, the supernatant was discarded. The pellet was resuspended in 600 µL buffer SL containing lysozyme, proteinase K, and a mixture of glass beads (0.1 and 0.4–0.6 mm). The mixture was vortexed using a high-speed homogenizer (FastPrep-24 5G, MP Biomedicals), centrifuged, and the supernatant was collected. Subsequently, 600 µL of Buffer GL was added to the supernatant, and the mixture was incubated in a water bath at 80°C for 15 min. Ethanol was then added and mixed thoroughly, after which the mixture was transferred to an adsorption column and centrifuged. The remaining procedures were performed according to the manufacturer’s instructions.

Approximately 1 µg of genomic DNA from each sample was sheared to an average size of 350 bp using a Covaris system. Sequencing libraries were generated through a series of steps, including end repair, A-tailing, adapter ligation, purification, and PCR enrichment. Fragment size distribution and integrity were checked with AATI analysis, and libraries meeting the expected insert size were quantified by qPCR (>3 nM). Libraries that passed quality control were pooled according to concentrations and sequenced on the Illumina NovaSeq 6000 platform (paired-end 150 bp), achieving a target depth of 10 Gb per sample.

Raw reads were processed with KneadData (v0.12.0) to retain high-quality microbial sequences while filtering out potential contaminants. Raw paired-end reads were quality-filtered using fastp (v0.21.0) (11) to remove adapter sequences, trim low-quality bases (*Q* < 20), and discard reads shorter than 50 bp. Reads were aligned to multiple reference genomes, including human (GRCh38 and CHM13), mouse (mm10), dog (canFam4), chicken (Galgal4), bacterial plasmids, complete plastomes, and UniVec sequences, using Bowtie2 (v2.4.2) (12). Reads mapping to these references were removed, and the remaining high-quality microbial reads were retained for downstream metagenomic analysis. Read counts before and after filtering were recorded for each sample.

### Microbial taxonomic and functional profiling

Taxonomic classification of bacteria, archaea, fungi, and viruses was performed using Kraken2, a k-mer-based taxonomic classifier, as previously described (12). A customized reference database was constructed using Jellyfish (v2.3.0) by counting distinct 31-mers from reference genomes, comprising 18,756 bacterial, 359 archaeal, and 9,346 viral genomes from NCBI RefSeq (January 2020), together with 1,094 fungal genomes obtained from NCBI RefSeq, FungiDB, and Ensembl (January 2020). Each k-mer in the sequencing reads was mapped to the lowest common ancestor of the matching reference genomes, and taxonomic assignment was determined by the maximum number of matching k-mers while pruning redundant branches in the taxonomy tree (13). To refine abundance estimation at the genus and species levels, Bracken (v2.8) was applied to re-estimate read counts derived from Kraken2 (14). Species-level read counts were then normalized to relative abundances for downstream analyses.

High-quality reads were *de novo* assembled into contigs with MEGAHIT (v1.2.9) using the “meta-sensitive” mode, and contigs shorter than 500 bp were discarded. Gene prediction was performed with Prodigal (v2.6.3) in metagenomic mode (-p meta). Non-redundant microbial gene catalogs were generated with CD-HIT (identity threshold = 0.95; minimal coverage = 0.9). Transcript abundance was quantified using Salmon (v1.8.0) in metagenomic mode (--meta), with index construction based on the non-redundant gene catalog. Functional annotation was carried out using EggNOG-mapper (v2.1.7) against the EggNOG (evolutionary genealogy of genes: Non-supervised Orthologous Groups) orthology database (v5.0.2). The relative abundances of EggNOG genes, KEGG Orthology (KO) groups, and pathways were summarized by aggregating the abundances of annotated genes belonging to the same functional categories.

### Co-abundance analysis of multiple kingdoms

To explore the associations among different species, FastSpar (v1.0.0) (15) was employed to construct a compositionality-corrected microbial interaction network capable of estimating correlation values from compositional data. Correlations were refined over 20 iterations, and the statistical significance of each interaction was assessed using 1,000 permutations. The median correlation strength across identical interaction pairs was calculated as the combined association value. Only correlation coefficients (|*r*|) > 0.2 and *P* < 0.005 were retained for downstream analyses. The resulting network was visualized using Gephi (v1.0.0).

### Statistical analysis

Clinical characteristics are presented as median and interquartile ranges unless stated otherwise. For comparisons between two groups, the Student’s *t*-test or Mann–Whitney *U* test was applied for highly skewed distributions. Differences across three or more groups were assessed using one-way analysis of variance, followed by Tukey’s *post hoc* test for normally distributed data, or the Kruskal-Wallis test with Dunn’s *post hoc* test for nonparametric data, followed by false discovery rate (FDR) correction for multiple comparisons. Categorical variables were compared using Fisher’s exact test.

Alpha (α) diversity was calculated for each sample across the four microbial kingdoms. Microbial community similarities were evaluated by principal coordinates analysis (PCoA) based on Bray–Curtis distances, and group differences were assessed using permutational multivariate analysis of variance (PERMANOVA) with 999 permutations (vegan package, R v4.4.3) (16). Differential abundance was analyzed using analysis of compositions of microbiomes with bias correction 2 (ANCOM-BC2) (17, 18) (implemented in the R package ANCOMBC) and multivariable association with linear models 2 (MaAsLin2) (19, 20) (implemented in the R package Maaslin2). Multiple comparisons were corrected using the Holm-Bonferroni method for ANCOM-BC2 and the Benjamini-Hochberg method for MaAsLin2. Both models included adjustments for significant confounders, including age, sex, and medication exposure (antibiotics, probiotics, and proton pump inhibitors).

Differentially abundant KO genes and pathways were identified using the Wilcoxon rank-sum test, with significance thresholds of FDR-adjusted *q* < 0.05 for KO genes and *P* < 0.05 for pathways. Spearman correlation was then applied to assess associations between differential KO genes and clinical indicators across SBS types.

All statistical analyses were two-tailed, with significance defined as *P* < 0.05. Data analysis and visualization were conducted in R (v4.4.3), GraphPad Prism (v8), FastSpar (v1.0.0), Gephi (v0.10.1), and the Bioinfo Intelligent Cloud (BIC; https://www.bic.ac.cn/BIC/).

### STORMS checklist

This study was conducted according to the STORMS checklist (DOI: https://doi.org/10.5281/zenodo.21286945).

## RESULTS

### Study population

Sixty children were included, consisting of 26 controls and 34 SBS patients (8 SBS I, 15 SBS II, and 11 SBS III). The median ages of the controls, patients with SBS I, II, and III were 15, 6, 15, and 8 months, respectively (*P* = 0.003). Similarly, the median body weights varied across the SBS types, with 4.2 kg in SBS I, 9.8 kg in SBS II, and 6.8 kg in SBS III (*P* = 0.015). The median GA did not differ significantly among SBS subtypes—35.0, 34.0, and 36.0 weeks for SBS I–III, respectively (*P* = 0.233). Other variables—gender, birth weight, preterm birth, primary disease, residual small bowel length, and PPI use—showed no significant differences among the four groups. For biochemical indices, significant intergroup differences were identified in liver function markers, including AST (SBS I vs II vs III: 70.6 vs 48.0 vs 48.0 U/L; *P* = 0.039) and AKP (SBS I vs II vs III: 397.5 vs 349.0 vs 223.0 U/L; *P* = 0.026), as well as serum iron levels (SBS I vs II vs III: 33.9 vs 21.0 vs 20.8 μmol/L; *P* = 0.005). Other parameters, including liver function (TBA, ALT, GGT, TB, and DB) and nutrients (Cu, Zn, Ca, Mg, iron, and VD), showed no significant differences among the four groups (Table 1).

**TABLE 1:** Clinical characteristics of healthy controls and patients with type I, II, and III short bowel syndrome^*a*^

Characteristic	Control (*n* = 26)	SBS I (*n* = 8)	SBS II (*n* = 15)	SBS III (*n* = 11)	*P* value
Sex					0.56
Male	13 (50%)	5 (63%)	5 (33%)	6 (55%)	
Female	13 (50%)	3 (38%)	10 (67%)	5 (45%)	
BW (g)	–	2,925 (1,340, 3,250)	1,800 (1,150, 2,750)	2,700 (1,895, 3,042)	0.3
Age (months)	15 (9, 24)	6 (3, 7)	15 (7, 54)	8 (7, 17)	**0.003**
Preterm_birth (*n*)		6	13	11	0.248
GA (weeks)	–	35.0 (30.0–38.8)	34.0 (30.0–37.0)	36.0 (34.0–39.0)	0.233
Protopathy					0.414
CSBS	–	1 (13%)	0 (0%)	3 (27%)	
NEC	–	2 (25%)	4 (27%)	1 (9.1%)	
Intestinal atresia ‌	–	2 (25%)	2 (13%)	4 (36%)	
Volvulus	–	1 (13%)	5 (33%)	2 (18%)	
Ileus	–	0 (0%)	2 (13%)	0 (0%)	
Others	–	2 (25%)	2 (13%)	1 (9.1%)	
Antibiotics					0.434
No	–	0 (0%)	4 (27%)	2 (18%)	
Yes	–	8 (100%)	11 (73%)	9 (82%)	
Probiotics					0.398
No	–	6 (75%)	12 (80%)	6 (55%)	
Yes	–	2 (25%)	3 (20%)	5 (45%)	
PPIs					0.092
No	–	1 (13%)	8 (53%)	2 (18%)	
Yes	–	7 (88%)	7 (47%)	9 (82%)	
PN_duration (days)	–	148 (98.5, 204)	240 (171, 465)	199 (136, 307)	0.052
Remaining small intestine (cm)	–	51 (40, 57.5)	60 (35, 70)	52 (35, 62)	0.524
Remain_ileum					0.119
No	–	1 (17%)	4 (33%)	0 (0%)	
Yes	–	5 (83%)	8 (67%)	11 (100%)	
Ileocecal_valve					
No		8 (100.0%)	15 (100.0%)	0 (0.0%)	**<0.001**
Yes		0 (0.0%)	0 (0.0%)	11 (100.0%)	
Weight (kg)	–	4.2 (3.0, 6.3)	9.8 (5.5, 16.5)	6.8 (3.8, 8.0)	**0.015**
Height (cm)	–	62.0 (54.5, 65.5)	75.0 (61.0, 103.0)	67.0 (59.0, 72.0)	0.058
Intake of macronutrients from EN	–				
Carbohydrate (% Kcal)	–	44.1 (44.1, 47.4)	44.1 (28.0, 45.8)	44.1 (28.0, 44.1)	0.228
Protein (% Kcal)	–	10.7 (10.5, 11.2)	10.7 (8.0, 11.3)	10.7 (8.0, 10.7)	0.252
Fat (% Kcal)	–	44.9 (44.1, 47.0)	43.1 (30.6, 44.9)	44.9 (30.6, 44.9)	0.131
Solid foods					**<0.001**
No	3 (12%)^a^	6 (75%)^b^	7 (47%)^b^	8 (73%)^b^	
Yes	23 (88%)^a^	2 (25%)^b^	8 (53%)^b^	3 (27%)^b^	
Laboratory tests					
CRP (mg/L)	–	1.5 (1.0, 8.0)	1.0 (1.0, 6.0)	1.0 (1.0, 1.0)	0.4
WBC (10^9^)	–	9.6 (8.7, 11.5)	8.9 (5.7, 12.5)	9.4 (5.5, 11.1)	0.754
Albumin (g/L)	–	36.5 (34.2, 42.3)	40.8 (37.0, 43.4)	40.8 (37.4, 43.8)	0.450
Prealbumin (mg/L)	–	214 (191, 268)	249 (125, 302)	169 (124, 234)	0.196
BUN (mmol/L)	–	3.89 (3.10, 4.66)	4.70 (3.64, 6.08)	4.80 (3.66, 5.39)	0.311
SCr (μmol/L)	–	17.0 (13.7, 19.5)	16.7 (16.0, 22.2)	15.9 (13.0, 18.5)	0.345
UA (μmol/L)	–	145 (119, 182)	116 (94, 196)	151 (141, 189)	0.557
TBA (μmol/L)	–	7.2 (4.0, 19.4)	4.2 (2.1, 12.4)	5.7 (4.2, 17.4)	0.709
ALT (U/L)	–	91.0 (74.0, 150.0)	41.0 (29.0, 104.0)	38.0 (30.0, 68.0)	0.107
AST (U/L)	–	70.6 (65.5, 123.2)	48.0 (40.0, 87.0)	48.0 (37.0, 65.7)	**0.039**
AKP (U/L)	–	397.5 (325.5, 500.0)	349.0 (274.0, 776.0)	223.0 (160.0, 344.0)	**0.026**
GGT (U/L)	–	105.5 (55.0, 191.0)	28.0 (16.0, 118.0)	19.0 (13.0, 43.0)	0.062
TB (μmol/L)	–	12.3 (6.6, 57.6)	6.7 (5.4, 16.0)	5.6 (3.7, 10.8)	0.142
DB (μmol/L)	–	3.5 (0.5, 12.3)	1.6 (0.0, 4.7)	1.0 (0.0, 3.5)	0.425
Cu (μmol/L)	–	12.8 (11.4, 14.0)	12.7 (12.1, 17.4)	16.2 (11.7, 17.1)	0.526
Zn (μmol/L)	–	18.8 (15.4, 25.4)	16.9 (11.1, 24.5)	12.6 (10.6, 17.8)	0.321
Ca (mmol/L)	–	2.4 (2.3, 2.5)	2.4 (2.2, 2.5)	2.4 (2.4, 2.5)	0.180
Mg (mmol/L)	–	0.7 (0.7, 0.9)	0.8 (0.7, 0.9)	0.9 (0.8, 0.9)	0.059
Fe (μmol/L)	–	33.9 (31.3, 44.7)	21.0 (16.3, 28.8)	20.8 (15.2, 24.0)	**0.005**
VD (nmol/L)	–	69.0 (38.6, 89.3)	58.1 (44.3, 76.3)	60.0 (58.3, 85.9)	0.664

^*a*^ Values are presented as median and interquartile range for continuous variables and number and percentage for categorical variables. Categorical variables were compared using Fisher’s exact test. The comparisons of solid foods used the Fisher-Freeman-Halton exact test, with *post hoc* pairwise comparisons adjusted via Bonferroni correction (*P* < 0.0083). Continuous variables were compared using the Kruskal-Wallis rank-sum test. Bold font indicates significance (*P *≤ 0.05). The symbol “–” indicates data not collected for healthy controls or not applicable. AKP, alkaline phosphatase; ALT, alanine aminotransferase; AST, aspartate aminotransferase; BUN, blood urea nitrogen; BW, birth weight; Ca, calcium; CRP, C-reactive protein; CSBS, congenital short bowel syndrome; Cu, copper; DB, direct bilirubin; EN, enteral nutrition; Fe, iron; GA, gestational age; GGT, gamma-glutamyl transferase; ICV, ileocecal valve; Mg, magnesium; NEC, necrotizing enterocolitis; PN, parenteral nutrition; PPI, proton pump inhibitor; SCr, serum creatinine; TB, total bilirubin; TBA, total bile acid; UA, uric acid; VD, Vitamin D; WBC, white blood cell; and Zn, zinc. Different superscript letters indicate significant differences between groups; groups sharing the same superscript letters are not significantly different.

In subsequent pairwise comparisons (Table S1), we further examined specific differences in clinical and treatment-related factors. While age and body weight showed inherent variations among the study groups, the duration of PN was found to be significantly longer in patients with SBS I compared to those with SBS II (*P* = 0.013). Significant differences were also observed in medication profiles. Furthermore, probiotic administration showed group-specific patterns, with a significant increase observed in SBS II (*P* = 0.043) and SBS III (*P* = 0.001) relative to controls. No significant differences were observed in any of the other pairwise comparisons.

### Characterization of SBS-associated gut microbiota in four kingdoms

**Fig 1:**
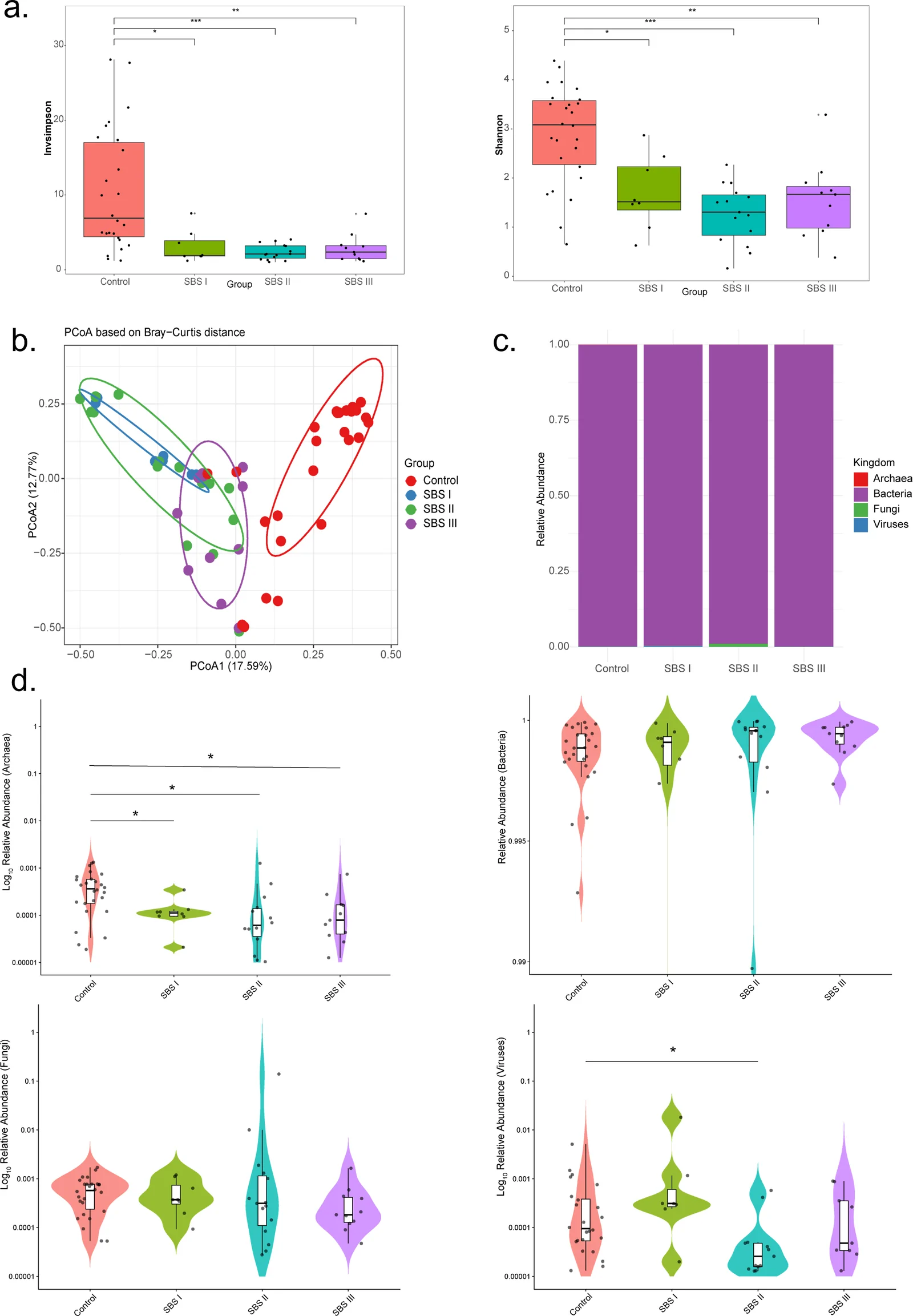
Decreased microbial diversity and altered gut microbiota composition in children with SBS. (**a**) Alpha diversity was estimated based on the Shannon and Inverse Simpson indices among the controls (*n* = 26), children with SBS I (*n* = 8), SBS II (*n* = 15), and SBS III (*n* = 11). (**b**) Principal coordinates analysis of samples from four groups based on Bray–Curtis distance. *P* values of beta diversity based on Bray–Curtis distance were calculated with PERMANOVA by 999 permutations (two-sided test). (**c**) Kingdom-level gut microbiota composition in different types of SBS patients compared with controls. Bar plots represent the relative abundance of the four microbial kingdoms—bacteria, fungi, archaea, and viruses. (**d**) The relative abundance of bacteria, fungi, archaea, and viruses at the kingdom level among different groups. *Adjusted *P* < 0.05, **adjusted *P* < 0.01, and ***adjusted *P* < 0.001.

By deep metagenomic sequencing, we generated an average of 37,977,281 raw reads per sample (minimum = 26,615,959; maximum = 49,302,224; median = 37,221,754), identifying 11,026 bacterial, 577 fungal, 1,118 archaeal, and 13,982 viral species. Based on the Shannon and Iinverse Simpson indices, α-diversity was decreased across all three SBS types, with no significant differences observed among them (Fig. 1a). Furthermore, PCoA revealed distinct clustering between controls and SBS patients, and PERMANOVA testing confirmed significant compositional differences between SBS I and SBS III (*P* = 0.001; Fig. 1b). This clustering pattern suggests that the microbial remodeling in SBS is not primarily driven by sampling site (stomal effluent vs fecal output) but rather by the functional status of the residual intestine. Overall, compositional alterations across all four kingdoms were observed in SBS patients compared with controls (Fig. 1c; Fig. S1a and b). While the relative abundances of bacteria and fungi remained comparable across the four groups, viral abundance was significantly lower in the SBS II group than in controls. Notably, archaeal abundance was consistently depleted across all SBS types (Fig. 1d).

**Fig 2:**
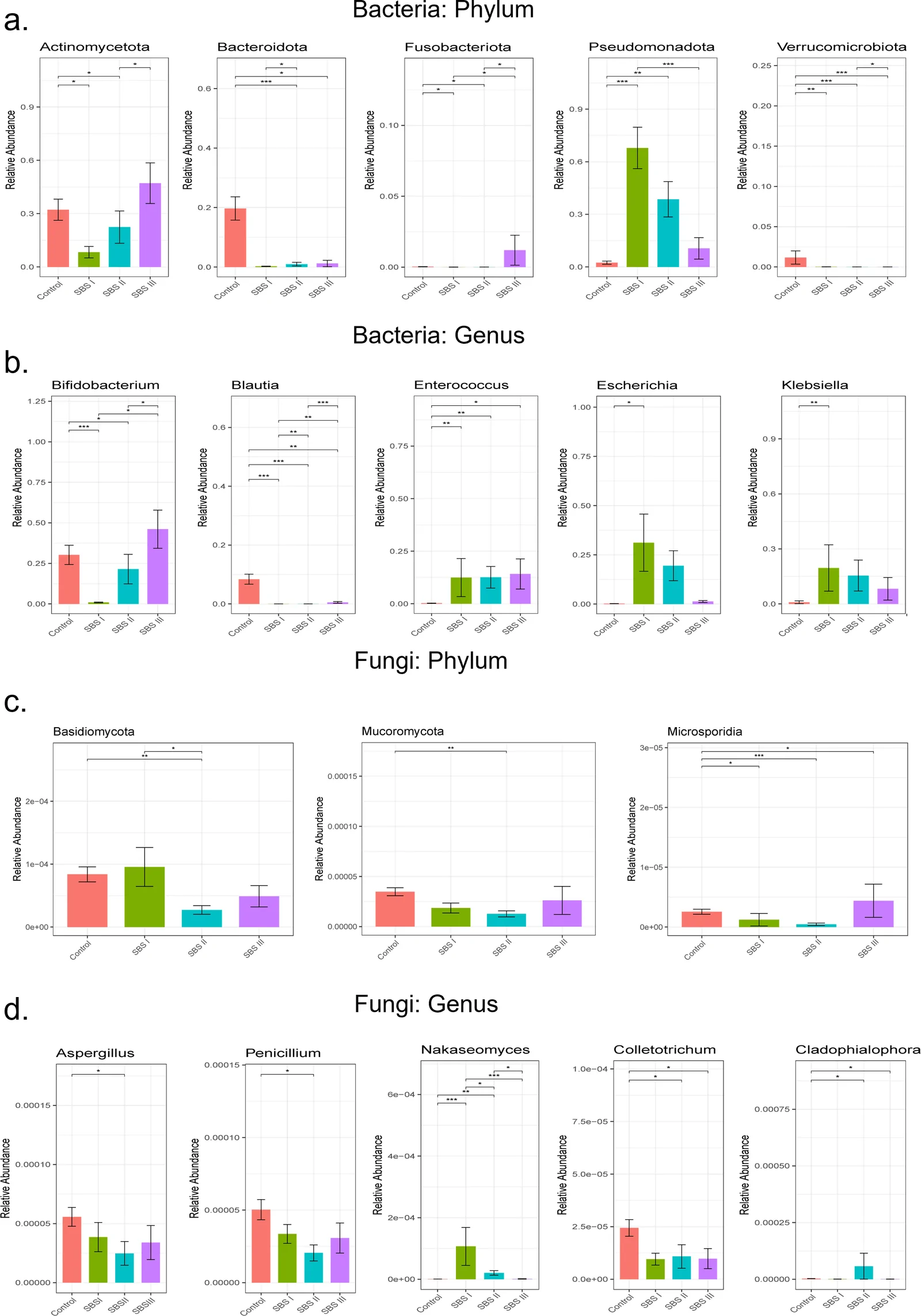
Microbial composition at the phylum and genus levels for bacteria and fungi in controls and children with SBS. Bar plots show the relative abundance of differentially abundant bacterial phyla (**a**) and genera (**b**), as well as fungal phyla (**c**) and genera (**d**) among the top five most abundant taxa. *Adjusted *P <* 0.05, **adjusted *P* < 0.01, and ***adjusted *P* < 0.001.

Next, we analyzed the differential microbes at the phylum and genus level for the four kingdoms. Within the bacterial kingdom, the top five most abundant differential phyla and genera were identified to characterize group-specific microbial shifts (Fig. 2a and b). SBS I and SBS II exhibited significant reductions in Actinomycetota, Bacteroidota, Verrucomicrobiota, and Fusobacteriota, accompanied by an enrichment of Pseudomonadota. In contrast, SBS III was primarily characterized by a depletion of Verrucomicrobiota (Fig. 2a). At the genus level, SBS I and SBS II showed decreased abundances of *Bifidobacterium* and *Blautia*, along with enrichment of *Enterococcus*. Additionally, SBS I demonstrated increased levels of *Escherichia* and *Klebsiella*. Conversely, SBS III displayed reduced *Blautia* and increased *Enterococcus* levels, with higher levels of *Bifidobacterium* and *Blautia* compared with SBS I and SBS II (Fig. 2b).

**Fig 3:**
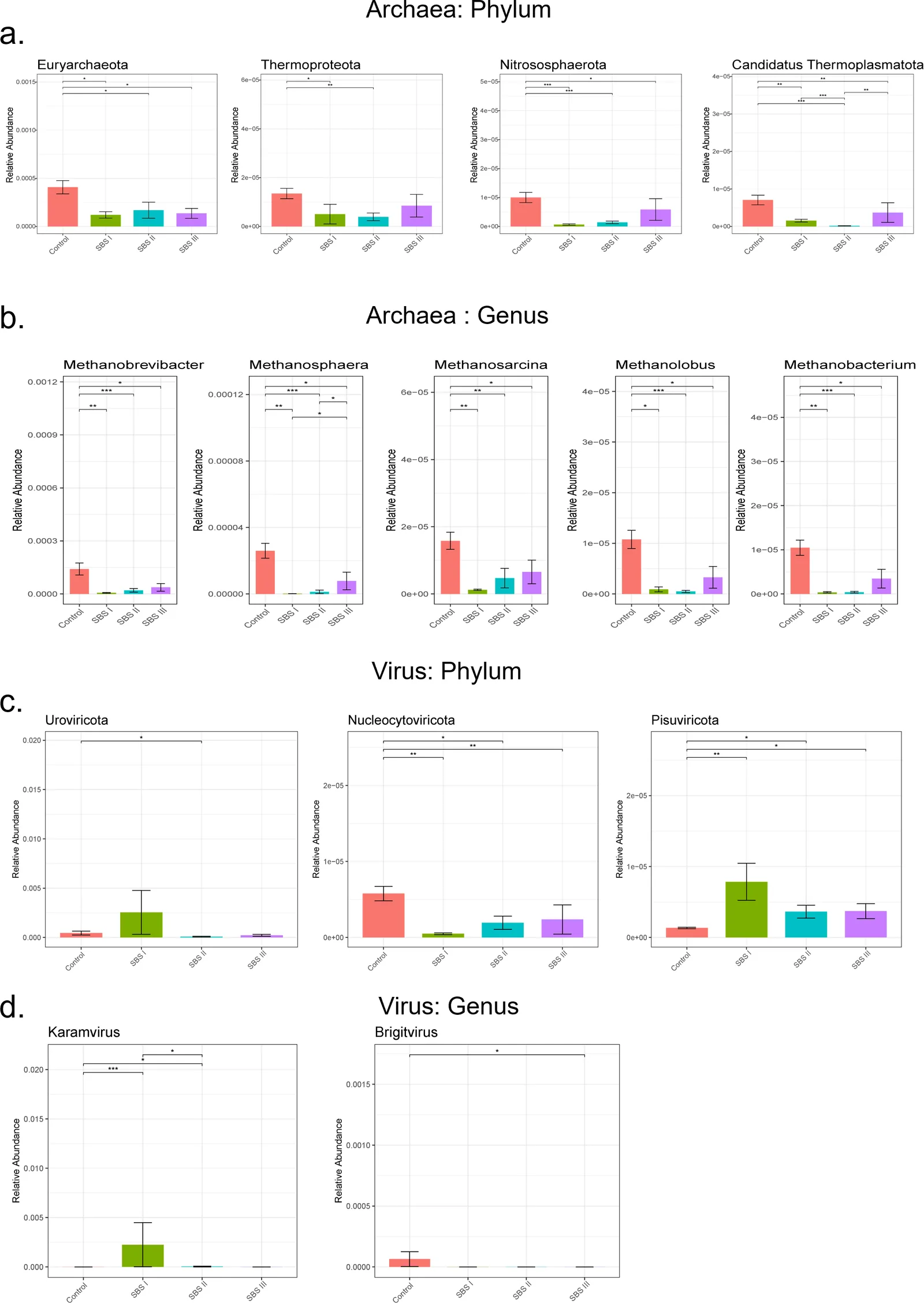
Microbial composition at the phylum and genus levels for archaea and viruses in controls and children with SBS. Bar plots show the relative abundance of differentially abundant archaeal phyla (**a**) and genera (**b**), as well as viral phyla (**c**) and genera (**d**) among the top five most abundant taxa. *Adjusted *P* < 0.05, **adjusted *P* < 0.01, and ***adjusted *P* < 0.001.

Differentially abundant taxa were also identified for fungal, archaeal, and viral communities (Fig. 2c, d, and 3). Among fungi, SBS II exhibited reductions in Basidiomycota, Mucoromycota, and Microsporidia, whereas Microsporidia abundance increased in SBS III but decreased in SBS I. At the genus level, distinct compositional patterns were observed across SBS types. In SBS I, the pathogenic genus *Nakaseomyces* was enriched, while *Aspergillus*, *Penicillium*, and *Colletotrichum* were depleted. SBS II showed enrichment of both *Cladophialophora* and *Nakaseomyces*, whereas SBS III displayed decreased *Colletotrichum* and elevated *Cladophialophora*, with *Nakaseomyces* levels remaining lower than in SBS I and SBS II. Among archaea, Euryarchaeota, Nitrososphaerota, and Candidatus Thermoplasmatota were significantly decreased in all types, while Thermoproteota levels were reduced in both SBS I and SBS II. At the genus level, a widespread depletion of *Methanobrevibacter*, *Methanosphaera*, *Methanosarcina*, *Methanolobus*, and *Methanobacterium* was observed in all SBS types. For viruses, Nucleocytoviricota abundance decreased and Pisuviricota abundance increased across all SBS groups, whereas Uroviricota specifically declined in SBS I. At the genus level, *Karamvirus* was enriched in SBS I and SBS II, whereas *Brigitvirus* was reduced in SBS III. These results reveal that SBS-associated dysbiosis is a multi-kingdom phenomenon, involving not only bacteria but also fungi, archaea, and viruses, with each group displaying unique type-specific signatures.

### Intergroup analysis of differential species between SBS types and controls

**Fig 4:**
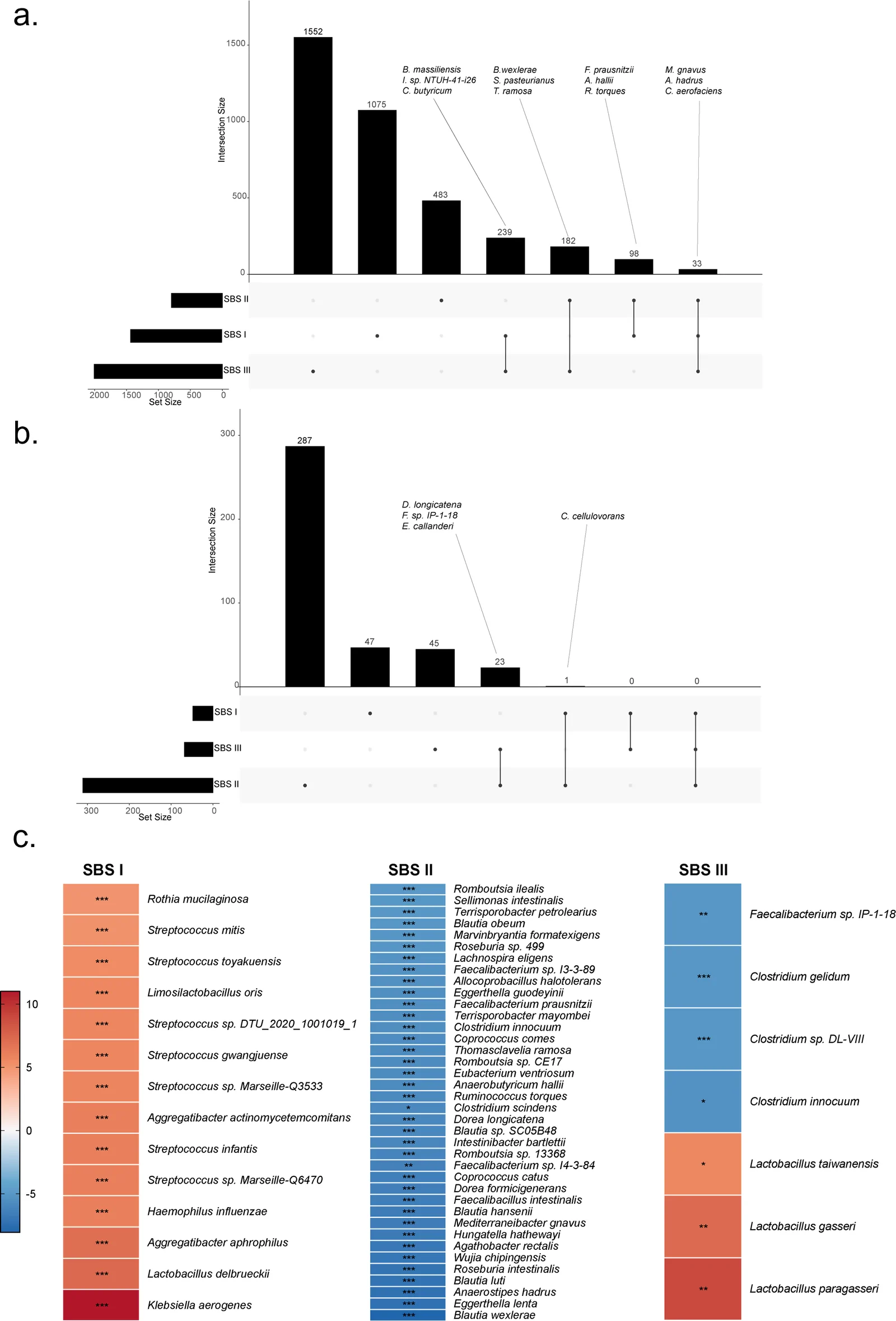
Identification of SBS-associated differential species in multi-kingdom microbiota. UpSet plots depicting differential species identified by ANCOM-BC2 (**a**) and MaAsLin2 (**b**) by comparing SBS types (SBS I, SBS II, and SBS III) and controls. Column labels indicate the number of differential species; set sizes on the left denote the total identified in each type–control comparison; and connected dots show species shared across multiple comparisons. (**c**) Heatmap illustrating differential species shared by ANCOM-BC2 and MaAsLin2 by comparing all SBS types and controls. The color intensity represents the log_2_ fold change (log_2_FC). Significance levels are indicated as *adjusted *P* < 0.05, **adjusted *P* < 0.01, and ***adjusted *P* < 0.001.

To identify differentially abundant species, we applied the ANCOM-BC2 method to analyze microbial composition at the species level, adjusting for significant confounders. Compared with controls, several species, including *Mediterraneibacter gnavus*, *Anaerostipes hadrus*, and *Collinsella aerofaciens*, exhibited a consistent reduction across all three types; *Faecalibacterium prausnitzii*, *Anaerobutyricum hallii*, and *Ruminococcus torques* were reduced in both SBS I and SBS II groups; *Blautia wexlerae*, *Streptococcus pasteurianus*, and *Thomasclavelia ramosa* were declined in SBS II and SBS III; and *Blautia massiliensis*, *Intestinibacillus* sp*.* NTUH-41-i26, and *Clostridium butyricum* were decreased in SBS I and SBS III. Across the SBS types, 1,075, 483, and 1,552 unique differential species were identified in SBS I, SBS II, and SBS III, respectively (Fig. 4a; Table S2).

Considering that ANCOM-BC2 served as the primary method for differential taxa analysis but may have certain limitations in result reproducibility and cross-data set consistency (20), MaAsLin2 was additionally applied as a complementary approach to support and refine the ANCOM-BC2-based results. Relative to the control group, *Clostridium cellulovorans* was decreased in both SBS I and SBS II; *Dorea longicatena*, *Faecalibacterium* sp*.* IP 1.18, and *Eubacterium callanderi* were consistently reduced in both SBS II and SBS III. Analysis across SBS types revealed 47, 287, and 45 unique differential species for SBS I, SBS II, and SBS III, respectively (Fig. 4b; Table S3).

To identify type-specific species, we implemented stringent filters (|log_2_ fold change | > 5 and adjusted *P* < 0.05) for the shared differential species identified by ANCOM-BC2 and MaAsLin2, yielding 14, 38, and 7 species for SBS I, SBS II, and SBS III, respectively. Distinct microbial signatures were identified across the SBS types: SBS I was characterized by an enrichment of pathogenic bacteria, including *Klebsiella aerogenes*, *Haemophilus influenzae*, and various species within the *Streptococcus* genus; SBS II exhibited a significant depletion of beneficial short-chain fatty acid (SCFA)-producing species, such as *Blautia wexlerae*, *Faecalibacterium prausnitzii*, and *Roseburia intestinalis*; and SBS III showed a reduction in bile acid-metabolizing species (e.g., *Clostridium gelidum*) and butyrate producers (e.g., *Faecalibacterium* sp*.* IP-1-18), accompanied by a significant increase in *Lactobacillus* species, specifically *Lactobacillus gasseri, Lactobacillus paragasseri*, and *Lactobacillus taiwanensis*.

### Intergroup analysis of differential species among SBS types

To investigate the impact of the presence or absence of the colon and ICV on gut microbial composition, we conducted differential taxa analyses using ANCOM-BC2 and MaAsLin2 methods to examine species-level microbiome differences between SBS I and SBS II, and between SBS II and SBS III, adjusting for significant confounders. For the comparison between SBS I and SBS II, *Clostridium scindens*, a key bile acid 7α-dehydroxylating anaerobe that converts cholic acid to disease-associated secondary bile acids, was significantly enriched in SBS I, while *Lactobacillus isalae* was enriched in SBS II (Table S4). For the comparison between SBS II and SBS III, *Adlercreutzia hattorii* was markedly decreased in SBS III, while *Pseudomonas khazarica* and several others were significantly increased in SBS III (Table S4). Additionally, MaAsLin2 analysis detected no differential species between SBS I and SBS II but revealed multiple species between SBS II and SBS III, such as *Mediterraneibacter gnavus*, which were significantly increased in SBS III (Table S5). To further explore whether additional clinical variables might influence gut microbial composition, we next examined the effects of residual small bowel length, antibiotic exposure, and GA.

To assess whether residual small bowel length was associated with alterations in gut microbial diversity, we compared α- and β-diversity between patients with remaining small bowel length ≤55 cm and >55 cm (Fig. S2). No significant differences were observed in α-diversity metrics, including Shannon index and richness (Fig. S2a and b). Similarly, PCoA revealed a high degree of overlap between the two groups (PCoA1: 20.26% and PCoA2: 14.09%; Fig. S2c). In addition, no correlation was observed between remaining SI length and α-diversity indices, including richness, Chao1, Shannon, Simpson, and Inverse Simpson (all *P* > 0.05; Fig. S2d).

To further evaluate the potential influence of antibiotic exposure, we compared the gut microbiota composition between healthy controls and SBS children without antibiotic treatment (Fig. S3). Differences in the relative abundances of dominant taxa were observed at the phylum, family, and genus levels. In particular, *Bifidobacterium* was more abundant in the SBS group (45%) than in healthy controls (28%). We further assessed whether GA was associated with microbial diversity within the SBS cohort; however, no significant associations were observed between GA and multiple α-diversity indices, including richness, Chao1, ACE, Shannon, Simpson, and inverse Simpson (all *P* > 0.05; Table S9).

### Alterations of the multi-kingdom co-abundance network in patients with SBS

To investigate the interkingdom interactions among species across SBS types, we conducted co-abundance association analyses based on the relative abundance of differential species identified by ANCOM-BC2 and MaAsLin2, after adjusting for confounders with significant differences. Associations were retained using an absolute correlation coefficient threshold of |*r*| > 0.2 with *P* < 0.005; for SBS I, which contained a substantially larger number of species, a stricter threshold (|*r*| > 0.25) was applied to improve interpretability (Table S6).

**Fig 5:**
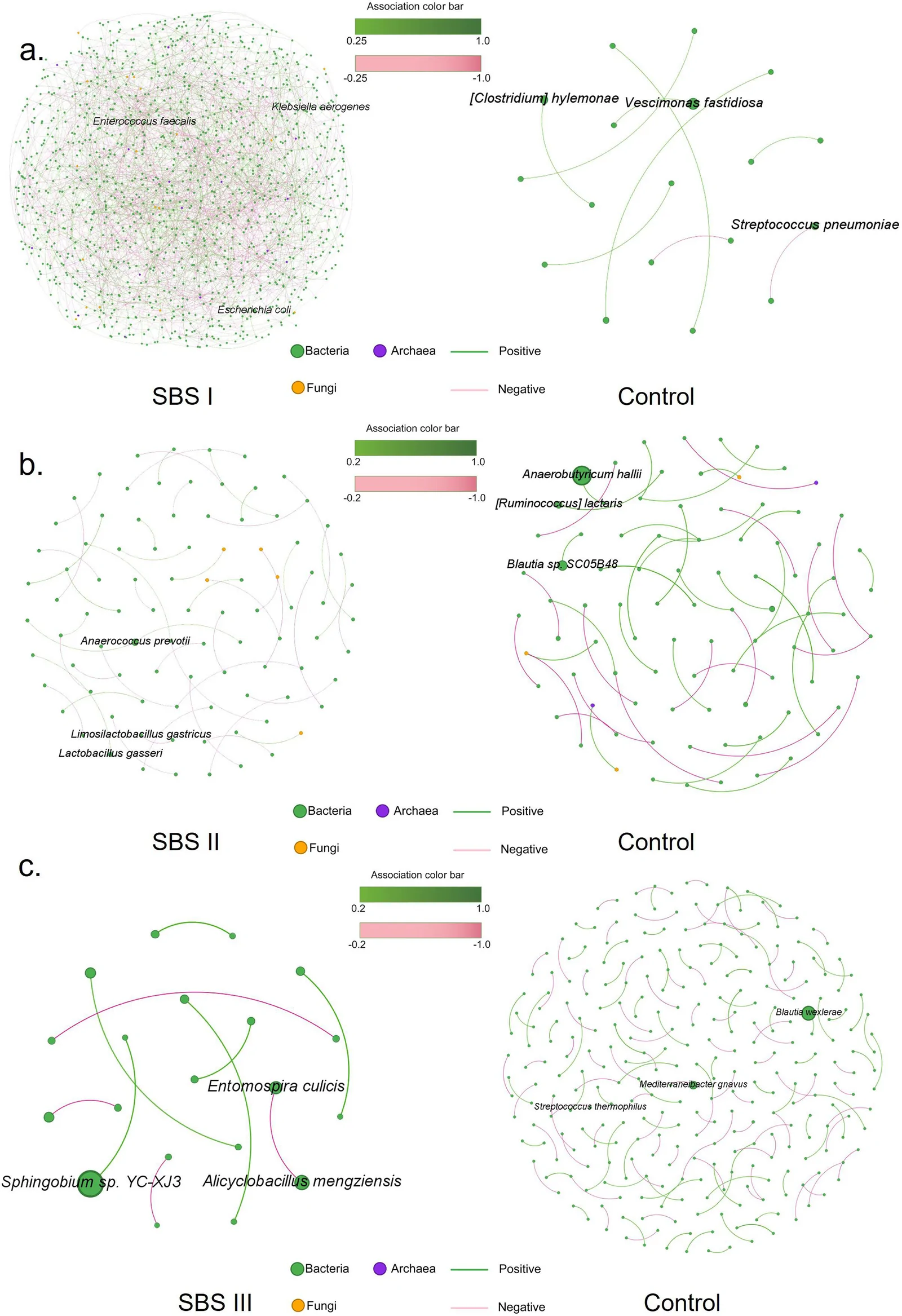
Co-abundance correlations among multi-kingdom species in patients with SBS and controls. Co-abundance networks involving combined differential species from bacteria, fungi, and archaea were constructed for SBS types and controls: (**a**) SBS I and controls, (**b**) SBS II and controls, and (**c**) SBS III and controls. The colors of nodes indicate species from bacteria (green), fungi (orange), and archaea (purple). For SBS I, only significant absolute correlations above 0.25 are shown; for SBS II and SBS III, correlations above 0.20 are shown. Green lines indicate positive species interactions, and pink lines indicate negative interactions.

Overall, the microbial co-occurrence networks of SBS types showed distinct alterations compared with their respective controls. The ecological correlations were predominantly driven by bacterial species, as viral species were not identified as differential species and were therefore excluded from the co-occurrence network analysis. Specifically, after incorporating 1,458 differential microbial species, SBS I showed a pathologically hyper-expanded and highly correlated network (1,262 species and 1,482 associations) vs controls (12 species and 6 associations). Notably, *Escherichia coli* was the most abundant species within this hyper-connected network, and it may play a crucial role in SBS I (Fig. 5a). Based on the analysis of 920 differential microbial species, SBS II displayed an intermediate level of network complexity (92 species and 48 associations), similar to its control group (88 species and 48 associations). As the highest-abundance species, the role of *Anaerococcus prevotii* in recombinant microbial communities of SBS II warrants attention (Fig. 5b). In contrast, SBS III demonstrated the simplest and least connected network (20 species and 10 associations), which was substantially less complex than that of its controls (288 species and 157 associations), an assessment based on the analysis of 2,039 differential species (Fig. 5c). Within this drastically simplified network, *Sphingobium* sp*.* YC-XJ3 was identified as the most abundant species. Critically, while SBS I and SBS II networks featured interkingdom interactions—specifically between bacteria and fungi or archaea—the SBS III network was confined exclusively to intra-kingdom bacterial associations. The notably lower number of interkingdom connections in SBS III suggests that the retained ICV preserves the small intestinal ecosystem’s structural integrity, minimizing the profound microbial shift seen in SBS I.

### Alterations of microbial function in pediatric SBS

**Fig 6:**
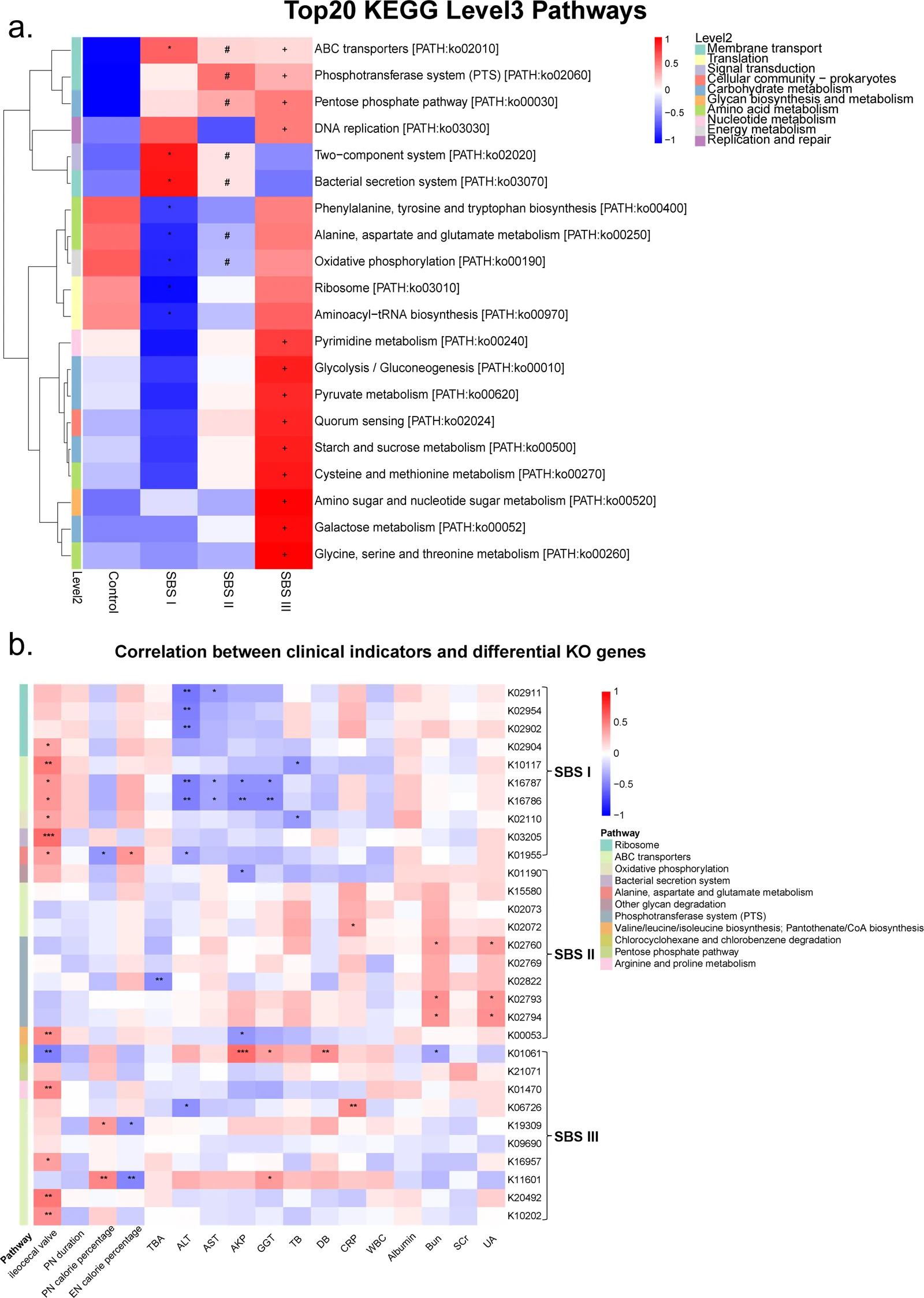
Alterations in microbial function in children with SBS. (**a**) Heatmap illustrating the distribution of the top 20 differential KEGG pathways at level 3 across the control, SBS I, SBS II, and SBS III groups, with annotations indicating significant differences: *, control vs SBS I; #, control vs SBS II; and +, control vs SBS III. (**b**) Heatmap of Spearman’s correlations between differential KO genes (top 10 KO features extracted from significantly altered pathways) and clinical indicators across SBS types. Significance levels are indicated as *adjusted *P* < 0.05, **adjusted *P* < 0.01, and ***adjusted *P* < 0.001.

Given the observed alterations in the gut microbiota composition in children with SBS, we next explored the functional changes by analyzing KO genes and pathways. Compared with controls, SBS I showed broad depletion of core metabolic pathways, including those involved in protein synthesis, nucleotide metabolism, energy production, carbohydrate metabolism, and amino acid metabolism. In addition, we found that the SBS I group showed enrichment of the ABC transporters, two-component system, and bacterial secretion system pathways, indicating increased nutrient uptake efficiency and an intensified capacity for sensing and responding to the stressed intestinal microenvironment. In SBS II, we observed moderate but significant enrichment of ABC transporters, phosphotransferase systems, and pentose phosphate pathways. Conversely, pathways related to aspartate/glutamate metabolism and oxidative phosphorylation were notably diminished, indicating a metabolic adaptation of the microbiota, prioritizing rapid carbohydrate uptake and anaerobic fermentation over complex amino acid metabolism and aerobic respiration. In contrast, SBS III was primarily characterized by an enrichment in carbohydrate and amino acid metabolism relative to the control group, suggesting a more stable environment and longer transit time when preserving the ICV and colon (Fig. 6a; Table S7).

Next, we investigated the associations between differential KO genes and clinical parameters in SBS. For each SBS type, the top 10 differential KEGG pathways were identified relative to controls, and from these pathways, we selected the top 10 most enriched KO genes for each type, yielding a total of 30 representative KO genes (Table S8). Notably, the status of the ICV emerged as the primary clinical parameter associated with microbial gene abundance, showing significant correlations with 13 of the 30 KO genes. Specifically, within the bacterial secretion system pathway, *virD4/lvhD4* (K03205, known as type IV secretion system protein VirD4) exhibited a robust positive correlation with the presence of ICV in SBS I. These data indicate that the impairment of fundamental bacterial metabolic capacity is intimately linked with the progression of host hepatic injury in SBS I. In SBS II, the positive correlation between the phosphotransferase system and renal function markers (BUN, SCr, and UA) suggests that accelerated bacterial carbohydrate fermentation may contribute to the accumulation of gut-derived metabolites, thereby increasing renal excretory burden and exacerbating potential kidney impairment. In SBS III, we observed positive correlations between ICV and genes from the ABC transporters and arginine and proline metabolism pathways. Specifically, within the ABC transporter pathway, *bcrA* (K19309, bacitracin transport system ATP-binding protein) and *mntC* (K11601, manganese transport substrate-binding protein) were positively correlated with the proportion of calories derived from PN (Fig. 6b).

## DISCUSSION

Most studies have primarily focused on gut bacterial alterations in pediatric SBS (21), while the critical roles of non-bacterial microorganisms remain unexplored. In this study, we performed a comprehensive analysis of the gut multi-kingdom using deep metagenomic sequencing in different types of pediatric SBS. We showed that the α-diversity was reduced in all SBS types, while no significant differences were detected among them. Distinct type-specific dysbiosis was identified, characterized by an influx of opportunistic pathogens in SBS I, the loss of essential SCFA producers in SBS II, and a shift toward *Lactobacillus* dominance with reduced bile acid metabolism in SBS III. The core metabolic function was profoundly impaired in SBS I patients, with marked suppression of pathways involved in energy production, amino acid metabolism, and protein synthesis.

Previous studies have shown that, compared with controls, children with SBS exhibit distinct characteristics in their gut microbiota, including a significant reduction in microbial diversity; an increased proportion of pro-inflammatory bacteria (such as Proteobacteria, particularly Enterobacteriaceae); and a marked decrease in “beneficial bacteria” (such as Bacteroidota) (6, 22–24). In line with previous findings, we showed a type-associated expansion of pathogenic bacteria and depletion of beneficial bacteria along with a decreased microbial diversity in all types of SBS. Interestingly, among fungi, the relative abundance of *Nakaseomyces* (e.g., formerly classified as *Candida glabrata*) was significantly increased in patients with SBS I and SBS II compared with those with SBS III and controls. Since this genus has also been detected in SBS and other inflammatory bowel diseases (25–28), targeting *Nakaseomyces* or its core species may help alleviate intestinal inflammation.

It is noteworthy that the patients enrolled in our study exhibited a considerable age span, and age is traditionally recognized as a primary factor influencing the maturation of the intestinal microbiota (29). However, several clinical factors inherent to SBS may have modulated this influence. All patients in this study were in the intestinal adaptation phase, a critical clinical stage characterized by the transition from PN to EN. This shared compensatory state, to a certain extent, appears to have weakened the independent impact of age on the microbial community. This is evidenced by our finding that α-diversity was significantly and consistently reduced across all SBS types, regardless of the age differences between the SBS I and SBS II groups. To ensure the scientific rigor of our findings, we employed robust statistical models (ANCOM-BC2 and MaAsLin2) to adjust for age as a confounder. The most significant changes in gut microbiota composition occur during the dietary transition from milk to a diet that includes other foods (30). Due to severely impaired absorptive function, SBS patients typically had a low and limited variety of complementary food intake, which likely hindered the normal age-related diversification of the gut microbiota.

Anatomically, despite partial colon preservation in SBS II relative to SBS I, the characteristic accelerated intestinal transit in SBS patients, coupled with the loss of ICV function in SBS II cases, blurs the microbiological distinction between the distal small bowel and colon, resulting in compositional convergence of their respective microbiomes (6). The key anatomical distinction between SBS II and SBS III is the presence or absence of the ICV. Previous research has demonstrated that even in non-SBS patients, ICV resection could contribute to intestinal dysbiosis. This condition is characterized by the loss of beneficial taxa, such as Lachnospiraceae and *Akkermansia*, coupled with an expansion of potentially pathogenic species, including *Clostridium* and *Enterococcus* (31). Huang et al. (24) demonstrated that the ICV exerts a profound influence on microbial community composition, revealing distinct intestinal microenvironments between anatomical SBS II and SBS III. They found that SBS II was characterized by an expansion of Proteobacteria, while SBS III was characterized by a predominance of *Lactobacillus*. Our study revealed pronounced differences in gut microbiota composition between SBS II and SBS III. In particular, SBS II exhibited a significant depletion of beneficial taxa, such as *Blautia wexlerae*, along with an increased abundance of potentially pathogenic bacteria, such as *Pseudochrobactrum algeriensis*. Consistent with previous reports, these findings further highlight the pivotal role of the ICV in preserving intestinal microbial homeostasis.

Consistent with previous findings (24, 32), our study demonstrates that SBS displays marked functional depletion across key metabolic pathways, particularly those involved in carbohydrate metabolism (e.g., glycolysis/gluconeogenesis and the pentose phosphate pathway), amino acid metabolism, and energy metabolism. Type-resolved analyses revealed that the magnitude of this metabolic suppression correlated strongly with disease severity—being most profound in SBS I, moderate in SBS II, and reversed in SBS III. Strikingly, several metabolic pathways in SBS III showed partial recovery or even modest enhancement relative to controls, suggesting the presence of a compensatory functional shift during later stages of adaptation. Future studies should focus on identifying the molecular drivers of this compensatory response to better understand the mechanisms underlying intestinal adaptation.

Our differential gene profiling establishes anatomical integrity and nutritional status as the dominant forces shaping the functional landscape of the microbiome in pediatric SBS. The enrichment of *virD4/lvhD4* in patients retaining an ICV suggests that the ICV preserves a critical ecological niche, sustaining complex host interaction machinery that is depleted in resected phenotypes. Notably, the *ecfA1/A2* complex appears to modulate the gut-liver axis; the inverse relationship between these transporters and liver enzymes implies that efficient microbial nutrient scavenging may help mitigate hepatic stress during intestinal failure. Regarding nutritional recovery, stress- and transport-related genes (*bcrA* and *mntC*) tracked with higher parenteral intake, whereas *carB* abundance indicated reduced needs, underscoring its pivotal role in successful adaptation.

This study has several limitations. First, due to its retrospective design, detailed information on dietary composition and intake was not available, which precluded us from assessing the potential impact of dietary factors on gut microbiota composition. Second, given the substantial heterogeneity in the type, duration, and route of antibiotic administration among patients, antibiotic exposure was only analyzed as a binary variable. Third, all healthy controls were full-term infants, whereas most SBS patients were preterm, resulting in a marked imbalance in GA distribution between the two groups and limiting its inclusion in multivariable models. In addition, we acknowledge that differences in sample origin between controls and SBS patients (e.g., stomal effluent vs fecal output) may introduce potential bias, as microbial communities naturally differ between the small intestine and colon. Future prospective studies incorporating preterm controls, clinically stable home-managed SBS patients, and systematic dietary records are warranted to further validate these findings.

In conclusion, children with SBS exhibit profound gut microbial dysbiosis, with each SBS type displaying distinct taxonomic and functional signatures across bacteria, fungi, archaea, and viruses. Beyond compositional and pathway-level alterations, type-specific associations between differential KO genes and clinical indicators highlight the functional relevance of the microbiome in shaping disease manifestations. Our findings provide a framework for future precision medicine, emphasizing the potential for targeted modulation of the gut microbiota tailored to specific pediatric SBS types.

## Data Availability

The data sets generated and analyzed in the course of this research are accessible upon formal request to the corresponding author. Raw sequencing data from metagenomic sequencing were registered at NCBI under BioProject PRJNA1348713.

## SUPPLEMENTAL MATERIAL

Supplemental figures msystems.00316-26-s0001.docx Fig. S1 to S3.

Supplemental Tables msystems.00316-26-s0002.pdf Tables S1 to S9.

ASM does not own the copyrights to Supplemental Material that may be linked to, or accessed through, an article. The authors have granted ASM a non-exclusive, world-wide license to publish the Supplemental Material files. Please contact the corresponding author directly for reuse.
