# Supplementary material for: Multi-kingdom gut microbiota analyses identify biomarkers of different types of pediatric short bowel syndrome
Source: mSystems. 2026 Sep 1;11(9):e00316-26. doi: 10.1128/msystems.00316-26 (PMC13595922; doi:10.1128/msystems.00316-26)
Supplement: Supplementary file 1 [file msystems.00316-26-s0002.pdf]

Supplementary Table 1. Statistical significance of demographic and clinical characteristics of patients

**Variable**	***P* value**	
Age	SBS I vs. SBS II	**0.003**
	SBS I vs. SBS III	**0.023**
	SBS I vs. Control	**0.001**
	SBS II vs. SBS III	**0.032**
	SBS III vs. Control	**0.039**
PN_duration	SBS I vs. SBS II	**0.013**
Antibiotics	SBS I vs. Control	**<0.001**
	SBS II vs. Control	**<0.001**
	SBS III vs. Control	**<0.001**
probiotics	SBS I vs. Control	**0.05**
	SBS II vs. Control	**0.043**
	SBS III vs. Control	**0.001**
PPI	SBS I vs. Control	**<0.001**
	SBS II vs. Control	**<0.001**
	SBS III vs. Control	**<0.001**

Note: Wilcoxon test. SBS I: short bowel syndrome type 1; SBS II: short bowel syndrome type 2; SBS III: short bowel syndrome type 3; Control: healthy controls; PPI: proton pump inhibitor.

Supplementary Table 2. List of differential microbial species between controls and SBS types identified by ANCOM-BC2, related to Fig. 4a.

SBS I vs. Control	SBS II vs. Control	SBS III vs. Control
Intestinibacter bartlettii	Blautia wexlerae	Streptococcus pasteurianus
Faecalibacillus intestinalis	Eggerthella lenta	[Clostridium] innocuum
Anaerostipes hadrus	Anaerostipes hadrus	Faecalibacterium sp. I2-3-92
Mediterraneibacter gnavus	Blautia luti	Akkermansia muciniphila
Eubacterium ventriosum	Roseburia intestinalis	Intestinibacter bartlettii
Clostridium sp. BJN0001	Faecalibacterium sp. BCRC 81399	Clostridium sp. DL-VIII
Faecalibacterium sp. IP-1-18	Wujia chipingensis	Clostridium gelidum
Paludicola sp. MB14-C6	Agathobacter rectalis	Thomasclavelia ramosa
Clostridium chauvoei	Hungatella hathewayi	Faecalibacterium sp. IP-1-18
Collinsella aerofaciens	Mediterraneibacter gnavus	Bacteroides sp. DH3716P
Crassaminicella thermophila	Blautia hansenii	Bacteroides caecimuris
Tannockella kyphosi	Faecalibacillus intestinalis	Bacteroides faecis
Brevibacillus sp. WF146	Dorea formicigenerans	Bacteroides fragilis
Bacillus pumilus	Coprococcus catus	Parabacteroides johnsonii
Paenibacillus graminis	Faecalibacterium sp. I4-3-84	Bacteroides zoogleoformans
Thermoanaerobacterium sp. CMT5567-10	Pseudomonas mandelii	Romboutsia ilealis
Carboxydothermus hydrogenoformans	Serpentinicella alkaliphila	Clostridium bornimense
uncultured Desulfobulbus sp.	Romboutsia sp. 13368	Clostridium chauvoei
Paenibacillus sp. FSL R10-2734	Intestinibacter bartlettii	Anaerostipes hadrus

Serpentinicella alkaliphila	Collinsella aerofaciens	Collinsella aerofaciens
Paenibacillus sp. URB8-2	Blautia sp. SC05B48	Intestinibacillus sp. NTUH-41-i26
Luteolibacter sp. SL250	Dorea longicatena	Bacteroides caccae
Rosistilla carotiformis	[Clostridium] scindens	Clostridium butyricum
Laceyella sacchari	[Ruminococcus] torques	Prevotella denticola
Olsenella sp. oral taxon 807	Anaerobutyricum hallii	Pyramidobacter piscolens
Streptomyces aurantiacus	Eubacterium ventriosum	Parolsenella catena
Magnetococcus marinus	Romboutsia sp. CE17	Parabacteroides chongii
Nitrospina watsonii	Thomasclavelia ramosa	Faecalibacillus intestinalis
Thermococcus sp. MAR1	Coprococcus comes	Ruminococcus bovis
Mycobacterium vicinigordonae	[Clostridium] innocuum	Clostridium sp. C1
Prevotella multiformis	Terrisporobacter mayombei	Butyricimonas faecihominis
Halopseudomonas sp. SMJS2	Faecalibacterium sp. HTF-F	Bacteroides luhongzhouii
Pectobacterium wasabiae	Faecalibacterium prausnitzii	Bacteroides nordii
Lachnospira eligens	Eggerthella guodeyinii	Terrisporobacter mayombei
Faecalibacterium sp. IP-3-29	Allocoprobacillus halotolerans	Blautia wexlerae
Roseburia rectibacter	Faecalibacterium duncaniae	Prevotella multiformis
[Ruminococcus] torques	Faecalibacterium sp. I3-3-89	Gordonibacter pamelaeae
Blautia pseudococcoides	Ruminococcus bicirculans (ex Wegman et al. 2014)	Prevotella herbatica
Vescimonas coprocola	Lachnospira eligens	Bacillus atrophaeus
Blautia hansenii	Faecalibacterium sp. I3-3-33	Serpentinicella alkaliphila
Blautia producta	Roseburia sp. 499	Dialister massiliensis
Faecalibacterium duncaniae	Marvinbryantia formatexigens	Treponema putidum
Marvinbryantia formatexigens	Brachybacterium kimchii	Romboutsia sp. 13368
Faecalibacterium sp. I4-3-84	Blautia obeum	Peptoclostridium acidaminophilum
Anaerobutyricum hallii	Terrisporobacter petrolearius	Coprobacter fastidiosus
Roseburia intestinalis	Sellimonas intestinalis	Mediterraneibacter gnavus
Kineothrix sp. MB12-C1	Romboutsia ilealis	Caminicella sporogenes
Anaerostipes rhamnosivorans	Eubacterium sp. MSJ-33	Bacteroides cellulosilyticus
Blautia coccoides	Blautia producta	Terrisporobacter glycolicus
Lacrimispora xylanolytica	Bacillus paralicheniformis	Bacteroides sp. HF-162
Qiania dongpingensis	Blautia argi	Thermoanaerobacterium sp. RBIITD
Faecalibacterium sp. BCRC 81399	Ruthenibacterium lactatiformans	Tannockella kyphosi
Simiaoa sunii	Macrococcus armenti	Oceanirhabdus sp. W0125-5
Clostridium baratii	Anaerostipes rhamnosivorans	Streptococcus infantarius
Roseburia hominis	Simiaoa sunii	Clostridium felsineum
Agathobacter rectalis	Clostridium gasigenes	Bacteroides heparinolyticus
Eggerthella lenta	Monoglobus pectinilyticus	Streptococcus alactolyticus
Ruminococcus bicirculans (ex Wegman et al. 2014)	Anaerocolumna cellulosilytica	Clostridium pasteurianum
Roseburia sp. 499	Butyrivibrio crossotus	Bacteroides humanifaecis
Faecalibacterium sp. I3-3-33	Clostridium cadaveris	Aggregatibacter aphrophilus

Enterocloster bolteae	Clostridium sp. MD294	[Clostridium] colinum
Faecalibacterium prausnitzii	[Ruminococcus] lactaris	Chryseobacterium arthrosphaerae
Coprococcus catus	Anaerocolumna sp. AGMB13025	Caldanaerobacter subterraneus
Faecalibacterium sp. I3-3-89	Roseburia sp. 831b	Coriobacterium glomerans
Blautia sp. SC05B48	Bacillus atrophaeus	Weissella confusa
Ruminococcus sp. SR1/5	Ruminococcus gauvreauii	Clostridium scatologenes
Blautia obeum	Bacillus pseudomycoides	Neobacillus sp. Marseille-Q6967
Acutalibacter muris	Clostridium sp. LQ25	Turicibacter sp. H121
Blautia argi	Butyrivibrio hungatei	Clostridium manihotivorum
Clostridium sp. M62/1	Clostridium taeniosporum	Butyricimonas virosa
Pseudobutyrivibrio xylanivorans	Clostridium gelidum	Streptobacillus moniliformis
Faecalibacterium sp. HTF-F	Faecalibacterium sp. IP-1-18	Bacteroides sp. ZJ-18
Blautia liquoris	Butyrivibrio proteoclasticus	Cellulosilyticum sp. WCF-2
Megamonas hypermegale	Streptococcus pasteurianus	Sutterella megalosphaeroides
Intestinimonas butyriciproducens	Tepidibacter hydrothermalis	Brevibacillus composti
Blautia hydrogenotrophica	Clostridium sp. BJN0001	Leptotrichia wadei
Dorea longicatena	Coprococcus eutactus	Denitrobacterium detoxificans
Coprococcus comes	Clostridium isatidis	Clostridium botulinum
Ruminococcus champanellensis	Clostridium baratii	Solibacillus sp. FSL W8-0372
Hungatella hathewayi	Anaerocolumna chitinilytica	Paenibacillus graminis
Enterocloster asparagiformis	Candidatus Arthromitus sp. SFB-rat-Yit	Muribaculum gordoncarteri
Bifidobacterium animalis	Kineothrix sp. MB12-C1	Fusobacterium varium
Faecalibacterium sp. I4-1-79	Solibacillus sp. FSL W8-0372	Bacteroides sp. MSB163
Lachnoclostridium phocaeense	Wansuia hejianensis	Paenibacillus woosongensis
Lachnoanaerobaculum gingivalis	Paenibacillus sp. FSL R5-0912	Planococcus sp. N022
Bacteroides sp. D2	Bacillus sp. Y1	Mammaliicoccus sciuri
Clostridioides difficile	Crassaminicella indica	Parolsenella massiliensis
Eggerthella guodeyinii	Oscillibacter sp. PEA192	Methanobrevibacter sp.
Eubacterium sp. MSJ-33	Lacrimispora xylanolytica	Segatella bryantii
Coprococcus sp. ART55/1	Bacillus swezeyi	Eubacterium limosum
Fusobacterium pseudoperiodonticum	Keratinibaculum paraultunense	Gallibacterium anatis
Anaerocolumna sp. MB42-C2	Clostridium bornimense	Macrococcoides caseolyticum
Clostridium tyrobutyricum	Anaerotignum sp. MB30-C6	Proteiniborus sp. MB09-C3
Chordicoccus furentiruminis	Lachnoclostridium phytofermentans	Dehalobacter sp. CF
Blautia luti	Turicibacter bilis	Chryseobacterium camelliae
[Clostridium] scindens	Lactococcus formosensis	Hathewaya histolytica
Clostridium perfringens	Paenibacillus graminis	Vescimonas coprocola
Clostridium saccharoperbutylacetonicum	Jeotgalibaca arthritidis	Eubacterium ventriosum
Bacteroides ovatus	Enterocloster clostridioformis	Crassaminicella thermophila
Dysosmobacter welbionis	Planococcus sp. N022	uncultured Bacteroides sp.
Intestinibacillus sp. NTUH-41-i26	Methanobrevibacter ruminantium	Enterococcus gilvus

Selenomonas sputigena	Streptococcus lutetiensis	Bacillus pumilus
Oscillibacter sp. PEA192	Ruminococcus bovis	Radiobacillus kanasensis
Clostridium butyricum	Clostridium septicum	Paludicola sp. MB14-C6
Flintibacter sp. KGMB00164	Radiobacillus kanasensis	Halanaerobium hydrogeniformans
[Clostridium] hylemonae	Halanaerobium praevalens	Streptococcus thermophilus
Solobacterium moorei	Clostridium argentinense	Petrimonas mucosa
Eubacterium maltosivorans	Oceanirhabdus sp. W0125-5	Fusobacterium vincentii
Veillonella sp. S12025-13	Blautia parvula	Priestia flexa
Clostridium beijerinckii	Anaeromicropila herbilytica	Liquorilactobacillus hordei
uncultured Trichococcus sp.	Fannyhessea vaginae	Clostridium tagluense
Blautia massiliensis (ex Durand et al. 2017)	Caldicellulosiruptor morganii	Priestia filamentosa
Clostridium septicum	Clostridium acetobutylicum	Oxalobacter formigenes
Peptostreptococcus equinus	Anaerostipes caccae	Pseudodesulfovibrio aespoeensis
uncultured Enterococcus sp.	Leptotrichia hongkongensis	Alistipes senegalensis
Paenibacillus urinalis	Thermoanaerobacterium sp. CMT5567-10	Polynucleobacter necessarius
Veillonella nakazawae	Clostridium chauvoei	Emergencia sp. JLR.KK010
Leptotrichia sp. oral taxon 221	Clostridium sp. BNL1100	Heyndrickxia vini
Lacrimispora saccharolytica	Bacillus sp. NEB1478	Ruminococcus champanellensis
Leptotrichia wadei	Gracilibacillus salitolerans	Coprobacter secundus
Lachnoanaerobaculum umeaense	Komagataeibacter oboediens	Prevotella sp. E15-22
Flavonifractor plautii	Lysinibacillus sp. SGAir0095	Sedimentibacter sp. zth1
Peribacillus frigoritolerans	Clostridium diolis	Muribaculum intestinale
Bifidobacterium adolescentis	Anaerocolumna sedimenticola	Enterococcus sp. DIV2402
Olsenella timonensis	Blautia coccoides	Desulfocurvibacter africanus
Veillonella rogosae	Priestia koreensis	Olsenella uli
Leptotrichia sp. oral taxon 218	Candidatus Minimicrobia sp. QA0096	Burkholderia pseudomallei
Paenibacillus mucilaginosus	Paenalkalicoccus suaedae	Terrisporobacter petrolearius
Collinsella stercoris	Staphylococcus gallinarum	Acidilutibacter cellobiosedens
Selenomonas sp. oral taxon 920	Herbinix luporum	Thermoanaerobacterium sp. CMT5567-10
Campylobacter jejuni	Corynebacterium endometrii	Paraliobacillus zengyii
Anaerostipes caccae	Metabacillus sp. FJAT-52054	Solibacillus daqui
Desulfovibrio desulfuricans	Paraclostridium bifermentans	Clostridium sp. OS1-26
Fusobacterium nucleatum	Clostridium sp. OS1-26	Enterobacter chuandaensis
Sporomusa ovata	Anaerocolumna sp. MB42-C2	Gracilibacillus salitolerans
Anaerotruncus colihominis	Fusobacterium varium	Methanobrevibacter woesei
Clostridium isatidis	Bacillus sp. 1NLA3E	Niallia sp. RD1
Parafannyhessea umbonata	Alkalihalophilus pseudofirmus	Bacillus sp. S3
Vescimonas fastidiosa	Corynebacterium lizhenjunii	Natranaerobius thermophilus
Catenibacterium sp. co_0103	Paenibacillus sp. HWE-109	Mobiluncus sp. Marseille-Q7826
Pusillibacter faecalis	Halobacillus shinanisalinarum	Lactococcus cremoris
Atopobium sp. oral taxon 416	Bacteroides sp. A1C1	Clostridium fermenticellae

Paenibacillus macerans	Clostridium tyrobutyricum	Brachyspira intermedia
Trichlorobacter lovleyi	Streptococcus sp. 29892	Pullulanibacillus sp. KACC 23026
Enterococcus gilvus	Crassaminicella thermophila	Moorella humiferrea
Helicobacter pylori	Petrocella atlantisensis	Clostridium tetani
Neobacillus sp. Marseille-Q6967	Staphylococcus ratti	Arthrobacter alpinus
Clostridium saccharobutylicum	Cytobacillus kochii	Clostridium gasigenes
Arabiibacter massiliensis	Helicovermis profundi	Brevibacillus brevis
Bacteroides finegoldii	Paenibacillus lentus	Sodaliphilus pleomorphus
Peptacetobacter hiranonis	Leminorella richardii	Monoglobus pectinilyticus
Paenibacillus sp. N3/727	Paludicola sp. MB14-C6	Leptotrichia sp. HMT-225
Cloacibacillus porcorum	Desemzia incerta	Chryseobacterium sp. MA9
Gordonibacter pamelaeae	Clostridium sp. JN-1	Rahnella aceris
Emergencia sp. JLR.KK010	Acetivibrio saccincola	Ilyonectria robusta
Sphingobacterium multivorum	Chryseobacterium gleum	Metabacillus sp. FJAT-52054
Chryseobacterium gleum	Sulfurimonas sp.	Botrytis sinoallii
Draconibacterium halophilum	Francisella philomiragia	Candidatus Erwinia haradaeae
Paenibacillus lycopersici	Paenibacillus sp. FSL K6-3182	Clostridium sp. CM028
Niallia circulans	Echinicola marina	Providencia rettgeri
Romboutsia hominis	Vallitalea guaymasensis	Segatella oris
Paenibacillus xylanilyticus	Candidatus Arthromitus sp. SFB-mouse	Aminipila luticellarii
Selenomonas sp. TAMA-11512	Tenacibaculum ovolyticum	Bombilactobacillus bombi
Paenibacillus dendritiformis	Lactococcus cremoris	Streptococcus urinalis
Brachyspira hyodysenteriae	Hypoxylon fragiforme	Prevotella dentalis
Candidatus Minimicrobia vallesae	Anaerocolumna sp. AGMB13020	Delftia acidovorans
Clostridium botulinum	uncultured Sphaerochaeta sp.	Clostridium cochlearium
Paenibacillus sp. H1-7	Gloeobacter violaceus	Rummeliibacillus stabekisii
Paenibacillus cellulositrophicus	Halobacillus halophilus	Neobacillus novalis
Emergencia timonensis	Massilistercora timonensis	Clostridium sp. BJN0001
Anoxybacter fermentans	Listeria ivanovii	Corynebacterium durum
Neobacillus sp. PS2-9	Clostridium sp. JS66	Haemophilus pittmaniae
Enterocloster clostridioformis	Bacillus safensis	Candida jiufengensis
Sporosarcina ureae	Penicillium robsamsonii	Vagococcus intermedius
Lachnoclostridium phytofermentans	Halarcobacter sp.	Clostridium acetobutylicum
Gottfriedia acidiceleris	Erysipelothrix sp. HDW6C	Pestalotiopsis fici
Paenibacillus silvisoli	Fictibacillus arsenicus	Leadbettera azotonutricia
Paenibacillus sp. MBLB1832	Caloranaerobacter azorensis	Streptomyces tanashiensis
Candidatus Minimicrobia sp. QA0096	Paenibacillus woosongensis	Bacillus swezeyi
Aminipila luticellarii	Streptomyces sp. NBC_00376	Bacteroides graminisolvens
uncultured Methanobrevibacter sp.	Treponema peruense	Methanobrevibacter arboriphilus
Tepidibacter hydrothermalis	Bibersteinia trehalosi	Echinicola marina
Bifidobacterium catenulatum	Intestinimonas butyriciproducens	Paenibacillus sp. FSL H8-0457

Pectobacterium aroidearum	Sporosarcina sp. Te-1	Staphylococcus cohnii
Chondrinema litorale	Clostridium sp. MB40-C1	Abyssicoccus albus
Duodenibacillus massiliensis	Paenibacillus terrae	Desulfoluna limicola
Paenibacillus elgii	Ruminiclostridium cellulolyticum	Caldicellulosiruptor diazotrophicus
Allobaculum mucilyticum	Proteiniborus sp. MB09-C3	Geobacillus thermodenitrificans
Yersinia frederiksenii	Halobacillus mangrovi	Blautia sp. KLE_1732_HM_1032
Bacillus mycoides	Fastidiosipila sanguinis	Vibrio natriegens
Coprobacter fastidiosus	Carnobacterium viridans	Bacillus shivajii
Loigolactobacillus coryniformis	Fusarium venenatum	Komagataeibacter oboediens
Desulfoscipio gibsoniae	Lysinibacillus timonensis	Sporosarcina sp. PTS2304
Flavobacterium ginsengisoli	Alkalicoccus halolimnae	Berryella wangjianweii
Thermophilibacter immobilis	Glutamicibacter arilaitensis	Paenibacillus kribbensis
Empedobacter brevis	Clostridium scatologenes	Carnobacterium viridans
Methanobrevibacter sp.	Luteolibacter sp. LG18	Floricoccus penangensis
Aneurinibacillus uraniidurans	Bacillus amyloliquefaciens	Cytobacillus dafuensis
Mogibacterium pumilum	Rubrobacter radiotolerans	Paenibacillus kyungheensis
Hyaloscypha bicolor	Acetivibrio clariflavus	Bacillus cereus
Clostridium thermarum	Novisyntrophococcus fermenticellae	Xiashengella succiniciproducens
Proteus mirabilis	Fusobacterium ulcerans	Paenibacillus odorifer
Streptobacillus moniliformis	Schnuerera ultunensis	Desemzia incerta
Gemella sanguinis	Neisseria canis	Helcococcus kunzii
Paenibacillus tritici	Tissierella sp. MB52-C2	Campylobacter concisus
Chryseobacterium camelliae	Talaromyces stipitatus	Metabacillus sediminilitoris
Enterococcus wangshanyuanii	Qiania dongpingensis	Anaerococcus prevotii
uncultured Desulfuromonas sp.	Halomonas sp. 7T	Olsenella sp. oral taxon 807
Planococcus sp. MB-3u-03	Streptomyces sp. NBC_01352	Fictibacillus enclensis
Parolsenella catena	Ruminiclostridium papyrosolvens	Halothece sp. PCC 7418
Desulfobulbus propionicus	Lysinibacillus sp. 2017	Fusobacterium mortiferum
Desulfocurvibacter africanus	Alternaria burnsii	Prevotella veroralis
Gudongella oleilytica	Fenollaria massiliensis	Tepidibacillus sp. LSZ-M11000
Pelobacter propionicus	Solibacillus sp. R5-41	Intestinibaculum porci
Pedobacter riviphilus	Pseudodesulfovibrio sp. 5S69	Campylobacter sp. RM6914
Treponema brennaborense	Paraliobacillus zengyii	Gelria sp. Kuro-4
Planococcus sp. N016	Pontibacillus sp. HMF3514	Bacillus sp. OxB-1
Peptoniphilus sp. SAHP1	Pseudoleptotrichia goodfellowii	Desulfurivibrio alkaliphilus
Enterococcus rotai	Winogradskyella sediminis	Laribacter hongkongensis
Mammaliicoccus vitulinus	Dictyoglomus thermophilum	Pontibacter sp. G13
Paenibacillus sp. JNUCC-31	Terrisporobacter glycolicus	Arthrobacter sp. U41
Mesobacillus sp. AQ2	Blautia liquoris	Prevotella sp. oral taxon 299
uncultured Flavobacterium sp.	[Clostridium] colinum	Geovibrio thiophilus
Methanococcus voltae	Paenibacillus sp. FSL H8-0537	Rossellomorea marisflavi

Enterococcus sp. DIV2402	Fulvia fulva	Gallibacterium salpingitidis
Thermoanaerobacterium thermosaccharolyticum	Berryella intestinalis	Hyaloscypha bicolor
Peribacillus sp. R9-11	Oligella ureolytica	Sedimentibacter sp. MB35-C1
Clostridium sp. JN-9	Pelodictyon luteolum	Globicatella sp. PHS-GS-PNBC-21-1553
Riemerella anatipestifer	Sulfitobacter sp. S223	Clostridium thermarum
Sphingobacterium spiritivorum	Ehrlichia ruminantium	Arcobacter sp. L
Sporomusa termitida	Burkholderia sp. THE68	Brevibacillus choshinensis
Methylococcus capsulatus	Oxalobacter formigenes	Aneurinibacillus sp. Ricciae_BoGa-3
Tepiditoga spiralis	Marinomonas algicola	Kwoniella pini
Aequorivita sp. Ant34-E75	Pseudodesulfovibrio profundus	Pantoea soli
Pseudodesulfovibrio indicus	Candida jiufengensis	Flavobacterium aestivum
Flavobacterium ginsenosidimutans	Deinococcus proteolyticus	Limnobacter sp. SAORIC-580
Treponema socranskii	Clostridium cochlearium	Sebaldella termitidis
Enterococcus sp. DIV1094	Paenibacillus sp. URB8-2	Fenollaria massiliensis
Babjeviella inositovora	Haloarcula litorea	Propioniciclava sp. MC1595
Spirosoma endbachense	Leptotrichia sp. HMT-225	Geotalea daltonii
Desulfosarcina ovata	Actinomyces sp. zg-332	Lysinibacillus sp. 2017
Lacrimispora sphenoides	Chordicoccus furentiruminis	Staphylococcus debuckii
Bacillus methanolicus	Desulfosarcina sp. BuS5	Gimesia aquarii
Wickerhamomyces ciferrii	Rosistilla carotiformis	uncultured Enterococcus sp.
Paenibacillus sp. RC67	Yersinia intermedia	Arcobacter nitrofigilis
Halorubrum ezzemoulense	Paraburkholderia kirstenboschensis	Treponema saccharophilum
Candidatus Sulfidibacterium hydrothermale	Endocarpon pusillum	Macrococcoides canis
Pseudomonas sp. CCOS 191	Candidatus Endolissoclinum faulkneri	Vibrio furnissii
Lysinibacillus sp. SGAir0095	Lysinibacillus irui	Haematobacter massiliensis
Chitinophaga oryzae	Niallia oryzisoli	Vibrio mangrovi
Bacillus sp. OxB-1	Chryseobacterium sp. MEBOG06	Pseudomonas sp. gcc21
Archangium violaceum	Yersinia mollaretii	Ignavibacterium sp.
Parvimonas micra	Erysipelothrix piscisicarius	Fervidibacillus halotolerans
Edwardsiella piscicida	Lysinibacillus parviboronicapiens	Pediococcus inopinatus
Paenibacillus sp. FSL W8-0194	Acaryochloris marina	Pedobacter mucosus
Planococcus sp. N056	Leptodesmis sichuanensis	Cardiobacterium sp. Marseille-Q4385
Neobacillus sp. OS1-2	Parageobacillus caldoxylosilyticus	Clostridium estertheticum
Cohnella candidum	Methanobrevibacter thaueri	Hymenobacter aerilatus
Methylosinus sp. C49	Sporosarcina sp. T2O-4	Pseudoalteromonas rubra
Trichoderma atroviride	Legionella micdadei	Aliivibrio fischeri
Azorhizobium caulinodans	Peribacillus butanolivorans	Desulfoferula mesophilus
Lautropia mirabilis	Streptococcus didelphis	Dickeya solani
Imperialibacter roseus	uncultured Methanosphaera sp.	Marispirochaeta aestuarii
Penicillium soppii	Wallemia ichthyophaga	Colletotrichum chrysophilum
Gottschalkia acidurici	Isoalcanivorax pacificus	Oceanotoga sp. DSM 15011

Vaginimicrobium propionicum	Staphylococcus chromogenes	Jeotgalicoccus sp. ATCC 8456
Mogibacterium neglectum	Tetrapisispora blattae	[Candida] subhashii
Ideonella dechloratans	Oenococcus oeni	Alicyclobacillus mengziensis
Campylobacter coli	Nostoc sp. PCC 7120 = FACHB-418	Penicillium subrubescens
Chromobacterium vaccinii	Streptomyces thermocarboxydus	Terribacillus sp. DMT04
Sphingobacterium sp. DR205	Clostridium sp. SY8519	Pseudalkalibacillus hwajinpoensis
Kovacikia minuta	Candidatus Epulopiscium viviparus	Clostridium sp. MF28
Vanderwaltozyma polyspora	Nodularia spumigena	Microbulbifer sp. MI-G
Paraclostridium sordellii	Brevibacterium sandarakinum	Tetragenococcus halophilus
Tichowtungia aerotolerans	Alkalihalobacillus sp. LMS6	Acinetobacter schindleri
Zymomonas mobilis	Haploplasma axanthum	Roseovarius sp. W115
Parazoarcus communis	Actinobacillus delphinicola	Melittangium boletus
Clostridium estertheticum	Thermochromatium tepidum	Vagococcus penaei
Paraburkholderia tropica	Sulfitobacter mediterraneus	Staphylococcus carnosus
Zhouia spongiae	Priestia filamentosa	Pseudodesulfovibrio sp. zrk46
Salipaludibacillus sp. LMS25	Evansella cellulosilytica	Ligilactobacillus animalis
uncultured Desulfobacter sp.	Brachyspira pilosicoli	Lentinula edodes
Chitinophaga agri	Coprococcus sp. ART55/1	Nocardiopsis dassonvillei
Clostridium sp. 001	Leptotrichia trevisanii	Lacticaseibacillus pantheris
Planococcus versutus	Brevibacillus sp. WF146	Methanospirillum sp.
Brevibacillus ruminantium	Saccharomyces kudriavzevii	Prevotella sp. E2-28
Cohnella cholangitidis	Trichoderma breve	Berryella intestinalis
Deinococcus wulumuqiensis	Pseudomonas atacamensis	Sulfuricaulis limicola
Blattabacterium cuenoti	Phycicoccus endophyticus	Methylobacter sp. YRD-M1
Piscinibacter sp. XHJ-5	Trichothermofontia sichuanensis	Acinetobacter baumannii
Fusobacterium hwasookii	Kingella oralis	Mycoplasmopsis felis
Photobacterium leiognathi	Peptacetobacter hiranonis	Ornithinibacillus sp. FSL M8-0202
Treponema vincentii	Corynebacterium sp. P3-F1	Chryseobacterium balustinum
Candidatus Contubernalis alkalaceticus	Undibacterium parvum	Desulfotalea psychrophila
Chitinimonas koreensis	Cyanobacterium sp. Dongsha4	Staphylococcus devriesei
Streptomyces sp. SCA4-21	Cellulophaga sp. L1A9	Agrobacterium leguminum
Mariniflexile sp. TRM1-10	Staphylococcus agnetis	Bacillus sp. 1NLA3E
Paenibacillus sp. YPG26	Vibrio spartinae	Paenibacillus sp. URB8-2
Flavobacterium sp. KBS0721	Phototrophicus methaneseepsis	Thermobacillus composti
uncultured Desulfosarcina sp.	Trametes versicolor	Desulforamulus reducens
Rhodopseudomonas sp. BAL398	Tatumella ptyseos	Streptomyces fradiae
Actinomyces sp. oral taxon 169	Streptomyces sp. M92	Campylobacter pinnipediorum
Lutibacter sp. A64	Flavobacterium cerinum	Desulfobulbus oralis
Agrobacterium tumefaciens	Roseovarius faecimaris	Amycolatopsis sp. FBCC-B4732
Rubrobacter radiotolerans	Aspergillus puulaauensis	Deinococcus wulumuqiensis
Vibrio rhizosphaerae	Anaerobacillus isosaccharinicus	Lancefieldella sp. Marseille-Q7238

Wenyingzhuangia fucanilytica	Chryseobacterium sp. G0186	Campylobacter sputorum
Brevundimonas diminuta	Streptomyces sp. NBC_00448	Leptogranulimonas caecicola
Chromobacterium violaceum	Acidovorax sp. NCPPB 3576	Desulfofarcimen acetoxidans
Tumebacillus avium	Salaquimonas pukyongi	Pseudomonas extremaustralis
Kaustia mangrovi	Bacidia gigantensis	Methanosarcina sp.
Megasphaera elsdenii	Aspergillus caelatus	uncultured Methanolobus sp.
Vibrio vulnificus	Dermabacter vaginalis	Marinilactibacillus sp. 15R
Neobacillus sp. DY30	Peptoniphilus harei	Lysinibacillus pakistanensis
Flavobacterium sp. GSB-24	Paenibacillus durus	Blautia massiliensis (ex Durand et al. 2017)
Synechococcus elongatus	Pantoea sp. At-9b	Tissierella sp. Yu-01
Pediococcus inopinatus	Bernardetia litoralis	Wenyingzhuangia fucanilytica
Paraburkholderia aromaticivorans	Suillus discolor	Bacillus methanolicus
Olivibacter sp. LS-1	Mycoplasma mycoides	Salisediminibacterium selenitireducens
Ornithobacterium rhinotracheale	Massilia sp. W12	Pantoea ananatis
Eremothecium gossypii	Psychrobacter sp. DAB_AL43B	Enterococcus sp. DIV1094
Azospirillum brasilense	Mycolicibacillus koreensis	Candidatus Sulfidibacterium hydrothermale
Corynebacterium vitaeruminis	Arthrobacter oryzae	Solibaculum mannosilyticum
uncultured Anaeromusa sp.	Flavobacterium sp. N1719	Fundicoccus culcitae
Methylocystis rosea	Tepiditoga spiralis	Lentibacillus amyloliquefaciens
Flavobacterium johnsoniae	Microbulbifer sp. THAF38	Photobacterium damselae
Rhodocytophaga rosea	Deinococcus sp. AB2017081	Chryseobacterium paludis
Catellatospora sp. IY07-71	Nostocoides sp. HKS02	Alkaliphilus oremlandii
Erythrobacter litoralis	Paenibacillus peoriae	Flavobacterium covae
Rubrobacter xylanophilus	Clostridium drakei	Polycladomyces abyssicola
Vagococcus sp. B2T-5	Halobacillus naozhouensis	Diaphorobacter aerolatus
Ferribacterium limneticum	Kurthia zopfii	Leuconostoc carnosum
Methylomagnum ishizawai	Allochromatium vinosum	Paenibacillus albus
uncultured Sphaerochaeta sp.	Sporosarcina sp. B2O-1	Desulfonema limicola
Proteiniclasticum sp. QWL-01	Candidatus Mycosynbacter amalyticus	Saprochaete ingens
Facklamia hominis	Ignavibacterium sp.	Trichoderma reesei
Pseudomonas viridiflava	Bacillus weihaiensis	Echinicola rosea
Halomonas alkalicola	Micromonospora krabiensis	Cytobacillus kochii
Alkalihalophilus pseudofirmus	Pelodictyon phaeoclathratiforme	Petrocella atlantisensis
Candidatus Nanosynbacter lyticus	Macrococcoides caseolyticum	Bacillus nitratireducens
Abyssalbus ytuae	Lysinibacillus fusiformis	Microbulbifer sp. YPW1
Hyphomicrobium nitrativorans	Brenneria izadpanahii	Gilbertella persicaria
Methylobacterium organophilum	Nocardioides pantholopis	Pantoea dispersa
Flavobacterium azooxidireducens	Serratia sp. UGAL515B_01	Letharia columbiana
Haliangium ochraceum	Hahella chejuensis	Aminipila butyrica
Hirsutella rhossiliensis	Staphylococcus kloosii	Staphylococcus edaphicus
Paenibacillus sp. FSL H3-0469	Thermobacillus composti	Amycolatopsis sp. NBC_01480

Salinimonas sediminis	Marinobacter sp. NP-4(2019)	Amycolatopsis sp. HUAS 11-8
Candida tropicalis	Yersinia rohdei	Bacillus vallismortis
Haematobacter massiliensis	Coraliomargarita sp. J2-16	Niallia sp. XMNu-256
Parastagonospora nodorum	Rhizobium sp. NLR16a	Peribacillus simplex
Cytobacillus oceanisediminis	Collinsella sp. zg1085	Hydrogenophaga sp. SL48
Bdellovibrio sp. 22V	Paraclostridium sordellii	Jeotgalibaca arthritidis
Mesorhizobium sp. 8	Legionella sp. PC997	Xanthomonas campestris
Peterkaempfera bronchialis	Aspergillus sclerotioniger	Petrimonas sp.
Eikenella corrodens	Brevibacterium luteolum	Komagataeibacter nataicola
Gluconobacter thailandicus	Natronomonas sp.	Comamonas odontotermitis
uncultured Acetobacteroides sp.	Methanoplanus endosymbiosus	Enterococcus sp. 9E7_DIV0242
Syntrophotalea acetylenica	Bacillus siamensis	Streptomyces liliifuscus
Nitrogeniibacter mangrovi	Alteracholeplasma palmae	Suillus fuscotomentosus
Miniimonas sp. S16	Vibrio aestuarianus	Amygdalobacter nucleatus
Neoehrlichia mikurensis	Sulfurovum indicum	Brevibacillus sp. WF146
Paraburkholderia edwinii	Annulohypoxylon maeteangense	Phycomyces blakesleeanus
Liquorilactobacillus hordei	Candida margitis	Rhizophagus irregularis
Herbaspirillum frisingense	Desulfosporosinus youngiae	Adhaeribacter radiodurans
Limnochorda pilosa	Novacetimonas hansenii	Acetobacter pasteurianus
Laribacter hongkongensis	Ilyonectria robusta	Avibacterium paragallinarum
Vibrio anguillarum	Penicillium subrubescens	Caminibacter mediatlanticus
Pseudomonas knackmussii	Campylobacter lanienae	Neoarthrinium moseri
Pseudomonas orientalis	Vibrio astriarenae	Desulfosudis oleivorans
Phaeoacremonium minimum	Bipolaris victoriae	Serpula lacrymans
Paenibacillus naphthalenovorans	Thalassotalea atypica	Leisingera thetidis
Sphingobacterium sp. ML3W	Arthrobacter caoxuetaonis	Facklamia hominis
Asticcacaulis excentricus	Bacillus spizizenii	Mycobacterium conspicuum
Aureibaculum algae	Bacillus pumilus	Staphylococcus muscae
Geobacillus zalihae	Bacillus glycinifermentans	Methanococcoides methylutens
Dechloromonas sp. A34	Tamlana carrageenivorans	Thalassolituus sp. C2-1
Vibrio navarrensis	Pseudomonas mosselii	Salinispira pacifica
Sutcliffiella horikoshii	Polaribacter sp. Hel1_33_78	Staphylococcus pasteuri
Pseudodesulfovibrio sp. zrk46	Streptomyces sp. NA04227	Paenibacillus physcomitrellae
Arthroderma uncinatum	Macrococcus bovicus	Erwinia billingiae
Halopseudomonas salegens	Nonomuraea sp. NBC_01738	Achromobacter sp. HaoLi
Mytilinidion resinicola	Paraburkholderia sp. SJ_3	Sphingobacterium sp. DR205
Burkholderia contaminans	Pochonia chlamydosporia	Limnohabitans sp. MORI2
Labrys sp. KNU-23	Providencia heimbachae	Streptacidiphilus sp. P02-A3a
Agromyces aureus	Streptomyces sp. NBC_00178	Tellurirhabdus rosea
Pectobacterium polaris	Lysinibacillus pakistanensis	Providencia alcalifaciens
Paracoccus stylophorae	Pseudonocardia autotrophica	Caldicellulosiruptor naganoensis

Geomonas subterranea	Pseudarthrobacter oxydans	Sulfurospirillum diekertiae
Streptomyces sp. WZ-12	Talaromyces proteolyticus	Bacillus wiedmannii
Dyella thiooxydans	Halanaerobium hydrogeniformans	Brucella anthropi
Salinibacter ruber	Bacillus inaquosorum	Pollutimonas thiosulfatoxidans
Beauveria bassiana	Alicyclobacillus dauci	Caldicellulosiruptor hydrothermalis
Massilia endophytica	Dothidotthia symphoricarpi	Ustilago maydis
Nocardioides anomalus	Bacillus sp. THAF10	Streptomyces aurantiacus
Symbiopectobacterium purcellii	Ferrigenium kumadai	Marinobacterium sediminicola
Bacillus thuringiensis	Lysinibacillus louembei	Sulfurimicrobium lacus
Pseudomonas arsenicoxydans	Lysinibacillus agricola	Bacillus altitudinis
Prochlorococcus marinus	Aneurinibacillus sp. Ricciae_BoGa-3	Pseudomonas knackmussii
Catenulispora acidiphila	Luteolibacter sp. SL250	Chryseobacterium manosquense
Shewanella polaris	Heliorestis convoluta	Deinococcus aquaticus
Mycobacterium sp. ELW1	Solidesulfovibrio carbinoliphilus	Chloracidobacterium aggregatum
Sphingomonas changnyeongensis	Didymosphaeria variabile	Gardnerella leopoldii
Gimesia chilikensis	Bacillus tequilensis	Tunicatimonas pelagia
Sphingopyxis sp. 113P3	Streptomyces sp. NBC_00162	Polaribacter litorisediminis
Marinobacter sp. NP-4(2019)	Virgibacillus sp. SK37	Trichophyton verrucosum
Cryptococcus neoformans	Staphylococcus arlettae	Apilactobacillus kunkeei
Brucella intermedia	Trueperella pecoris	Fusarium solani
Agrobacterium larrymoorei	Streptomyces aurantiacus	Sulfitobacter sp. S223
Pelagerythrobacter marinus	Defluviitoga tunisiensis	Bacillus sp. Y1
Alicyclobacillus acidocaldarius	Pseudomonas resinovorans	Thermosipho ferrireducens
Liberibacter crescens	Brachybacterium faecium	Botrimarina mediterranea
Colletotrichum karsti	Paenibacillus sp. FSL R10-2734	Cellulomonas xiejunii
Natrinema salaciae	Flavobacterium nackdongense	Rothia aeria
Ruminiclostridium cellulolyticum	Corynebacterium riegelii	Leptotrichia trevisanii
Mucilaginibacter celer	Planktothrix agardhii	Pasteurella multocida
Streptomyces asoensis	Streptomyces sp. NBC_01224	Paenibacillus sp. IHBB 10380
Halarcobacter sp.	Nocardia sp. NBC_00403	Staphylococcus argenteus
Flavobacterium sp. IMCC34852	Magnetococcus marinus	Ruminiclostridium papyrosolvens
Sulfuricurvum kujiense	Pseudanabaena sp. ABRG5-3	Methanimicrococcus hacksteinii
Ralstonia insidiosa	Burkholderia pseudomultivorans	Cellulophaga lytica
uncultured Lichenicoccus sp.	Corynebacterium faecale	Bacillus sp. KH172YL63
Trichoderma citrinoviride	Paenibacillus sp. MBLB1832	Vibrio campbellii
Cupriavidus nantongensis	Paraburkholderia sp. PGU19	Acidithiobacillus ferrooxidans
Jeongeupia sp. USM3	Bacillus carboniphilus	Kroppenstedtia pulmonis
Alkalitalea saponilacus	Paracoccus saliphilus	Obesumbacterium proteus
Planctopirus limnophila	Burkholderia pseudomallei	Haploplasma axanthum
Hypoxylon fragiforme	Agrobacterium sp. RAC06	Microsporum canis
Cucurbitaria berberidis	Trichoderma atroviride	Flavobacterium fluviale

Maridesulfovibrio sp.	Postia placenta	Uncinocarpus reesii
Sporolactobacillus terrae	Thiorhodovibrio frisius	Peribacillus muralis
Candidatus Desulforudis audaxviator	Kocuria sp. KD4	Histophilus somni
Fusarium vanettenii	Cordyceps fumosorosea	Bombilactobacillus thymidiniphilus
Nodularia sphaerocarpa	Limnohabitans sp. MORI2	Stenotrophomonas rhizophila
Mucilaginibacter rubeus	Caballeronia sp. SL2Y3	Candidatus Desulfovibrio trichonymphae
Mesoterricola silvestris	Micromonospora coriariae	Sphingobium cloacae
Aquiflexum balticum	Streptacidiphilus sp. P02-A3a	Neobacillus drentensis
Gluconobacter oxydans	Mycolicibacterium pallens	Mucilaginibacter sp. SMC90
Streptomyces sp. NEAU-sy36	Vibrio plantisponsor	Streptomyces qinzhouensis
Caulobacter sp. FWC26	Geobacillus subterraneus	Mucilaginibacter mali
Duganella zoogloeoides	Saccharomonospora azurea	Terriglobus roseus
Labrenzia sp. VG12	Debaryomyces fabryi	Vagococcus xieshaowenii
Mahella australiensis	Aureispira sp. CCB-E	Geoalkalibacter halelectricus
Pedobacter ginsengisoli	Streptomyces sp. DG1A-41	Aestuariibaculum lutulentum
Flavobacterium sp. N2038	Tannockella kyphosi	Symbiobacterium thermophilum
Elizabethkingia ursingii	Halomonas sp. THAF5a	Thiorhodovibrio frisius
Paracoccus zhejiangensis	Chryseobacterium scophthalmum	Penicillium antarcticum
Sphingopyxis indica	Jonesia denitrificans	Pseudomonas mucidolens
Simplicispira suum	Paenibacillus sp. CAA11	Streptomyces sp. CB01881
Devosia beringensis	Fusobacterium gonidiaformans	uncultured Desulfuromusa sp.
Polaribacter tangerinus	Roseiflexus sp. RS-1	Desulfobaculum bizertense
Paenibacillus algicola	Actinomadura sp. WMMA1423	Paenibacillus sp. FSL R10-2734
Pseudomonas alcaligenes	Nocardioides marmorisolisilvae	Bacillus sp. THAF10
Flavobacterium psychrophilum	Methylomonas denitrificans	Paenibacillus lutimineralis
Mycolicibacterium rutilum	Janthinobacterium sp. 1_2014MBL_MicDiv	Megalodesulfovibrio gigas
Paroceanicella profunda	Sphingosinicella flava	Nocardia sp. CS682
Lancefieldella sp. Marseille-Q7238	Candidatus Nitrosotalea sinensis	Propionibacterium freudenreichii
Streptomyces rimosus	Mycobacterium vicinigordonae	Comamonas fluminis
Flavobacterium pallidum	Rhodoferax sp. PAMC 29310	Mucilaginibacter sabulilitoris
Buttiauxella agrestis	Acidothermus cellulolyticus	Methanococcus voltae
Pediococcus claussenii	Roseobacter ponti	Mollisia scopiformis
Bordetella genomosp. 9	Halopseudomonas sabulinigri	Trichoderma gamsii
Sulfitobacter alexandrii	Carboxydothermus hydrogenoformans	Streptomyces sp. f51
Cercospora kikuchii	Corynebacterium auris	Paenibacillus algicola
Candidatus Thiodictyon syntrophicum	Kangiella sediminilitoris	Syntrophotalea acetylenica
Kribbella sp. NBC_01245	Streptomyces sp. NBC_00562	Macrococcus epidermidis
Actinomadura luteofluorescens	Deinococcus sp. AJ005	Vibrio aerogenes
Gemella morbillorum	Nostoc sp. PCC 7107	Arthrobacter sp. zg-Y1171
Skermanella pratensis	Marinomonas profundi	Deinococcus sp. NW-56
Conexibacter woesei	uncultured Anaeromusa sp.	Diaphorobacter sp. C33

Amycolatopsis sp. FDAARGOS 1241	Halococcus sediminicola	Micromonospora aurantiaca
Pseudorhizobium flavum	Delftia sp. Cs1-4	Micromonospora sp. WMMD975
Endocarpon pusillum	Streptomyces sp. NBC_00247	Photobacterium sp. GJ3
Paenibacillus alvei	Mollisia scopiformis	Clostridium fungisolvens
Moraxella sp. FZFQ2102	Desulfitobacterium dichloroeliminans	Nodularia spumigena
Luteolibacter luteus	Desulfoglaeba alkanexedens	Isoalcanivorax pacificus
Sphingobium sp. RAC03	Halobacteriovorax sp. BALOs_7	Massilia endophytica
Adhaeribacter radiodurans	Ligilactobacillus faecis	Flavobacterium nitratireducens
Azospirillum sp. TSA2s	Alternaria rosae	Paenibacillus sp. FSL R7-0216
Anaeromyxobacter paludicola	uncultured Methanoregula sp.	Massilia litorea
Flavobacterium kingsejongi	Erythrobacter neustonensis	Chryseobacterium cucumeris
Emticicia oligotrophica	Snodgrassella communis	Aeromonas media
Ralstonia pseudosolanacearum	Teredinibacter turnerae	Vallitalea pronyensis
Kribbella flavida	Poriferisphaera corsica	Jeotgalibaca dankookensis
Chenggangzhangella methanolivorans	Legionella spiritensis	Bacillus paralicheniformis
Mesorhizobium sp. L-2-11	Corynebacterium anserum	Vibrio gazogenes
Halobacteroides halobius	Parasphingorhabdus cellanae	Ornithinimicrobium avium
Aspergillus tanneri	Paenibacillus sp. BIC5C1	Buttiauxella agrestis
Chryseobacterium sp. Y16C	Paenibacillus sabinae	Levilactobacillus brevis
Desulfonema limicola	Chlorobium phaeovibrioides	Photobacterium sp. CCB-ST2H9
Actinomyces sp. zg-332	Mycolicibacterium tokaiense	Vibrio vulnificus
Pseudoalteromonas arctica	Pseudoalteromonas luteoviolacea	Trinickia violacea
Thermomonospora amylolytica	Desulforamulus ferrireducens	Endozoicomonas euniceicola
Pseudoduganella sp. SL102	Selenomonas sp. TAMA-11512	Amycolatopsis orientalis
Ruegeria sp. SCSIO 43209	Selenomonas ruminantium	Nocardia gipuzkoensis
Methylobacter sp. YRD-M1	Azospira inquinata	Bordetella trematum
Sphingomonas sp. NBWT7	Fusarium mangiferae	Streptomyces asoensis
Chryseobacterium sp. ZHDP1	Colwellia psychrerythraea	Marinobacter sp. LQ44
Phenylobacterium parvum	Pseudomonas sp. Z8(2022)	Priestia aryabhattai
Micromonospora sp. NBC_00330	Bacterioplanes sanyensis	Aminomonas paucivorans
Pelagovum pacificum	Pseudomonas granadensis	Streptomyces violaceus
Vibrio mimicus	Alkalimarinus coralli	Thiosocius teredinicola
Thermaerobacter marianensis	Neptunomonas phycophila	Mangrovibacillus cuniculi
Qipengyuania flava	Nostoc sp. UHCC 0870	Natranaerofaba carboxydovora
Radiomyces spectabilis	Spirosoma rhododendri	Streptomyces microflavus
Aquisediminimonas profunda	Gemella sp. zg-570	Leptospira noguchii
Salaquimonas pukyongi	Aspergillus neoniger	Marinomonas fungiae
Janthinobacterium sp. J1-1	Nosocomiicoccus ampullae	Cryptobacterium curtum
Kitasatospora sp. YST-16	Lactobacillus intestinalis	Mesorhizobium sp. B2-8-5
Chryseobacterium sp. LJ668	Hymenobacter sp. DG25A	Wickerhamomyces anomalus
Neorhizobium petrolearium	Paenibacillus thiaminolyticus	Micromonospora ferruginea

Massilia violaceinigra	Thermococcus sp. MAR1	Acinetobacter sp. PK01
Kribbella sp. NBC_00482	Microbacterium plantarum	Virgibacillus sp. SK37
Croceicoccus sp. YJ47	Micromonospora rifamycinica	Fusobacterium gonidiaformans
Chryseobacterium indoltheticum	uncultured Desulfobulbus sp.	Selenomonas sp. oral taxon 478
Devosia sp. A16	Halopseudomonas sp. SMJS2	Tolypothrix sp. PCC 7910
Spirosoma oryzicola	Microbacterium sp. CGR1	Halopseudomonas pelagia
Cyclobacterium marinum	Geobacter metallireducens	Pollutimonas sp. M17
Devosia sp.	Candidatus Fonsibacter ubiquis	Paracoccidioides lutzii
Pseudanabaena sp. ABRG5-3	Candidatus Vondammii sp. HM_W22	Lutibacter sp. A64
Brevundimonas sp. MF30-B	Halorubrum sp. BV1	Shewanella piezotolerans
Beutenbergia cavernae	Methanolobus chelungpuianus	Endozoicomonas sp. 4G
Tricharina praecox	Trichoderma gamsii	Roseovarius sp. Pro17
Paraburkholderia phenoliruptrix	Streptomyces sp. WZ-12	Pseudohongiella spirulinae
Miltoncostaea oceani	Thermodesulfobium narugense	Flavobacterium sp. KACC 22763
Pseudothermotoga thermarum	Robiginitalea biformata	Cryobacterium sp. 10S3
Paraconexibacter antarcticus	Cryptococcus amylolentus	Myroides odoratimimus
Rouxiella chamberiensis	Hymenobacter tibetensis	Aspergillus alliaceus
Pseudoduganella lutea	Pandoraea vervacti	Aaosphaeria arxii
Thermosipho melanesiensis	Moellerella wisconsensis	Micromonospora terminaliae
Fuerstiella marisgermanici	Thioalkalivibrio sp. K90mix	Staphylococcus xylosus
Thermococcus sp. GR4	Paenibacillus sp. FSL H8-0079	Pseudomonas sp. HS-18
Lentzea guizhouensis	Spirosoma sp. KCTC 42546	Penicilliopsis zonata
Methylobacterium phyllosphaerae	Halobaculum sp. DT92	Curvibacter sp. AEP1-3
Pseudomonas oryzihabitans	Natronorubrum texcoconense	Candidatus Planktophila dulcis
Paracoccus pantotrophus	Enterococcus rotai	Burkholderia metallica
Stieleria neptunia	Laceyella sacchari	Paenibacillus sp. FSL W8-1187
Paenibacillus andongensis	Clavibacter phaseoli	Arthrobacter sp. ATA002
Sulfitobacter mediterraneus	Pectobacterium wasabiae	Marinobacter salinus
Paenibacillus tianjinensis	Paracrocinitomix mangrovi	Paenibacillus polygoni
Pseudomonas sp. DTU_2021_1001937_2_SI_NGA_ILE_001	Flavobacterium praedii	Thermaerobacter sp. PB12/4term
[Pseudomonas] carboxydohydrogena	Brachybacterium halotolerans	Mesobacillus subterraneus
Methylocystis bryophila	Neokomagataea tanensis	Vibrio owensii
Geothrix sp. 21YS21S-2	Idiomarina sp. PL1-037	Pontibacter actiniarum
Pseudomonas cavernae	Clostridium ljungdahlii	Thiorhodovibrio litoralis
Methylobacterium indicum	Arthrobacter sp. StoSoilB22	Halomonas sp. 18071143
Buchnera aphidicola	Echinicola strongylocentroti	Kingella oralis
Streptomyces sp. NBC_00490	Serratia fonticola	Pseudodesulfovibrio sp. 5S69
Diaphorobacter sp. HDW4B	Aspergillus lentulus	Brevundimonas mediterranea
Runella sp. SP2	Paenibacillus sp. FSL R5-0345	Paraburkholderia aromaticivorans
Polaribacter sp. KT25b	Owenweeksia hongkongensis	Streptomyces sp. NBC_01334
Tardiphaga robiniae	Alicyclobacillus sp. ALC3	Daldinia caldariorum

Micromonospora sp. NBC_01740	Neopusillimonas aromaticivorans	Caldicellulosiruptor morganii
Streptomyces sp. NBC_01304	Anseongella ginsenosidimutans	Anaeropeptidivorans aminofermentans
Sparassis crispa	Bermanella marisrubri	Cyanobacterium aponinum
Blastomonas sp.	Moorena producens	Natrialba sp. SSL1
Brucella pseudintermedia	Micromonospora sp. NBC_00821	Bacteriovorax stolpii
Mycotypha africana	Rhodococcus sp. USK13	Vibrio cortegadensis
Paracoccus jeotgali	Planococcus sp. 107-1	Aquimarina sp. ERC-38
Marivirga harenae	Shewanella baltica	Maribacter dokdonensis
Dechloromonas denitrificans	Chlorobium limicola	Methylocaldum marinum
Amycolatopsis roodepoortensis	Xanthomonas euroxanthea	Pseudomonas sp. KHPS1
Streptomyces sp. NBC_00390	Halomonas sp. DN3	Ureibacillus thermophilus
Saccharomycodes ludwigii	Neisseria wadsworthii	Siansivirga zeaxanthinifaciens
Rhizobium sp. BG4	Pontibacter pudoricolor	Marinobacter sp. M3C
Streptomyces flavofungini	Haloplanus sp. CK5-1	Leptospirillum ferrooxidans
Sphingomonas bisphenolicum	Thermococcus sp.	Marivirga tractuosa
Polaribacter reichenbachii	Pectinatus frisingensis	Geothrix oryzae
Qipengyuania spongiae	Cruoricaptor ignavus	Vibrio sinaloensis
Umbelopsis ramanniana	Natronomonas gomsonensis	Rhodococcus globerulus
Opitutus terrae	Renibacterium salmoninarum	Ensifer canadensis
Brevundimonas albigilva	Erysipelothrix enhydrae	Rossellomorea sp. DA94
Marinobacter nanhaiticus	Pseudomonas sp. 31-12	Rhodomicrobium lacus
Gymnodinialimonas ceratoperidinii	Yersinia similis	Paracoccus sp. MA
Pandoraea thiooxydans	Lactobacillus amylolyticus	Pantoea vagans
Opitutus sp. GAS368	Synechococcus sp. PCC 6312	Mammaliicoccus sp. Marseille-Q6498
Azospirillum thiophilum	Microbacterium sp. 1.5R	Hymenobacter sp. BRD67
Hydrogenibacillus sp. N12	Actinotignum schaalii	Rouxiella chamberiensis
Sphingobium cloacae	Fusarium keratoplasticum	Poseidonibacter parvus
Cladophialophora psammophila	Micromonospora sp. WMMD998	Geobacillus thermoleovorans
Novosphingopyxis iocasae	Streptomyces sp. NBC_00353	Beauveria bassiana
Pararhodospirillum photometricum	Psychrobacillus glaciei	Carnobacterium divergens
Azospirillum humicireducens	Paenibacillus sp. RUD330	Candidatus Korobacter versatilis
Rhizobium sp. AB2/73	Corynebacterium lactis	Kerstersia gyiorum
Leisingera sp. S132	Alternaria ethzedia	Gimesia benthica
Rhodanobacter sp. FDAARGOS 1247	Levilactobacillus zymae	Arcobacter ellisii
Niallia sp. Man26	Paenibacillus sp. FSL R7-0331	Serratia proteamaculans
Aaosphaeria arxii	Thermodesulfobacterium geofontis	Streptomyces calvus
Rhizobium lusitanum	Gleimia hominis	Mesorhizobium sp. AR07
Dickeya fangzhongdai	Thermosediminibacter oceani	Cellulomonas sp. NTE-D12
Streptomyces cynarae	Pseudomonas psychrophila	Roseiflexus castenholzii
Cardiobacterium hominis	Neisseria lisongii	Aspergillus puulaauensis
Luteimonas chenhongjianii	Fulvitalea axinellae	Desarmillaria tabescens

Bradyrhizobium sp. BWC-3-1	Staphylococcus equorum	Bacillus arachidis
Rahnella sikkimica	Pseudalkalibacillus hwajinpoensis	Paenibacillus swuensis
uncultured Cohaesibacter sp.	Acidiferrobacter thiooxydans	Oryzomicrobium terrae
Jiangella sp. DSM 45060	Aminobacter aminovorans	Arthrobacter sp. zg-Y20
Ectothiorhodospira sp. BSL-9	Desulfosediminicola flagellatus	Caproicibacter sp. BJN0012
Acidithiobacillus caldus	Aspergillus saccharolyticus	Exophiala spinifera
Thermus aquaticus	Mycobacterium paraterrae	Luteimonas sp. JM171
Acidisoma sp. PAMC 29798	Chryseobacterium gambrini	Pantoea alhagi
Metabacillus sediminilitoris	Marinagarivorans cellulosilyticus	Paenibacillus sp. FSL F4-0243
Erythrobacter sp.	Thermobifida halotolerans	Qipengyuania aurantiaca
Halomonas sp. 3H	Aeromonas simiae	Synechococcus sp. CBW1107
Cedecea lapagei	Methanogenium organophilum	Methanococcus vannielii
Dechloromonas sp. HYN0024	Proteiniphilum saccharofermentans	Rufibacter radiotolerans
Peteryoungia desertarenae	Capnocytophaga gingivalis	Pontibacter akesuensis
Penicillium waksmanii	Lactobacillus acidophilus	Polaribacter tangerinus
Trinickia caryophylli	Persicobacter psychrovividus	Rosistilla carotiformis
Streptomyces actuosus	Halorientalis sp. IM1011	Kocuria turfanensis
Rhodoferax ferrireducens	Pseudomonas tritici	Hydrogenimonas cancrithermarum
Rhizosphaericola mali	Enterococcus sp. DIV0849a	Shewanella sp. Arc9-LZ
Methylococcus geothermalis	Halobellus sp. DFY28	Ureibacillus thermosphaericus
Legionella cherrii	Olsenella sp. oral taxon 807	Oceanidesulfovibrio marinus
Pseudoxanthomonas sp. X-1	Enterobacter cloacae complex sp. ECL405	Hymenobacter tibetensis
Pyruvatibacter mobilis	Klebsiella sp. WP4-W18-ESBL-05	Botrytis byssoidea
Phialemonium atrogriseum	Gordonia hongkongensis	Psychromonas sp. CNPT3
Mesorhizobium sp. Pch-S	Kitasatospora sp. MMS16-BH015	Paenibacillus sp. FSL R5-0766
Brevundimonas sp. M20	Aspergillus pseudonomiae	Paenibacillus jilunlii
Mongoliitalea daihaiensis	Arcanobacterium wilhelmae	Vibrio navarrensis
Luteimonas fraxinea	Streptomyces hirsutus	Bartonella apihabitans
Pseudoduganella flava	Pseudanabaena sp. PCC 7367	Paracoccus denitrificans
Thermospira aquatica	Corynebacterium diphtheriae	Flavobacterium sp. N502536
Streptomyces sp. NBC_00287	Candidatus Pelagibacter sp. FZCC0015	Streptomyces sp. NBC_00162
Moritella sp. 24	Paenibacillus sp. 32O-W	Rasamsonia emersonii
Cladophialophora carrionii	Sulfurivermis fontis	Ruegeria conchae
Sulfuritortus calidifontis	Streptomyces argyrophyllae	Sulfurihydrogenibium azorense
Vibrio aerogenes	Tolumonas auensis	Gallionella capsiferriformans
Sorangium cellulosum	Porphyromonas endodontalis	Vibrio metoecus
Cellulomonas shaoxiangyii	Methanosarcina thermophila	Chryseobacterium sp. JJR-5R
Rhizorhabdus dicambivorans	Acetobacter tropicalis	Psychrobacter arenosus
Thioclava sp. GXIMD4216	Fusobacterium necrophorum	Nitrospina watsonii
Zunongwangia sp. HGR-M22	Campylobacter gracilis	Cyanobium usitatum
Orrella daihaiensis	Geobacillus zalihae	Weissella paramesenteroides

Pannonibacter phragmitetus	Legionella waltersii	Cylindrospermum stagnale
Gallionella capsiferriformans	Aeromonas caviae	Tepidiforma flava
Jeongeupia sp. HS-3	Penicillium cosmopolitanum	Lentibacillus sp. CBA3610
Agrobacterium sp. MA01	Sphingobacterium mizutaii	Massilia sp. W12
Planctopirus ephydatiae	Microbulbifer variabilis	uncultured Holophaga sp.
Mesorhizobium sp. AR07	Pasteurella skyensis	Caballeronia sp. SBC2
Sphingomonas sp. S2-65	Pseudomassariella vexata	Moraxella haemolytica
Mucilaginibacter mali	Bordetella avium	Francisella adeliensis
Sphingorhabdus sp. SMR4y	Bacteroides heparinolyticus	Mannheimia varigena
Tatumella ptyseos	Synechococcus sp. CBW1108	Achromobacter mucicolens
Alloyangia pacifica	Hymenobacter sublimis	Bibersteinia trehalosi
Streptomyces sp. HUAS YS2	Pleurocapsa sp. FMAR1	Limnochorda pilosa
Sphingopyxis lindanitolerans	Sulfurospirillum cavolei	Methanobrevibacter cuticularis
Cereibacter sphaeroides	Tannerella forsythia	Bordetella genomosp. 9
Desulfosudis oleivorans	Azospira sp. I09	Paracrocinitomix mangrovi
Tropicibacter oceani	Thiolapillus brandeum	Calothrix sp. 336/3
Cryobacterium sp. SO2	Streptomyces roseochromogenus	Helicobacter cinaedi
Ferrovibrio terrae	Flavobacterium magnum	Cryptococcus deuterogattii
Kutzneria sp. CA-103260	[Candida] haemuloni	Dyella thiooxydans
Tsuneonella mangrovi	Halothiobacillus neapolitanus	Arthrobacter sp. zg-Y820
Burkholderia latens	Flagellimonas marinaquae	Tritonibacter scottomollicae
Microbacterium sp. PM5	Limnohabitans sp. 103DPR2	Hyphopichia burtonii
Methylocella silvestris	Mycobacterium spongiae	Arthrobacter sp. Rue61a
Rhizobium gei	Bipolaris maydis	Enterobacter sp. RHBSTW-00593
Paraburkholderia megapolitana	Cohaesibacter sp. ES.047	Staphylococcus agnetis
Agrobacterium vitis	Secundilactobacillus paracollinoides	Paenibacillus sp. BIHB 4019
Pseudorhodoplanes sinuspersici	Maridesulfovibrio hydrothermalis	Halorubrum aethiopicum
uncultured Martelella sp.	Actinobacillus genomosp. 1	Luteibacter anthropi
Rhizophagus irregularis	Halorussus halophilus	Aureobasidium namibiae
Magnetospirillum sp. XM-1	Pseudomonas nitroreducens	Desulfocapsa sulfexigens
Tsuneonella sp. CC-YZS046	Lactobacillus sp. ESL0677	Schizosaccharomyces osmophilus
Phreatobacter cathodiphilus	Corynebacterium rouxii	Gramella sp. MAR_2010_147
Actibacterium sp. EMB200-NS6	Janthinobacterium sp. HH102	Mycobacterium saskatchewanense
Neobacillus sp. YX16	Nitrospina watsonii	Pseudarthrobacter phenanthrenivorans
Brevundimonas sp. SL130	Deinococcus irradiatisoli	Chelatococcus daeguensis
Candida oxycetoniae	Aspergillus nidulans	Photorhabdus asymbiotica
Synechococcus sp. CBW1107	Companilactobacillus futsaii	Coniosporium apollinis
Kickxella alabastrina	Levilactobacillus suantsaii	Streptomyces spongiicola
Leptospira sp. severe_002	Coniophora puteana	Fusarium redolens
Streptomyces sp. NBC_00178	Cardiobacterium sp. Marseille-Q4385	Leptolyngbya sp. O-77
Pseudomonas fluorescens	Lacimicrobium alkaliphilum	Cordyceps militaris

Rhizobium leguminosarum	Maritalea myrionectae	Halobacterium salinarum
Auraticoccus monumenti	Petrimonas sp.	Actinoplanes sp. N902-109
Aquibium microcysteis	Psychrobacillus sp. AK 1817	Nocardia yunnanensis
Hydrogenophaga sp. PAMC20947	Duncaniella dubosii	Brevundimonas sp. 'scallop'
Thermomonospora curvata	Aureobasidium subglaciale	Paenibacillus sp. R14(2021)
Sphingomonas sabuli	Thermococcus gorgonarius	Streptomyces sp. DSM 40868
Nitrospirillum amazonense	Enterococcus saccharolyticus	Arthrobacter sunyaminii
Acuticoccus sp. I52.16.1	Petrimonas mucosa	Hymenobacter sp. GOD-10R
Azospirillum oryzae	Trichoderma citrinoviride	Flavobacterium sp. KACC 22758
Ensifer adhaerens	Zobellia galactanivorans	Paenibacillus psychroresistens
Bradyrhizobium guangxiense	Dissulfurimicrobium hydrothermale	Paralysiella testudinis
Paucibacter sp. S2-9	Parachlamydia acanthamoebae	Mycolicibacterium confluentis
Roseitalea porphyridii	Nodosilinea sp. E11	Streptomyces sp. SM18
Chryseobacterium sp. T16E-39	Haloterrigena salifodinae	Conexibacter sp. S30A1
Pseudomonas sp. GCEP-101	Thermosulfurimonas marina	Marinobacter salarius
Microvirga sp. VF16	Herbaspirillum sp. meg3	Brevundimonas sp. SL130
Candidatus Phaeomarinobacter ectocarpi	Leclercia sp. W17	Pseudolysobacter antarcticus
Paucibacter sp. KCTC 42545	Megasphaera massiliensis	Chitinophaga sp. XS-30
Caulobacter rhizosphaerae	Enterobacter sp. PGRG2	Nitrosomonas eutropha
Penicillium griseofulvum	Acinetobacter equi	Desulfobulbus oligotrophicus
Thyridium curvatum	Pontibacter korlensis	Streptomyces sp. NBC_01230
Lysobacter avium	uncultured Amphritea sp.	Mobiluncus curtisii
Burkholderia sp. MSMB0856	Lactobacillus sp. ESL0785	Burkholderia sp. PAMC 26561
Haloarchaeobius sp. HME9146	Arthrobacter sp. 24S4-2	Dickeya fangzhongdai
Pseudomonas sp. MPC6	Anaerococcus prevotii	Achromobacter pestifer
Microbacterium sp. Y-01	Otariodibacter oris	Trinickia caryophylli
Candidatus Methanoplasma termitum	Arthrobacter sp. DNA4	Penicillium macrosclerotiorum
Microbacterium suwonense	Moraxella cuniculi	Paracoccus jeotgali
Microbacterium sp. M28	Streptomyces sp. NBC_01296	Exiguobacterium alkaliphilum
Nocardioides sp. WS12	Mycolicibacter minnesotensis	Jeongeupia sp. HS-3
Microbacterium sp. ET2	Vibrio zhugei	Shewanella algae
Halobaculum sp. DT92	Porphyromonas somerae	Christiangramia salexigens
Thermococcus sp. Bubb.Bath	Aeoliella mucimassa	Streptomyces finlayi
Clavibacter michiganensis	Anaerococcus vaginalis	Tropicibacter oceani
Alphabaculovirus chorosaceanae	Ereboglobus luteus	Auraticoccus monumenti
uncultured Hyphomonas sp.	Venatoribacter cucullus	Winogradskyella sp. PG-2
Halogranum amylolyticum	Selenomonas sp. oral taxon 478	Cryptococcus wingfieldii
Aspergillus thermomutatus	Haladaptatus sp. DYF46	Pseudomonas oryzae
Ferroglobus placidus	Porphyromonas cangingivalis	Marinobacter sp. es.042
Pseudomonas thivervalensis	Hydrogenophaga sp. NH-16	Streptomyces sp. NBC_00385
Halogeometricum sp. S1BR25-6	Pontibacter actiniarum	Bacillus carboniphilus

Lelliottia amnigena	Halarchaeum grantii	Phytobacter ursingii
Purpureocillium lilacinum	Thalassolituus oleivorans	Natronococcus sp.
Janibacter sp. A1S7	Fonsecaea nubica	Thiobacillus denitrificans
Halolamina rubra	Streptomyces sp. JB150	Bacillus paranthracis
Microbacterium sp. zg-B185	Vreelandella venusta	Ralstonia pseudosolanacearum
Hydrogenophaga sp. BPS33	Limosilactobacillus pontis	Methanohalophilus levihalophilus
Haladaptatus sp. DJG-WS-42	Limosilactobacillus gastricus	Pseudomonas sp. SG20056
Sphingomonas sp. J315	Halopenitus persicus	Mycoplasma anserisalpingitidis
Sphingomonas sp. HDW15A	Lactobacillus helveticus	Candidatus Thiodictyon syntrophicum
Corynebacterium glaucum	Thermomonas paludicola	Stenotrophomonas oahuensis
Brevundimonas sp. NIBR11	Saccharolobus caldissimus	Flammeovirga pectinis
Corynebacterium timonense	Arthrobacter sp. StoSoilA2	Arthrobacter sp. PM3
Mycobacterium sp. ITM-2016-00318	Legionella fallonii	Candidatus Methylopumilus planktonicus
Candidatus Planktophila dulcis	Actinobacillus suis	Candidatus Symbiobacter mobilis
Burkholderia aenigmatica	Spirosoma sp. KUDC1026	Chloroflexus aggregans
Gordonia sp. PP30	Acidisarcina polymorpha	Limnochorda sp. LNt
Sphingomonas ginsengisoli An et al. 2013	Parascardovia denticolens	Parasedimentitalea psychrophila
Streptomyces capitiformicae	Candidatus Sulfuricurvum sp. RIFRC-1	Shinella oryzae
Salmonella sp. SA14318	Acidithiobacillus sp. AMEEHan	Pseudooceanicola algae
Heterobasidion irregulare	Neisseria musculi	Vibrio pectenicida
Spirillospora sp. NBC_00431	Spirosoma aerolatum	Syntrophus aciditrophicus
Cellulomonas xiejunii	Nostoc sp. C052	Aquitalea magnusonii
Sphingobium sp. TKS	Hymenobacter sp. GOD-10R	Chryseobacterium capnotolerans
Maribacter sp. MJ134	Colletotrichum siamense	Daldinia childiae
Pseudomonas umsongensis	Natronococcus occultus	Photobacterium leiognathi
Kosakonia radicincitans	Haloglomus salinum	Synchytrium microbalum
Sphingomonas sp. LM7	Chlorobaculum tepidum	Fastidiosipila sanguinis
Rathayibacter rathayi	Lactobacillus isalae	Meiothermus ruber
Pseudomonas laurylsulfatiphila	Hymenobacter volaticus	Vibrio artabrorum
Nocardioides sp. InS609-2	Flavobacterium album	Mycolicibacter terrae
Microbacterium endophyticum	Pseudomonas sp. S34	Candidatus Nanopelagicus abundans
Pseudomonas sp. DR48	Methanobrevibacter arboriphilus	Streptomyces sp. NBC_00576
Rhodococcus sp. B7740	Colletotrichum abscissum	Brasilonema sennae
Brachybacterium avium	Prevotella herbatica	Labrenzia sp. CP4
Curtobacterium sp. C1	Hymenobacter sp. YIM 151500-1	Novacetimonas hansenii
Flavobacterium sp. 20NA77.7	Streptomyces sp. NBC_01261	Desulforapulum autotrophicum
Actinoplanes friuliensis	Anaerococcus obesiensis	Salinimonas iocasae
Croceicoccus sp. Ery15	Enterobacter sp. HK169	Polaribacter vadi
Stutzerimonas stutzeri	Pontibacter kalidii	Aeromonas schubertii
Corynebacterium flavescens	Lactobacillus sp. ESL0684	Nocardioides sp. dk884
Leptothrix cholodnii	Enterobacter sp. S-33	Aquiluna borgnonia

Devosia algicola	Psychroflexus torquis	Aromatoleum petrolei
Bradyrhizobium sp. TM102	Limosilactobacillus panis	Lodderomyces elongisporus
Rathayibacter oskolensis	Methanoregula formicica	uncultured Acetobacterium sp.
Microbacterium sp. zg-Y818	Halomicroarcula limicola	Blattabacterium cuenoti
Streptomyces netropsis	Pseudomonas sp. PDNC002	Sphaerulina musiva
Streptomyces sp. ITFR-21	Lactobacillus sp. 3B(2020)	Prosthecochloris aestuarii
Exserohilum turcicum	Niallia sp. XMNu-256	Candidatus Thiothrix anitrata
Planctomyces sp. SH-PL14	Prevotella corporis	Bizionia sp. M204
Candidatus Nanopelagicus limnes	Pseudomonas sp. TUM22785	Pseudomonas sp. LS44
Archangium gephyra	Muricauda sp. SCSIO 65647	Xenorhabdus poinarii
Nonomuraea glycinis	Klebsiella africana	Bacidia gigantensis
Herbiconiux sp. L3-i23	Hoylesella buccalis	Luteolibacter sp. SL250
Streptomyces sp. NBC_01224	Corynebacterium crudilactis	Desulfococcus multivorans
Thiomonas sp. X19	Flavobacterium sp. B183	Nocardioides sp. S-1144
Demequina sp. OYTSA14	Segatella oris	Candidatus Pelagisphaera phototrophica
Arthrobacter sp. FW306-2-2C-D06B	Lysinibacillus sp. FSL K6-3209	Sinomicrobium kalidii
Brevundimonas sp. NIBR10	Prevotella fusca	Sphingobacterium hotanense
Corynebacterium mustelae	Prevotella scopos	Chloracidobacterium validum
Variovorax sp. PBS-H4	Corynebacterium kefirresidentii	Methanothermococcus okinawensis
Pseudomonas fuscovaginae	Dialister hominis	Persicobacter psychrovividus
Kribbella sp. CA-293567	Enterobacter sp. RHBSTW-00975	Zhaonella formicivorans
Nocardioides sp. JQ2195	Abyssicoccus albus	Halopseudomonas salegens
Mesorhizobium sp. M1D.F.Ca.ET.043.01.1.1	Lactobacillus ultunensis	Geomonas sp. RF6
Jannaschia sp. GRR-S6-38	Segatella bryantii	Methanosarcina siciliae
Saccharomyces eubayanus	Prevotella sp. Rep29	Pseudoalteromonas distincta
Sphingopyxis sp. GC21	Lactobacillus johnsonii	Pseudazoarcus pumilus
Hartmannibacter diazotrophicus	Colwellia sp. 20A7	Histoplasma mississippiense (nom. inval.)
Phenylobacterium zucineum	Prevotella sp. oral taxon 475	Saprospira grandis
Cellulomonas sp. NTE-D12	Lactobacillus gasseri	Halomonas meridiana
Georgenia yuyongxinii	Scardovia inopinata	Thermanaerovibrio acidaminovorans
Amycolatopsis sp. NBC_01286	Limosilactobacillus reuteri	Limnochorda sp. L945t
Lysinibacillus fusiformis	Prevotella sp. E13-17	Mycena indigotica
Isoptericola variabilis	Ligilactobacillus acidipiscis	Suillus clintonianus
Altererythrobacter epoxidivorans	Prevotella multiformis	Aquimarina sp. AD10
Microbacterium sp. NIBRBAC000506063	Streptomyces sp. NBC_00151	Vibrio rhizosphaerae
Methylobacterium sp. C1	Hoylesella enoeca	Methylophaga pinxianii
Corynebacterium humireducens	Limosilactobacillus frumenti	Kordia antarctica
Schizosaccharomyces japonicus	Limosilactobacillus vaginalis	Pseudorhizobium flavum
Janibacter cremeus	Enterobacter sp. ODB01	Vibrio quintilis
Mycobacterium gordonae		Ruficoccus sp. ZRK36
Mycolicibacterium phocaicum		Arthrobacter sp. YC-RL1

Deinococcus geothermalis		Emericellopsis cladophorae
Microbacterium invictum		Aquimarina sp. TRL1
Allokutzneria albata		Nitratireductor sp. OM-1
Streptomyces jietaisiensis		Sinorhizobium numidicum
Sulfitobacter sp. THAF37		Stenotrophomonas indicatrix
Nakamurella antarctica		Desulfolutivibrio sulfodismutans
Priestia koreensis		Roseobacter ponti
Pseudomonas sp. LS44		Corynebacterium aurimucosum
Streptosporangium roseum		Pigmentiphaga aceris
Dermatophilus congolensis		Streptomyces platensis
Micromonospora sp. WMMA1998		Microbulbifer celer
Rhodoluna lacicola		Isosphaera pallida
Diaphorobacter nitroreducens		Teredinibacter haidensis
Microbacterium sp. CGR1		Microbulbifer hydrolyticus
Amycolatopsis sp. BJA-103		Kribbella sp. NBC_01245
Microbacterium schleiferi		Magnetococcus marinus
Pseudomonas sp. B21-019		Leuconostoc pseudomesenteroides
Kribbella qitaiheensis		Paracoccus saliphilus
Jejubacter calystegiae		Thermoanaerobacter sp. X514
Agrococcus sp. SCSIO52902		Geosporobacter ferrireducens
Acaromyces ingoldii		Thalassovita sp.
Rhizorhabdus wittichii		Sulfuricystis thermophila
Streptomyces rapamycinicus		Ammonifex degensii
Streptomyces sp. NBC_01280		Aquiflexum balticum
Curtobacterium sp. SGAir0471		Penicillium cataractarum
Rhodanobacter sp. AS-Z3		Aliamphritea spongicola
Kitasatospora sp. NBC_01246		Telmatocola sphagniphila
Mycobacterium sp. JS623		Streptomyces decoyicus
Nocardia farcinica		Spirobacillus cienkowskii
Microbacterium sp. 1S1		Aspergillus nidulans
Bartonella alsatica		Roseibacterium elongatum
Cupriavidus sp. USMAA2-4		Marinobacterium sp. LSUCC0821
Sanguibacter sp. 4.1		Undibacterium parvum
Mycolicibacter virginiensis		Halorussus marinus
Microbacterium sp. YJN-G		Alternaria metachromatica
Cryobacterium arcticum		Solitalea lacus
Streptomyces sp. NBC_01431		Acinetobacter wuhouensis
Yersinia massiliensis		Pseudomassariella vexata
Parasphingorhabdus sp. SCSIO 66989		Streptantibioticus cattleyicolor
Microlunatus soli		Thalassomonas viridans
Cronobacter universalis		Erysipelothrix sp. HDW6A

Halomonas sp. CKK8		Truepera radiovictrix
Xylanimonas protaetiae		Achromobacter sp. AONIH1
Acinetobacter schindleri		Synechococcus sp. PROS-9-1
Conexibacter sp. DBS9H8		Hirsutella rhossiliensis
Variovorax sp. RA8		Sulfuriflexus mobilis
Natronomonas halophila		uncultured Vibrio sp.
Corynebacterium auriscanis		Apiotrichum porosum
Maricaulis maris		Gordonia terrae
Lysobacter capsici		Kitasatospora herbaricolor
Kitasatospora sp. HUAS 3-15		Sutcliffiella horikoshii
Streptomyces olivoreticuli		Maribacter sp. MJ134
Erythrobacter sp. SCSIO 43205		Paraburkholderia phymatum
Rhodococcus triatomae		Salinicola tamaricis
Comamonas endophytica		Streptomyces ficellus
Microbacterium sp. nov. GSS16		Austwickia chelonae
Cellulomonas wangsupingiae		Francisella tularensis
Streptomyces sp. NBC_01296		Kaistella antarctica
Rosistilla oblonga		Purpureocillium takamizusanense
Lysobacter sp. S4-A87		Gimesia panareensis
Brachybacterium vulturis		Nitrosococcus watsonii
Halococcus sp. PRR34		Kordiimonas sp. SCSIO 12603
Aeromicrobium erythreum		Desulfoglaeba alkanexedens
Dermacoccus nishinomiyaensis		Rahnella sikkimica
Actinomyces marmotae		Ferribacterium limneticum
Oceanicola sp. D3		Nitrogeniibacter aestuarii
Streptomyces sp. NBC_01237		Methanolobus sediminis
Microbacterium oxydans		Colletotrichum gloeosporioides
Uncinocarpus reesii		Stanieria cyanosphaera
Bradyrhizobium sp. CCBAU 53340		Sulfitobacter sp. SK012
Thermococcus sp. 21S7		Prochlorococcus marinus
Streptomyces sp. NBC_00273		Halioglobus japonicus
Microbacterium neungamense		Acinetobacter bouvetii
Aeromonas veronii		Verrucosispora sp. WMMD1129
Azospirillum sp. TSH58		Paenibacillus protaetiae
Lysobacter lycopersici		Pseudoduganella albidiflava
Arthrobacter woluwensis		Erwinia tasmaniensis
Fluviicola taffensis		Streptomyces sp. NBC_01317
Shinella sp. XGS7		Pseudoduganella sp. SL102
Nocardioides piscis		Mycoplasmopsis glycophila
Verminephrobacter eiseniae		Aureobasidium subglaciale
Halovivax cerinus		Cyclobacterium marinum

Rhodococcus sp. DMU1		Rhizobium sp. 11515TR
Corynebacterium sp. sy039		Candida dubliniensis
Mycolicibacterium pallens		Dechloromonas sp. HYN0024
Lichenihabitans psoromatis		Micromonospora sp. WMMD812
Nocardia huaxiensis		Halotia branconii
Plantibacter flavus		Chromohalobacter salexigens
Rubrivivax gelatinosus		Hymenobacter sp. YIM 151858-1
Actinacidiphila glaucinigra		Thermothelomyces thermophilus
Streptomyces sp. NBC_01217		Postia placenta
Corynebacterium ulcerans		Virgibacillus sp. MSP4-1
Marispirochaeta sp.		Orrella daihaiensis
Nocardioides cynanchi		Streptomyces sp. NBC_00287
Polymorphospora rubra		Trichocoleus desertorum
Chromobacterium subtsugae		Mangrovivirga cuniculi
Geomonas oryzisoli		Pseudomonas sp. S34
Rhodococcus sovatensis		Halopenitus persicus
Bernardetia sp. ABR2-2B		Streptomyces sp. HUAS CB01
Corynebacterium terpenotabidum		Pseudomonas sp. TMP25
Curtobacterium sp. MCLR17_032		Aspergillus melleus
Salinibacterium sp. ZJ70		Chromobacterium rhizoryzae
Microbacterium lushaniae		Deinococcus geothermalis
Aeromicrobium choanae		Pseudomonas sp. DTU12.3
Cellulomonas sp. NS3		Clavispora lusitaniae
Corynebacterium testudinoris		uncultured Nitrososphaera sp.
Streptosporangium sp. 'caverna'		Microbacterium sp. ET2
Leptolyngbya sp. O-77		Mesorhizobium opportunistum
Gordonia hydrophobica		Pseudomonas sediminis
Xanthomonas phaseoli		Tilletiopsis washingtonensis
Nocardia cyriacigeorgica		Caballeronia sp. SL2Y3
Nitrospira japonica		Sulfurospirillum halorespirans
Microbacterium sp. H1-D42		Spongiibacter sp. IMCC21906
Rossellomorea sp. y25		Natronobacterium texcoconense
Streptomyces sp. NBC_01216		Candidatus Sulfurimonas marisnigri
Corynebacterium gottingense		Rhizobium gei
Psychrobacter sp. WB2		Sphingobium sp. YBL2
Pseudomonas promysalinigenes		[Mycoplasma] gypis
Halopseudomonas nanhaiensis		Erwinia sp. QL-Z3
Sanguibacter sp. HDW7		Sinorhizobium sp. CCBAU 05631
Variovorax paradoxus		Pseudomonas sp. PDNC002
Micromonospora maris		Kalmanozyma brasiliensis
Streptomyces sp. Q6		Halomarina sp. PSRA2

Mycolicibacterium aurum		Rhodococcus sp. USK13
Limosilactobacillus vaginalis		Bradyrhizobium betae
Kitasatospora aureofaciens		Lachancea lanzarotensis
Comamonas piscis		Methylobacterium phyllosphaerae
Lactobacillus johnsonii		Mycobacterium leprae
Mesorhizobium ciceri		Pseudomonas sp. ADAK13
Arthrobacter sp. QXT-31		Sphingomonas radiodurans
Starkeya sp. ORNL1		Ferrovibrio terrae
Stenotrophomonas sp. BIO128-Bstrain		Chryseobacterium sp. T16E-39
Sphingobium sp. AP49		Pseudooceanicola spongiae
Pseudomonas sp. DC1.2		Candidatus Planktophila limnetica
Betabaculovirus spliturae		Bosea sp. ANAM02
Kosakonia oryzae		Fonsecaea erecta
Pseudomonas toyotomiensis		Bradyrhizobium sp. NP1
Ornithinimicrobium flavum		Streptomyces sp. HUAS CX7
Micromonospora sp. NBC_01796		Sphingomonas sp. AAP5
Cellulomonas wangleii		Nocardia otitidiscaviarum
Burkholderia glumae		Ceraceosorus guamensis
Variovorax boronicumulans		Pseudomonas monteilii
Corynebacterium aquilae		Polynucleobacter sp. TSB-Sco08W16
Cnuibacter physcomitrellae		Mesorhizobium mediterraneum
Ralstonia sp. RRA.1		Mesorhizobium terrae
Streptomyces antibioticus		Salinilacihabitans rarus
Ornithinimicrobium cryptoxanthini		Thermococcus sp. 21S7
Cellulomonas flavigena		Fusarium subglutinans
Streptomyces liliifuscus		Pseudomonas idahonensis
Piscinibacter gummiphilus		Bradyrhizobium diazoefficiens
Actinomadura madurae		Microbacterium schleiferi
Pseudocitrobacter corydidari		Novosphingobium olei
Sinomonas atrocyanea		Geobacillus genomosp. 3
Georgenia sp. M64		Bradyrhizobium sp. 1(2017)
Pasteurella skyensis		Bosea sp. NBC_00550
Mycobacterium decipiens		Serratia odorifera
Roseivivax marinus		Bosea sp. (in: a-proteobacteria)
Microbacterium chengjingii		Bacillus sp. JS
Geothrix sp. PMB-07		Spirosoma rhododendri
Metschnikowia bicuspidata		Ferroplasma sp. Type II
Curtobacterium sp. MCBD17_040		Methanocella sp.
Flaviflexus salsibiostraticola		Salinadaptatus halalkaliphilus
Actinomyces sp. HMT897		Halorubrum sp. Atlit-26R
Sphingomonas sp. KRR8		Bosea sp. F3-2

Mycobacterium tuberculosis		Streptomyces sp. ST1015
Bartonella henselae		Bacillus cytotoxicus
Corynebacterium glutamicum		Sphingobium sp. JS3065
Marinobacter sp. CA1		Halococcus sediminicola
Paracoccus sanguinis		Rhodopseudomonas boonkerdii
Paraoerskovia sediminicola		Natronoarchaeum rubrum
Mycobacterium colombiense		Modestobacter marinus
Mesorhizobium sp. INR15		Novosphingobium sp. THN1
Microcella indica		Rhizobium sp. BT04
Coniophora puteana		Sphingomonas sp. J315
Corynebacterium cyclohexanicum		Winogradskyella sediminis
Microbacterium hydrocarbonoxydans		Microbacterium lushaniae
Niveibacterium microcysteis		Mycoplasma sp. 888
Caballeronia sp. Lep1P3		Paenibacillus rhizovicinus
Roseateles sp. XES5		Cupriavidus sp. USMAHM13
Streptomyces sp. NBC_00243		Bradyrhizobium lablabi
Aeromicrobium duanguangcaii		Bradyrhizobium sp. ORS 278
Corynebacterium faecale		Amylibacter sp. IMCC11727
Photobacterium sp. TY1-4		Bradyrhizobium sp. BWA-3-5
Silicimonas algicola		Streptomyces sp. NA03103
Trueperella abortisuis		Novosphingobium sp. 9
Exophiala dermatitidis		Spirochaeta africana
Paraburkholderia caffeinilytica		Mycoplasmopsis columbina
Nocardia yunnanensis		Streptomyces sp. VN1
Halococcus qingdaonensis		Bosea vaviloviae
Corynebacterium hesseae		Microbacterium arborescens
Streptomyces phaeoluteigriseus		Alysiella filiformis
Aspergillus wentii		Sanguibacter sp. 4.1
Microcella sp.		Mesorhizobium sp. 131-3-5
Mycobacterium sp. MS1601		Pseudomonas sp. PSKL.D1
Streptomyces durmitorensis		Bradyrhizobium sp. 200
Pseudomonas sp. NIBR-H-19		Halocatena marina
Mycolicibacterium sp. MU0053		Meira miltonrushii
Microbacterium hominis		Bradyrhizobium roseus
Arthrobacter koreensis		Salimicrobium jeotgali
Nesterenkonia pannonica		Deinococcus actinosclerus
Actinomadura verrucosospora		Bosea sp. AS-1
Leucobacter luti		Pseudoalteromonas sp. HL-AS1
Saccharopolyspora gloriosae		Brevibacterium sp. CS2
Agromyces badenianii		Bradyrhizobium brasilense
Mycobacterium heckeshornense		Natronosalvus caseinilyticus

Acidipropionibacterium jensenii		Rubrobacter indicoceani
Corynebacterium crudilactis		Pseudomonas sp. IzPS59
Leucobacter sp. Psy1		Novosphingobium sp. ES2-1
Trueperella bernardiae		Sulfurovum sp. NBC37-1
Mycolicibacterium tokaiense		Xanthomonas theicola
Schaalia sp. JY-X169		Bradyrhizobium sp. CIAT3101
Sinomonas sp. R1AF57		Microbacterium aurum
Ancylobacter polymorphus		Wielerella bovis
Leucobacter triazinivorans		Aspergillus thermomutatus
Erythrobacter sp. 3-20A1M		[Pseudomonas] carboxydohydrogena
Actinomyces lilanjuaniae		Rhizobium sp. SL42
Kineococcus sp. NBC_00420		Bradyrhizobium sp. SEMIA
Microlunatus sagamiharensis		Sphingomonas profundi
Arthrobacter jiangjiafuii		Bradyrhizobium icense
Streptomyces canus		Novosphingobium sp. EMRT-2
Mycolicibacterium aubagnense		Halorussus lipolyticus
Pararhizobium sp. YC-54		Streptomyces sp. NBC_01725
Luteipulveratus mongoliensis		Bradyrhizobium sp. C-145
Pseudarthrobacter sp. NIBRBAC000502772		Natronolimnobius baerhuensis
Cryobacterium sp. LW097		Mesorhizobium sp. B2-1-1
Pseudarthrobacter sp. MM222		Aspergillus luchuensis
Paracoccus mutanolyticus		Halalkaliarchaeum desulfuricum
Thiorhodovibrio litoralis		Neobacillus sp. OS1-33
Cedecea neteri		Microbacterium sp. 4R-513
Corynebacterium argentoratense		Paenibacillus thiaminolyticus
Mycolicibacterium fluoranthenivorans		Vibrio casei
Variovorax sp. PAMC26660		Amycolatopsis sp. NBC_01286
Marinobacter sp. CP1		Methylophilus methylotrophus
Schaalia turicensis		Chromobacterium sp. Beijing
Natrinema altunense		Gloeothece citriformis
Aureispira sp. CCB-E		Bradyrhizobium sp. CB1650
Microbacterium caowuchunii		Bosea vestrisii
Microbacterium binotii		Caulobacter soli
Leclercia pneumoniae		Paraburkholderia terrae
Georgenia wutianyii		Paenibacillus antarcticus
Mycolicibacterium fortuitum		Bradyrhizobium sp. NDS-1
Cellulophaga omnivescoria		Corynebacterium auris
Arthrobacter sp. NEB 688		Natronolimnobius sp. AArcel1
Streptococcus sp. zg-86		Methanosalsum natronophilum
Cellulomonas sp. Y8		Rhodococcus sp. P1Y
Algoriphagus sp. NBT04N3		Bradyrhizobium sp. SK17

Aurantimicrobium sp. MWH-Uga1		Campylobacter armoricus
Corynebacterium variabile		Bradyrhizobium sp. 41S5
Microbacterium esteraromaticum		Aeromonas sp. CA23
Cryobacterium breve		Bradyrhizobium elkanii
Rathayibacter sp. VKM Ac-2760		Secundilactobacillus malefermentans
Arthrobacter sp. StoSoilB5		Bradyrhizobium cosmicum
Crossiella sp. CA-258035		Bradyrhizobium quebecense
Buttiauxella sp. 3AFRM03		Agromyces flavus
Microbacterium sp. ABRD28		Bradyrhizobium sp. 2S1
Streptomyces chartreusis		Hydrogenimonas urashimensis
Blastochloris tepida		Proteus sp. NMG38-2
Variovorax sp. PBL-H6		Bosea sp. Tri-49
Streptomyces sp. NBC_01727		Halobacterium hubeiense
Bradyrhizobium sp. 195		Pseudomonas hefeiensis
Actinoplanes sp. NBC_00393		Sphingomonas sp. HMP6
Curtobacterium sp. 458		Bradyrhizobium arachidis
Streptomyces sp. NBC_00335		Bradyrhizobium sp. CCBAU 53421
Bordetella genomosp. 13		Gordonia alkanivorans
Stenotrophomonas acidaminiphila		Streptomyces sp. LRE541
Halosegnis marinus		Planctoellipticum variicoloris
Polaromonas naphthalenivorans		Serratia surfactantfaciens
Schaalia sp. 19OD2882		Akkermansia glycaniphila
Streptococcus merionis		Bradyrhizobium australafricanum
Pseudonocardia sp. DSM 110487		Methylomusa anaerophila
Paraoerskovia marina		Novosphingobium capsulatum
Streptomyces bathyalis		Halolamina rubra
Streptomyces scopuliridis		Pseudomonas sp. RSB 5.4
Microcella humidisoli		[Candida] duobushaemulonis
Arcanobacterium canis		Halorubrum yunnanense
Pseudomonas tructae		Acinetobacter sp. MYb10
Paeniglutamicibacter sp. ZC-3		Methylophilus sp. TWE2
Microlunatus elymi		Avibacterium sp. 21-595
Streptomyces sp. HM190		Thermococcus sp. LS1
Dickeya zeae		Bradyrhizobium xenonodulans
Serpula lacrymans		Curtobacterium sp. MCSS17_007
Rhodococcus pseudokoreensis		Mesorhizobium sp. M2A.F.Ca.ET.046.03.2.1
Salinibacterium sp. M195		Schaalia sp. JY-X169
Arthrobacter caoxuetaonis		Moraxella bovis
Desulfovibrio ferrophilus		Synechococcus sp. CB0101
Streptomyces sp. NBC_01230		Vibrio europaeus
Haloarchaeobius litoreus		Streptomyces sp. JUS-F4

Sporisorium graminicola		Roseovarius sp. THAF8
Microbacterium fandaimingii		Borrelia miyamotoi
Variovorax sp. 38R		Bradyrhizobium sp. CCBAU 53340
Streptomyces atratus		Bradyrhizobium sp. 186
Nocardia sp. NBC_01503		Endozoicomonas sp. 8E
Agromyces marinus		Nostoc flagelliforme
Micromonospora sp. HUAS LYJ1		Bartonella alsatica
Xanthomonas euvesicatoria		Thermus aquaticus
Acidaminococcus fermentans		Candidatus Profftia sp. (ex Adelges kitamiensis)
Actinobaculum sp. 313		Thalassotalea piscium
Corynebacterium suedekumii		Janthinobacterium sp. 1_2014MBL_MicDiv
Rhodococcus sp. NBC_00297		Hyphomonas sp. KY3
Dietzia sp. B32		Lonsdalea populi
Cedecea sp. FDAARGOS_727		Halobium salinum
Agromyces archimandritae		Kangiella aquimarina
Botrimarina mediterranea		Atlantibacter hermannii
Leucobacter insecticola		Microbacterium sp. SL75
Mesorhizobium sp. M4B.F.Ca.ET.058.02.1.1		Leuconostoc falkenbergense
Candidatus Pelagibacter sp. FZCC0015		Methylocystis bryophila
Propionibacterium freudenreichii		Bradyrhizobium sp. BEA-2-5
Labilithrix luteola		Bradyrhizobium sp. 6(2017)
Tsukamurella pulmonis		Brevundimonas sp. Bb-A
Streptococcus sanguinis		Streptomyces uncialis
Corynebacterium hindlerae		Burkholderia sp. IDO3
Microcella daejeonensis		Sulfurimonas marina
Agromyces laixinhei		Pseudomonas sp. Z8(2022)
Helcobacillus massiliensis		Halorubellus sp. PRR65
Streptomyces sp. HUAS 2-6		Bradyrhizobium pachyrhizi
Escherichia fergusonii		Mycoplasma bradburyae
Mycolicibacterium rhodesiae		Sporosarcina sp. P37
Mycobacteroides chelonae		Fonsecaea monophora
Streptomyces sp. NBC_01450		Micromonospora peucetia
Brevibacterium linens		Sphingomonas qomolangmaensis
Mycobacterium kansasii		Halalkalicoccus sp. SEDH24
Thioflavicoccus mobilis		Bradyrhizobium sp. CCBAU 53338
Dickeya dianthicola		Haloarcula sp. DT43
Saccharothrix syringae		Curtobacterium sp. C1
Mycetocola zhujimingii		Dietzia sp. oral taxon 368
Capronia epimyces		Methanosphaera sp. BMS
Halobacterium bonnevillei		Thalassococcus sp. S3
Natrialba sp. PRR66		Alternaria infectoria

Agromyces protaetiae		Mariniplasma anaerobium
Streptomyces sp. MA3_2.13		Bradyrhizobium ottawaense
Microbacterium elymi		Halovenus carboxidivorans
Actinoplanes sichuanensis		Pseudoalteromonas agarivorans
Staphylococcus aureus		Idiomarina piscisalsi
Herbiconiux sp. SALV-R1		Mesorhizobium sp. WSM1497
Arthrobacter sp. zg-Y820		Streptomyces coralus
Oceanisphaera sp. IT1-181		Gordonia amicalis
Ornithinimicrobium pratense		Thiothrix winogradskyi
Nocardia puris		Rhodococcus sp. NBC_00294
Corynebacterium occultum		Alicyclobacillus cycloheptanicus
Pseudomonas gozinkensis		Corynebacterium confusum
Curtobacterium sp. DN_7.5		Pseudomonas sp. B21-028
Pseudomonas argentinensis		Thalassospira indica
Leucobacter muris		Pseudomonas sp. FJ2-5-13
Micrococcus luteus		Microbacterium sp. KUDC0406
Acinetobacter sp. AS23		Salinibacterium sp. dk2585
Curtobacterium sp. L6-1		Microbacterium sp. BH-3-3-3
Agromyces mangrovi		Bradyrhizobium septentrionale
Curtobacterium flaccumfaciens		Halarcobacter ebronensis
Haloterrigena sp. KLK7		Bacillus spizizenii
Hymenobacter sp. 5317J-9		Leminorella richardii
Gryllotalpicola protaetiae		Nostoc sp. PCC 7120 = FACHB-418
Barrientosiimonas endolithica		Amycolatopsis sp. QT-25
Serratia liquefaciens		Rhodococcus triatomae
Streptomyces sp. NBC_00316		Paenalcaligenes hominis
Corynebacterium efficiens		Pseudomonas tructae
Janibacter indicus		Azospirillum baldaniorum
Leisingera thetidis		Thermomonospora curvata
Rhizobium sp. NXC24		Pseudomonas thivervalensis
Kytococcus sedentarius		Sporothrix schenckii
Enterobacter kobei		Kocuria sp. KD4
Clavibacter sepedonicus		Methanohalophilus halophilus
Enterobacter cloacae complex sp. FDA-CDC-AR_0164		Halobaculum sp. YSMS11
Streptococcus sp. 29896		Halarcobacter bivalviorum
Citricoccus muralis		Spiroplasma alleghenense
Flavobacterium nitratireducens		Peribacillus asahii
Acinetobacter sp. WCHAc010034		Paenibacillus sp. FSL M7-0896
Streptococcus sp. HN38		Streptomyces sp. V17-9
Enterobacter sp. R4-368		Halobaculum sp. DT31
Pseudomonas guguanensis		Flavobacterium sp. LPB0248

Pseudonocardia abyssalis		Streptomyces peucetius
Cupriavidus pinatubonensis		Leptospira sp. GIMC2001
Cellulomonas dongxiuzhuiae		Staphylococcus condimenti
Haloplanus sp. HW8-1		Candidatus Vondammii sp. HM_W22
Schaalia radingae		Pseudomonas sp. B21-032
Streptomyces griseus		Endomicrobium proavitum
Streptococcus australis		Coxiella endosymbiont of Amblyomma nuttalli
Microbacterium sp. CBA3102		Paenibacillus barcinonensis
Streptomyces sp. NBC_01255		Streptomyces sp. NBC_00304
Trueperella pyogenes		Rhodococcus qingshengii
Avibacterium paragallinarum		Chlamydia serpentis
Streptococcus rubneri		Hymenobacter sp. BT18
Boudabousia tangfeifanii		Brevibacterium siliguriense
Streptomyces sp. NBC_00536		Pseudomonas sp. MPC6
Roseivirga spongicola		Pectobacterium atrosepticum
Aspergillus aculeatus		Spiroplasma diminutum
Pantoea vagans		Microbacterium sp. 10M-3C3
Variovorax sp. RKNM96		Arthrobacter sp. D5-1
Micromonospora sp. WMMD882		Halorussus salinisoli
Nocardioides baekrokdamisoli		Parvularcula bermudensis
Agromyces flavus		Janthinobacterium sp. TB1-E2
Mycolicibacter terrae		Penicillium rubens
Actinomyces gaoshouyii		Vibrio sp. YMD68
Demequina sediminis		Neorickettsia findlayensis
Aeromonas media		Kosakonia sacchari
Mycetocola sp. JXN-3		Halococcus sp. IIIV-5B
Comamonas flocculans		Occallatibacter riparius
Saccharopolyspora erythraea		Zunongwangia sp. HRR-M8
Dietzia cinnamea		Francisella philomiragia
Microterricola viridarii		Aspergillus costaricensis
Serratia ficaria		Rathayibacter sp. VKM Ac-2760
Thermobispora bispora		Aquicella lusitana
Halovivax ruber		Borreliella japonica
Thermomonas brevis		Rodentibacter caecimuris
Brevibacterium zhoupengii		Candidatus Thioglobus autotrophicus
Actinobacillus equuli		Bombyx mori iflavirus
Streptococcus constellatus		Paenibacillus aurantius
Corynebacterium freiburgense		Corynebacterium camporealensis
Enterobacter sp. PGRG2		Methanoculleus horonobensis
Pectobacterium sp. A5351		Kovacikia minuta
Natronorubrum sulfidifaciens		Campylobacter sp. CS_NA3

Rathayibacter tritici		Methanocaldococcus fervens
Streptomyces rectiverticillatus		Olleya sp. HaHaR_3_96
Amycolatopsis sp. NBC_01480		Mycobacterium lentiflavum
Streptococcus koreensis		Synechococcus sp. CC9605
Streptomyces calvus		Pseudoalteromonas carrageenovora
Mycolicibacterium chubuense		Pseudomonas sp. BW7P1
Klebsiella electrica		Methanocaldococcus bathoardescens
Enterococcus mundtii		Rhodococcus aetherivorans
Raoultella ornithinolytica		Pseudescherichia vulneris
Mycetocola spongiae		Shewanella spartinae
Arcanobacterium haemolyticum		Gracilibacillus caseinilyticus
Leucobacter rhizosphaerae		Thermanaeromonas toyohensis
Streptococcus gordonii		Halomarina salina
Curtobacterium sp. MCLR17_007		Acetohalobium arabaticum
Streptococcus cristatus		Curtobacterium sp. MCLR17_058
Corynebacterium incognita		Brevundimonas fontaquae
Rhodoluna limnophila		Natrarchaeobius halalkaliphilus
Enterobacter sp. SES19		Flaviflexus ciconiae
Mycolicibacterium goodii		Mycolicibacterium rhodesiae
Nocardia sp. NBC_01730		Corynebacterium sp. FDAARGOS 1242
Streptomyces hygroscopicus		Glaciimonas sp. PAMC28666
Massilia sp. YMA4		Undibacterium sp. 20NA77.5
Schaalia cardiffensis		Marinobacter shengliensis
Gulosibacter sediminis		Nostoc sp. C057
Klebsiella huaxiensis		Mesorhizobium sp. WSM4906
Arthrobacter sp. PAMC25284		Ancylobacter novellus
Arthrobacter sp. ATA002		Candidatus Purcelliella pentastirinorum
Botrytis cinerea		Aspergillus uvarum
Enterobacter ludwigii		Streptomyces sp. NBC_00523
Saccharomonospora viridis		Paenibacillus sp. FSL R7-0331
Arthrobacter sp. KBS0702		Micromonospora sp. WMMA1976
Arthrobacter sp. zg-Y20		Caballeronia sp. Sq4a
Streptomyces sp. Tue 6075		Caldisphaera sp.
Sulfitobacter sp. S190		Methanoculleus sp. CWC-02
Corynebacterium pseudokroppenstedtii		Penicillium mononematosum
Cronobacter sakazakii		Exiguobacterium sp. 9-2
Citricoccus sp. I39-566		Acinetobacter bereziniae
Klebsiella sp. WP3-S18-ESBL-05		Corynebacterium simulans
Pseudonocardia dioxanivorans		Acidothermus cellulolyticus
Pasteurella multocida		Brevundimonas sp. NCCP 15609
Streptomyces sp. HUAS 14-6		Kribbella sp. CA-293567

Pseudarthrobacter sp. NBSH8		Halapricum salinum
Jiangella alkaliphila		Halorubrum lipolyticum
Klebsiella oxytoca		Geobacter sp. DSM 9736
Streptococcus anginosus		Halosimplex rubrum
Streptomyces broussonetiae		Colletotrichum tamarilloi
Candidatus Aquiluna sp. UB-MaderosW2red		Gracilinema caldarium
Shimwellia blattae		Candidatus Blochmannia ocreatus
Nocardioides scoriae		Vibrio toranzoniae
Streptosporangium sp. NBC_01495		Pseudodesulfovibrio nedwellii
Protaetiibacter larvae		Campylobacter volucris
Nocardioides sp. NBC_00368		Tupanvirus deep ocean
Bordetella trematum		Phaeobacter porticola
Glutamicibacter mishrai		Pseudomonas sp. B11D7D
Mixta hanseatica		Streptomyces sp. FR-008
Gleimia europaea		Cercospora kikuchii
Enterococcus faecium		Haladaptatus cibarius
Schaalia sp. ZJ405		uncultured Methanocorpusculum sp.
Arthrobacter sp. PM3		Aneurinibacillus uraniidurans
Escherichia coli		Streptomyces sp. HU2014
Mycobacterium stomatepiae		Sphingobium sp. YC-XJ3
Arthrobacter sp. FB24		Prochlorococcus sp. MIT 0604
Salipiger profundus		Serinicoccus profundi
Tessaracoccus sp. MC1865		Streptomyces longhuiensis
Neisseria yangbaofengii		Cohnella sp. LGH
Streptomyces sp. NBC_00370		Zhouia spongiae
Lysinibacter sp. HNR		Pseudomonas sp. Pc102
Streptomyces sp. RTd22		Tolumonas auensis
Alcaligenes aquatilis		Thermococcus sp. MAR1
Corynebacterium maris		Pseudomonas kribbensis
Leclercia sp. LSNIH3		Halomonas halophila
Paludibaculum fermentans		Peptoniphilus ivorii
Aquitalea aquatilis		Bacillus velezensis
Arthrobacter sp. ERGS1:01		Nostoc sp. 'Lobaria pulmonaria (5183) cyanobiont'
Cryobacterium sp. SO1		Pseudomonas inefficax
Propioniciclava sp. MC1595		Streptomyces sp. HP-A2021
Streptomyces sp. DSM 40750		Natronorubrum tibetense
Streptomyces ficellus		Paenibacillus azoreducens
Citricoccus sp. NR2		Comamonas koreensis
Janibacter terrae		Sediminibacillus dalangtanensis
Neisseria flavescens		Thiospirochaeta perfilievii
Natronobiforma cellulositropha		Halalkalicoccus tibetensis

Catenovulum sp. TS8		Acinetobacter sp. LoGeW2-3
Cellulomonas palmilytica		Cellvibrio sp. KY-GH-1
Haemophilus parahaemolyticus		Vibrio sp. SCSIO 43137
Arthrobacter alpinus		Natronobiforma cellulositropha
Enterococcus faecalis		Halorhodospira halophila
Flaviflexus ciconiae		Cetobacterium somerae
Miltoncostaea marina		Pantoea sp. SOD02
Neisseria sp. Marseille-Q6792		Bdellovibrio sp. KM01
Streptomyces tubbatahanensis		Pseudoxanthomonas sp. JBR18
Nocardiopsis alba		Halogeometricum sp. S1BR25-6
Glutamicibacter nicotianae		Streptomyces sp. TN58
Neisseria lactamica		Rhodoluna limnophila
Enterobacter sp. JBIWA003		Actinobacillus indolicus
Neisseria subflava		Bartonella quintana
Mycolicibacterium celeriflavum		Mesomycoplasma bovoculi
Luteimonas sp. YGD11-2		Vibrio sp. VB16
Mycobacterium simiae		Fructilactobacillus sanfranciscensis
Agrococcus sp. Marseille-Q4369		Pseudomonas rhodesiae
Enterobacter sp. Crenshaw		Paenibacillus sp. FSL R7-0273
Cellulomonas taurus		Actinoplanes sp. OR16
Pseudomonas trivialis		Marivirga sp. ABR2-2
Cronobacter malonaticus		Orientia tsutsugamushi
Rhodococcus globerulus		Selenomonas sp. oral taxon 920
Klebsiella pasteurii		Dietzia cinnamea
Curtobacterium sp. MR_MD2014		Haloprofundus halobius
Lelliottia sp. WB101		Ilyobacter polytropus
Pseudarthrobacter defluvii		Paenibacillus amylolyticus
Brachybacterium ginsengisoli		Corynebacterium sanguinis
Streptomyces sp. NBC_01717		Corynebacterium genitalium
Enterobacter cloacae complex sp. R_G8		Methylacidimicrobium sp. AP8
Kitasatospora fiedleri		Vibrio cholerae
Kocuria rosea		Atopobium sp. oral taxon 416
Actinoplanes oblitus		Listeria welshimeri
Nakaseomyces glabratus		Pseudomonas sp. SC3(2021)
Curtobacterium sp. TC1		Halorubrum depositum
Glutamicibacter protophormiae		Modestobacter sp. L9-4
Glaciihabitans sp. INWT7		Paenibacillus pabuli
Streptococcus phage SpSL1		Corynebacterium mycetoides
Mycolicibacterium sp. TUM20985		Nostoc piscinale
Malassezia restricta		Pseudomonas hamedanensis
Nocardia sp. NBC_01327		Moraxella canis

Penicillium cosmopolitanum		Nanchangia anserum
Micrococcus terreus		Candidatus Arsenophonus lipoptenae
Glutamicibacter creatinolyticus		Capnocytophaga ochracea
Arthrobacter sp. NicSoilB8		Candidatus Schneideria nysicola
Micromonospora purpureochromogenes		Horticoccus luteus
Micromonospora auratinigra		Alicyclobacillus acidoterrestris
Acinetobacter oleivorans		Penicillium lagena
Streptomyces sp. f51		Alkalihalobacillus sp. AL-G
Kocuria varians		Halomicroarcula rubra
Rothia kristinae		Aquisalinus flavus
Arcanobacterium pinnipediorum		Gordonia sp. KTR9
Klebsiella michiganensis		Acinetobacter sp. ACNIH2
Malassezia globosa		Methanolobus mangrovi
Mycolicibacterium gadium		Metamycoplasma hominis
Streptomyces lavendulae		Radiobacillus deserti
Rathayibacter sp. VKM Ac-2805		Microbacterium neungamense
Micropruina glycogenica		Shewanella halifaxensis
Brevibacterium limosum		Halobacillus salinarum
Paeniglutamicibacter sp. Y32M11		Aspergillus niger
Corynebacterium frankenforstense		Natronorubrum sulfidifaciens
Saccharomonospora glauca		Enterococcus sp. 12C11_DIV0727
Kocuria rhizophila		Sulfitobacter sp. DSM 110093
Ligilactobacillus agilis		Tumebacillus avium
Protaetiibacter intestinalis		Polynucleobacter antarcticus
Streptomyces sp. 891-h		Halanaeroarchaeum sp. HSR-CO
Streptococcus lactarius		Lacinutrix neustonica
Raoultella planticola		Chamaesiphon minutus
Brevibacterium luteolum		Neobacillus sp. PS3-40
Kocuria turfanensis		Polaribacter huanghezhanensis
Rathayibacter toxicus		Oceanobacillus sp. FSL K6-0127
Neisseria cinerea		Leptospira mayottensis
Brachybacterium huguangmaarense		Pseudomonas sp. ADAK18
Erwinia sp. QL-Z3		Altererythrobacter sp. BO-6
Brachybacterium sp. NBEC-018		Alcanivorax sp.
Streptomyces pratensis		Polynucleobacter sp. MWH-P3-07-1
Aeromicrobium wangtongii		Methylovorus glucosotrophus
Arthrobacter crystallopoietes		Mycolicibacterium aurum
Klebsiella quasipneumoniae		Photobacterium swingsii
Arthrobacter sp. Helios		Vagococcus luciliae
Ruania suaedae		Actinobacillus suis
Haemophilus pittmaniae		Desulfitobacterium dichloroeliminans

Lactobacillus crispatus		Spiroplasma citri
Enterobacter sp. Colony194		Aspergillus wentii
Streptococcus sp. 1643		Halolamina sediminis
Aggregatibacter sp. oral taxon 513		Aspergillus bombycis
Rothia aeria		Pseudomonas sp. MUP55
Arthrobacter sp. PAMC25564		Altererythrobacter sp. TH136
Basilea psittacipulmonis		Sporosarcina pasteurii
Lactobacillus paragasseri		Yersinia intermedia
Paenarthrobacter ureafaciens		Synechococcus sp. WH 8101
Actinomyces pacaensis		Citrobacter sp. RHBSTW-00887
Serinicoccus profundi		Pseudomonas sp. TUM22785
Caldicellulosiruptor hydrothermalis		Mycoplasma tullyi
Arthrobacter sulfonylureivorans		Arcobacter roscoffensis
Kocuria flava		Candidatus Mycobacterium wuenschmannii
Kingella denitrificans		Mycobacterium shigaense
Streptococcus pneumoniae		Zymoseptoria tritici
Serratia ureilytica		Aliivibrio salmonicida
Rothia amarae		Nitrosopumilus adriaticus
Arthrobacter sp. CDRTa11		Pseudomonas sp. AO-1
Serinicoccus marinus		Roseomonas mucosa
Streptococcus pseudopneumoniae		Microbulbifer sp. MLAF003
Streptococcus sp. D7B5		Pleurocapsa sp. PCC 7327
Enterobacter sp. AN-K1		Microbacterium sp. str. 'China'
Scandinavium goeteborgense		Kocuria sp. BT304
Klebsiella quasivariicola		Shewanella bicestrii
Aurantimicrobium photophilum		Moraxella lincolnii
Kosakonia cowanii		Staphylococcus hyicus
Streptococcus phage PH10		Alternaria conjuncta
Enterobacter sp. N18-03635		Halorubrum laminariae
Streptococcus sp. SP4011		Corynebacterium sp. Z-1
Streptococcus sp. NPS 308		Pseudochrobactrum sp. Wa41.01b-1
Aggregatibacter sp. 2125159857		Thiothrix litoralis
Rothia dentocariosa		Qipengyuania aquimaris
Klebsiella sp. A52		Asticcacaulis sp. DW145
Stenotrophomonas sp. ZAC14D2_NAIMI4_6		Fusobacterium sp. oral taxon 203
Streptococcus sp. 116-D4		Methylomonas sp. DH-1
Streptococcus oralis		Methylocystis heyeri
Pseudomonas sessilinigenes		Candidatus Nitrosopelagicus brevis
Enterobacter sp. DSM 30060		Blastomyces dermatitidis
Haemophilus sp. oral taxon 036		Limnohabitans sp. TEGF004
Streptococcus sp. FSL W8-0197		Methanoregula formicica

Streptococcus phage PH15		Nitrosopumilus oxyclinae
Neisseria elongata		Haloarcula terrestris
Rothia mucilaginosa		Pelistega ratti
Streptococcus sp. oral taxon 064		Cyberlindnera jadinii
Streptococcus mitis		Stenotrophomonas sp. BIO128-Bstrain
Enterobacter sp. JJBC		Desulfopila sp. IMCC35008
Leclercia sp. W6		Pantoea sp. X85
Streptococcus toyakuensis		Streptomyces alboniger
Klebsiella africana		Weeksella virosa
Citrobacter koseri		Pseudomonas sp. CCC3.1
Klebsiella pneumoniae		Paenibacillus baekrokdamisoli
Streptococcus sp. oral taxon 431		Curtobacterium sp. MCJR17_043
Neisseria sp. oral taxon 014		Mycoplasma crocodyli
Limosilactobacillus oris		Aliarcobacter thereius
Stenotrophomonas pavanii		Ferrimonas sp. YFM
Streptococcus sp. DTU_2020_1001019_1_SI_AUS_MUR_006		Borrelia sp. HM
Streptococcus gwangjuense		Mycoplasma cottewii
Aggregatibacter segnis		Moraxella catarrhalis
Stenotrophomonas sp. ESTM1D_MKCIP4_1		Novibacillus thermophilus
Haemophilus parainfluenzae		Vibrio cyclitrophicus
Streptococcus sp. Marseille-Q3533		Aquella oligotrophica
Streptococcus sp. oral taxon 061		Paenibacillus chitinolyticus
Limosilactobacillus mucosae		Candidatus Aciduliprofundum boonei
Aggregatibacter actinomycetemcomitans		Acidilobus sp. 7A
Streptococcus infantis		Fischerella sp. NIES-3754
Streptococcus sp. Marseille-Q6470		Thiomicrorhabdus sediminis
Haemophilus influenzae		Penicillium vulpinum
Stenotrophomonas sp. Pemsol		Ferrimonas sp. SCSIO 43195
Phytobacter sp. SCO41		Streptomyces sp. MBT27
Aggregatibacter aphrophilus		Candidatus Kinetoplastibacterium galatii
Lactobacillus delbrueckii		Salinicoccus halodurans
Klebsiella aerogenes		Thermothielavioides terrestris
		Alcaligenes faecalis
		Mycobacterium vicinigordonae
		Rathayibacter sp. VKM Ac-2805
		Geomonas oryzisoli
		Symbiopectobacterium purcellii
		Campylobacter hominis
		Devosia sp. RR2S18
		Pantoea sp. Z09
		Nitrosopumilus ureiphilus

		Gordonia hongkongensis
		Sphingopyxis sp. TUF1
		Kosakonia arachidis
		Sphingopyxis sp. GC21
		Bacillus smithii
		Shewanella marisflavi
		Brucella pseudintermedia
		Pectobacterium polaris
		Methanocorpusculum labreanum
		Methanimicrococcus stummii
		Aminobacterium sp. MB27-C1
		Chlorobaculum limnaeum
		Paenibacillus sp. FSL k6-2145
		Pseudomonas sp. C27(2019)
		Vibrio sp. B1FLJ16
		Brevibacillus ruminantium
		Acinetobacter baylyi
		Candidatus Nitrosotalea okcheonensis
		Bradyrhizobium sp. CW1
		Nocardioides ungokensis
		Penicillium solitum
		Anabaena sp. 90
		Oligella urethralis
		Pectobacterium versatile
		Stenotrophomonas sp. Pemsol
		Salmonella bongori
		Bacillus sp. FSL K6-0972
		Streptomyces sp. WAC00288
		Candidatus Nasuia deltocephalinicola
		Ketogulonicigenium robustum
		Flavobacterium kingsejongi
		Vibrio bathopelagicus
		Syntrophobacter fumaroxidans
		Enterococcus sp. ZQ21
		Pseudomonas sp. P5_109
		Aeribacillus sp. FSL W8-0870
		Psychrobacter sp. LV10R520-6
		Rheinheimera sp. MMS21-TC3
		Streptomyces erythrochromogenes
		Planococcus maritimus
		Pseudomonas sp. 15A4

		Polynucleobacter sp. JS-JIR-II-50
		Enterobacter mori
		Leuconostoc mesenteroides
		Marinithermus hydrothermalis
		Dictyoglomus turgidum
		Methanoculleus sp. YWC-01
		Oceanisphaera profunda
		Acinetobacter sp. AHP123
		Saccharomycodes ludwigii
		Sporomusa sphaeroides
		Mycobacterium ulcerans
		Mycoplasma sp. NEAQ87857
		Limnobacter thiooxidans
		Candidatus Gullanella endobia
		Rathayibacter rathayi
		Macrococcus bovicus
		Polynucleobacter sp. AP-Nino-20-G2
		Methanocaldococcus sp.
		Natronorubrum halophilum
		Streptomyces sp. GS7
		Paenibacillus sp. MMS20-IR301
		Halopseudomonas sp. SMJS2
		Microbacterium sp. F6_8S_P_2B
		Methylacidiphilum kamchatkense
		Cutibacterium equinum
		Sphingobium sp. WTD-1
		Streptomyces sp. NBC_01267
		Candidatus Pantoea carbekii
		Acinetobacter colistiniresistens
		Schaalia sp. ZJ405
		Legionella waltersii
		Paenibacillus lautus
		Hymenobacter baengnokdamensis
		Streptomyces griseus
		Bernardetia sp. Wsw4-3y2
		Pandoravirus inopinatum
		Nocardioides sp. JQ2195
		Staphylococcus sp. 11-B-312
		uncultured Desulfobulbus sp.
		Streptomyces sp. S399
		Natronococcus amylolyticus

		Aeromonas rivuli
		Legionella geestiana
		Thermobaculum terrenum
		Donellivirus gee
		Serratia inhibens
		Georgenia faecalis
		Pseudomonas sp. G166
		Cohnella herbarum
		Legionella micdadei
		Natronolimnohabitans sp. A-GB9
		Chlorobium phaeobacteroides
		Candidatus Pelagibacter giovannonii
		Methanobrevibacter olleyae
		Gilliamella sp. ESL0441
		Eremothecium sinecaudum
		Mycobacterium sp. JS623
		Campylobacter ureolyticus
		Staphylococcus sp. 17KM0847
		Acidianus sp. HS-5
		Paenibacillus alvei
		Bacillus safensis
		Raoultella planticola
		Burkholderia aenigmatica
		Halonotius roseus
		Superficieibacter sp. HKU1
		Bacillus badius
		Paecilomyces variotii
		Pseudomonas sp. 02C 26
		Malassezia pachydermatis
		Candidatus Ichthyocystis hellenicum
		Geoglobus ahangari
		Streptococcus phage SpSL1
		Moritella yayanosii
		Dickeya zeae
		Cyclobacterium amurskyense
		Methanoculleus sp. FWC-SCC3
		Janthinobacterium sp. 17J80-10
		Methanomethylophilus alvi
		Bacillus sp. DX3.1
		Xenorhabdus hominickii
		Thermococcus peptonophilus

		Gemella sp. oral taxon 928
		Leptospira weilii
		Sporomusa termitida
		Paenibacillus sp. FSL K6-0276
		Pseudodesulfovibrio piezophilus
		Paracoccus albus
		Campylobacter avium
		Nitrososphaera viennensis
		Methylomonas paludis
		Candidatus Mycobacterium methanotrophicum
		Erysipelothrix sp. HDW6C
		Fulvitalea axinellae
		Moorena producens
		Halococcus dombrowskii
		Candidatus Mycoplasma mahonii
		Sphingobacterium sp. PCS056
		Micromonospora viridifaciens
		Metabacillus sp. B2-18
		Lysobacter sp. S4-A87
		Flavobacterium sp. M31R6
		Mycobacteroides immunogenum
		Brevibacterium limosum
		Thermosipho africanus
		Anabaena sp. WA102
		Bordetella genomosp. 6
		Selenomonas sp. oral taxon 136
		Colletotrichum fioriniae
		Nonlabens sp. MB-3u-79
		Arthrobacter sp. StoSoilB13
		Sulfitobacter sp. W002
		Mycoplasma sp. OR1901
		Pseudomonas sp. TMP9
		Metabacillus sp. cB07
		Acidiferrobacter sp. SPIII_3
		Streptomyces sp. NBC_00569
		Streptomyces malaysiensis
		Pyrofollis japonicus
		Cellulomonas wangleii
		Streptomyces pseudovenezuelae
		Lysobacter ciconiae
		Kordiimonas sp. SCSIO 12610

		Pseudomonas sp. GM17
		Methanotorris formicicus
		Kwoniella dejecticola
		Lactobacillus sp. ESL0684
		Halococcus hamelinensis
		Candidatus Minimicrobia vallesae
		Pseudomonas atacamensis
		Candidatus Thioglobus sp. NP1
		Bacterioplanes sanyensis
		Oceanobacillus sp. FSL W7-1281
		Moumouvirus moumou
		Paenibacillus sp. YPG26
		cyanobacterium endosymbiont of Epithemia clementina EcSB
		Candidatus Kinetoplastibacterium sorsogonicusi
		Arcanobacterium canis
		Colwellia sp. Arc7-D
		Pseudomonas sp. DR48
		Nitrososphaera sp. AFS
		Aspergillus piperis
		Buttiauxella ferragutiae
		Vibrio azureus
		Glaesserella parasuis
		Shewanella sp. MTB7
		Janthinobacterium sp. PAMC25594
		Metabacillus sp. FJAT-53654
		Thiothrix subterranea
		Polaribacter sp. SA4-10
		Psychrobacter urativorans
		Bacillus amyloliquefaciens
		Paenibacillus xylanilyticus
		Beijerinckia indica
		Sneathia vaginalis
		Candidatus Ishikawella capsulata
		Campylobacter curvus
		Spartinivicinus ruber
		Candidatus Babela massiliensis
		Virgibacillus sp. NKC19-16
		Streptomyces sp. NBC_01358
		Kitasatospora aureofaciens
		Mycoplasma sp. Pen4
		Streptomyces sp. NBC_00273

		Nitrosopumilus sp. b1
		Corynebacterium pseudogenitalium
		Glaciihabitans sp. INWT7
		Vibrio astriarenae
		Sediminispirochaeta smaragdinae
		Vibrio tarriae
		Lactiplantibacillus pentosus
		Halobacterium wangiae
		Bradyrhizobium sp. TM102
		Ketobacter alkanivorans
		Mergibacter septicus
		Treponema sp. OMZ 788
		Mesoplasma melaleucae
		Caloramator sp. mosi_1
		Erysipelothrix piscisicarius
		Psychrobacillus sp. AK 1817
		Planococcus sp. 107-1
		Citrobacter sp. RHBSTW-00903
		Streptomyces sp. NBC_00236
		Biavirus raunefjordenense
		Geobacillus sp. C56-T3
		Salipaludibacillus sp. LMS25
		Thiothrix lacustris
		Synechocystis sp. PCC 6714
		Salinibaculum sp. YCN56
		Legionella israelensis
		Rheinheimera mangrovi
		Bacillus tequilensis
		Enterobacter cloacae complex sp. ECL352
		Acidimicrobium ferrooxidans
		Arsenophonus apicola
		Gordonia sp. PP30
		Paenibacillus uliginis
		Synechococcus sp. JA-2-3B'a(2-13)
		Pseudarthrobacter sp. NIBRBAC000502772
		Williamsoniiplasma luminosum
		Streptomyces sp. NBC_01336
		Cryptococcus amylolentus
		Cronobacter muytjensii
		Phormidium yuhuli
		Agrobacterium fabrum

		Campylobacter sp. 2014D-0216
		Bacillus sp. Cs-700
		Lactococcus sp. LG606
		Candida parapsilosis
		Cyanobacterium sp. Dongsha4
		Bifidobacterium sp. ESL0704
		Acinetobacter sp. TGL-Y2
		Acinetobacter sp. 10FS3-1
		Peribacillus psychrosaccharolyticus
		Brachybacterium sp. P6-10-X1
		Sulfurisphaera ohwakuensis
		Solibacillus sp. FSL R5-0691
		Candidatus Methylopumilus turicensis
		Priceomyces carsonii
		Paenibacillus sp. FSL R5-0470
		Citrobacter sedlakii
		Rickettsiales endosymbiont of Stachyamoeba lipophora
		Acinetobacter pseudolwoffii
		Schizosaccharomyces japonicus
		Methanosarcina vacuolata
		Cohaesibacter marisflavi
		Paenibacillus sp. CC-CFT742
		Clostridium sp. 16K-1-R1
		Xenorhabdus griffiniae
		Enterobacter sp. N18-03635
		Gilliamella sp. wkB7
		Carboxydothermus hydrogenoformans
		Spiroplasma sp. SV19
		Brevibacillus sp. FSL K6-6036
		Citrobacter sp. LUTT5
		Desulfonema magnum
		Halorubrum rutilum
		Pantoea sp. Nvir
		Nostoc sp. MS1
		Streptomyces sp. NBC_00444
		Aeromonas popoffii
		Algibacter sp. L3A6
		Sulfolobus tengchongensis
		Bacillus pacificus
		Psychrobacillus sp. INOP01
		Pseudomonas sp. RtIB026

		Candidatus Nanopusillus massiliensis
		Streptococcus viridans
		Pediococcus claussenii
		Pseudomonas asplenii
		Moraxella bovoculi
		Aquimarina sp. BL5
		Zygosaccharomyces rouxii
		Enterobacter sp. SGAir0187
		Pseudomonas sp. P9_31
		Thermotoga profunda
		Shimwellia blattae
		Enterobacter sp. S-33
		Clostridium sp. AWRP
		Polynucleobacter sp. MWH-UH35A
		Brochothrix thermosphacta
		Pneumocystis murina
		Klebsiella sp. WP8-S18-ESBL-06
		Pantoea sp. CCBC3-3-1
		Methylophaga marina
		Paracoccus tegillarcae
		Caldisphaera lagunensis
		Citrobacter freundii complex sp. CFNIH2
		Peribacillus sp. R9-11
		Bartonella sp. TP
		Streptomyces cinereoruber
		Aliarcobacter trophiarum
		Acinetobacter towneri
		Citrobacter sp. RHBSTW-00524
		Nostoc sp. PCC 7107
		Nostoc sp. NIES-3756
		Podospora pseudocomata
		Metarhizium robertsii
		Rhodococcus sp. 21391
		Bifidobacterium sp. ESL0690
		Citrobacter sp. BDA59-3
		Psychrobacter cryohalolentis
		Thiomicrorhabdus indica
		Pseudoalteromonas mariniglutinosa
		Spiroplasma helicoides
		Deinococcus puniceus
		Flammeovirga sp. MY04

		Athalassotoga saccharophila
		Halorubrum sp. BV1
		Citrobacter sp. RHB35-C21
		Candidatus Liberibacter americanus
		Simonsiella muelleri
		Penicillium roqueforti
		Chitinibacter bivalviorum
		Aliarcobacter lanthieri
		Lactococcus sp. bn62
		Candidatus Nitrosocosmicus oleophilus
		Deinococcus sp. AJ005
		Neisseria flavescens
		Simiduia agarivorans
		Methanolobus halotolerans
		Klebsiella sp. WP3-S18-ESBL-05
		secondary endosymbiont of Ctenarytaina eucalypti
		Vagococcus sp. B2T-5
		Pectobacterium wasabiae
		Hydrogenibacillus sp. N12
		Clostridium autoethanogenum
		Halorubrum sp. CBA1229
		Candidatus Nitrosocosmicus sp. SS
		Encephalitozoon cuniculi
		Citrobacter sp. Colony322
		Bifidobacterium eulemuris
		Weissella ceti
		Methanosarcina sp. 1.H.T.1A.1
		Citrobacter sp. RHBSTW-01013
		Enterobacter sp. R4-368
		Citrobacter sp. B72
		Methanobrevibacter boviskoreani
		Arcobacter cloacae
		Thiomicrospira sp. S5
		Amphibacillus xylanus
		Sphingopyxis sp. PAMC25046
		Mycoavidus sp. B2-EB
		Thermodesulfobium acidiphilum
		Methanospirillum lacunae
		Gimesia fumaroli
		Leclercia sp. J807
		Laceyella sacchari

		Thiomicrospira sp. R3
		Corynebacterium kutscheri
		Acetobacter tropicalis
		Pseudoalteromonas issachenkonii
		Actinobacillus pleuropneumoniae
		Escherichia coli
		Campylobacter peloridis
		Marivirga sp. BKB1-2
		Proteus penneri
		Methylomicrobium album
		Halobiforma haloterrestris
		Janibacter cremeus
		Streptomyces antimycoticus
		Streptomyces sp. NBC_00869
		Microbacterium sp. EF45047
		Chryseobacterium sp. CP-77
		Citrobacter koseri
		Borreliella burgdorferi
		Acidithiobacillus thiooxidans
		Candidatus Fervidibacter sacchari
		Alicyclobacillus sp. ALC3
		Legionella lansingensis
		Klebsiella sp. WP3-W18-ESBL-02
		Bacillus sp. JAS24-2
		Moritella sp. 24
		Alternaria incomplexa
		Staphylothermus marinus
		Pseudanabaena sp. PCC 7367
		Methanogenium organophilum
		Fluviibacter phosphoraccumulans
		Blochmannia endosymbiont of Camponotus (Colobopsis) obliquus
		Arcobacter peruensis
		Companilactobacillus futsaii
		Mycolicibacterium anyangense
		Aspergillus aculeatus
		Halovenus salina
		Neisseria subflava
		Polynucleobacter sp. MG-6-Vaara-E2
		Aciduliprofundum sp. MAR08-339
		Streptococcus phage PH10
		Levilactobacillus suantsaii

		Litorilituus sediminis
		Enterobacter cloacae complex sp.
		Lactobacillus helsingborgensis
		Salicibibacter cibarius
		Vittaforma corneae
		Candidatus Profftia lariciata
		Citrobacter sp. RHB36-C18
		Paenibacillus sp. FSL H8-0079
		Thalassotalea sp. LPB0316
		Curtobacterium sp. MCBA15_012
		Paenibacillus sp. FSL E2-0190
		Campylobacter sp. 19-13652
		Candidatus Endolissoclinum faulkneri
		Haloferax elongans
		Agarilytica rhodophyticola
		Cruoricaptor ignavus
		Polynucleobacter sp. UB-Siik-W21
		Mycoplasma sp. Mirounga ES2805-ORL
		Hydrogenovibrio crunogenus
		Citrobacter sp. RHBSTW-00986
		Vairimorpha ceranae
		Methanococcoides sp. NM1
		Enterobacteriaceae endosymbiont of Plateumaris braccata
		Sphingobacterium sp.
		Proteus appendicitidis
		Bifidobacterium sp. ESL0732
		Raoultella sp. HC6
		Sphingobacterium faecium
		Flavobacterium nitrogenifigens
		Neokomagataea tanensis
		Methanosarcina sp. MSH10X1
		Methanocaldococcus villosus
		Citrobacter sp. RHBSTW-00570
		Polynucleobacter sp. VK25
		Celerinatantimonas diazotrophica
		Methanocalculus taiwanensis
		endosymbiont DhMRE of Dentiscutata heterogama
		Candidatus Nitrosopumilus sp. SW
		Pectobacterium sp. MAFF 302110
		Tessaracoccus sp. T21
		Lactobacillus sp. PV037

		Microbacterium sp. PAMC21962
		Klebsiella sp. WP4-W18-ESBL-05
		Halosimplex aquaticum
		Gilliamella sp. B3022
		Secundilactobacillus paracollinoides
		Cylindrospermopsis raciborskii
		Acinetobacter septicus
		Acetobacter senegalensis
		Alkalimarinus coralli
		Suillus bovinus
		Atlantibacter subterranea
		Shewanella sp. FJAT-52076
		Rickettsia australis
		Candidatus Blochmannia vicinus
		Caldicellulosiruptor saccharolyticus
		Neptunomonas phycophila
		Streptomyces sp. So13.3
		Mycobacterium simiae
		Rhizobium sp. NLR16a
		Pseudomonas oleovorans
		Vibrio sp. SCSIO 43132
		Francisella orientalis
		Lactobacillus acetotolerans
		Candidatus Moranella endobia
		Spiroplasma clarkii
		Rubinisphaera brasiliensis
		Sulfurospirillum cavolei
		Mycoplasma capricolum
		Pseudoalteromonas sp. 1_2015MBL_MicDiv
		Methanobacterium sp. CWC-01
		Arsenophonus nasoniae
		Thermodesulfobacterium geofontis
		Citrobacter enshiensis
		Magnetospira sp. QH-2
		Pseudomonas benzopyrenica
		Streptomyces sp. NBC_00441
		Lactobacillus sp. ESL0677
		Metamycoplasma salivarium
		Streptomyces olivaceus
		Polynucleobacter ibericus
		Marinagarivorans cellulosilyticus

		Ottowia sp. oral taxon 894
		Blattabacterium sp. DPU
		Halobacterium zhouii
		Exiguobacterium profundum
		Pseudomonas sp. B21-053
		Limosilactobacillus portuensis
		Pseudomonas sp. BSw22131
		Bifidobacterium sp. ESL0682
		Capronia coronata
		Salinivibrio proteolyticus
		Borrelia crocidurae
		Acinetobacter sp. ESL0695
		Marinomonas sp. FW-1
		Polynucleobacter sp. MWH-S4W17
		Pseudomonas sp. PP3
		Acinetobacter equi
		Methanosarcina sp. DH2
		Bacillus sp. ZJS3
		Citrobacter sp. Colony219
		Treponema sp. Marseille-Q4132
		Acinetobacter lactucae
		Fructilactobacillus ixorae
		Vitreoscilla massiliensis
		Citrobacter sp. R56
		Shewanella zhangzhouensis
		Borrelia sp. P9F1
		Kribbella sp. NBC_01484
		Klebsiella sp. A52
		Nematocida major
		Anaplasma phagocytophilum
		Candidatus Fonsibacter ubiquis
		Spiroplasma floricola
		Citrobacter sp. FDAARGOS_156
		Methanobacterium aggregans
		Citrobacter cronae
		Halobellus captivus
		Vulcanisaeta sp. JCM 16161
		Spiroplasma platyhelix
		Lactobacillus kullabergensis
		Fructilactobacillus hinvesii
		Pseudomonas sp. CCOS 191

		Borrelia turcica
		Aspergillus eucalypticola
		Entomospira culicis
		Corynebacterium ammoniagenes
		Halobellus sp. LT62
		Haladaptatus pallidirubidus
		Pseudomonas cedrina
		Limosilactobacillus frumenti
		Magnetospirillum sp. XM-1
		Arthrobacter sp. YN
		Lactobacillus taiwanensis
		Nocardiopsis akebiae
		Haloplanus sp. GDY1
		Citrobacter arsenatis
		Enterobacter sp. RHBSTW-00422
		Enterobacteriaceae endosymbiont of Donacia vulgaris
		Lactobacillus gasseri
		Xanthomonas cucurbitae
		Lactobacillus paragasseri

Note: Species adjusted model for age, probiotics and PPI (SBS I vs. Control); Species adjusted model for antibiotics, probiotics, and PPI (SBS II vs. Control); Species adjusted model for age, antibiotics and probiotics (SBS III vs. Control)

Supplementary Table 3. List of differential microbial species between controls and SBS types identified by MaAsLin2, related to Fig. 4b.

SBS I vs. Control	SBS II vs. Control	SBS III vs. Control
Klebsiella.aerogenes	Blautia.wexlerae	Photobacterium.leiognathi
Lactobacillus.delbrueckii	Faecalibacterium.sp..IP.1.18	Neobacillus.sp..Marseille.Q6967
Aggregatibacter.aphrophilus	Faecalibacillus.intestinalis	Lactobacillus.paragasseri
Streptococcus.phage.EJ.1	Dysosmobacter.welbionis	Lactobacillus.gasseri
Streptococcus.phage.PH15	Faecalibacterium.sp..BCRC.81399	Allocoprobacillus.halotolerans
Aggregatibacter.actinomycetemcomitans	Mediterraneibacter.gnavus	Lactobacillus.taiwanensis
Haemophilus.influenzae	Eggerthella.lenta	Clostridium.sp..JS66
Streptococcus.sp..Marseille.Q6470	Ruminococcus.sp..SR1.5	Ruminococcus.bovis
Streptococcus.infantis	Dorea.formicigenerans	Bacillus.atrophaeus
Streptococcus.sp..oral.taxon.061	X.Ruminococcus..torques	Chryseobacterium.arthrosphaerae
Limosilactobacillus.oris	Dorea.longicatena	Legionella.pneumophila

Streptococcus.sp..Marseille.Q3533	Faecalibacterium.prausnitzii	Actinobacillus.equuli
Rothia.sp..SD9660Na	Anaerostipes.hadrus	Clostridium.bornimense
Streptococcus.gwangjuense	Anaerobutyricum.hallii	Clostridium.sp..DL.VIII
Streptococcus.sp..DTU_2020_1001019_1_SI_AUS_MUR_006	Faecalibacterium.sp..I4.3.84	Facklamia.hominis
Streptococcus.sp..oral.taxon.431	Simiaoa.sunii	Clostridium.drakei
Natrialba.swarupiae	Blautia.luti	X.Clostridium..innocuum
Pectobacterium.wasabiae	X.Ruminococcus..lactaris	Parolsenella.massiliensis
Rothia.sp..ZJ932	Faecalibacterium.sp..I2.3.92	Clostridium.gelidum
Arthrobacter.sp..DNA4	Coprococcus.comes	Neobacillus.sp..DY30
Streptococcus.toyakuensis	Anaerotruncus.colihominis	Mannheimia.varigena
Avibacterium.sp..21.595	Roseburia.rectibacter	Marispirochaeta.aestuarii
Streptococcus.mitis	Wujia.chipingensis	Brachyspira.pilosicoli
Rothia.mucilaginosa	Roseburia.intestinalis	Caminicella.sporogenes
Halomonas.sp..XH26	Blautia.sp..SC05B48	Achromobacter.sp..R39
Arthrobacter.sp..zg.Y919	Intestinibacter.bartlettii	Faecalibacterium.sp..IP.1.18
Streptococcus.oralis	Coprococcus.catus	Gordonibacter.pamelaeae
Streptococcus.sp..116.D4	Blautia.hansenii	Pedobacter.mucosus
Pseudomonas.sessilinigenes	Faecalibacterium.sp..IP.3.29	Pseudodesulfovibrio.sediminis
Streptococcus.sp..NPS.308	X.Clostridium..innocuum	Tannockella.kyphosi
Erwinia.sp..QL.Z3	Romboutsia.ilealis	Cytobacillus.gottheilii
Micrococcus.yunnanensis	Hungatella.hathewayi	Eubacterium.callanderi
Lactobacillus.crispatus	Flavonifractor.plautii	Azonexus.fungiphilus
Rothia.terrae	Blautia.obeum	Deinococcus.wulumuqiensis
Gephyromycinifex.aptenodytis	Faecalibacterium.sp..I3.3.89	Neurospora.tetraspora
Arthrobacter.crystallopoietes	Agathobacter.rectalis	Clostridium.sp..C1
Glutamicibacter.creatinolyticus	Sellimonas.intestinalis	Lactobacillus.johnsonii
Miltoncostaea.marina	Lachnospira.eligens	Globicatella.sanguinis
Kocuria.palustris	Blautia.coccoides	Pseudomonas.sp..FP2262
Methanocaldococcus.infernus	Ruminococcus.gauvreauii	Clostridium.acetobutylicum
Leucobacter.rhizosphaerae	Anaerostipes.caccae	Postia.placenta
Microbacterium.elymi	Blautia.parvula	Streptococcus.salivarius
Bradyrhizobium.sp..195	Romboutsia.sp..13368	Paenibacillus.lentus
Microbacterium.sp..ABRD28	Enterocloster.bolteae	Burkholderia.pseudomallei
Microbacterium.caowuchunii	X.Clostridium..scindens	Cellvibrio.sp..PSBB006
Microbacterium.hominis	Blautia.argi	Chryseobacterium.camelliae
Babjeviella.inositovora	Roseburia.hominis	Helcococcus.kunzii

Clostridium.cellulovorans	Intestinimonas.butyriciproducens	Vibrio.ruber
	Ruthenibacterium.lactatiformans	Staphylococcus.rostri
	Eggerthella.guodeyinii	Cellulosilyticum.lentocellum
	Terrisporobacter.glycolicus	Staphylococcus.lugdunensis
	Blautia.producta	Clostridium.butyricum
	Thomasclavelia.ramosa	Sodiomyces.alkalinus
	Qiania.dongpingensis	Cellulosilyticum.sp..WCF.2
	Clostridium.sp..M62.1	Virgibacillus.natechei
	Eubacterium.ventriosum	Paenibacillus.sp..BIHB.4019
	Novisyntrophococcus.fermenticellae	Paenibacillus.tianjinensis
	Turicibacter.bilis	Enterococcus.rotai
	Clostridium.bornimense	Halobacillus.shinanisalinarum
	Enterocloster.asparagiformis	Tepidibacillus.sp..LSZ.M11000
	Allocoprobacillus.halotolerans	Neoarthrinium.moseri
	Coprococcus.sp..ART55.1	Marinifilum.fragile
	Roseburia.sp..499	Mesobacillus.subterraneus
	Blautia.pseudococcoides	Dorea.longicatena
	Cellulosilyticum.lentocellum	Bacillus.sp..KH172YL63
	Clostridium.perfringens	uncultured.Carboxylicivirga.sp.
	Clostridium.sp..SY8519	Aureimonas.altamirensis
	Intestinibacillus.sp..NTUH.41.i26	Lactobacillus.isalae
	Romboutsia.sp..CE17	
	Oscillibacter.hominis	
	Pusillibacter.faecalis	
	Marvinbryantia.formatexigens	
	Blautia.liquoris	
	Clostridium.isatidis	
	Enterocloster.clostridioformis	
	Thomasclavelia.spiroformis	
	Methanosphaera.stadtmanae	
	Clostridium.cadaveris	
	Anaerostipes.rhamnosivorans	
	Bacillus.paralicheniformis	
	Lacrimispora.saccharolytica	
	Terrisporobacter.petrolearius	
	Clostridium.septicum	

	Roseburia.sp..831b	
	Acutalibacter.muris	
	Terrisporobacter.mayombei	
	Blautia.hydrogenotrophica	
	Butyrivibrio.crossotus	
	Clostridium.sp..MD294	
	Eubacterium.sp..MSJ.33	
	Romboutsia.hominis	
	Claveliimonas.bilis	
	Clostridium.sp..BJN0001	
	Lacrimispora.xylanolytica	
	Clostridium.beijerinckii	
	Longicatena.caecimuris	
	Macrococcus.armenti	
	Anaerocolumna.sp..AGMB13025	
	Lactococcus.petauri	
	Eubacterium.callanderi	
	Lacrimispora.sphenoides	
	Butyrivibrio.proteoclasticus	
	Massilistercora.timonensis	
	Wansuia.hejianensis	
	Clostridium.gasigenes	
	X.Clostridium..hylemonae	
	Clostridium.gelidum	
	Anaeromicropila.herbilytica	
	Anaerocolumna.sp..AGMB13020	
	Anaerocolumna.cellulosilytica	
	Clostridium.saccharoperbutylacetonicum	
	Clostridium.tyrobutyricum	
	Anaerocolumna.sedimenticola	
	Bacillus.atrophaeus	
	Coprococcus.eutactus	
	Anaerotignum.sp..MB30.C6	
	Paraclostridium.sordellii	
	Lachnoclostridium.phytofermentans	
	Oceanirhabdus.sp..W0125.5	
	Butyrivibrio.fibrisolvens	
	Chordicoccus.furentiruminis	
	Bacillus.pseudomycoides	
	Anaerocolumna.chitinilytica	

	Bacillus.altitudinis	
	Salicibibacter.halophilus	
	Sporosarcina.sp..Te.1	
	Clostridium.diolis	
	Butyrivibrio.hungatei	
	Paenibacillus.sp..G2S3	
	Petrocella.atlantisensis	
	Clostridium.sp..DL.VIII	
	Tissierella.sp..MB52.C2	
	Clostridium.acetobutylicum	
	Christensenella.minuta	
	Crassaminicella.indica	
	Schnuerera.ultunensis	
	Clostridium.sp..JS66	
	Halocella.sp..SP3.1	
	Paenibacillus.typhae	
	Clostridium.sp..JN.1	
	Paludicola.sp..MB14.C6	
	Clostridium.chauvoei	
	Sedimentibacter.sp..zth1	
	Clostridium.intestinale	
	Caproicibacterium.lactatifermentans	
	Crassaminicella.thermophila	
	Vagococcus.intermedius	
	Geosporobacter.ferrireducens	
	Berryella.intestinalis	
	Clostridium.cellulovorans	
	Clostridium.argentinense	
	Caminicella.sporogenes	
	Solibacillus.sp..R5.41	
	Clostridium.taeniosporum	
	Clostridium.tetani	
	Clostridium.sporogenes	
	Caldicellulosiruptor.acetigenus	
	Clostridium.sp..BNL1100	
	Paenibacillus.woosongensis	
	Clostridium.drakei	
	Paraliobacillus.zengyii	
	Treponema.peruense	
	Pseudanabaena.sp..ABRG5.3	
	Helicovermis.profundi	

	Tepidibacter.hydrothermalis	
	Paenibacillus.sp..FSL.K6.3182	
	uncultured.Caproiciproducens.sp.	
	Fusobacterium.hominis	
	Bacillus.weihaiensis	
	Paenibacillus.lautus	
	Oceanobacillus.zhaokaii	
	Paenibacillus.sp..DCT19	
	Paenibacillus.sp..HWE.109	
	Jeotgalibaca.arthritidis	
	Caldanaerobacter.subterraneus	
	Providencia.rettgeri	
	Staphylococcus.arlettae	
	Clostridium.fungisolvens	
	Paenibacillus.sp..RC67	
	Clostridium.carboxidivorans	
	Bernardetia.litoralis	
	Desulfitobacterium.metallireducens	
	Paenibacillus.lentus	
	Dermabacter.vaginalis	
	Staphylococcus.agnetis	
	Priestia.filamentosa	
	Clostridium.scatologenes	
	Mammaliicoccus.lentus	
	Tannockella.kyphosi	
	Bacillus.sp..Y1	
	Campylobacter.sp..RM6914	
	Brachyspira.pilosicoli	
	Lysinibacillus.sp..SGAir0095	
	Metabacillus.sp..FJAT.52054	
	Solibacillus.sp..FSL.W8.0372	
	Bacillus.sp..1NLA3E	
	Vallitalea.guaymasensis	
	Listeria.ivanovii	
	Chryseobacterium.gleum	
	Fusobacterium.gonidiaformans	
	Streptococcus.sp..29887	
	Cytobacillus.dafuensis	
	Planococcus.sp..N022	
	Clostridium.autoethanogenum	
	Macrococcus.epidermidis	

	Ruminiclostridium.cellulolyticum	
	Brevibacillus.sp..WF146	
	Tepiditoga.spiralis	
	Pontibacillus.sp..HMF3514	
	Endocarpon.pusillum	
	Microbulbifer.sp..MI.G	
	Virgibacillus.necropolis	
	Acaryochloris.marina	
	Sporosarcina.pasteurii	
	Penicillium.robsamsonii	
	Corynebacterium.endometrii	
	Clostridium.estertheticum	
	Niallia.circulans	
	Alkaliphilus.sp..B6464	
	Brevibacterium.luteolum	
	Cellulosilyticum.sp..WCF.2	
	Paenibacillus.sp..IHBB.10380	
	Paenisporosarcina.antarctica	
	Vibrio.sp..DW001	
	Staphylococcus.rostri	
	Paenibacillus.polygoni	
	Bacillus.fonticola	
	Ornithinimicrobium.avium	
	Carnobacterium.sp..17.4	
	Gracilibacillus.salitolerans	
	Alteracholeplasma.palmae	
	Priestia.koreensis	
	Clostridium.sp..OS1.26	
	Tetragenococcus.koreensis	
	Cellvibrio.sp..PSBB006	
	Eggerthella.sp..YY7918	
	Calothrix.sp..336.3	
	Helicobacter.pylori	
	Defluviitoga.tunisiensis	
	Bacillus.pumilus	
	Ornithinibacillus.sp..FSL.M8.0202	
	Evansella.sp..LMS18	
	Leminorella.richardii	
	Halobacillus.shinanisalinarum	
	Lysinibacillus.louembei	
	Oceanobacillus.oncorhynchi	

	Acetivibrio.clariflavus	
	Penicillium.brevicompactum	
	Parageobacillus.caldoxylosilyticus	
	Lysinibacillus.sp..FSL.H8.0500	
	Bacillus.sp..KH172YL63	
	Paenibacillus.sp..FSL.H8.0537	
	Erysipelothrix.sp..HDW6C	
	Clostridium.cochlearium	
	Staphylococcus.cohnii	
	Alkalihalophilus.pseudofirmus	
	Pasteurella.dagmatis	
	Radiobacillus.kanasensis	
	Cytobacillus.kochii	
	Flavobacterium.cerinum	
	Tenacibaculum.jejuense	
	Staphylococcus.debuckii	
	Endozoicomonas.sp..SCSIO.W0465	
	Cylindrospermum.stagnale	
	Corynebacterium.pseudotuberculosis	
	endosymbiont..TC1..of.Trimyema.compressum	
	Flavobacterium.psychrophilum	
	Sulfurimonas.sp.	
	Halarcobacter.ebronensis	
	Wickerhamomyces.anomalus	
	Trichothermofontia.sichuanensis	
	Dyella.thiooxydans	
	Pseudoalteromonas.phenolica	
	Leptospira.santarosai	
	Bacillus.tropicus	
	Bacillus.sp..THAF10	
	Tenacibaculum.ovolyticum	
	Acidovorax.sp..NCPPB.3576	
	Xenorhabdus.doucetiae	
	Vibrio.casei	
	Advenella.alkanexedens	
	Providencia.sneebia	
	Alkalihalobacillus.sp..LMS6	
	Proteiniborus.sp..MB09.C3	
	Staphylococcus.kloosii	
	Labilibaculum.sp.	

	Clostridium.sp..AWRP	
	Acinetobacter.baylyi	
	Lysinibacillus.parviboronicapiens	
	Vibrio.metschnikovii	
	Actinomyces.sp..zg.332	
	Arcanobacterium.phocae	
	Mammaliicoccus.sciuri	
	Psychromonas.sp..CNPT3	
	Geobacillus.thermodenitrificans	
	Macrococcus.brunensis	
	Rhodococcus.sp..WMMA185	
	Staphylococcus.shinii	
	Luteolibacter.sp..SL250	
	Alkalicoccus.halolimnae	
	Wohlfahrtiimonas.chitiniclastica	
	Yersinia.canariae	
	Poseidonibacter.lekithochrous	
	Neobacillus.sp..Marseille.Q6967	
	Ferroplasma.sp.	
	Mongoliitalea.daihaiensis	
	Corynebacterium.silvaticum	
	Legionella.longbeachae	
	Pseudomonas.sp..GCEP.101	
	Corynebacterium.glaucum	
	Enterococcus.faecalis	

Note:Species adjusted model for age, antibiotics, probiotics and PPI (SBS I vs. Control); Species adjusted model for antibiotics, probiotics, and PPI (SBS II vs. Control); Species adjusted model for age, antibiotics and probiotics (SBS III vs. Control)

Supplementary Table 4. Differential abundance analysis using ANCOM-BC2

**Species adjusted model for age and PN duration (SBS I vs. SBS II)**					
taxon	Enriched group	lfc_GroupSBS II	se_GroupSBS II	W_GroupSBS II	q-values
[Clostridium] scindens	SBS I	-2.948692497	0.398320759	-7.402808992	0.01363994
Lactobacillus isalae	SBS II	5.899099954	0.33179387	17.77941213	0.018560573
**Species adjusted model for age (SBS II vs. SBS III)**					

taxon	Enriched group	lfc_GroupSBS III	se_GroupSBS III	W_GroupSBS III	q-values
Pseudomonas khazarica	SBS III	17.35951947	0.320651803	54.138225	0.00686898811754596
Proteus sp. ZN5	SBS III	11.26810289	0.464559259	24.25546941	0.000509295
Terrisporobacter hibernicus	SBS III	10.19224234	0.498883475	20.43010611	0.000340711
Fusobacterium sp. oral taxon 203	SBS III	9.00419082	0.215356745	41.81058191	0.019263896
Paenibacillus sp. G2S3	SBS III	8.340995306	0.228418611	36.51626834	0.0000296969281621462
Clostridium argentinense	SBS III	7.956558095	0.569667798	13.96701398	0.000238274
Geosporobacter ferrireducens	SBS III	7.344559425	0.373105001	19.68496645	0.002153432
Paenibacillus sp. DCT19	SBS III	6.763890862	0.300409974	22.51553358	0.004954043
Candidatus Arthromitus sp. SFB-rat-Yit	SBS III	6.62115874	0.274225806	24.14491489	3.27E-03
Chryseobacterium nakagawai	SBS III	6.342327692	0.289029933	21.94349778	0.005769493
Lysinibacillus sp. SGAir0095	SBS III	6.253785511	0.453859824	13.77911235	0.007320748
Mediterraneibacter gnavus	SBS III	6.234386789	0.881030395	7.07624484	0.005474174
Blautia wexlerae	SBS III	5.907282573	0.502324183	11.75990083	3.27105E-07
Alkaliphilus sp. B6464	SBS III	5.898277746	0.345290162	17.08209034	9.86125E-05
Flavonifractor plautii	SBS III	5.645134608	0.796765027	7.085068262	0.013011957
[Ruminococcus] torques	SBS III	5.610329445	0.636517905	8.814095249	1.66E-04
Dorea longicatena	SBS III	5.438033472	0.646793146	8.407685684	2.53E-04
Blautia argi	SBS III	5.434641144	0.691305024	7.861422898	3.10E-03
[Clostridium] scindens	SBS III	5.415137633	0.521257849	10.38859682	9.79398E-06
Ruminococcus sp. SR1/5	SBS III	5.317248599	0.502023454	10.59163383	0.000451031

Blautia obeum	SBS III	5.282162654	0.582201738	9.072735971	6.80359E-05
Blautia luti	SBS III	5.223013644	0.444679377	11.74557202	7.05E-06
Enterocloster bolteae	SBS III	5.081836041	0.777163474	6.53895379	0.011168108
Wujia chipingensis	SBS III	5.074190081	0.570451797	8.895037416	0.023145574
Dorea formicigenerans	SBS III	4.925663179	0.610928621	8.062583761	0.00216505
Blautia producta	SBS III	4.897534272	0.58731895	8.338798321	0.003181537
Leminorella richardii	SBS III	4.891001184	0.252172867	19.39543001	0.002384344
Anaerobutyricum hallii	SBS III	4.876190629	0.543264484	8.975721353	0.000186405
Coprococcus comes	SBS III	4.849439876	0.557771288	8.69431607	0.000470965
Blautia liquoris	SBS III	4.629446899	0.43444664	10.65596203	0.001772694
Marvinbryantia formatexigens	SBS III	4.584509415	0.492144901	9.315365058	0.001244443
Blautia hydrogenotrophica	SBS III	4.524035337	0.497953718	9.085252646	0.001716785
[Ruminococcus] lactaris	SBS III	4.515484408	0.519663127	8.689253052	0.003027196
Anaerostipes hadrus	SBS III	4.511408753	0.621113274	7.26342351	0.003686616
Methylovulum psychrotolerans	SBS III	4.481695359	0.231720864	19.34092291	0.002430754
Blautia sp. SC05B48	SBS III	4.45224663	0.513515878	8.67012457	0.000491501
Roseburia hominis	SBS III	4.264684297	0.514000163	8.297048527	0.005403631
Ruthenibacterium lactatiformans	SBS III	4.232933955	0.53931111	7.84877944	0.006994809
Blautia pseudococcoides	SBS III	4.160401957	0.536370465	7.75658286	0.019288403
Lachnospira eligens	SBS III	4.156169148	0.485480593	8.560937768	1.42E-03
Eubacterium ventriosum	SBS III	4.064371753	0.424531461	9.573782216	0.00028874

Enterocloster asparagiformis	SBS III	3.850408802	0.40301266	9.554064125	0.000107999
Collinsella aerofaciens	SBS III	3.698470293	0.587492304	6.295351055	0.02421882
Lawsonibacter asaccharolyticus	SBS III	3.629795661	0.369669881	9.819019217	0.001156557
Faecalibacterium sp. BCRC 81399	SBS III	3.620603996	0.480132659	7.540840909	0.04177637
Phascolarctobacterium faecium	SBS III	3.602530064	0.508735252	7.081345449	0.004144875
Ruminococcus bicirculans (ex Wegman et al. 2014)	SBS III	3.592112731	0.407299975	8.819329619	0.00250737
Clostridium sp. M62/1	SBS III	3.560292278	0.513597058	6.932072962	0.033225534
Oscillibacter sp. PEA192	SBS III	3.513911187	0.353139357	9.950494381	0.000981411
Subdoligranulum variabile	SBS III	3.323133158	0.411070875	8.084088064	0.011944379
uncultured Caproiciproducens sp.	SBS III	3.104744631	0.311363547	9.971445475	0.016072791
Christensenella minuta	SBS III	2.98055767	0.309247567	9.638095778	0.005251636
Intestinibacillus sp. NTUH-41-i26	SBS III	2.914767401	0.376510179	7.741536792	0.031475637
uncultured Sphaerochaeta sp.	SBS III	2.622237646	0.362750962	7.228754488	0.019822909
Pseudomonas sp. 31-12	SBS II	-3.435548589	0.208072284	-16.51132253	0.030971307
Butyricimonas faecihominis	SBS II	-3.939561075	0.387690874	-10.16160385	0.013516286
Butyricimonas virosa	SBS II	-5.197110032	0.414761235	-12.53036589	0.046780424
Pseudochrobactrum algeriensis	SBS II	-5.592975145	0.238346957	-23.46568722	0.003876149
Adlercreutzia hattorii	SBS II	-6.745930234	0.285574763	-23.62229132	0.000611612

Supplementary Table 5. Differential abundance analysis using MaAsLin2

**Species adjusted model for age and PN duration (SBS I vs. SBS II)**				
feature	Enriched group	coef	stderr	q-values

NA	NA	NA	NA	NA
**Species adjusted model for age and height (SBS II vs. SBS III)**				
feature	Enriched group	coef	stderr	q-values
Ruminococcus.sp..SR1.5	SBS III	8.648520059	0.944514963	0.0000791
Blautia.wexlerae	SBS III	8.732541294	1.052067644	0.000228355
Faecalibacterium.sp..BCRC.81399	SBS III	7.289630435	0.939928716	0.00048877
Blautia.luti	SBS III	8.820303372	1.182235462	0.000560454
Lachnospira.eligens	SBS III	7.680475343	1.021768489	0.000560454
Eubacterium.ventriosum	SBS III	7.242911957	0.99485594	0.00069805
Ruminococcus.bicirculans..ex.Wegman.et.al..2014.	SBS III	7.253972732	1.012467316	0.000776077
Roseburia.rectibacter	SBS III	7.96950696	1.155302552	0.001105994
X.Ruminococcus..torques	SBS III	9.2349406	1.333792547	0.001105994
Blautia.coccoides	SBS III	8.055171101	1.188056258	0.001170798
Oscillibacter.sp..PEA192	SBS III	7.298704593	1.074629168	0.001170798
X.Ruminococcus..lactaris	SBS III	9.204144371	1.363916542	0.001170798
Dorea.formicigenerans	SBS III	8.246954951	1.23653599	0.001297234
Dorea.longicatena	SBS III	8.303345243	1.264709851	0.001533071
Romboutsia.ilealis	SBS III	7.136911992	1.091920168	0.001533071
Blautia.obeum	SBS III	8.095324595	1.267250981	0.001889428
Dysosmobacter.welbionis	SBS III	7.677430513	1.21391117	0.001889428
Faecalibacterium.sp..I2.3.92	SBS III	5.826549804	0.9118601	0.001889428
Faecalibacterium.sp..IP.1.18	SBS III	5.982024491	0.945052007	0.001889428
Mediterraneibacter.gnavus	SBS III	9.823364339	1.544655815	0.001889428
Anaerostipes.hadrus	SBS III	7.458849352	1.192892571	0.002132369
Wujia.chipingensis	SBS III	6.908927793	1.117558584	0.002406459
Blautia.sp..SC05B48	SBS III	7.407812857	1.215416673	0.002715548
Ruthenibacterium.lactatiformans	SBS III	8.277386786	1.357694648	0.002715548
Blautia.pseudococcoides	SBS III	6.913556772	1.144585742	0.002970498
Flavonifractor.plautii	SBS III	9.665465306	1.621517239	0.003097959
Lachnoclostridium.phytofermentans	SBS III	5.889774544	0.983667158	0.003097959
Wansuia.hejianensis	SBS III	6.915286665	1.154995712	0.003097959
Clostridium.sp..M62.1	SBS III	6.83970923	1.156238193	0.00323111
Coprococcus.sp..ART55.1	SBS III	6.768285756	1.143751329	0.00323111
Coprococcus.comes	SBS III	7.91318966	1.350739133	0.003482445
Roseburia.sp..499	SBS III	7.154998089	1.220023886	0.003482445
Enterocloster.asparagiformis	SBS III	6.565956946	1.127040852	0.003656394
Faecalibacterium.sp..IP.3.29	SBS III	5.17770384	0.903321299	0.004445336
Marvinbryantia.formatexigens	SBS III	7.53287571	1.316600965	0.004445336
Blautia.hydrogenotrophica	SBS III	7.185297626	1.277241705	0.004966195
Enterocloster.bolteae	SBS III	7.541673675	1.341385056	0.004966195
Hungatella.hathewayi	SBS III	7.214446703	1.276230884	0.004966195

Methanosphaera.stadtmanae	SBS III	5.440232932	0.964387128	0.004966195
Ruminococcus.gauvreauii	SBS III	6.993680169	1.243328402	0.004966195
Anaerobutyricum.hallii	SBS III	7.931313868	1.41693835	0.005029969
Blautia.producta	SBS III	8.033636536	1.433896557	0.005029969
Eubacterium.limosum	SBS III	6.650835843	1.192630804	0.005172384
Butyrivibrio.hungatei	SBS III	4.916943523	0.885640172	0.005199271
Coprococcus.eutactus	SBS III	6.066107299	1.098515322	0.005199271
Pusillibacter.faecalis	SBS III	6.153377173	1.108405257	0.005199271
Roseburia.hominis	SBS III	7.754805554	1.404296581	0.005199271
Simiaoa.sunii	SBS III	7.334097985	1.327159369	0.005199271
X.Clostridium..scindens	SBS III	8.168286899	1.472630394	0.005199271
Roseburia.sp..831b	SBS III	7.608242003	1.382270781	0.005325645
Blautia.liquoris	SBS III	7.315729184	1.333392384	0.005452981
Novisyntrophococcus.fermenticellae	SBS III	6.974263464	1.277320852	0.005695439
Qiania.dongpingensis	SBS III	6.636215966	1.218580684	0.005695439
Blautia.argi	SBS III	8.759542582	1.613388677	0.00576364
Romboutsia.sp..CE17	SBS III	5.909476323	1.08902004	0.00576364
Romboutsia.sp..13368	SBS III	5.649629071	1.044910872	0.005943212
Anaerocolumna.sp..AGMB13025	SBS III	5.919242396	1.10273076	0.006429164
Acutalibacter.muris	SBS III	5.716153138	1.078591688	0.007165182
Collinsella.aerofaciens	SBS III	5.886539656	1.11077951	0.007165182
Intestinibacillus.sp..NTUH.41.i26	SBS III	5.875122892	1.109139162	0.007165182
Anaerotruncus.colihominis	SBS III	6.874871351	1.307525053	0.007646104
Anaerostipes.rhamnosivorans	SBS III	5.938689521	1.133400307	0.007789262
Clostridium.septicum	SBS III	7.109442324	1.366899597	0.008143333
Clostridium.sp..SY8519	SBS III	5.859856077	1.126167017	0.008143333
Coprococcus.catus	SBS III	6.897705434	1.327361165	0.008143333
Lawsonibacter.asaccharolyticus	SBS III	5.879258156	1.12833607	0.008143333
Faecalibacterium.sp..I3.3.89	SBS III	6.608670594	1.274069225	0.008151325
Phascolarctobacterium.faecium	SBS III	5.711313158	1.102798215	0.008160575
Oscillibacter.hominis	SBS III	6.798152878	1.317594607	0.008447059
Anaerocolumna.sp..AGMB13020	SBS III	5.703743923	1.107174591	0.0085001
Faecalibacterium.prausnitzii	SBS III	7.025956351	1.372906205	0.008895005
Vescimonas.coprocola	SBS III	6.106868662	1.19165672	0.008895005
Faecalibacillus.intestinalis	SBS III	5.90529187	1.164521105	0.009734739
X.Clostridium..hylemonae	SBS III	7.073337742	1.39966064	0.01003917
Eggerthella.lenta	SBS III	5.650745622	1.119736215	0.010094434
Subdoligranulum.variabile	SBS III	6.069021839	1.223058006	0.012293011
Flintibacter.sp..KGMB00164	SBS III	5.734354761	1.169383005	0.014044437
Faecalibacterium.sp..I4.3.84	SBS III	6.16103031	1.260509382	0.014444401
Massilistercora.timonensis	SBS III	5.127783653	1.059008209	0.015994501

Lactococcus.garvieae	SBS III	4.88323344	1.011253243	0.016155527
Anaerocolumna.cellulosilytica	SBS III	5.533216659	1.150940603	0.016480301
Lacrimispora.xylanolytica	SBS III	5.643559051	1.172872085	0.016480301
Anaerotignum.sp..MB30.C6	SBS III	4.812376958	1.003056561	0.016707438
Alkaliphilus.sp..B6464	SBS III	4.450229979	0.929016668	0.016839112
Clostridium.cadaveris	SBS III	5.953595122	1.246719927	0.017289137
Raoultibacter.timonensis	SBS III	3.814651708	0.799847293	0.017374309
Sellimonas.intestinalis	SBS III	7.493112121	1.573058761	0.017446196
Butyrivibrio.crossotus	SBS III	4.857695683	1.021714817	0.01765715
Agathobacter.rectalis	SBS III	6.749403966	1.422464205	0.017900939
Eggerthella.guodeyinii	SBS III	4.001779317	0.850952636	0.019297874
Lachnoclostridium.phocaeense	SBS III	5.710785509	1.216175494	0.019447298
Vescimonas.fastidiosa	SBS III	5.52245793	1.194659831	0.022886956
Faecalibacterium.duncaniae	SBS III	5.433485563	1.179848288	0.02323638
Claveliimonas.bilis	SBS III	6.858741391	1.507186522	0.026377692
Anaeromicropila.herbilytica	SBS III	5.129437382	1.133022771	0.027004997
Faecalibacterium.sp..HTF.F	SBS III	5.515388592	1.219332665	0.027004997
Listeria.weihenstephanensis	SBS III	3.583218888	0.791605159	0.027004997
Roseburia.intestinalis	SBS III	7.274872246	1.605569756	0.027004997
Blautia.parvula	SBS III	6.979536558	1.55248107	0.0284275
Lacrimispora.sphenoides	SBS III	5.724360619	1.27612492	0.028895947
Chordicoccus.furentiruminis	SBS III	4.800911126	1.079275035	0.030933337
Ruminococcus.albus	SBS III	4.424992603	0.996280338	0.031199439
Intestinimonas.butyriciproducens	SBS III	5.674687959	1.28443835	0.032281312
Intestinibacter.bartlettii	SBS III	5.534444228	1.258405634	0.033398196
Lactococcus.petauri	SBS III	4.160513228	0.95712855	0.037057085
Vibrio.parahaemolyticus	SBS III	3.978329685	0.920720285	0.038934143
Luteolibacter.ambystomatis	SBS III	3.357091235	0.777521874	0.038949519
Anaerocolumna.chitinilytica	SBS III	4.959161174	1.15142318	0.039113523
Eubacterium.callanderi	SBS III	6.007840136	1.39410439	0.039113523
Paenibacillus.sp..DCT19	SBS III	3.193521337	0.741205743	0.039113523
Christensenella.minuta	SBS III	4.4421145	1.03731106	0.041286946
Bacteroides.ovatus	SBS III	5.117259717	1.202782772	0.043610581
Fusobacterium.canifelinum	SBS III	4.6455371	1.093708473	0.044059749
Antarcticibacterium.arcticum	SBS III	2.879469983	0.680161591	0.044977227
Butyrivibrio.fibrisolvens	SBS III	4.976988056	1.183208753	0.047453615
Romboutsia.hominis	SBS III	5.47315561	1.303724287	0.048103353
Anaerocolumna.sedimenticola	SBS III	4.729503192	1.127438159	0.048151743

Supplementary Table 6. Correlations in SBS network compared to that in control network

Group	Source	Target	SBS_corr	SBS_pval	SBS_qval	Control_corr	Control_pval	Control_qval	Source_Kingdom	Target_Kingdom	Type
SBS I vs. Control	Aaosphaeria arxii	Penicillium soppii	-0.2632	0.004	0.892940731	NA	NA	NA	Bacteria	Bacteria	intra
SBS I vs. Control	Abyssalbus ytuae	Glaciihabitans sp. INWT7	-0.2819	0.001	0.892940731	NA	NA	NA	Bacteria	Bacteria	intra
SBS I vs. Control	Abyssalbus ytuae	Piscinibacter gummiphilus	0.3495	0.002	0.892940731	NA	NA	NA	Bacteria	Bacteria	intra
SBS I vs. Control	Acaromyces ingoldii	Clostridium septicum	0.2856	0.004	0.892940731	NA	NA	NA	Bacteria	Bacteria	intra
SBS I vs. Control	Acaromyces ingoldii	Fusobacterium hwasookii	-0.3355	0.002	0.892940731	NA	NA	NA	Bacteria	Bacteria	intra
SBS I vs. Control	Acidipropionibacterium jensenii	Candidatus Sulfidibacterium hydrothermale	0.259	0.004	0.892940731	NA	NA	NA	Bacteria	Bacteria	intra
SBS I vs. Control	Acidipropionibacterium jensenii	Curtobacterium sp. SGAir0471	-0.2596	0.001	0.892940731	NA	NA	NA	Bacteria	Bacteria	intra
SBS I vs. Control	Acidipropionibacterium jensenii	uncultured Methanobrevibacter sp	-0.2999	0.003	0.892940731	NA	NA	NA	Bacteria	Bacteria	intra
SBS I vs. Control	Acidisoma sp. PAMC 29798	Protaetiibacter intestinalis	0.2599	0.004	0.892940731	NA	NA	NA	Bacteria	Bacteria	intra
SBS I vs. Control	Acidithiobacillus caldus	Cedecea neteri	-0.2525	0.001	0.892940731	NA	NA	NA	Bacteria	Bacteria	intra
SBS I vs. Control	Acidithiobacillus caldus	Raoultella ornithinolytica	-0.2742	0.001	0.892940731	NA	NA	NA	Bacteria	Bacteria	intra
SBS I vs. Control	Acinetobacter oleivorans	Bordetella trematum	0.3611	0.003	0.892940731	NA	NA	NA	Bacteria	Bacteria	intra
SBS I vs. Control	Acinetobacter oleivorans	Nocardia yunnanensis	0.3293	0.003	0.892940731	NA	NA	NA	Bacteria	Bacteria	intra
SBS I vs. Control	Acinetobacter schindleri	Cupriavidus pinatubonensis	-0.3516	0.004	0.892940731	NA	NA	NA	Bacteria	Bacteria	intra
SBS I vs. Control	Acinetobacter schindleri	Mycolicibacterium celeriflavum	-0.3051	0.002	0.892940731	NA	NA	NA	Bacteria	Bacteria	intra
SBS I vs. Control	Acinetobacter sp. AS23	Auraticoccus monumenti	0.2961	0.004	0.892940731	NA	NA	NA	Bacteria	Bacteria	intra
SBS I vs. Control	Acinetobacter sp. AS23	Rhodococcus globerulus	0.2736	0.004	0.892940731	NA	NA	NA	Bacteria	Bacteria	intra
SBS I vs. Control	Acinetobacter sp. WCHAc010034	Priestia koreensis	0.2564	0.004	0.892940731	NA	NA	NA	Bacteria	Bacteria	intra
SBS I vs.	Actibacterium sp. EMB200-NS6	Lysobacter avium	0.379	0.002	0.892940731	NA	NA	NA	Bacteria	Bacteria	intra

Control											
SBS I vs. Control	Actinacidiphila glaucinigra	Methylobacterium phyllosphaerae	-0.337	0.001	0.892940731	NA	NA	NA	Bacteria	Bacteria	intra
SBS I vs. Control	Actinobacillus equuli	Ornithinimicrobium cryptoxanthini	0.3709	0.001	0.892940731	NA	NA	NA	Bacteria	Bacteria	intra
SBS I vs. Control	Actinobacillus equuli	Paenibacillus tritici	0.2668	0.004	0.892940731	NA	NA	NA	Bacteria	Bacteria	intra
SBS I vs. Control	Actinobaculum sp. 313	Micromonospora sp. WMMD882	0.2796	0.004	0.892940731	NA	NA	NA	Bacteria	Bacteria	intra
SBS I vs. Control	Actinomadura madurae	Bradyrhizobium sp. BWC-3-1	0.2576	0.001	0.892940731	NA	NA	NA	Bacteria	Bacteria	intra
SBS I vs. Control	Actinomadura madurae	Corynebacterium cyclohexanicum	-0.2988	0.002	0.892940731	NA	NA	NA	Bacteria	Bacteria	intra
SBS I vs. Control	Actinomadura madurae	Lachnoclostridium phytofermentans	-0.312	0.004	0.892940731	NA	NA	NA	Bacteria	Bacteria	intra
SBS I vs. Control	Actinomadura madurae	Streptococcus mitis	-0.2741	0.003	0.892940731	NA	NA	NA	Bacteria	Bacteria	intra
SBS I vs. Control	Actinomadura verrucosospora	Corynebacterium hindlerae	0.2511	0.003	0.892940731	NA	NA	NA	Bacteria	Bacteria	intra
SBS I vs. Control	Actinomadura verrucosospora	Hymenobacter sp. 5317J-9	0.3935	0.001	0.892940731	NA	NA	NA	Bacteria	Bacteria	intra
SBS I vs. Control	Actinomadura verrucosospora	Pseudomonas umsongensis	0.3193	0.004	0.892940731	NA	NA	NA	Bacteria	Bacteria	intra
SBS I vs. Control	Actinomyces gaoshouyii	Arthrobacter sp. zg-Y820	-0.2698	0.003	0.892940731	NA	NA	NA	Bacteria	Bacteria	intra
SBS I vs. Control	Actinomyces gaoshouyii	Fusobacterium nucleatum	0.2868	0.003	0.892940731	NA	NA	NA	Bacteria	Bacteria	intra
SBS I vs. Control	Actinomyces gaoshouyii	Polaribacter sp. KT25b	-0.291	0.004	0.892940731	NA	NA	NA	Bacteria	Bacteria	intra
SBS I vs. Control	Actinomyces gaoshouyii	Streptomyces sp. HUAS 2-6	0.3004	0.004	0.892940731	NA	NA	NA	Bacteria	Bacteria	intra
SBS I vs. Control	Actinomyces lilanjuaniae	Buchnera aphidicola	0.2715	0.003	0.892940731	NA	NA	NA	Bacteria	Bacteria	intra
SBS I vs. Control	Actinomyces lilanjuaniae	Flavobacterium sp. N2038	-0.2677	0.002	0.892940731	NA	NA	NA	Bacteria	Bacteria	intra
SBS I vs. Control	Actinomyces lilanjuaniae	Veillonella sp. S12025-13	0.2881	0.003	0.892940731	NA	NA	NA	Bacteria	Bacteria	intra
SBS I vs. Control	Actinomyces marmotae	Aminipila luticellarii	-0.3033	0.002	0.892940731	NA	NA	NA	Bacteria	Bacteria	intra
SBS I vs. Control	Actinomyces marmotae	Verminephrobacter eiseniae	-0.2742	0.001	0.892940731	NA	NA	NA	Bacteria	Bacteria	intra

SBS I vs. Control	Actinomyces pacaensis	Agromyces laixinhei	0.2868	0.004	0.892940731	NA	NA	NA	Bacteria	Bacteria	intra
SBS I vs. Control	Actinomyces pacaensis	Corynebacterium variabile	-0.2664	0.001	0.892940731	NA	NA	NA	Bacteria	Bacteria	intra
SBS I vs. Control	Actinomyces pacaensis	Faecalibacterium prausnitzii	-0.2735	0.003	0.892940731	NA	NA	NA	Bacteria	Bacteria	intra
SBS I vs. Control	Actinomyces pacaensis	Neoehrlichia mikurensis	-0.2556	0.004	0.892940731	NA	NA	NA	Bacteria	Bacteria	intra
SBS I vs. Control	Actinomyces sp. HMT897	Phaeoacremonium minimum	0.2645	0.004	0.892940731	NA	NA	NA	Bacteria	Bacteria	intra
SBS I vs. Control	Actinomyces sp. oral taxon 169	Streptomyces sp. NBC_01296	0.3586	0.003	0.892940731	NA	NA	NA	Bacteria	Bacteria	intra
SBS I vs. Control	Actinomyces sp. zg-332	Paracoccus zhejiangensis	0.3017	0.003	0.892940731	NA	NA	NA	Bacteria	Bacteria	intra
SBS I vs. Control	Actinomyces sp. zg-332	Variovorax sp. RKNM96	-0.2526	0.003	0.892940731	NA	NA	NA	Bacteria	Bacteria	intra
SBS I vs. Control	Actinoplanes friuliensis	Arthrobacter sp. NicSoilB8	-0.3073	0.004	0.892940731	NA	NA	NA	Bacteria	Bacteria	intra
SBS I vs. Control	Actinoplanes friuliensis	Avibacterium paragallinarum	-0.2689	0.003	0.892940731	NA	NA	NA	Bacteria	Bacteria	intra
SBS I vs. Control	Actinoplanes oblitus	Nitrospina watsonii	-0.2976	0.002	0.892940731	NA	NA	NA	Bacteria	Bacteria	intra
SBS I vs. Control	Actinoplanes oblitus	Priestia koreensis	0.2561	0.003	0.892940731	NA	NA	NA	Bacteria	Bacteria	intra
SBS I vs. Control	Actinoplanes sichuanensis	Pseudomonas cavernae	0.3286	0.003	0.892940731	NA	NA	NA	Bacteria	Bacteria	intra
SBS I vs. Control	Actinoplanes sp. NBC_00393	Aeromonas media	-0.2522	0.003	0.892940731	NA	NA	NA	Bacteria	Bacteria	intra
SBS I vs. Control	Actinoplanes sp. NBC_00393	Caldicellulosiruptor hydrothermalis	-0.2828	0.004	0.892940731	NA	NA	NA	Bacteria	Bacteria	intra
SBS I vs. Control	Actinoplanes sp. NBC_00393	Dorea longicatena	-0.2721	0.003	0.892940731	NA	NA	NA	Bacteria	Bacteria	intra
SBS I vs. Control	Actinoplanes sp. NBC_00393	Paenibacillus sp. MBLB1832	-0.2529	0.001	0.892940731	NA	NA	NA	Bacteria	Bacteria	intra
SBS I vs. Control	Actinoplanes sp. NBC_00393	Paludicola sp. MB14-C6	0.2673	0.001	0.892940731	NA	NA	NA	Bacteria	Bacteria	intra
SBS I vs. Control	Actinoplanes sp. NBC_00393	Shinella sp. XGS7	-0.2712	0.003	0.892940731	NA	NA	NA	Bacteria	Bacteria	intra
SBS I vs. Control	Adhaeribacter radiodurans	Halogeometricum sp. S1BR25-6	0.2811	0.003	0.892940731	NA	NA	NA	Bacteria	Archaea	inter
SBS I vs.	Adhaeribacter radiodurans	Plantibacter flavus	0.2982	0.001	0.892940731	NA	NA	NA	Bacteria	Bacteria	intra

Control											
SBS I vs. Control	Aequorivita sp. Ant34-E75	Pseudoduganella flava	0.3658	0.003	0.892940731	NA	NA	NA	Bacteria	Bacteria	intra
SBS I vs. Control	Aequorivita sp. Ant34-E75	Streptomyces durmitorensis	-0.2623	0.001	0.892940731	NA	NA	NA	Bacteria	Bacteria	intra
SBS I vs. Control	Aeromicrobium choanae	Fusobacterium hwasookii	0.2793	0.002	0.892940731	NA	NA	NA	Bacteria	Bacteria	intra
SBS I vs. Control	Aeromicrobium duanguangcaii	Microvirga sp. VF16	0.2678	0.002	0.892940731	NA	NA	NA	Bacteria	Bacteria	intra
SBS I vs. Control	Aeromicrobium erythreum	Flavonifractor plautii	0.2808	0.003	0.892940731	NA	NA	NA	Bacteria	Bacteria	intra
SBS I vs. Control	Aeromicrobium wangtongii	Micromonospora sp. HUAS LYJ1	0.3371	0.001	0.892940731	NA	NA	NA	Bacteria	Bacteria	intra
SBS I vs. Control	Aeromicrobium wangtongii	Paraburkholderia phenoliruptrix	-0.2875	0.003	0.892940731	NA	NA	NA	Bacteria	Bacteria	intra
SBS I vs. Control	Aeromonas media	Microbacterium suwonense	-0.3533	0.004	0.892940731	NA	NA	NA	Bacteria	Bacteria	intra
SBS I vs. Control	Aeromonas media	Pseudomonas alcaligenes	0.416	0.001	0.892940731	NA	NA	NA	Bacteria	Bacteria	intra
SBS I vs. Control	Aeromonas media	Variovorax boronicumulans	-0.3234	0.002	0.892940731	NA	NA	NA	Bacteria	Bacteria	intra
SBS I vs. Control	Aeromonas veronii	Pseudomonas cavernae	0.2705	0.001	0.892940731	NA	NA	NA	Bacteria	Bacteria	intra
SBS I vs. Control	Agathobacter rectalis	[Ruminococcus] torques	0.2683	0.001	0.892940731	NA	NA	NA	Bacteria	Bacteria	intra
SBS I vs. Control	Aggregatibacter actinomycetemcomitans	Blautia producta	0.2635	0.004	0.892940731	NA	NA	NA	Bacteria	Bacteria	intra
SBS I vs. Control	Aggregatibacter actinomycetemcomitans	Empedobacter brevis	-0.2826	0.004	0.892940731	NA	NA	NA	Bacteria	Bacteria	intra
SBS I vs. Control	Aggregatibacter aphrophilus	Streptomyces atratus	0.3016	0.003	0.892940731	NA	NA	NA	Bacteria	Bacteria	intra
SBS I vs. Control	Aggregatibacter aphrophilus	Streptomyces bathyalis	0.4141	0.003	0.892940731	NA	NA	NA	Bacteria	Bacteria	intra
SBS I vs. Control	Aggregatibacter segnis	Chordicoccus furentiruminis	-0.2571	0.001	0.892940731	NA	NA	NA	Bacteria	Bacteria	intra
SBS I vs. Control	Aggregatibacter segnis	Flavobacterium sp. IMCC34852	-0.3566	0.002	0.892940731	NA	NA	NA	Bacteria	Bacteria	intra
SBS I vs. Control	Aggregatibacter segnis	Halosegnis marinus	-0.3768	0.003	0.892940731	NA	NA	NA	Bacteria	Archaea	inter
SBS I vs. Control	Aggregatibacter segnis	Pseudarthrobacter sp. NIBRBAC000502772	-0.3038	0.004	0.892940731	NA	NA	NA	Bacteria	Bacteria	intra

SBS I vs. Control	Aggregatibacter segnis	Zhouia spongiae	-0.2848	0.003	0.892940731	NA	NA	NA	Bacteria	Bacteria	intra
SBS I vs. Control	Aggregatibacter sp. 2125159857	Haemophilus influenzae	-0.3779	0.001	0.892940731	NA	NA	NA	Bacteria	Bacteria	intra
SBS I vs. Control	Aggregatibacter sp. 2125159857	Ornithinimicrobium cryptoxanthini	-0.3941	0.004	0.892940731	NA	NA	NA	Bacteria	Bacteria	intra
SBS I vs. Control	Agrobacterium larrymoorei	Chordicoccus furentiruminis	-0.3781	0.001	0.892940731	NA	NA	NA	Bacteria	Bacteria	intra
SBS I vs. Control	Agrobacterium larrymoorei	Enterococcus gilvus	0.2877	0.003	0.892940731	NA	NA	NA	Bacteria	Bacteria	intra
SBS I vs. Control	Agrobacterium larrymoorei	Miltoncostaea oceani	-0.2769	0.003	0.892940731	NA	NA	NA	Bacteria	Bacteria	intra
SBS I vs. Control	Agrobacterium sp. MA01	Brachybacterium sp. NBEC-018	-0.3211	0.004	0.892940731	NA	NA	NA	Bacteria	Bacteria	intra
SBS I vs. Control	Agrobacterium sp. MA01	Corynebacterium incognita	-0.2717	0.002	0.892940731	NA	NA	NA	Bacteria	Bacteria	intra
SBS I vs. Control	Agrobacterium sp. MA01	Paenibacillus silvisoli	0.2806	0.001	0.892940731	NA	NA	NA	Bacteria	Bacteria	intra
SBS I vs. Control	Agrobacterium sp. MA01	Selenomonas sputigena	-0.2639	0.004	0.892940731	NA	NA	NA	Bacteria	Bacteria	intra
SBS I vs. Control	Agrobacterium sp. MA01	Wenyingzhuangia fucanilytica	-0.3282	0.004	0.892940731	NA	NA	NA	Bacteria	Bacteria	intra
SBS I vs. Control	Agrobacterium vitis	Microbacterium sp. ABRD28	0.2953	0.002	0.892940731	NA	NA	NA	Bacteria	Bacteria	intra
SBS I vs. Control	Agrococcus sp. Marseille-Q4369	Flavobacterium psychrophilum	-0.3706	0.004	0.892940731	NA	NA	NA	Bacteria	Bacteria	intra
SBS I vs. Control	Agrococcus sp. SCSIO52902	Peptostreptococcus equinus	0.2579	0.002	0.892940731	NA	NA	NA	Bacteria	Bacteria	intra
SBS I vs. Control	Agrococcus sp. SCSIO52902	Rothia dentocariosa	-0.3339	0.004	0.892940731	NA	NA	NA	Bacteria	Bacteria	intra
SBS I vs. Control	Agrococcus sp. SCSIO52902	Shewanella polaris	0.2966	0.002	0.892940731	NA	NA	NA	Bacteria	Bacteria	intra
SBS I vs. Control	Agrococcus sp. SCSIO52902	Streptococcus mitis	-0.5197	0.003	0.892940731	NA	NA	NA	Bacteria	Bacteria	intra
SBS I vs. Control	Agrococcus sp. SCSIO52902	Streptomyces rapamycinicus	-0.2509	0.004	0.892940731	NA	NA	NA	Bacteria	Bacteria	intra
SBS I vs. Control	Agromyces aureus	Tsuneonella sp. CC-YZS046	0.3207	0.004	0.892940731	NA	NA	NA	Bacteria	Bacteria	intra
SBS I vs. Control	Agromyces badenianii	Microbacterium sp. YJN-G	-0.3691	0.003	0.892940731	NA	NA	NA	Bacteria	Bacteria	intra
SBS I vs.	Agromyces flavus	Georgenia yuyongxinii	0.3198	0.003	0.892940731	NA	NA	NA	Bacteria	Bacteria	intra

Control											
SBS I vs. Control	Agromyces flavus	Stenotrophomonas sp. Pemsol	-0.257	0.003	0.892940731	NA	NA	NA	Bacteria	Bacteria	intra
SBS I vs. Control	Agromyces flavus	Streptomyces sp. RTd22	0.3599	0.001	0.892940731	NA	NA	NA	Bacteria	Bacteria	intra
SBS I vs. Control	Agromyces laixinhei	Thermoanaerobacterium thermosaccharolyticum	0.3116	0.002	0.892940731	NA	NA	NA	Bacteria	Bacteria	intra
SBS I vs. Control	Agromyces mangrovi	Labilithrix luteola	0.3589	0.002	0.892940731	NA	NA	NA	Bacteria	Bacteria	intra
SBS I vs. Control	Agromyces mangrovi	Mytilinidion resinicola	-0.2803	0.002	0.892940731	NA	NA	NA	Bacteria	Bacteria	intra
SBS I vs. Control	Agromyces mangrovi	Sporomusa ovata	-0.2858	0.003	0.892940731	NA	NA	NA	Bacteria	Bacteria	intra
SBS I vs. Control	Agromyces marinus	Arthrobacter crystallopoietes	0.2633	0.004	0.892940731	NA	NA	NA	Bacteria	Bacteria	intra
SBS I vs. Control	Agromyces marinus	Chromobacterium violaceum	-0.3407	0.004	0.892940731	NA	NA	NA	Bacteria	Bacteria	intra
SBS I vs. Control	Agromyces marinus	Clostridium isatidis	0.2569	0.003	0.892940731	NA	NA	NA	Bacteria	Bacteria	intra
SBS I vs. Control	Agromyces marinus	Thermoanaerobacterium sp. CMT5567-10	-0.3223	0.001	0.892940731	NA	NA	NA	Bacteria	Bacteria	intra
SBS I vs. Control	Algoriphagus sp. NBT04N3	Enterococcus rotai	0.4219	0.001	0.892940731	NA	NA	NA	Bacteria	Bacteria	intra
SBS I vs. Control	Alkalitalea saponilacus	Corynebacterium frankenforstense	-0.2699	0.003	0.892940731	NA	NA	NA	Bacteria	Bacteria	intra
SBS I vs. Control	Alkalitalea saponilacus	Nocardioides scoriae	0.2876	0.003	0.892940731	NA	NA	NA	Bacteria	Bacteria	intra
SBS I vs. Control	Alkalitalea saponilacus	Streptomyces sp. NBC_00370	0.2793	0.002	0.892940731	NA	NA	NA	Bacteria	Bacteria	intra
SBS I vs. Control	Allobaculum mucilyticum	Microbacterium sp. CGR1	-0.2732	0.002	0.892940731	NA	NA	NA	Bacteria	Bacteria	intra
SBS I vs. Control	Allobaculum mucilyticum	Trinickia caryophylli	0.3283	0.003	0.892940731	NA	NA	NA	Bacteria	Bacteria	intra
SBS I vs. Control	Allobaculum mucilyticum	Veillonella sp. S12025-13	0.3657	0.001	0.892940731	NA	NA	NA	Bacteria	Bacteria	intra
SBS I vs. Control	Allokutzneria albata	Microterricola viridarii	0.4284	0.002	0.892940731	NA	NA	NA	Bacteria	Bacteria	intra
SBS I vs. Control	Alphabaculovirus chorosaceanae	Streptococcus merionis	-0.268	0.002	0.892940731	NA	NA	NA	Bacteria	Bacteria	intra
SBS I vs. Control	Altererythrobacter epoxidivorans	Rothia amarae	-0.2641	0.004	0.892940731	NA	NA	NA	Bacteria	Bacteria	intra

SBS I vs. Control	Altererythrobacter epoxidivorans	Streptomyces sp. SCA4-21	0.2577	0.001	0.892940731	NA	NA	NA	Bacteria	Bacteria	intra
SBS I vs. Control	Aminipila luticellarii	Blautia producta	-0.3027	0.001	0.892940731	NA	NA	NA	Bacteria	Bacteria	intra
SBS I vs. Control	Aminipila luticellarii	Corynebacterium pseudokroppenstedtii	0.3544	0.002	0.892940731	NA	NA	NA	Bacteria	Bacteria	intra
SBS I vs. Control	Aminipila luticellarii	Geomonas subterranea	0.2574	0.004	0.892940731	NA	NA	NA	Bacteria	Bacteria	intra
SBS I vs. Control	Aminipila luticellarii	Glutamicibacter creatinolyticus	-0.2732	0.001	0.892940731	NA	NA	NA	Bacteria	Bacteria	intra
SBS I vs. Control	Amycolatopsis roodepoortensis	Microbacterium sp. NIBRBAC000506063	-0.293	0.001	0.892940731	NA	NA	NA	Bacteria	Bacteria	intra
SBS I vs. Control	Amycolatopsis sp. BJA-103	Basilea psittacipulmonis	-0.3756	0.001	0.892940731	NA	NA	NA	Bacteria	Bacteria	intra
SBS I vs. Control	Amycolatopsis sp. BJA-103	Streptococcus sp. oral taxon 064	-0.2738	0.003	0.892940731	NA	NA	NA	Bacteria	Bacteria	intra
SBS I vs. Control	Amycolatopsis sp. BJA-103	[Ruminococcus] torques	0.3168	0.003	0.892940731	NA	NA	NA	Bacteria	Bacteria	intra
SBS I vs. Control	Amycolatopsis sp. FDAARGOS 1241	Beutenbergia cavernae	-0.2554	0.001	0.892940731	NA	NA	NA	Bacteria	Bacteria	intra
SBS I vs. Control	Amycolatopsis sp. FDAARGOS 1241	Methanocaldococcus infernus	0.3659	0.004	0.892940731	NA	NA	NA	Bacteria	Bacteria	intra
SBS I vs. Control	Amycolatopsis sp. NBC_01286	Nitrospirillum amazonense	-0.3844	0.003	0.892940731	NA	NA	NA	Bacteria	Bacteria	intra
SBS I vs. Control	Amycolatopsis sp. NBC_01286	Rhizorhabdus dicambivorans	-0.2775	0.004	0.892940731	NA	NA	NA	Bacteria	Bacteria	intra
SBS I vs. Control	Anaerocolumna sp. MB42-C2	Aquitalea aquatilis	0.2605	0.001	0.892940731	NA	NA	NA	Bacteria	Bacteria	intra
SBS I vs. Control	Anaerocolumna sp. MB42-C2	Gordonia hydrophobica	-0.2597	0.004	0.892940731	NA	NA	NA	Bacteria	Bacteria	intra
SBS I vs. Control	Anaerocolumna sp. MB42-C2	Variovorax sp. RA8	0.2662	0.002	0.892940731	NA	NA	NA	Bacteria	Bacteria	intra
SBS I vs. Control	Anaeromyxobacter paludicola	Oceanisphaera sp. IT1-181	0.2508	0.004	0.892940731	NA	NA	NA	Bacteria	Bacteria	intra
SBS I vs. Control	Anaeromyxobacter paludicola	Plantibacter flavus	-0.2926	0.003	0.892940731	NA	NA	NA	Bacteria	Bacteria	intra
SBS I vs. Control	Anaerostipes caccae	Methylococcus geothermalis	-0.3525	0.001	0.892940731	NA	NA	NA	Bacteria	Bacteria	intra
SBS I vs. Control	Anaerostipes hadrus	Streptomyces sp. NBC_01727	0.28	0.002	0.892940731	NA	NA	NA	Bacteria	Bacteria	intra
SBS I vs.	Anaerostipes hadrus	Wenyingzhuangia fucanilytica	0.2984	0.003	0.892940731	NA	NA	NA	Bacteria	Bacteria	intra

Control											
SBS I vs. Control	Anaerostipes rhamnosivorans	Caldicellulosiruptor hydrothermalis	-0.2742	0.004	0.892940731	NA	NA	NA	Bacteria	Bacteria	intra
SBS I vs. Control	Anaerostipes rhamnosivorans	Streptococcus sp. Marseille-Q6470	-0.3272	0.001	0.892940731	NA	NA	NA	Bacteria	Bacteria	intra
SBS I vs. Control	Anaerotruncus colihominis	Natronobiforma cellulositropha	-0.2942	0.002	0.892940731	NA	NA	NA	Bacteria	Bacteria	intra
SBS I vs. Control	Aneurinibacillus uraniidurans	Streptococcus sp. FSL W8-0197	-0.3168	0.003	0.892940731	NA	NA	NA	Bacteria	Bacteria	intra
SBS I vs. Control	Anoxybacter fermentans	Candidatus Sulfidibacterium hydrothermale	-0.278	0.004	0.892940731	NA	NA	NA	Bacteria	Bacteria	intra
SBS I vs. Control	Anoxybacter fermentans	Kineothrix sp. MB12-C1	0.3061	0.003	0.892940731	NA	NA	NA	Bacteria	Bacteria	intra
SBS I vs. Control	Aquibium microcysteis	Imperialibacter roseus	-0.2651	0.003	0.892940731	NA	NA	NA	Bacteria	Bacteria	intra
SBS I vs. Control	Aquiflexum balticum	Mycolicibacter virginiensis	0.3792	0.003	0.892940731	NA	NA	NA	Bacteria	Bacteria	intra
SBS I vs. Control	Aquiflexum balticum	Schaalia sp. ZJ405	0.3354	0.002	0.892940731	NA	NA	NA	Bacteria	Bacteria	intra
SBS I vs. Control	Aquiflexum balticum	Tannockella kyphosi	-0.3197	0.001	0.892940731	NA	NA	NA	Bacteria	Bacteria	intra
SBS I vs. Control	Aquisediminimonas profunda	Mucilaginibacter celer	-0.2798	0.003	0.892940731	NA	NA	NA	Bacteria	Bacteria	intra
SBS I vs. Control	Aquisediminimonas profunda	Rathayibacter oskolensis	-0.2948	0.002	0.892940731	NA	NA	NA	Bacteria	Bacteria	intra
SBS I vs. Control	Aquitalea aquatilis	Halopseudomonas nanhaiensis	-0.3535	0.004	0.892940731	NA	NA	NA	Bacteria	Bacteria	intra
SBS I vs. Control	Arabiibacter massiliensis	Clostridium botulinum	0.2783	0.003	0.892940731	NA	NA	NA	Bacteria	Bacteria	intra
SBS I vs. Control	Arabiibacter massiliensis	Enterobacter sp. PGRG2	-0.2862	0.001	0.892940731	NA	NA	NA	Bacteria	Bacteria	intra
SBS I vs. Control	Arabiibacter massiliensis	Micromonospora purpureochromogenes	0.3642	0.002	0.892940731	NA	NA	NA	Bacteria	Bacteria	intra
SBS I vs. Control	Arcanobacterium canis	Botrimarina mediterranea	-0.3388	0.004	0.892940731	NA	NA	NA	Bacteria	Bacteria	intra
SBS I vs. Control	Arcanobacterium haemolyticum	Blautia obeum	-0.3811	0.003	0.892940731	NA	NA	NA	Bacteria	Bacteria	intra
SBS I vs. Control	Arcanobacterium haemolyticum	Gluconobacter oxydans	-0.2577	0.002	0.892940731	NA	NA	NA	Bacteria	Bacteria	intra
SBS I vs. Control	Arcanobacterium haemolyticum	Ralstonia sp. RRA.1	0.2953	0.004	0.892940731	NA	NA	NA	Bacteria	Bacteria	intra

SBS I vs. Control	Archangium violaceum	Brachybacterium huguangmaarense	-0.251	0.002	0.892940731	NA	NA	NA	Bacteria	Bacteria	intra
SBS I vs. Control	Archangium violaceum	Corynebacterium flavescens	0.3145	0.003	0.892940731	NA	NA	NA	Bacteria	Bacteria	intra
SBS I vs. Control	Archangium violaceum	Cupriavidus sp. USMAA2-4	-0.2507	0.004	0.892940731	NA	NA	NA	Bacteria	Bacteria	intra
SBS I vs. Control	Arthrobacter alpinus	Gluconobacter oxydans	0.2553	0.004	0.892940731	NA	NA	NA	Bacteria	Bacteria	intra
SBS I vs. Control	Arthrobacter caoxuetaonis	Sphingomonas bisphenolicum	-0.3246	0.002	0.892940731	NA	NA	NA	Bacteria	Bacteria	intra
SBS I vs. Control	Arthrobacter caoxuetaonis	Variovorax sp. PBS-H4	-0.3061	0.001	0.892940731	NA	NA	NA	Bacteria	Bacteria	intra
SBS I vs. Control	Arthrobacter crystallopoietes	Saccharomonospora viridis	-0.2903	0.004	0.892940731	NA	NA	NA	Bacteria	Bacteria	intra
SBS I vs. Control	Arthrobacter crystallopoietes	Streptomyces sp. NBC_01280	0.2834	0.001	0.892940731	NA	NA	NA	Bacteria	Bacteria	intra
SBS I vs. Control	Arthrobacter jiangjiafuii	Paracoccus stylophorae	0.2778	0.002	0.892940731	NA	NA	NA	Bacteria	Bacteria	intra
SBS I vs. Control	Arthrobacter koreensis	Diaphorobacter nitroreducens	-0.2715	0.004	0.892940731	NA	NA	NA	Bacteria	Bacteria	intra
SBS I vs. Control	Arthrobacter koreensis	Mariniflexile sp. TRM1-10	0.2802	0.002	0.892940731	NA	NA	NA	Bacteria	Bacteria	intra
SBS I vs. Control	Arthrobacter koreensis	Nesterenkonia pannonica	-0.288	0.002	0.892940731	NA	NA	NA	Bacteria	Bacteria	intra
SBS I vs. Control	Arthrobacter koreensis	Verminephrobacter eiseniae	-0.2657	0.002	0.892940731	NA	NA	NA	Bacteria	Bacteria	intra
SBS I vs. Control	Arthrobacter sp. ATA002	Nocardiopsis alba	-0.2891	0.003	0.892940731	NA	NA	NA	Bacteria	Bacteria	intra
SBS I vs. Control	Arthrobacter sp. CDRTa11	Yersinia frederiksenii	-0.2692	0.003	0.892940731	NA	NA	NA	Bacteria	Bacteria	intra
SBS I vs. Control	Arthrobacter sp. DNA4	Vibrio mimicus	-0.3084	0.002	0.892940731	NA	NA	NA	Bacteria	Bacteria	intra
SBS I vs. Control	Arthrobacter sp. ERGS1:01	Mycobacterium vicinigordonae	-0.2727	0.002	0.892940731	NA	NA	NA	Bacteria	Bacteria	intra
SBS I vs. Control	Arthrobacter sp. FB24	Olsenella timonensis	-0.2745	0.002	0.892940731	NA	NA	NA	Bacteria	Bacteria	intra
SBS I vs. Control	Arthrobacter sp. FB24	Stutzerimonas stutzeri	0.2932	0.001	0.892940731	NA	NA	NA	Bacteria	Bacteria	intra
SBS I vs. Control	Arthrobacter sp. Helios	Corynebacterium occultum	0.2889	0.004	0.892940731	NA	NA	NA	Bacteria	Bacteria	intra
SBS I vs.	Arthrobacter sp. Helios	Mycobacterium kansasii	-0.2556	0.003	0.892940731	NA	NA	NA	Bacteria	Bacteria	intra

Control											
SBS I vs. Control	Arthrobacter sp. KBS0702	Faecalibacterium sp. I4-1-79	-0.3105	0.001	0.892940731	NA	NA	NA	Bacteria	Bacteria	intra
SBS I vs. Control	Arthrobacter sp. KBS0702	Ornithinimicrobium cryptoxanthini	-0.2503	0.002	0.892940731	NA	NA	NA	Bacteria	Bacteria	intra
SBS I vs. Control	Arthrobacter sp. KBS0702	Pseudorhizobium flavum	-0.2675	0.004	0.892940731	NA	NA	NA	Bacteria	Bacteria	intra
SBS I vs. Control	Arthrobacter sp. NEB 688	Marinobacter sp. NP-4(2019)	-0.2528	0.001	0.892940731	NA	NA	NA	Bacteria	Bacteria	intra
SBS I vs. Control	Arthrobacter sp. NicSoilB8	Chryseobacterium sp. LJ668	-0.3362	0.001	0.892940731	NA	NA	NA	Bacteria	Bacteria	intra
SBS I vs. Control	Arthrobacter sp. PAMC25284	Leptospira sp. severe_002	0.285	0.003	0.892940731	NA	NA	NA	Bacteria	Bacteria	intra
SBS I vs. Control	Arthrobacter sp. PAMC25284	Streptococcus toyakuensis	0.3758	0.003	0.892940731	NA	NA	NA	Bacteria	Bacteria	intra
SBS I vs. Control	Arthrobacter sp. PM3	Gimesia chilikensis	-0.2587	0.004	0.892940731	NA	NA	NA	Bacteria	Bacteria	intra
SBS I vs. Control	Arthrobacter sp. PM3	Pseudorhizobium flavum	0.3084	0.001	0.892940731	NA	NA	NA	Bacteria	Bacteria	intra
SBS I vs. Control	Arthrobacter sp. StoSoilB5	Micromonospora sp. WMMD882	-0.2555	0.003	0.892940731	NA	NA	NA	Bacteria	Bacteria	intra
SBS I vs. Control	Arthrobacter sp. StoSoilB5	Simiaoa sunii	0.2972	0.001	0.892940731	NA	NA	NA	Bacteria	Bacteria	intra
SBS I vs. Control	Arthrobacter sp. zg-Y20	Rhizosphaericola mali	0.3237	0.003	0.892940731	NA	NA	NA	Bacteria	Bacteria	intra
SBS I vs. Control	Arthrobacter sp. zg-Y820	Klebsiella africana	0.2974	0.003	0.892940731	NA	NA	NA	Bacteria	Bacteria	intra
SBS I vs. Control	Arthrobacter sp. zg-Y820	Microvirga sp. VF16	0.3364	0.004	0.892940731	NA	NA	NA	Bacteria	Bacteria	intra
SBS I vs. Control	Arthrobacter sp. zg-Y919	Aureispira sp. CCB-E	-0.3584	0.002	0.892940731	NA	NA	NA	Bacteria	Bacteria	intra
SBS I vs. Control	Arthrobacter sp. zg-Y919	Neisseria yangbaofengii	-0.2884	0.001	0.892940731	NA	NA	NA	Bacteria	Bacteria	intra
SBS I vs. Control	Arthrobacter sp. zg-Y919	Tsukamurella pulmonis	-0.2571	0.003	0.892940731	NA	NA	NA	Bacteria	Bacteria	intra
SBS I vs. Control	Arthrobacter sulfonylureivorans	Treponema brennaborense	-0.2533	0.001	0.892940731	NA	NA	NA	Bacteria	Bacteria	intra
SBS I vs. Control	Arthroderma uncinatum	Bernardetia sp. ABR2-2B	-0.2674	0.003	0.892940731	NA	NA	NA	Bacteria	Bacteria	intra
SBS I vs. Control	Arthroderma uncinatum	Corynebacterium vitaeruminis	0.3287	0.002	0.892940731	NA	NA	NA	Bacteria	Bacteria	intra

SBS I vs. Control	Arthroderma uncinatum	Mycolicibacterium chubuense	-0.2795	0.001	0.892940731	NA	NA	NA	Bacteria	Bacteria	intra
SBS I vs. Control	Aspergillus aculeatus	Rathayibacter rathayi	0.2599	0.001	0.892940731	NA	NA	NA	Fungi	Bacteria	inter
SBS I vs. Control	Aspergillus tanneri	Duganella zoogloeoides	-0.2932	0.003	0.892940731	NA	NA	NA	Fungi	Bacteria	inter
SBS I vs. Control	Aspergillus tanneri	Klebsiella oxytoca	-0.3015	0.003	0.892940731	NA	NA	NA	Fungi	Bacteria	inter
SBS I vs. Control	Aspergillus tanneri	Rhizobium sp. NXC24	-0.2929	0.003	0.892940731	NA	NA	NA	Fungi	Bacteria	inter
SBS I vs. Control	Aspergillus tanneri	Sphingomonas sp. HDW15A	-0.2983	0.003	0.892940731	NA	NA	NA	Fungi	Bacteria	inter
SBS I vs. Control	Aspergillus thermomutatus	Aureibaculum algae	0.266	0.003	0.892940731	NA	NA	NA	Fungi	Bacteria	inter
SBS I vs. Control	Aspergillus thermomutatus	Mammaliicoccus vitulinus	-0.2829	0.001	0.892940731	NA	NA	NA	Fungi	Bacteria	inter
SBS I vs. Control	Aspergillus thermomutatus	Miniimonas sp. S16	-0.2628	0.004	0.892940731	NA	NA	NA	Fungi	Bacteria	inter
SBS I vs. Control	Aspergillus wentii	Leucobacter luti	-0.251	0.003	0.892940731	NA	NA	NA	Fungi	Bacteria	inter
SBS I vs. Control	Aspergillus wentii	Microbacterium fandaimingii	0.3009	0.004	0.892940731	NA	NA	NA	Fungi	Bacteria	inter
SBS I vs. Control	Aspergillus wentii	Photobacterium sp. TY1-4	0.2511	0.003	0.892940731	NA	NA	NA	Fungi	Bacteria	inter
SBS I vs. Control	Asticcacaulis excentricus	Clostridium thermarum	-0.2583	0.003	0.892940731	NA	NA	NA	Bacteria	Bacteria	intra
SBS I vs. Control	Asticcacaulis excentricus	Corynebacterium occultum	0.2585	0.004	0.892940731	NA	NA	NA	Bacteria	Bacteria	intra
SBS I vs. Control	Asticcacaulis excentricus	Halopseudomonas sp. SMJS2	0.3826	0.003	0.892940731	NA	NA	NA	Bacteria	Bacteria	intra
SBS I vs. Control	Atopobium sp. oral taxon 416	Corynebacterium suedekumii	-0.3423	0.003	0.892940731	NA	NA	NA	Bacteria	Bacteria	intra
SBS I vs. Control	Atopobium sp. oral taxon 416	Paenibacillus macerans	-0.3009	0.002	0.892940731	NA	NA	NA	Bacteria	Bacteria	intra
SBS I vs. Control	Atopobium sp. oral taxon 416	Thiorhodovibrio litoralis	-0.3188	0.001	0.892940731	NA	NA	NA	Bacteria	Bacteria	intra
SBS I vs. Control	Aurantimicrobium photophilum	Cedecea lapagei	-0.3882	0.002	0.892940731	NA	NA	NA	Bacteria	Bacteria	intra
SBS I vs. Control	Aurantimicrobium photophilum	Paraburkholderia tropica	-0.2565	0.002	0.892940731	NA	NA	NA	Bacteria	Bacteria	intra
SBS I vs.	Aurantimicrobium sp.	Kitasatospora sp. NBC_01246	-0.2972	0.002	0.892940731	NA	NA	NA	Bacteria	Bacteria	intra

Control	MWH-Uga1										
SBS I vs. Control	Auraticoccus monumenti	Leisingera sp. S132	-0.3172	0.001	0.892940731	NA	NA	NA	Bacteria	Bacteria	intra
SBS I vs. Control	Auraticoccus monumenti	Methylobacterium sp. C1	-0.2974	0.003	0.892940731	NA	NA	NA	Bacteria	Bacteria	intra
SBS I vs. Control	Aureibaculum algae	Flavobacterium pallidum	-0.2801	0.002	0.892940731	NA	NA	NA	Bacteria	Bacteria	intra
SBS I vs. Control	Aureibaculum algae	Marinobacter nanhaiticus	-0.3058	0.002	0.892940731	NA	NA	NA	Bacteria	Bacteria	intra
SBS I vs. Control	Aureibaculum algae	Natronorubrum sulfidifaciens	-0.3223	0.001	0.892940731	NA	NA	NA	Bacteria	Bacteria	intra
SBS I vs. Control	Aureibaculum algae	Ruminococcus champanellensis	-0.3456	0.003	0.892940731	NA	NA	NA	Bacteria	Bacteria	intra
SBS I vs. Control	Aureibaculum algae	Streptomyces durmitorensis	-0.2915	0.002	0.892940731	NA	NA	NA	Bacteria	Bacteria	intra
SBS I vs. Control	Aureibaculum algae	Thermococcus sp. GR4	-0.2877	0.003	0.892940731	NA	NA	NA	Bacteria	Bacteria	intra
SBS I vs. Control	Aureispira sp. CCB-E	Fusobacterium nucleatum	0.2574	0.002	0.892940731	NA	NA	NA	Bacteria	Bacteria	intra
SBS I vs. Control	Avibacterium paragallinarum	Rouxiella chamberiensis	-0.2844	0.003	0.892940731	NA	NA	NA	Bacteria	Bacteria	intra
SBS I vs. Control	Azorhizobium caulinodans	Flaviflexus salsibiostraticola	0.2612	0.001	0.892940731	NA	NA	NA	Bacteria	Bacteria	intra
SBS I vs. Control	Azorhizobium caulinodans	Kickxella alabastrina	0.3205	0.002	0.892940731	NA	NA	NA	Bacteria	Bacteria	intra
SBS I vs. Control	Azospirillum brasilense	Kocuria rhizophila	-0.2551	0.001	0.892940731	NA	NA	NA	Bacteria	Bacteria	intra
SBS I vs. Control	Azospirillum humicireducens	Micromonospora auratinigra	-0.2624	0.001	0.892940731	NA	NA	NA	Bacteria	Bacteria	intra
SBS I vs. Control	Azospirillum humicireducens	Niveibacterium microcysteis	-0.3233	0.003	0.892940731	NA	NA	NA	Bacteria	Bacteria	intra
SBS I vs. Control	Azospirillum humicireducens	Sporosarcina ureae	0.2752	0.003	0.892940731	NA	NA	NA	Bacteria	Bacteria	intra
SBS I vs. Control	Azospirillum oryzae	Pedobacter riviphilus	-0.3828	0.001	0.892940731	NA	NA	NA	Bacteria	Bacteria	intra
SBS I vs. Control	Azospirillum sp. TSH58	Nocardia sp. NBC_01730	-0.2629	0.003	0.892940731	NA	NA	NA	Bacteria	Bacteria	intra
SBS I vs. Control	Azospirillum sp. TSH58	Streptococcus australis	-0.3216	0.001	0.892940731	NA	NA	NA	Bacteria	Bacteria	intra
SBS I vs. Control	Azospirillum thiophilum	Limosilactobacillus vaginalis	-0.2539	0.003	0.892940731	NA	NA	NA	Bacteria	Bacteria	intra

SBS I vs. Control	Azospirillum thiophilum	Moraxella sp. FZFQ2102	-0.2827	0.001	0.892940731	NA	NA	NA	Bacteria	Bacteria	intra
SBS I vs. Control	Babjeviella inositovora	Methylobacterium sp. C1	0.3607	0.004	0.892940731	NA	NA	NA	Bacteria	Bacteria	intra
SBS I vs. Control	Babjeviella inositovora	Niallia circulans	0.3647	0.003	0.892940731	NA	NA	NA	Bacteria	Bacteria	intra
SBS I vs. Control	Babjeviella inositovora	Paenibacillus mucilaginosus	0.349	0.004	0.892940731	NA	NA	NA	Bacteria	Bacteria	intra
SBS I vs. Control	Babjeviella inositovora	Streptococcus sp. DTU_2020_1001019_1_SI_AUS_MUR_006	0.2919	0.003	0.892940731	NA	NA	NA	Bacteria	Bacteria	intra
SBS I vs. Control	Bacillus methanolicus	Desulfosarcina ovata	0.2571	0.001	0.892940731	NA	NA	NA	Bacteria	Bacteria	intra
SBS I vs. Control	Bacillus methanolicus	Halosegnis marinus	-0.4214	0.002	0.892940731	NA	NA	NA	Bacteria	Archaea	inter
SBS I vs. Control	Bacillus methanolicus	Maridesulfovibrio sp.	-0.2864	0.004	0.892940731	NA	NA	NA	Bacteria	Bacteria	intra
SBS I vs. Control	Bacillus methanolicus	Microbacterium lushaniae	-0.3308	0.001	0.892940731	NA	NA	NA	Bacteria	Bacteria	intra
SBS I vs. Control	Bacillus methanolicus	Pseudonocardia sp. DSM 110487	-0.2775	0.004	0.892940731	NA	NA	NA	Bacteria	Bacteria	intra
SBS I vs. Control	Bacillus mycoides	Stenotrophomonas sp. Pemsol	-0.3239	0.001	0.892940731	NA	NA	NA	Bacteria	Bacteria	intra
SBS I vs. Control	Bacillus sp. OxB-1	Brachybacterium avium	0.2948	0.003	0.892940731	NA	NA	NA	Bacteria	Bacteria	intra
SBS I vs. Control	Bacteroides finegoldii	Mycobacterium sp. JS623	-0.3352	0.004	0.892940731	NA	NA	NA	Bacteria	Bacteria	intra
SBS I vs. Control	Bacteroides finegoldii	Rothia sp. ZJ932	0.4386	0.004	0.892940731	NA	NA	NA	Bacteria	Bacteria	intra
SBS I vs. Control	Bacteroides finegoldii	Verminephrobacter eiseniae	-0.2615	0.002	0.892940731	NA	NA	NA	Bacteria	Bacteria	intra
SBS I vs. Control	Bacteroides finegoldii	Yersinia frederiksenii	-0.2644	0.003	0.892940731	NA	NA	NA	Bacteria	Bacteria	intra
SBS I vs. Control	Bacteroides ovatus	Simplicispira suum	-0.4915	0.001	0.892940731	NA	NA	NA	Bacteria	Bacteria	intra
SBS I vs. Control	Bacteroides sp. D2	Psychrobacter sp. WB2	0.2691	0.004	0.892940731	NA	NA	NA	Bacteria	Bacteria	intra
SBS I vs. Control	Barrientosiimonas endolithica	Chryseobacterium camelliae	-0.2514	0.002	0.892940731	NA	NA	NA	Bacteria	Bacteria	intra
SBS I vs. Control	Barrientosiimonas endolithica	Faecalibacterium prausnitzii	0.3256	0.002	0.892940731	NA	NA	NA	Bacteria	Bacteria	intra

SBS I vs. Control	Barrientosiimonas endolithica	Flavobacterium azooxidireducens	-0.3445	0.001	0.892940731	NA	NA	NA	Bacteria	Bacteria	intra
SBS I vs. Control	Bartonella alsatica	Chryseobacterium gleum	0.3632	0.0008	0.892940731	NA	NA	NA	Bacteria	Bacteria	intra
SBS I vs. Control	Bartonella henselae	Sulfitobacter alexandrii	0.29	0.002	0.892940731	NA	NA	NA	Bacteria	Bacteria	intra
SBS I vs. Control	Basilea psittacipulmonis	Ornithinimicrobium cryptoxanthini	-0.3009	0.001	0.892940731	NA	NA	NA	Bacteria	Bacteria	intra
SBS I vs. Control	Basilea psittacipulmonis	[Ruminococcus] torques	-0.2879	0.003	0.892940731	NA	NA	NA	Bacteria	Bacteria	intra
SBS I vs. Control	Bdellovibrio sp. 22V	Leptotrichia wadei	-0.2586	0.003	0.892940731	NA	NA	NA	Bacteria	Bacteria	intra
SBS I vs. Control	Beauveria bassiana	Qiania dongpingensis	0.2722	0.001	0.892940731	NA	NA	NA	Fungi	Bacteria	inter
SBS I vs. Control	Bernardetia sp. ABR2-2B	Curtobacterium sp. TC1	-0.3023	0.002	0.892940731	NA	NA	NA	Bacteria	Bacteria	intra
SBS I vs. Control	Bernardetia sp. ABR2-2B	Halomonas sp. XH26	0.2555	0.004	0.892940731	NA	NA	NA	Bacteria	Bacteria	intra
SBS I vs. Control	Betabaculovirus spliturae	Gluconobacter oxydans	-0.2963	0.003	0.892940731	NA	NA	NA	Bacteria	Bacteria	intra
SBS I vs. Control	Betabaculovirus spliturae	Marinobacter nanhaiticus	0.2905	0.001	0.892940731	NA	NA	NA	Bacteria	Bacteria	intra
SBS I vs. Control	Betabaculovirus spliturae	Paenibacillus tritici	0.3484	0.002	0.892940731	NA	NA	NA	Bacteria	Bacteria	intra
SBS I vs. Control	Bifidobacterium adolescentis	Desulfovibrio desulfuricans	-0.2657	0.001	0.892940731	NA	NA	NA	Bacteria	Bacteria	intra
SBS I vs. Control	Bifidobacterium adolescentis	Xylanimonas protaetiae	-0.2574	0.003	0.892940731	NA	NA	NA	Bacteria	Bacteria	intra
SBS I vs. Control	Bifidobacterium animalis	Ornithinimicrobium cryptoxanthini	0.2599	0.001	0.892940731	NA	NA	NA	Bacteria	Bacteria	intra
SBS I vs. Control	Bifidobacterium catenulatum	Corynebacterium incognita	0.2534	0.003	0.892940731	NA	NA	NA	Bacteria	Bacteria	intra
SBS I vs. Control	Bifidobacterium catenulatum	Methanobrevibacter sp.	-0.3227	0.002	0.892940731	NA	NA	NA	Bacteria	Bacteria	intra
SBS I vs. Control	Blastochloris tepida	Mesorhizobium sp. M4B.F.Ca.ET.058.02.1.1	-0.258	0.002	0.892940731	NA	NA	NA	Bacteria	Bacteria	intra
SBS I vs. Control	Blastomonas sp.	Lancefieldella sp. Marseille-Q7238	-0.2623	0.002	0.892940731	NA	NA	NA	Bacteria	Bacteria	intra
SBS I vs. Control	Blastomonas sp.	Microlunatus sagamiharensis	0.2666	0.001	0.892940731	NA	NA	NA	Bacteria	Bacteria	intra
SBS I vs.	Blattabacterium cuenoti	Thermococcus sp. MAR1	-0.2874	0.004	0.892940731	NA	NA	NA	Bacteria	Bacteria	intra

Control												
SBS I vs. Control	Blautia argi	Rubrobacter radiotolerans	-0.2859	0.002	0.892940731	NA	NA	NA	Bacteria	Bacteria	intra
SBS I vs. Control	Blautia coccoides	Caballeronia sp. Lep1P3	-0.2733	0.004	0.892940731	NA	NA	NA	Bacteria	Bacteria	intra
SBS I vs. Control	Blautia coccoides	Lachnoanaerobaculum umeaense	-0.3222	0.003	0.892940731	NA	NA	NA	Bacteria	Bacteria	intra
SBS I vs. Control	Blautia hansenii	Herbiconiux sp. L3-i23	0.2585	0.002	0.892940731	NA	NA	NA	Bacteria	Bacteria	intra
SBS I vs. Control	Blautia hansenii	Leucobacter muris	0.3184	0.002	0.892940731	NA	NA	NA	Bacteria	Bacteria	intra
SBS I vs. Control	Blautia hydrogenotrophica	Devosia sp.	-0.2818	0.003	0.892940731	NA	NA	NA	Bacteria	Bacteria	intra
SBS I vs. Control	Blautia luti	Kosakonia cowanii	0.2552	0.001	0.892940731	NA	NA	NA	Bacteria	Bacteria	intra
SBS I vs. Control	Blautia obeum	Paenibacillus macerans	-0.2505	0.004	0.892940731	NA	NA	NA	Bacteria	Bacteria	intra
SBS I vs. Control	Blautia producta	Cohnella candidum	-0.2678	0.002	0.892940731	NA	NA	NA	Bacteria	Bacteria	intra
SBS I vs. Control	Blautia producta	Tsuneonella sp. CC-YZS046	-0.2645	0.001	0.892940731	NA	NA	NA	Bacteria	Bacteria	intra
SBS I vs. Control	Blautia pseudococcoides	Mycobacterium decipiens	0.2603	0.003	0.892940731	NA	NA	NA	Bacteria	Bacteria	intra
SBS I vs. Control	Blautia sp. SC05B48	Brachybacterium huguangmaarense	-0.3377	0.002	0.892940731	NA	NA	NA	Bacteria	Bacteria	intra
SBS I vs. Control	Blautia sp. SC05B48	Erythrobacter sp.	0.2563	0.001	0.892940731	NA	NA	NA	Bacteria	Bacteria	intra
SBS I vs. Control	Bordetella genomosp. 9	Clavibacter sepedonicus	0.3076	0.002	0.892940731	NA	NA	NA	Bacteria	Bacteria	intra
SBS I vs. Control	Bordetella trematum	Cladophialophora carrionii	-0.3015	0.003	0.892940731	NA	NA	NA	Bacteria	Fungi	inter
SBS I vs. Control	Bordetella trematum	Megamonas hypermegale	-0.2542	0.004	0.892940731	NA	NA	NA	Bacteria	Bacteria	intra
SBS I vs. Control	Botrytis cinerea	Flavobacterium sp. KBS0721	0.2756	0.004	0.892940731	NA	NA	NA	Fungi	Bacteria	inter
SBS I vs. Control	Botrytis cinerea	Gleimia europaea	0.3772	0.004	0.892940731	NA	NA	NA	Fungi	Bacteria	inter
SBS I vs. Control	Botrytis cinerea	Streptomyces sp. NBC_01304	-0.2931	0.004	0.892940731	NA	NA	NA	Fungi	Bacteria	inter
SBS I vs. Control	Boudabousia tangfeifanii	Ideonella dechloratans	-0.2706	0.001	0.892940731	NA	NA	NA	Bacteria	Bacteria	intra

SBS I vs. Control	Boudabousia tangfeifanii	Mycobacterium sp. ELW1	-0.2731	0.004	0.892940731	NA	NA	NA	Bacteria	Bacteria	intra
SBS I vs. Control	Boudabousia tangfeifanii	Rhodococcus triatomae	-0.2574	0.002	0.892940731	NA	NA	NA	Bacteria	Bacteria	intra
SBS I vs. Control	Brachybacterium avium	Parvimonas micra	0.3568	0.003	0.892940731	NA	NA	NA	Bacteria	Bacteria	intra
SBS I vs. Control	Brachybacterium avium	Salipaludibacillus sp. LMS25	0.259	0.001	0.892940731	NA	NA	NA	Bacteria	Bacteria	intra
SBS I vs. Control	Brachybacterium ginsengisoli	Flintibacter sp. KGMB00164	0.3894	0.003	0.892940731	NA	NA	NA	Bacteria	Bacteria	intra
SBS I vs. Control	Brachybacterium ginsengisoli	Mycolicibacterium fortuitum	-0.2906	0.001	0.892940731	NA	NA	NA	Bacteria	Bacteria	intra
SBS I vs. Control	Brachybacterium huguangmaarense	Luteimonas sp. YGD11-2	-0.3575	0.004	0.892940731	NA	NA	NA	Bacteria	Bacteria	intra
SBS I vs. Control	Brachybacterium huguangmaarense	Mucilaginibacter celer	-0.298	0.001	0.892940731	NA	NA	NA	Bacteria	Bacteria	intra
SBS I vs. Control	Brachybacterium sp. NBEC-018	Rathayibacter toxicus	-0.3105	0.001	0.892940731	NA	NA	NA	Bacteria	Bacteria	intra
SBS I vs. Control	Brachybacterium sp. NBEC-018	Sphingobium sp. AP49	0.2734	0.001	0.892940731	NA	NA	NA	Bacteria	Bacteria	intra
SBS I vs. Control	Brachybacterium vulturis	Clostridium botulinum	0.2986	0.004	0.892940731	NA	NA	NA	Bacteria	Bacteria	intra
SBS I vs. Control	Brachybacterium vulturis	Streptococcus toyakuensis	-0.2778	0.002	0.892940731	NA	NA	NA	Bacteria	Bacteria	intra
SBS I vs. Control	Brachyspira hyodysenteriae	Diaphorobacter sp. HDW4B	-0.2842	0.002	0.892940731	NA	NA	NA	Bacteria	Bacteria	intra
SBS I vs. Control	Brachyspira hyodysenteriae	Lichenihabitans psoromatis	0.2604	0.002	0.892940731	NA	NA	NA	Bacteria	Bacteria	intra
SBS I vs. Control	Brachyspira hyodysenteriae	Streptomyces antibioticus	0.2934	0.002	0.892940731	NA	NA	NA	Bacteria	Bacteria	intra
SBS I vs. Control	Bradyrhizobium guangxiense	Colletotrichum karsti	0.2687	0.004	0.892940731	NA	NA	NA	Bacteria	Fungi	inter
SBS I vs. Control	Bradyrhizobium guangxiense	Metabacillus sediminilitoris	-0.2946	0.002	0.892940731	NA	NA	NA	Bacteria	Bacteria	intra
SBS I vs. Control	Bradyrhizobium guangxiense	Paraoerskovia marina	-0.3184	0.001	0.892940731	NA	NA	NA	Bacteria	Bacteria	intra
SBS I vs. Control	Bradyrhizobium sp. 195	Eubacterium ventriosum	-0.278	0.004	0.892940731	NA	NA	NA	Bacteria	Bacteria	intra
SBS I vs. Control	Bradyrhizobium sp. 195	Halobacteroides halobius	0.3019	0.001	0.892940731	NA	NA	NA	Bacteria	Bacteria	intra
SBS I vs.	Bradyrhizobium sp. 195	Methylobacter sp. YRD-M1	0.2751	0.003	0.892940731	NA	NA	NA	Bacteria	Bacteria	intra

Control											
SBS I vs. Control	Bradyrhizobium sp. 195	Thermomonospora curvata	0.2842	0.002	0.892940731	NA	NA	NA	Bacteria	Bacteria	intra
SBS I vs. Control	Bradyrhizobium sp. CCBAU 53340	Comamonas piscis	0.3109	0.004	0.892940731	NA	NA	NA	Bacteria	Bacteria	intra
SBS I vs. Control	Bradyrhizobium sp. CCBAU 53340	Faecalibacterium prausnitzii	-0.3727	0.001	0.892940731	NA	NA	NA	Bacteria	Bacteria	intra
SBS I vs. Control	Bradyrhizobium sp. CCBAU 53340	Microbacterium sp. PM5	-0.2845	0.003	0.892940731	NA	NA	NA	Bacteria	Bacteria	intra
SBS I vs. Control	Bradyrhizobium sp. TM102	Limosilactobacillus oris	0.3391	0.001	0.892940731	NA	NA	NA	Bacteria	Bacteria	intra
SBS I vs. Control	Bradyrhizobium sp. TM102	Stenotrophomonas acidaminiphila	0.2586	0.003	0.892940731	NA	NA	NA	Bacteria	Bacteria	intra
SBS I vs. Control	Brevibacillus ruminantium	Citricoccus muralis	0.3005	0.004	0.892940731	NA	NA	NA	Bacteria	Bacteria	intra
SBS I vs. Control	Brevibacillus ruminantium	Corynebacterium aquilae	0.2772	0.003	0.892940731	NA	NA	NA	Bacteria	Bacteria	intra
SBS I vs. Control	Brevibacillus ruminantium	Eubacterium sp. MSJ-33	-0.2651	0.003	0.892940731	NA	NA	NA	Bacteria	Bacteria	intra
SBS I vs. Control	Brevibacillus sp. WF146	Microbacterium sp. M28	-0.2544	0.001	0.892940731	NA	NA	NA	Bacteria	Bacteria	intra
SBS I vs. Control	Brevibacillus sp. WF146	Mycobacterium kansasii	0.2547	0.004	0.892940731	NA	NA	NA	Bacteria	Bacteria	intra
SBS I vs. Control	Brevibacterium linens	Clostridium botulinum	0.3842	0.003	0.892940731	NA	NA	NA	Bacteria	Bacteria	intra
SBS I vs. Control	Brevibacterium linens	Simiaoa sunii	0.3102	0.002	0.892940731	NA	NA	NA	Bacteria	Bacteria	intra
SBS I vs. Control	Brevibacterium luteolum	Microbacterium oxydans	0.2715	0.002	0.892940731	NA	NA	NA	Bacteria	Bacteria	intra
SBS I vs. Control	Brevibacterium luteolum	Runella sp. SP2	-0.2506	0.001	0.892940731	NA	NA	NA	Bacteria	Bacteria	intra
SBS I vs. Control	Brevibacterium zhoupengii	Pseudarthrobacter sp. NIBRBAC000502772	0.3001	0.001	0.892940731	NA	NA	NA	Bacteria	Bacteria	intra
SBS I vs. Control	Brevibacterium zhoupengii	Rhodanobacter sp. AS-Z3	0.2671	0.001	0.892940731	NA	NA	NA	Bacteria	Bacteria	intra
SBS I vs. Control	Brevundimonas albigilva	Cellulomonas wangsupingiae	-0.2667	0.004	0.892940731	NA	NA	NA	Bacteria	Bacteria	intra
SBS I vs. Control	Brevundimonas albigilva	Hydrogenophaga sp. BPS33	-0.3262	0.002	0.892940731	NA	NA	NA	Bacteria	Bacteria	intra
SBS I vs. Control	Brevundimonas diminuta	Treponema brennaborense	0.2749	0.004	0.892940731	NA	NA	NA	Bacteria	Bacteria	intra

SBS I vs. Control	Brevundimonas sp. MF30-B	Streptomyces sp. NBC_01280	0.3267	0.001	0.892940731	NA	NA	NA	Bacteria	Bacteria	intra
SBS I vs. Control	Brevundimonas sp. NIBR10	Phaeoacremonium minimum	0.2764	0.004	0.892940731	NA	NA	NA	Bacteria	Bacteria	intra
SBS I vs. Control	Brevundimonas sp. NIBR10	Streptomyces tubbatahanensis	0.2651	0.003	0.892940731	NA	NA	NA	Bacteria	Bacteria	intra
SBS I vs. Control	Brevundimonas sp. NIBR11	uncultured Acetobacteroides sp	0.3605	0.001	0.892940731	NA	NA	NA	Bacteria	Bacteria	intra
SBS I vs. Control	Brevundimonas sp. SL130	Halovivax ruber	-0.3752	0.002	0.892940731	NA	NA	NA	Bacteria	Bacteria	intra
SBS I vs. Control	Brucella intermedia	Umbelopsis ramanniana	-0.2679	0.004	0.892940731	NA	NA	NA	Bacteria	Bacteria	intra
SBS I vs. Control	Brucella pseudintermedia	Streptococcus sp. 29896	-0.2638	0.004	0.892940731	NA	NA	NA	Bacteria	Bacteria	intra
SBS I vs. Control	Buchnera aphidicola	Leisingera thetidis	0.4174	0.001	0.892940731	NA	NA	NA	Bacteria	Bacteria	intra
SBS I vs. Control	Buchnera aphidicola	Leptotrichia sp. oral taxon 218	0.3253	0.001	0.892940731	NA	NA	NA	Bacteria	Bacteria	intra
SBS I vs. Control	Buchnera aphidicola	Shinella sp. XGS7	-0.2774	0.003	0.892940731	NA	NA	NA	Bacteria	Bacteria	intra
SBS I vs. Control	Buchnera aphidicola	Zunongwangia sp. HGR-M22	-0.3039	0.003	0.892940731	NA	NA	NA	Bacteria	Bacteria	intra
SBS I vs. Control	Burkholderia aenigmatica	Sulfitobacter sp. S190	-0.302	0.003	0.892940731	NA	NA	NA	Bacteria	Bacteria	intra
SBS I vs. Control	Burkholderia contaminans	Streptococcus sp. D7B5	-0.2689	0.002	0.892940731	NA	NA	NA	Bacteria	Bacteria	intra
SBS I vs. Control	Burkholderia glumae	Kocuria rosea	-0.2736	0.002	0.892940731	NA	NA	NA	Bacteria	Bacteria	intra
SBS I vs. Control	Burkholderia glumae	Thermococcus sp. MAR1	-0.2663	0.003	0.892940731	NA	NA	NA	Bacteria	Bacteria	intra
SBS I vs. Control	Burkholderia latens	Erythrobacter sp. SCSIO 43205	-0.2843	0.004	0.892940731	NA	NA	NA	Bacteria	Bacteria	intra
SBS I vs. Control	Burkholderia latens	Sphingobacterium multivorum	0.3177	0.003	0.892940731	NA	NA	NA	Bacteria	Bacteria	intra
SBS I vs. Control	Buttiauxella agrestis	Micromonospora sp. WMMA1998	-0.2509	0.004	0.892940731	NA	NA	NA	Bacteria	Bacteria	intra
SBS I vs. Control	Buttiauxella agrestis	Pseudomonas sp. MPC6	0.3022	0.001	0.892940731	NA	NA	NA	Bacteria	Bacteria	intra
SBS I vs. Control	Buttiauxella sp. 3AFRM03	Paraburkholderia phenoliruptrix	0.2555	0.003	0.892940731	NA	NA	NA	Bacteria	Bacteria	intra
SBS I vs.	Caballeronia sp. Lep1P3	Methylobacterium organophilum	0.252	0.002	0.892940731	NA	NA	NA	Bacteria	Bacteria	intra

Control											
SBS I vs. Control	Caballeronia sp. Lep1P3	Methylococcus geothermalis	-0.2538	0.004	0.892940731	NA	NA	NA	Bacteria	Bacteria	intra
SBS I vs. Control	Caballeronia sp. Lep1P3	Pseudomonas trivialis	0.3294	0.001	0.892940731	NA	NA	NA	Bacteria	Bacteria	intra
SBS I vs. Control	Caldicellulosiruptor hydrothermalis	Lactobacillus delbrueckii	-0.2585	0.003	0.892940731	NA	NA	NA	Bacteria	Bacteria	intra
SBS I vs. Control	Caldicellulosiruptor hydrothermalis	Leclercia sp. W6	-0.3396	0.002	0.892940731	NA	NA	NA	Bacteria	Bacteria	intra
SBS I vs. Control	Campylobacter coli	Geomonas oryzisoli	-0.331	0.004	0.892940731	NA	NA	NA	Bacteria	Bacteria	intra
SBS I vs. Control	Campylobacter coli	Klebsiella oxytoca	-0.3857	0.002	0.892940731	NA	NA	NA	Bacteria	Bacteria	intra
SBS I vs. Control	Campylobacter coli	Lacrimispora saccharolytica	0.2754	0.003	0.892940731	NA	NA	NA	Bacteria	Bacteria	intra
SBS I vs. Control	Campylobacter coli	Neobacillus sp. OS1-2	-0.3378	0.002	0.892940731	NA	NA	NA	Bacteria	Bacteria	intra
SBS I vs. Control	Candida oxycetoniae	Rhizobium leguminosarum	0.2686	0.004	0.892940731	NA	NA	NA	Fungi	Bacteria	inter
SBS I vs. Control	Candida tropicalis	Candidatus Minimicrobia vallesae	-0.4132	0.001	0.892940731	NA	NA	NA	Fungi	Bacteria	inter
SBS I vs. Control	Candida tropicalis	Halovivax ruber	0.2574	0.004	0.892940731	NA	NA	NA	Fungi	Bacteria	inter
SBS I vs. Control	Candidatus Aquiluna sp. UB-MaderosW2red	Cellulomonas taurus	-0.2996	0.004	0.892940731	NA	NA	NA	Bacteria	Bacteria	intra
SBS I vs. Control	Candidatus Desulforudis audaxviator	Flavobacterium sp. GSB-24	-0.2671	0.004	0.892940731	NA	NA	NA	Bacteria	Bacteria	intra
SBS I vs. Control	Candidatus Desulforudis audaxviator	Microbacterium sp. PM5	-0.2855	0.001	0.892940731	NA	NA	NA	Bacteria	Bacteria	intra
SBS I vs. Control	Candidatus Minimicrobia sp. QA0096	Corynebacterium pseudokroppenstedtii	-0.2886	0.004	0.892940731	NA	NA	NA	Bacteria	Bacteria	intra
SBS I vs. Control	Candidatus Minimicrobia sp. QA0096	Paracoccus jeotgali	-0.2781	0.001	0.892940731	NA	NA	NA	Bacteria	Bacteria	intra
SBS I vs. Control	Candidatus Minimicrobia sp. QA0096	Streptococcus gordonii	0.2765	0.004	0.892940731	NA	NA	NA	Bacteria	Bacteria	intra
SBS I vs. Control	Candidatus Minimicrobia sp. QA0096	Streptomyces sp. NBC_01431	0.2623	0.004	0.892940731	NA	NA	NA	Bacteria	Bacteria	intra
SBS I vs. Control	Candidatus Minimicrobia sp. QA0096	uncultured Acetobacteroides sp	0.2772	0.001	0.892940731	NA	NA	NA	Bacteria	Bacteria	intra
SBS I vs. Control	Candidatus Minimicrobia vallesae	Nakamurella antarctica	0.3639	0.004	0.892940731	NA	NA	NA	Bacteria	Bacteria	intra

SBS I vs. Control	Candidatus Nanopelagicus limnes	Fusobacterium nucleatum	-0.2858	0.003	0.892940731	NA	NA	NA	Bacteria	Bacteria	intra
SBS I vs. Control	Candidatus Nanopelagicus limnes	Glutamicibacter nicotianae	0.3159	0.003	0.892940731	NA	NA	NA	Bacteria	Bacteria	intra
SBS I vs. Control	Candidatus Nanopelagicus limnes	Marinobacter sp. NP-4(2019)	-0.3081	0.003	0.892940731	NA	NA	NA	Bacteria	Bacteria	intra
SBS I vs. Control	Candidatus Nanopelagicus limnes	Mesorhizobium sp. AR07	0.2683	0.004	0.892940731	NA	NA	NA	Bacteria	Bacteria	intra
SBS I vs. Control	Candidatus Pelagibacter sp. FZCC0015	Diaphorobacter sp. HDW4B	-0.273	0.002	0.892940731	NA	NA	NA	Bacteria	Bacteria	intra
SBS I vs. Control	Candidatus Pelagibacter sp. FZCC0015	Micromonospora sp. NBC_00330	-0.2586	0.003	0.892940731	NA	NA	NA	Bacteria	Bacteria	intra
SBS I vs. Control	Candidatus Pelagibacter sp. FZCC0015	Streptomyces sp. NBC_01727	0.2513	0.001	0.892940731	NA	NA	NA	Bacteria	Bacteria	intra
SBS I vs. Control	Candidatus Phaeomarinobacter ectocarpi	Streptosporangium roseum	0.4059	0.001	0.892940731	NA	NA	NA	Bacteria	Bacteria	intra
SBS I vs. Control	Candidatus Planktophila dulcis	Klebsiella pneumoniae	0.2823	0.004	0.892940731	NA	NA	NA	Bacteria	Bacteria	intra
SBS I vs. Control	Candidatus Planktophila dulcis	Streptomyces flavofungini	-0.2774	0.004	0.892940731	NA	NA	NA	Bacteria	Bacteria	intra
SBS I vs. Control	Candidatus Planktophila dulcis	Streptomyces jietaisiensis	0.3691	0.002	0.892940731	NA	NA	NA	Bacteria	Bacteria	intra
SBS I vs. Control	Candidatus Sulfidibacterium hydrothermale	Desulfobulbus propionicus	0.2581	0.001	0.892940731	NA	NA	NA	Bacteria	Bacteria	intra
SBS I vs. Control	Candidatus Sulfidibacterium hydrothermale	Eubacterium ventriosum	-0.283	0.002	0.892940731	NA	NA	NA	Bacteria	Bacteria	intra
SBS I vs. Control	Candidatus Sulfidibacterium hydrothermale	Mycobacterium sp. MS1601	0.3639	0.002	0.892940731	NA	NA	NA	Bacteria	Bacteria	intra
SBS I vs. Control	Candidatus Thiodictyon syntrophicum	uncultured Cohaesibacter sp	0.3283	0.004	0.892940731	NA	NA	NA	Bacteria	Bacteria	intra
SBS I vs. Control	Capronia epimyces	Methylocystis bryophila	-0.2638	0.001	0.892940731	NA	NA	NA	Fungi	Bacteria	inter
SBS I vs. Control	Capronia epimyces	Mycolicibacterium rutilum	0.4626	0.001	0.892940731	NA	NA	NA	Fungi	Bacteria	inter
SBS I vs. Control	Capronia epimyces	Sphingorhabdus sp. SMR4y	0.2835	0.001	0.892940731	NA	NA	NA	Fungi	Bacteria	inter
SBS I vs. Control	Catellatospora sp. IY07-71	Mesorhizobium sp. L-2-11	0.2999	0.002	0.892940731	NA	NA	NA	Bacteria	Bacteria	intra
SBS I vs. Control	Catellatospora sp. IY07-71	Mixta hanseatica	0.2625	0.002	0.892940731	NA	NA	NA	Bacteria	Bacteria	intra
SBS I vs.	Catellatospora sp. IY07-71	Paraburkholderia edwinii	0.268	0.003	0.892940731	NA	NA	NA	Bacteria	Bacteria	intra

Control											
SBS I vs. Control	Catenibacterium sp. co_0103	Ruminococcus bicirculans (ex Wegman et al. 2014)	0.3145	0.001	0.892940731	NA	NA	NA	Bacteria	Bacteria	intra
SBS I vs. Control	Caulobacter rhizosphaerae	Metschnikowia bicuspidata	-0.3625	0.004	0.892940731	NA	NA	NA	Bacteria	Bacteria	intra
SBS I vs. Control	Caulobacter rhizosphaerae	Microlunatus soli	0.296	0.004	0.892940731	NA	NA	NA	Bacteria	Bacteria	intra
SBS I vs. Control	Caulobacter rhizosphaerae	Miltoncostaea oceani	0.2978	0.001	0.892940731	NA	NA	NA	Bacteria	Bacteria	intra
SBS I vs. Control	Caulobacter rhizosphaerae	Spirosoma endbachense	0.334	0.004	0.892940731	NA	NA	NA	Bacteria	Bacteria	intra
SBS I vs. Control	Caulobacter sp. FWC26	Zymomonas mobilis	-0.3185	0.003	0.892940731	NA	NA	NA	Bacteria	Bacteria	intra
SBS I vs. Control	Cedecea lapagei	Isoptericola variabilis	0.2517	0.004	0.892940731	NA	NA	NA	Bacteria	Bacteria	intra
SBS I vs. Control	Cedecea lapagei	Sporisorium graminicola	-0.3365	0.001	0.892940731	NA	NA	NA	Bacteria	Bacteria	intra
SBS I vs. Control	Cedecea neteri	Laceyella sacchari	0.3111	0.001	0.892940731	NA	NA	NA	Bacteria	Bacteria	intra
SBS I vs. Control	Cedecea neteri	Propionibacterium freudenreichii	0.2686	0.002	0.892940731	NA	NA	NA	Bacteria	Bacteria	intra
SBS I vs. Control	Cedecea sp. FDAARGOS_727	Haemophilus parainfluenzae	-0.3141	0.001	0.892940731	NA	NA	NA	Bacteria	Bacteria	intra
SBS I vs. Control	Cedecea sp. FDAARGOS_727	Stenotrophomonas sp. BIO128-Bstrain	-0.2628	0.001	0.892940731	NA	NA	NA	Bacteria	Bacteria	intra
SBS I vs. Control	Cedecea sp. FDAARGOS_727	Streptococcus rubneri	-0.3436	0.001	0.892940731	NA	NA	NA	Bacteria	Bacteria	intra
SBS I vs. Control	Cellulomonas dongxiuzhuiae	Cohnella candidum	-0.2778	0.004	0.892940731	NA	NA	NA	Bacteria	Bacteria	intra
SBS I vs. Control	Cellulomonas dongxiuzhuiae	Roseivivax marinus	0.2539	0.004	0.892940731	NA	NA	NA	Bacteria	Bacteria	intra
SBS I vs. Control	Cellulomonas flavigena	Kineothrix sp. MB12-C1	-0.2749	0.003	0.892940731	NA	NA	NA	Bacteria	Bacteria	intra
SBS I vs. Control	Cellulomonas flavigena	Microbacterium sp. ABRD28	0.2824	0.004	0.892940731	NA	NA	NA	Bacteria	Bacteria	intra
SBS I vs. Control	Cellulomonas flavigena	Streptococcus mitis	-0.3359	0.002	0.892940731	NA	NA	NA	Bacteria	Bacteria	intra
SBS I vs. Control	Cellulomonas flavigena	Stutzerimonas stutzeri	-0.2694	0.001	0.892940731	NA	NA	NA	Bacteria	Bacteria	intra
SBS I vs. Control	Cellulomonas flavigena	Thermospira aquatica	-0.2634	0.001	0.892940731	NA	NA	NA	Bacteria	Bacteria	intra

SBS I vs. Control	Cellulomonas sp. NTE-D12	Erythrobacter litoralis	-0.3416	0.001	0.892940731	NA	NA	NA	Bacteria	Bacteria	intra
SBS I vs. Control	Cellulomonas sp. NTE-D12	Flavobacterium sp. IMCC34852	-0.2531	0.003	0.892940731	NA	NA	NA	Bacteria	Bacteria	intra
SBS I vs. Control	Cellulomonas sp. Y8	Leptotrichia sp. oral taxon 218	-0.3456	0.002	0.892940731	NA	NA	NA	Bacteria	Bacteria	intra
SBS I vs. Control	Cellulomonas sp. Y8	Marispirochaeta sp.	0.2778	0.004	0.892940731	NA	NA	NA	Bacteria	Bacteria	intra
SBS I vs. Control	Cellulomonas sp. Y8	Microbacterium sp. ET2	-0.324	0.004	0.892940731	NA	NA	NA	Bacteria	Bacteria	intra
SBS I vs. Control	Cellulomonas sp. Y8	Sphingomonas sp. KRR8	0.4345	0.003	0.892940731	NA	NA	NA	Bacteria	Bacteria	intra
SBS I vs. Control	Cellulomonas sp. Y8	Streptococcus merionis	-0.2617	0.002	0.892940731	NA	NA	NA	Bacteria	Bacteria	intra
SBS I vs. Control	Cellulomonas taurus	Cellulomonas wangsupingiae	0.2668	0.004	0.892940731	NA	NA	NA	Bacteria	Bacteria	intra
SBS I vs. Control	Cellulomonas taurus	Herbiconiux sp. L3-i23	-0.2592	0.001	0.892940731	NA	NA	NA	Bacteria	Bacteria	intra
SBS I vs. Control	Cellulomonas wangleii	Rubrivivax gelatinosus	0.3708	0.003	0.892940731	NA	NA	NA	Bacteria	Bacteria	intra
SBS I vs. Control	Cellulomonas wangleii	Streptomyces antibioticus	0.3072	0.002	0.892940731	NA	NA	NA	Bacteria	Bacteria	intra
SBS I vs. Control	Cellulomonas wangleii	Streptomyces asoensis	0.3015	0.003	0.892940731	NA	NA	NA	Bacteria	Bacteria	intra
SBS I vs. Control	Cellulomonas wangsupingiae	Thiomonas sp. X19	-0.2509	0.004	0.892940731	NA	NA	NA	Bacteria	Bacteria	intra
SBS I vs. Control	Cellulomonas xiejunii	Streptococcus toyakuensis	-0.263	0.001	0.892940731	NA	NA	NA	Bacteria	Bacteria	intra
SBS I vs. Control	Cellulophaga omnivescoria	Microlunatus sagamiharensis	-0.2701	0.002	0.892940731	NA	NA	NA	Bacteria	Bacteria	intra
SBS I vs. Control	Chenggangzhangella methanolivorans	Collinsella stercoris	-0.2934	0.004	0.892940731	NA	NA	NA	Bacteria	Bacteria	intra
SBS I vs. Control	Chenggangzhangella methanolivorans	Flavobacterium nitratireducens	-0.3488	0.001	0.892940731	NA	NA	NA	Bacteria	Bacteria	intra
SBS I vs. Control	Chenggangzhangella methanolivorans	Roseivirga spongicola	-0.4103	0.003	0.892940731	NA	NA	NA	Bacteria	Bacteria	intra
SBS I vs. Control	Chitinophaga agri	Flavobacterium sp. N2038	0.2568	0.002	0.892940731	NA	NA	NA	Bacteria	Bacteria	intra
SBS I vs. Control	Chitinophaga agri	Microbacterium sp. YJN-G	0.2554	0.001	0.892940731	NA	NA	NA	Bacteria	Bacteria	intra
SBS I vs.	Chitinophaga agri	Sphingobium sp. RAC03	-0.2863	0.004	0.892940731	NA	NA	NA	Bacteria	Bacteria	intra

Control											
SBS I vs. Control	Chitinophaga oryzae	Rhodococcus pseudokoreensis	-0.3124	0.003	0.892940731	NA	NA	NA	Bacteria	Bacteria	intra
SBS I vs. Control	Chitinophaga oryzae	Sinomonas sp. R1AF57	-0.2785	0.003	0.892940731	NA	NA	NA	Bacteria	Bacteria	intra
SBS I vs. Control	Chondrinema litorale	Neisseria sp. oral taxon 014	-0.2681	0.001	0.892940731	NA	NA	NA	Bacteria	Bacteria	intra
SBS I vs. Control	Chordicoccus furentiruminis	Nocardioides cynanchi	0.2869	0.002	0.892940731	NA	NA	NA	Bacteria	Bacteria	intra
SBS I vs. Control	Chordicoccus furentiruminis	Sphingomonas sp. HDW15A	0.3173	0.001	0.892940731	NA	NA	NA	Bacteria	Bacteria	intra
SBS I vs. Control	Chromobacterium vaccinii	Microbacterium sp. H1-D42	-0.2793	0.004	0.892940731	NA	NA	NA	Bacteria	Bacteria	intra
SBS I vs. Control	Chromobacterium vaccinii	Pararhodospirillum photometricum	0.2592	0.001	0.892940731	NA	NA	NA	Bacteria	Bacteria	intra
SBS I vs. Control	Chromobacterium violaceum	Desulfoscipio gibsoniae	0.2653	0.001	0.892940731	NA	NA	NA	Bacteria	Bacteria	intra
SBS I vs. Control	Chryseobacterium camelliae	Streptomyces jietaisiensis	0.4204	0.004	0.892940731	NA	NA	NA	Bacteria	Bacteria	intra
SBS I vs. Control	Chryseobacterium sp. LJ668	Mycobacteroides chelonae	0.2686	0.001	0.892940731	NA	NA	NA	Bacteria	Bacteria	intra
SBS I vs. Control	Chryseobacterium sp. T16E-39	Neisseria sp. Marseille-Q6792	-0.3529	0.001	0.892940731	NA	NA	NA	Bacteria	Bacteria	intra
SBS I vs. Control	Citricoccus sp. NR2	Streptococcus koreensis	-0.3799	0.001	0.892940731	NA	NA	NA	Bacteria	Bacteria	intra
SBS I vs. Control	Citricoccus sp. NR2	Streptosporangium sp. 'caverna'	0.3052	0.003	0.892940731	NA	NA	NA	Bacteria	Bacteria	intra
SBS I vs. Control	Citricoccus sp. NR2	Tricharina praecox	-0.2716	0.002	0.892940731	NA	NA	NA	Bacteria	Bacteria	intra
SBS I vs. Control	Citrobacter koseri	Helcobacillus massiliensis	-0.2833	0.004	0.892940731	NA	NA	NA	Bacteria	Bacteria	intra
SBS I vs. Control	Cladophialophora carrionii	Hydrogenophaga sp. PAMC20947	-0.2645	0.003	0.892940731	NA	NA	NA	Fungi	Bacteria	inter
SBS I vs. Control	Cladophialophora carrionii	Methylococcus capsulatus	-0.3021	0.002	0.892940731	NA	NA	NA	Fungi	Bacteria	inter
SBS I vs. Control	Cladophialophora psammophila	Flavobacterium ginsengisoli	-0.2916	0.004	0.892940731	NA	NA	NA	Fungi	Bacteria	inter
SBS I vs. Control	Cladophialophora psammophila	Microcella sp.	-0.2514	0.001	0.892940731	NA	NA	NA	Fungi	Bacteria	inter
SBS I vs. Control	Cladophialophora psammophila	Variovorax sp. 38R	0.3038	0.004	0.892940731	NA	NA	NA	Fungi	Bacteria	inter

SBS I vs. Control	Clavibacter michiganensis	Leptolyngbya sp. O-77	0.3337	0.004	0.892940731	NA	NA	NA	Bacteria	Bacteria	intra
SBS I vs. Control	Clavibacter michiganensis	Mycolicibacterium chubuense	0.256	0.001	0.892940731	NA	NA	NA	Bacteria	Bacteria	intra
SBS I vs. Control	Clavibacter michiganensis	Peptostreptococcus equinus	-0.3488	0.001	0.892940731	NA	NA	NA	Bacteria	Bacteria	intra
SBS I vs. Control	Clavibacter michiganensis	Streptomyces sp. NBC_01450	0.3592	0.003	0.892940731	NA	NA	NA	Bacteria	Bacteria	intra
SBS I vs. Control	Clavibacter sepedonicus	Sulfitobacter mediterraneus	-0.2552	0.004	0.892940731	NA	NA	NA	Bacteria	Bacteria	intra
SBS I vs. Control	Cloacibacillus porcorum	Mycobacterium kansasii	-0.2777	0.001	0.892940731	NA	NA	NA	Bacteria	Bacteria	intra
SBS I vs. Control	Clostridioides difficile	Rhodococcus sovatensis	-0.3188	0.003	0.892940731	NA	NA	NA	Bacteria	Bacteria	intra
SBS I vs. Control	Clostridium baratii	Streptococcus koreensis	-0.3078	0.003	0.892940731	NA	NA	NA	Bacteria	Bacteria	intra
SBS I vs. Control	Clostridium baratii	Streptomyces sp. NBC_00536	-0.3165	0.001	0.892940731	NA	NA	NA	Bacteria	Bacteria	intra
SBS I vs. Control	Clostridium beijerinckii	Herbiconiux sp. L3-i23	-0.3625	0.003	0.892940731	NA	NA	NA	Bacteria	Bacteria	intra
SBS I vs. Control	Clostridium beijerinckii	Rhodococcus sp. B7740	0.2503	0.001	0.892940731	NA	NA	NA	Bacteria	Bacteria	intra
SBS I vs. Control	Clostridium botulinum	Phenylobacterium parvum	-0.2797	0.003	0.892940731	NA	NA	NA	Bacteria	Bacteria	intra
SBS I vs. Control	Clostridium butyricum	Herbiconiux sp. SALV-R1	0.2801	0.001	0.892940731	NA	NA	NA	Bacteria	Bacteria	intra
SBS I vs. Control	Clostridium butyricum	Sphingobium sp. TKS	-0.3451	0.003	0.892940731	NA	NA	NA	Bacteria	Bacteria	intra
SBS I vs. Control	Clostridium butyricum	Streptomyces sp. NBC_01304	-0.3008	0.002	0.892940731	NA	NA	NA	Bacteria	Bacteria	intra
SBS I vs. Control	Clostridium cellulovorans	Corynebacterium glaucum	0.2525	0.004	0.892940731	NA	NA	NA	Bacteria	Bacteria	intra
SBS I vs. Control	Clostridium chauvoei	Halococcus sp. PRR34	0.4416	0.002	0.892940731	NA	NA	NA	Bacteria	Archaea	inter
SBS I vs. Control	Clostridium chauvoei	Paludicola sp. MB14-C6	0.2555	0.004	0.892940731	NA	NA	NA	Bacteria	Bacteria	intra
SBS I vs. Control	Clostridium estertheticum	Paraclostridium sordellii	-0.3483	0.004	0.892940731	NA	NA	NA	Bacteria	Bacteria	intra
SBS I vs. Control	Clostridium isatidis	Corynebacterium ulcerans	-0.435	0.003	0.892940731	NA	NA	NA	Bacteria	Bacteria	intra
SBS I vs.	Clostridium saccharobutylicum	Xanthomonas phaseoli	0.3011	0.002	0.892940731	NA	NA	NA	Bacteria	Bacteria	intra

Control											
SBS I vs. Control	Clostridium saccharoperbutylacetonicum	Methylosinus sp. C49	-0.2777	0.001	0.892940731	NA	NA	NA	Bacteria	Bacteria	intra
SBS I vs. Control	Clostridium septicum	Tessaracoccus sp. MC1865	-0.2654	0.003	0.892940731	NA	NA	NA	Bacteria	Bacteria	intra
SBS I vs. Control	Clostridium sp. 001	Faecalibacterium sp. IP-1-18	-0.267	0.004	0.892940731	NA	NA	NA	Bacteria	Bacteria	intra
SBS I vs. Control	Clostridium sp. 001	Pectobacterium wasabiae	0.347	0.002	0.892940731	NA	NA	NA	Bacteria	Bacteria	intra
SBS I vs. Control	Clostridium sp. BJN0001	Halobacterium bonnevillei	-0.3217	0.001	0.892940731	NA	NA	NA	Bacteria	Archaea	inter
SBS I vs. Control	Clostridium sp. JN-9	Faecalibacillus intestinalis	-0.2766	0.004	0.892940731	NA	NA	NA	Bacteria	Bacteria	intra
SBS I vs. Control	Clostridium sp. M62/1	Devosia sp.	-0.3684	0.004	0.892940731	NA	NA	NA	Bacteria	Bacteria	intra
SBS I vs. Control	Clostridium sp. M62/1	Opitutus sp. GAS368	0.2725	0.001	0.892940731	NA	NA	NA	Bacteria	Bacteria	intra
SBS I vs. Control	Clostridium sp. M62/1	Pseudanabaena sp. ABRG5-3	0.3719	0.001	0.892940731	NA	NA	NA	Bacteria	Bacteria	intra
SBS I vs. Control	Cnuibacter physcomitrellae	Jiangella alkaliphila	0.3975	0.003	0.892940731	NA	NA	NA	Bacteria	Bacteria	intra
SBS I vs. Control	Cnuibacter physcomitrellae	Methylobacterium phyllosphaerae	0.2587	0.004	0.892940731	NA	NA	NA	Bacteria	Bacteria	intra
SBS I vs. Control	Cnuibacter physcomitrellae	Microbacterium sp. H1-D42	-0.2565	0.004	0.892940731	NA	NA	NA	Bacteria	Bacteria	intra
SBS I vs. Control	Cnuibacter physcomitrellae	Piscinibacter gummiphilus	0.2646	0.004	0.892940731	NA	NA	NA	Bacteria	Bacteria	intra
SBS I vs. Control	Cohnella candidum	Glutamicibacter nicotianae	0.3082	0.003	0.892940731	NA	NA	NA	Bacteria	Bacteria	intra
SBS I vs. Control	Cohnella candidum	Leclercia sp. W6	0.3609	0.002	0.892940731	NA	NA	NA	Bacteria	Bacteria	intra
SBS I vs. Control	Cohnella candidum	Mycobacterium kansasii	0.2713	0.004	0.892940731	NA	NA	NA	Bacteria	Bacteria	intra
SBS I vs. Control	Cohnella candidum	Mycolicibacterium goodii	-0.2801	0.001	0.892940731	NA	NA	NA	Bacteria	Bacteria	intra
SBS I vs. Control	Cohnella candidum	Pseudobutyrivibrio xylanivorans	0.396	0.002	0.892940731	NA	NA	NA	Bacteria	Bacteria	intra
SBS I vs. Control	Cohnella cholangitidis	Paenarthrobacter ureafaciens	-0.3358	0.004	0.892940731	NA	NA	NA	Bacteria	Bacteria	intra
SBS I vs. Control	Cohnella cholangitidis	Streptococcus sp. FSL W8-0197	-0.317	0.003	0.892940731	NA	NA	NA	Bacteria	Bacteria	intra

SBS I vs. Control	Cohnella cholangitidis	Uncinocarpus reesii	0.2811	0.001	0.892940731	NA	NA	NA	Bacteria	Bacteria	intra
SBS I vs. Control	Collinsella aerofaciens	Pediococcus claussenii	-0.3277	0.002	0.892940731	NA	NA	NA	Bacteria	Bacteria	intra
SBS I vs. Control	Collinsella stercoris	Paraoerskovia marina	0.3679	0.001	0.892940731	NA	NA	NA	Bacteria	Bacteria	intra
SBS I vs. Control	Collinsella stercoris	Streptomyces sp. NBC_00316	-0.3106	0.001	0.892940731	NA	NA	NA	Bacteria	Bacteria	intra
SBS I vs. Control	Comamonas endophytica	Priestia koreensis	-0.3594	0.004	0.892940731	NA	NA	NA	Bacteria	Bacteria	intra
SBS I vs. Control	Comamonas endophytica	Pseudomonas toyotomiensis	0.344	0.003	0.892940731	NA	NA	NA	Bacteria	Bacteria	intra
SBS I vs. Control	Comamonas flocculans	Nocardioides anomalus	-0.3213	0.001	0.892940731	NA	NA	NA	Bacteria	Bacteria	intra
SBS I vs. Control	Comamonas piscis	Erwinia sp. QL-Z3	-0.2715	0.001	0.892940731	NA	NA	NA	Bacteria	Bacteria	intra
SBS I vs. Control	Comamonas piscis	Ferrovibrio terrae	-0.2686	0.003	0.892940731	NA	NA	NA	Bacteria	Bacteria	intra
SBS I vs. Control	Conexibacter sp. DBS9H8	Polaromonas naphthalenivorans	-0.3082	0.002	0.892940731	NA	NA	NA	Bacteria	Bacteria	intra
SBS I vs. Control	Conexibacter sp. DBS9H8	Priestia koreensis	-0.2586	0.002	0.892940731	NA	NA	NA	Bacteria	Bacteria	intra
SBS I vs. Control	Conexibacter sp. DBS9H8	Pseudonocardia abyssalis	-0.2582	0.003	0.892940731	NA	NA	NA	Bacteria	Bacteria	intra
SBS I vs. Control	Conexibacter sp. DBS9H8	Rhodoluna limnophila	0.2729	0.004	0.892940731	NA	NA	NA	Bacteria	Bacteria	intra
SBS I vs. Control	Conexibacter woesei	Ferribacterium limneticum	-0.2583	0.001	0.892940731	NA	NA	NA	Bacteria	Bacteria	intra
SBS I vs. Control	Conexibacter woesei	Selenomonas sp. TAMA-11512	0.3178	0.004	0.892940731	NA	NA	NA	Bacteria	Bacteria	intra
SBS I vs. Control	Conexibacter woesei	Streptococcus lactarius	-0.2593	0.003	0.892940731	NA	NA	NA	Bacteria	Bacteria	intra
SBS I vs. Control	Coniophora puteana	Klebsiella sp. WP3-S18-ESBL-05	-0.3898	0.004	0.892940731	NA	NA	NA	Fungi	Bacteria	inter
SBS I vs. Control	Coprobacter fastidiosus	Streptomyces sp. NBC_01304	0.3271	0.003	0.892940731	NA	NA	NA	Bacteria	Bacteria	intra
SBS I vs. Control	Coprococcus catus	Curtobacterium sp. 458	0.3504	0.003	0.892940731	NA	NA	NA	Bacteria	Bacteria	intra
SBS I vs. Control	Coprococcus catus	Leisingera sp. S132	-0.2699	0.002	0.892940731	NA	NA	NA	Bacteria	Bacteria	intra
SBS I vs.	Coprococcus catus	Pseudomonas sp. DC1.2	0.309	0.004	0.892940731	NA	NA	NA	Bacteria	Bacteria	intra

Control											
SBS I vs. Control	Coprococcus comes	Haemophilus parainfluenzae	0.3072	0.003	0.892940731	NA	NA	NA	Bacteria	Bacteria	intra
SBS I vs. Control	Coprococcus sp. ART55/1	Umbelopsis ramanniana	0.4405	0.002	0.892940731	NA	NA	NA	Bacteria	Bacteria	intra
SBS I vs. Control	Corynebacterium aquilae	Streptomyces sp. RTd22	-0.2666	0.004	0.892940731	NA	NA	NA	Bacteria	Bacteria	intra
SBS I vs. Control	Corynebacterium argentoratense	Sphingopyxis sp. GC21	0.2819	0.003	0.892940731	NA	NA	NA	Bacteria	Bacteria	intra
SBS I vs. Control	Corynebacterium auriscanis	Pseudonocardia dioxanivorans	-0.2574	0.002	0.892940731	NA	NA	NA	Bacteria	Bacteria	intra
SBS I vs. Control	Corynebacterium auriscanis	Ruminococcus sp. SR1/5	-0.2808	0.003	0.892940731	NA	NA	NA	Bacteria	Bacteria	intra
SBS I vs. Control	Corynebacterium crudilactis	Deinococcus wulumuqiensis	0.2972	0.002	0.892940731	NA	NA	NA	Bacteria	Bacteria	intra
SBS I vs. Control	Corynebacterium crudilactis	Pseudoalteromonas arctica	-0.2658	0.002	0.892940731	NA	NA	NA	Bacteria	Bacteria	intra
SBS I vs. Control	Corynebacterium crudilactis	Ruminiclostridium cellulolyticum	-0.2612	0.003	0.892940731	NA	NA	NA	Bacteria	Bacteria	intra
SBS I vs. Control	Corynebacterium cyclohexanicum	Intestinibacter bartlettii	-0.272	0.004	0.892940731	NA	NA	NA	Bacteria	Bacteria	intra
SBS I vs. Control	Corynebacterium cyclohexanicum	Mycolicibacterium sp. MU0053	0.2751	0.003	0.892940731	NA	NA	NA	Bacteria	Bacteria	intra
SBS I vs. Control	Corynebacterium efficiens	Mycolicibacter terrae	-0.2743	0.001	0.892940731	NA	NA	NA	Bacteria	Bacteria	intra
SBS I vs. Control	Corynebacterium efficiens	Nocardia cyriacigeorgica	-0.2808	0.004	0.892940731	NA	NA	NA	Bacteria	Bacteria	intra
SBS I vs. Control	Corynebacterium faecale	Veillonella sp. S12025-13	-0.2669	0.002	0.892940731	NA	NA	NA	Bacteria	Bacteria	intra
SBS I vs. Control	Corynebacterium flavescens	Sphingomonas bisphenolicum	0.2783	0.003	0.892940731	NA	NA	NA	Bacteria	Bacteria	intra
SBS I vs. Control	Corynebacterium frankenforstense	Leucobacter triazinivorans	0.3424	0.001	0.892940731	NA	NA	NA	Bacteria	Bacteria	intra
SBS I vs. Control	Corynebacterium frankenforstense	Sinomonas atrocyanea	0.2533	0.001	0.892940731	NA	NA	NA	Bacteria	Bacteria	intra
SBS I vs. Control	Corynebacterium freiburgense	Kitasatospora fiedleri	0.2673	0.004	0.892940731	NA	NA	NA	Bacteria	Bacteria	intra
SBS I vs. Control	Corynebacterium freiburgense	Streptobacillus moniliformis	-0.2678	0.001	0.892940731	NA	NA	NA	Bacteria	Bacteria	intra
SBS I vs. Control	Corynebacterium glaucum	Fusarium vanettenii	0.2771	0.003	0.892940731	NA	NA	NA	Bacteria	Fungi	inter

SBS I vs. Control	Corynebacterium glaucum	Haladaptatus sp. DJG-WS-42	-0.3039	0.001	0.892940731	NA	NA	NA	Bacteria	Archaea	inter
SBS I vs. Control	Corynebacterium glaucum	Sanguibacter sp. HDW7	0.2909	0.003	0.892940731	NA	NA	NA	Bacteria	Bacteria	intra
SBS I vs. Control	Corynebacterium glaucum	Streptomyces sp. NBC_00287	-0.3393	0.002	0.892940731	NA	NA	NA	Bacteria	Bacteria	intra
SBS I vs. Control	Corynebacterium gottingense	Kitasatospora sp. NBC_01246	-0.2519	0.002	0.892940731	NA	NA	NA	Bacteria	Bacteria	intra
SBS I vs. Control	Corynebacterium gottingense	Microbacterium sp. NIBRBAC000506063	-0.3225	0.002	0.892940731	NA	NA	NA	Bacteria	Bacteria	intra
SBS I vs. Control	Corynebacterium hesseae	Mycolicibacterium aurum	-0.3679	0.004	0.892940731	NA	NA	NA	Bacteria	Bacteria	intra
SBS I vs. Control	Corynebacterium hindlerae	Streptococcus mitis	0.3193	0.002	0.892940731	NA	NA	NA	Bacteria	Bacteria	intra
SBS I vs. Control	Corynebacterium incognita	Psychrobacter sp. WB2	-0.2503	0.003	0.892940731	NA	NA	NA	Bacteria	Bacteria	intra
SBS I vs. Control	Corynebacterium incognita	Rhodococcus pseudokoreensis	-0.2717	0.003	0.892940731	NA	NA	NA	Bacteria	Bacteria	intra
SBS I vs. Control	Corynebacterium maris	Kitasatospora aureofaciens	-0.3235	0.001	0.892940731	NA	NA	NA	Bacteria	Bacteria	intra
SBS I vs. Control	Corynebacterium maris	Streptococcus australis	-0.3612	0.001	0.892940731	NA	NA	NA	Bacteria	Bacteria	intra
SBS I vs. Control	Corynebacterium mustelae	Geobacillus zalihae	-0.2668	0.0048	0.892940731	NA	NA	NA	Bacteria	Bacteria	intra
SBS I vs. Control	Corynebacterium mustelae	Lysobacter avium	0.3045	0.001	0.892940731	NA	NA	NA	Bacteria	Bacteria	intra
SBS I vs. Control	Corynebacterium occultum	Megamonas hypermegale	-0.2554	0.002	0.892940731	NA	NA	NA	Bacteria	Bacteria	intra
SBS I vs. Control	Corynebacterium occultum	Methanocaldococcus infernus	-0.4065	0.002	0.892940731	NA	NA	NA	Bacteria	Bacteria	intra
SBS I vs. Control	Corynebacterium occultum	Trueperella pyogenes	-0.297	0.003	0.892940731	NA	NA	NA	Bacteria	Bacteria	intra
SBS I vs. Control	Corynebacterium pseudokroppenstedtii	Rhizophagus irregularis	0.4183	0.002	0.892940731	NA	NA	NA	Bacteria	Bacteria	intra
SBS I vs. Control	Corynebacterium pseudokroppenstedtii	Zunongwangia sp. HGR-M22	0.3635	0.002	0.892940731	NA	NA	NA	Bacteria	Bacteria	intra
SBS I vs. Control	Corynebacterium sp. sy039	Lentzea guizhouensis	0.2799	0.001	0.892940731	NA	NA	NA	Bacteria	Bacteria	intra
SBS I vs. Control	Corynebacterium suedekumii	Paenibacillus sp. JNUCC-31	0.3138	0.002	0.892940731	NA	NA	NA	Bacteria	Bacteria	intra
SBS I vs.	Corynebacterium suedekumii	Streptococcus sp. zg-86	-0.2603	0.004	0.892940731	NA	NA	NA	Bacteria	Bacteria	intra

Control											
SBS I vs. Control	Corynebacterium suedekumii	Streptomyces sp. NBC_01237	0.2901	0.004	0.892940731	NA	NA	NA	Bacteria	Bacteria	intra
SBS I vs. Control	Corynebacterium terpenotabidum	Mycobacterium heckeshornense	0.2776	0.001	0.892940731	NA	NA	NA	Bacteria	Bacteria	intra
SBS I vs. Control	Corynebacterium terpenotabidum	Purpureocillium lilacinum	0.3379	0.002	0.892940731	NA	NA	NA	Bacteria	Bacteria	intra
SBS I vs. Control	Corynebacterium terpenotabidum	Streptococcus sp. 29896	0.3293	0.003	0.892940731	NA	NA	NA	Bacteria	Bacteria	intra
SBS I vs. Control	Corynebacterium testudinoris	Haloterrigena sp. KLK7	0.2829	0.003	0.892940731	NA	NA	NA	Bacteria	Archaea	inter
SBS I vs. Control	Corynebacterium timonense	Curtobacterium sp. TC1	-0.2564	0.003	0.892940731	NA	NA	NA	Bacteria	Bacteria	intra
SBS I vs. Control	Corynebacterium ulcerans	Mycolicibacterium fluoranthenivorans	0.3476	0.003	0.892940731	NA	NA	NA	Bacteria	Bacteria	intra
SBS I vs. Control	Corynebacterium vitaeruminis	Curtobacterium sp. SGAir0471	0.3102	0.002	0.892940731	NA	NA	NA	Bacteria	Bacteria	intra
SBS I vs. Control	Corynebacterium vitaeruminis	Enterococcus mundtii	0.2662	0.001	0.892940731	NA	NA	NA	Bacteria	Bacteria	intra
SBS I vs. Control	Croceicoccus sp. Ery15	Psychrobacter sp. WB2	0.3662	0.001	0.892940731	NA	NA	NA	Bacteria	Bacteria	intra
SBS I vs. Control	Croceicoccus sp. YJ47	Edwardsiella piscicida	0.2641	0.004	0.892940731	NA	NA	NA	Bacteria	Bacteria	intra
SBS I vs. Control	Cronobacter malonaticus	Haemophilus sp. oral taxon 036	-0.2818	0.004	0.892940731	NA	NA	NA	Bacteria	Bacteria	intra
SBS I vs. Control	Cronobacter malonaticus	Streptococcus infantis	0.3115	0.002	0.892940731	NA	NA	NA	Bacteria	Bacteria	intra
SBS I vs. Control	Cronobacter sakazakii	uncultured Lichenicoccus sp	-0.31	0.003	0.892940731	NA	NA	NA	Bacteria	Bacteria	intra
SBS I vs. Control	Cronobacter universalis	Piscinibacter gummiphilus	-0.3212	0.001	0.892940731	NA	NA	NA	Bacteria	Bacteria	intra
SBS I vs. Control	Cronobacter universalis	Streptococcus rubneri	0.4229	0.002	0.892940731	NA	NA	NA	Bacteria	Bacteria	intra
SBS I vs. Control	Crossiella sp. CA-258035	Thermococcus sp. GR4	-0.2746	0.002	0.892940731	NA	NA	NA	Bacteria	Bacteria	intra
SBS I vs. Control	Cryobacterium arcticum	Mycetocola zhujimingii	0.3984	0.001	0.892940731	NA	NA	NA	Bacteria	Bacteria	intra
SBS I vs. Control	Cryobacterium arcticum	[Ruminococcus] torques	0.3825	0.003	0.892940731	NA	NA	NA	Bacteria	Bacteria	intra
SBS I vs. Control	Cryobacterium breve	Klebsiella aerogenes	0.2576	0.004	0.892940731	NA	NA	NA	Bacteria	Bacteria	intra

SBS I vs. Control	Cryobacterium sp. LW097	Paracoccus jeotgali	-0.3035	0.002	0.892940731	NA	NA	NA	Bacteria	Bacteria	intra
SBS I vs. Control	Cryobacterium sp. LW097	Rathayibacter rathayi	0.3919	0.003	0.892940731	NA	NA	NA	Bacteria	Bacteria	intra
SBS I vs. Control	Cryobacterium sp. SO2	Schaalia cardiffensis	-0.2507	0.002	0.892940731	NA	NA	NA	Bacteria	Bacteria	intra
SBS I vs. Control	Cryobacterium sp. SO2	Sphingobacterium sp. DR205	-0.2981	0.002	0.892940731	NA	NA	NA	Bacteria	Bacteria	intra
SBS I vs. Control	Cryptococcus neoformans	Solobacterium moorei	-0.3593	0.001	0.892940731	NA	NA	NA	Fungi	Bacteria	inter
SBS I vs. Control	Cucurbitaria berberidis	Kovacikia minuta	0.2749	0.0048	0.892940731	NA	NA	NA	Fungi	Bacteria	inter
SBS I vs. Control	Cucurbitaria berberidis	Tepidibacter hydrothermalis	0.2869	0.003	0.892940731	NA	NA	NA	Fungi	Bacteria	inter
SBS I vs. Control	Cupriavidus nantongensis	Kocuria rhizophila	-0.2786	0.004	0.892940731	NA	NA	NA	Bacteria	Bacteria	intra
SBS I vs. Control	Cupriavidus nantongensis	Leptospira sp. severe_002	0.3848	0.003	0.892940731	NA	NA	NA	Bacteria	Bacteria	intra
SBS I vs. Control	Cupriavidus nantongensis	Nocardioides cynanchi	-0.2615	0.003	0.892940731	NA	NA	NA	Bacteria	Bacteria	intra
SBS I vs. Control	Cupriavidus pinatubonensis	Ruminococcus champanellensis	-0.2798	0.001	0.892940731	NA	NA	NA	Bacteria	Bacteria	intra
SBS I vs. Control	Cupriavidus pinatubonensis	Streptococcus gwangjuense	-0.2696	0.001	0.892940731	NA	NA	NA	Bacteria	Bacteria	intra
SBS I vs. Control	Cupriavidus sp. USMAA2-4	Faecalibacterium sp. IP-3-29	0.2624	0.002	0.892940731	NA	NA	NA	Bacteria	Bacteria	intra
SBS I vs. Control	Cupriavidus sp. USMAA2-4	Streptomyces sp. SCA4-21	-0.2511	0.003	0.892940731	NA	NA	NA	Bacteria	Bacteria	intra
SBS I vs. Control	Curtobacterium sp. 458	Klebsiella pasteurii	-0.3226	0.002	0.892940731	NA	NA	NA	Bacteria	Bacteria	intra
SBS I vs. Control	Curtobacterium sp. 458	Lachnospira eligens	-0.2933	0.003	0.892940731	NA	NA	NA	Bacteria	Bacteria	intra
SBS I vs. Control	Curtobacterium sp. 458	Natronomonas halophila	0.3036	0.002	0.892940731	NA	NA	NA	Bacteria	Bacteria	intra
SBS I vs. Control	Curtobacterium sp. DN_7.5	Flavonifractor plautii	-0.291	0.001	0.892940731	NA	NA	NA	Bacteria	Bacteria	intra
SBS I vs. Control	Curtobacterium sp. DN_7.5	Glutamicibacter mishrai	-0.2591	0.003	0.892940731	NA	NA	NA	Bacteria	Bacteria	intra
SBS I vs. Control	Curtobacterium sp. DN_7.5	Xanthomonas euvesicatoria	-0.2634	0.002	0.892940731	NA	NA	NA	Bacteria	Bacteria	intra
SBS I vs.	Curtobacterium sp. L6-1	[Ruminococcus] torques	-0.2698	0.003	0.892940731	NA	NA	NA	Bacteria	Bacteria	intra

Control											
SBS I vs. Control	Curtobacterium sp. MCLR17_007	Hartmannibacter diazotrophicus	0.3346	0.004	0.892940731	NA	NA	NA	Bacteria	Bacteria	intra
SBS I vs. Control	Curtobacterium sp. MCLR17_007	Microbacterium binotii	-0.2653	0.002	0.892940731	NA	NA	NA	Bacteria	Bacteria	intra
SBS I vs. Control	Curtobacterium sp. MCLR17_007	Rathayibacter sp. VKM Ac-2805	0.2549	0.003	0.892940731	NA	NA	NA	Bacteria	Bacteria	intra
SBS I vs. Control	Curtobacterium sp. MCLR17_007	Syntrophotalea acetylenica	-0.299	0.002	0.892940731	NA	NA	NA	Bacteria	Bacteria	intra
SBS I vs. Control	Curtobacterium sp. MCLR17_032	Spirillospora sp. NBC_00431	-0.3226	0.002	0.892940731	NA	NA	NA	Bacteria	Bacteria	intra
SBS I vs. Control	Curtobacterium sp. MR_MD2014	Lysobacter sp. S4-A87	-0.3534	0.001	0.892940731	NA	NA	NA	Bacteria	Bacteria	intra
SBS I vs. Control	Curtobacterium sp. MR_MD2014	Nocardioides cynanchi	-0.3335	0.004	0.892940731	NA	NA	NA	Bacteria	Bacteria	intra
SBS I vs. Control	Curtobacterium sp. MR_MD2014	Pseudomonas cavernae	-0.2829	0.001	0.892940731	NA	NA	NA	Bacteria	Bacteria	intra
SBS I vs. Control	Curtobacterium sp. SGAir0471	Herbiconiux sp. SALV-R1	-0.2526	0.002	0.892940731	NA	NA	NA	Bacteria	Bacteria	intra
SBS I vs. Control	Cyclobacterium marinum	Saccharopolyspora erythraea	0.4657	0.001	0.892940731	NA	NA	NA	Bacteria	Bacteria	intra
SBS I vs. Control	Cyclobacterium marinum	Treponema vincentii	-0.279	0.004	0.892940731	NA	NA	NA	Bacteria	Bacteria	intra
SBS I vs. Control	Cytobacillus oceanisediminis	Enterobacter kobei	0.3925	0.002	0.892940731	NA	NA	NA	Bacteria	Bacteria	intra
SBS I vs. Control	Cytobacillus oceanisediminis	Erwinia sp. QL-Z3	-0.3474	0.003	0.892940731	NA	NA	NA	Bacteria	Bacteria	intra
SBS I vs. Control	Cytobacillus oceanisediminis	Klebsiella pneumoniae	-0.3383	0.003	0.892940731	NA	NA	NA	Bacteria	Bacteria	intra
SBS I vs. Control	Cytobacillus oceanisediminis	Sphingobacterium sp. ML3W	0.2543	0.001	0.892940731	NA	NA	NA	Bacteria	Bacteria	intra
SBS I vs. Control	Dechloromonas sp. A34	Gryllotalpicola protaetiae	0.2714	0.003	0.892940731	NA	NA	NA	Bacteria	Bacteria	intra
SBS I vs. Control	Dechloromonas sp. A34	Pyruvatibacter mobilis	0.2551	0.003	0.892940731	NA	NA	NA	Bacteria	Bacteria	intra
SBS I vs. Control	Deinococcus geothermalis	Streptococcus australis	0.2957	0.003	0.892940731	NA	NA	NA	Bacteria	Bacteria	intra
SBS I vs. Control	Deinococcus wulumuqiensis	Helcobacillus massiliensis	-0.2754	0.003	0.892940731	NA	NA	NA	Bacteria	Bacteria	intra
SBS I vs. Control	Demequina sediminis	Paenibacillus sp. RC67	0.2684	0.003	0.892940731	NA	NA	NA	Bacteria	Bacteria	intra

SBS I vs. Control	Demequina sp. OYTSA14	Methanococcus voltae	-0.3218	0.003	0.892940731	NA	NA	NA	Bacteria	Bacteria	intra
SBS I vs. Control	Demequina sp. OYTSA14	Neisseria yangbaofengii	-0.3108	0.002	0.892940731	NA	NA	NA	Bacteria	Bacteria	intra
SBS I vs. Control	Dermacoccus nishinomiyaensis	Tichowtungia aerotolerans	0.2846	0.001	0.892940731	NA	NA	NA	Bacteria	Bacteria	intra
SBS I vs. Control	Dermatophilus congolensis	Enterobacter sp. AN-K1	-0.2945	0.003	0.892940731	NA	NA	NA	Bacteria	Bacteria	intra
SBS I vs. Control	Dermatophilus congolensis	Gordonia hydrophobica	-0.2723	0.003	0.892940731	NA	NA	NA	Bacteria	Bacteria	intra
SBS I vs. Control	Dermatophilus congolensis	Klebsiella aerogenes	-0.2686	0.003	0.892940731	NA	NA	NA	Bacteria	Bacteria	intra
SBS I vs. Control	Desulfobulbus propionicus	Olsenella sp. oral taxon 807	-0.2716	0.001	0.892940731	NA	NA	NA	Bacteria	Bacteria	intra
SBS I vs. Control	Desulfocurvibacter africanus	Mycobacterium heckeshornense	0.2614	0.003	0.892940731	NA	NA	NA	Bacteria	Bacteria	intra
SBS I vs. Control	Desulfonema limicola	Leucobacter triazinivorans	0.2955	0.002	0.892940731	NA	NA	NA	Bacteria	Bacteria	intra
SBS I vs. Control	Desulfosarcina ovata	uncultured Methanobrevibacter sp	0.3297	0.004	0.892940731	NA	NA	NA	Bacteria	Bacteria	intra
SBS I vs. Control	Desulfoscipio gibsoniae	Trueperella bernardiae	0.2837	0.003	0.892940731	NA	NA	NA	Bacteria	Bacteria	intra
SBS I vs. Control	Desulfosudis oleivorans	Hyphomicrobium nitrativorans	-0.2657	0.004	0.892940731	NA	NA	NA	Bacteria	Bacteria	intra
SBS I vs. Control	Desulfosudis oleivorans	Roseburia sp. 499	-0.3232	0.002	0.892940731	NA	NA	NA	Bacteria	Bacteria	intra
SBS I vs. Control	Desulfovibrio desulfuricans	Leucobacter luti	0.2697	0.002	0.892940731	NA	NA	NA	Bacteria	Bacteria	intra
SBS I vs. Control	Desulfovibrio desulfuricans	Paenibacillus lycopersici	-0.2778	0.002	0.892940731	NA	NA	NA	Bacteria	Bacteria	intra
SBS I vs. Control	Desulfovibrio ferrophilus	Novosphingopyxis iocasae	-0.2545	0.004	0.892940731	NA	NA	NA	Bacteria	Bacteria	intra
SBS I vs. Control	Desulfovibrio ferrophilus	Paenibacillus sp. N3/727	0.2653	0.004	0.892940731	NA	NA	NA	Bacteria	Bacteria	intra
SBS I vs. Control	Devosia sp.	Geobacillus zalihae	0.3308	0.003	0.892940731	NA	NA	NA	Bacteria	Bacteria	intra
SBS I vs. Control	Devosia sp.	Saccharomonospora viridis	-0.3751	0.001	0.892940731	NA	NA	NA	Bacteria	Bacteria	intra
SBS I vs. Control	Devosia sp. A16	Mesorhizobium sp. 8	0.3196	0.002	0.892940731	NA	NA	NA	Bacteria	Bacteria	intra
SBS I vs.	Devosia sp. A16	Paraoerskovia sediminicola	0.3051	0.001	0.892940731	NA	NA	NA	Bacteria	Bacteria	intra

Control											
SBS I vs. Control	Diaphorobacter sp. HDW4B	Leptolyngbya sp. O-77	0.2577	0.003	0.892940731	NA	NA	NA	Bacteria	Bacteria	intra
SBS I vs. Control	Diaphorobacter sp. HDW4B	Neobacillus sp. PS2-9	0.2628	0.004	0.892940731	NA	NA	NA	Bacteria	Bacteria	intra
SBS I vs. Control	Diaphorobacter sp. HDW4B	Streptomyces rimosus	0.3519	0.001	0.892940731	NA	NA	NA	Bacteria	Bacteria	intra
SBS I vs. Control	Dickeya dianthicola	Thermoanaerobacterium thermosaccharolyticum	-0.2858	0.002	0.892940731	NA	NA	NA	Bacteria	Bacteria	intra
SBS I vs. Control	Dietzia cinnamea	Mycolicibacterium gadium	0.2524	0.001	0.892940731	NA	NA	NA	Bacteria	Bacteria	intra
SBS I vs. Control	Dietzia cinnamea	Pseudomonas fuscovaginae	0.2577	0.002	0.892940731	NA	NA	NA	Bacteria	Bacteria	intra
SBS I vs. Control	Dietzia cinnamea	Sanguibacter sp. 4.1	0.3192	0.004	0.892940731	NA	NA	NA	Bacteria	Bacteria	intra
SBS I vs. Control	Dietzia sp. B32	Microbacterium hydrocarbonoxydans	-0.2797	0.003	0.892940731	NA	NA	NA	Bacteria	Bacteria	intra
SBS I vs. Control	Dietzia sp. B32	uncultured Desulfosarcina sp	-0.4117	0.001	0.892940731	NA	NA	NA	Bacteria	Bacteria	intra
SBS I vs. Control	Dorea longicatena	Microbacterium sp. YJN-G	0.265	0.001	0.892940731	NA	NA	NA	Bacteria	Bacteria	intra
SBS I vs. Control	Draconibacterium halophilum	Faecalibacterium sp. I3-3-89	-0.4002	0.001	0.892940731	NA	NA	NA	Bacteria	Bacteria	intra
SBS I vs. Control	Draconibacterium halophilum	Kitasatospora sp. HUAS 3-15	-0.462	0.004	0.892940731	NA	NA	NA	Bacteria	Bacteria	intra
SBS I vs. Control	Draconibacterium halophilum	Nocardioides scoriae	-0.3993	0.001	0.892940731	NA	NA	NA	Bacteria	Bacteria	intra
SBS I vs. Control	Draconibacterium halophilum	Rothia sp. ZJ932	0.2729	0.004	0.892940731	NA	NA	NA	Bacteria	Bacteria	intra
SBS I vs. Control	Duganella zoogloeoides	Microbacterium fandaimingii	0.2863	0.003	0.892940731	NA	NA	NA	Bacteria	Bacteria	intra
SBS I vs. Control	Duganella zoogloeoides	Raoultella ornithinolytica	-0.2762	0.003	0.892940731	NA	NA	NA	Bacteria	Bacteria	intra
SBS I vs. Control	Duodenibacillus massiliensis	Faecalibacterium sp. IP-3-29	-0.2601	0.003	0.892940731	NA	NA	NA	Bacteria	Bacteria	intra
SBS I vs. Control	Duodenibacillus massiliensis	Mediterraneibacter gnavus	-0.2823	0.002	0.892940731	NA	NA	NA	Bacteria	Bacteria	intra
SBS I vs. Control	Duodenibacillus massiliensis	Pseudoxanthomonas sp. X-1	-0.3373	0.001	0.892940731	NA	NA	NA	Bacteria	Bacteria	intra
SBS I vs. Control	Duodenibacillus massiliensis	uncultured Trichococcus sp	0.2512	0.002	0.892940731	NA	NA	NA	Bacteria	Bacteria	intra

SBS I vs. Control	Dyella thiooxydans	Mycobacterium heckeshornense	0.254	0.001	0.892940731	NA	NA	NA	Bacteria	Bacteria	intra
SBS I vs. Control	Dyella thiooxydans	Penicillium cosmopolitanum	-0.3017	0.003	0.892940731	NA	NA	NA	Bacteria	Bacteria	intra
SBS I vs. Control	Dysosmobacter welbionis	Synechococcus sp. CBW1107	0.288	0.004	0.892940731	NA	NA	NA	Bacteria	Bacteria	intra
SBS I vs. Control	Edwardsiella piscicida	Halopseudomonas nanhaiensis	-0.3947	0.003	0.892940731	NA	NA	NA	Bacteria	Bacteria	intra
SBS I vs. Control	Eggerthella lenta	Parvimonas micra	-0.2805	0.003	0.892940731	NA	NA	NA	Bacteria	Bacteria	intra
SBS I vs. Control	Emergencia sp. JLR.KK010	Mongoliitalea daihaiensis	-0.3418	0.001	0.892940731	NA	NA	NA	Bacteria	Bacteria	intra
SBS I vs. Control	Emergencia sp. JLR.KK010	Pectobacterium polaris	-0.3202	0.001	0.892940731	NA	NA	NA	Bacteria	Bacteria	intra
SBS I vs. Control	Emergencia sp. JLR.KK010	Rothia dentocariosa	-0.2695	0.003	0.892940731	NA	NA	NA	Bacteria	Bacteria	intra
SBS I vs. Control	Emergencia timonensis	Glutamicibacter mishrai	-0.3164	0.003	0.892940731	NA	NA	NA	Bacteria	Bacteria	intra
SBS I vs. Control	Empedobacter brevis	Lactobacillus delbrueckii	0.2641	0.001	0.892940731	NA	NA	NA	Bacteria	Bacteria	intra
SBS I vs. Control	Empedobacter brevis	Methylosinus sp. C49	0.2844	0.003	0.892940731	NA	NA	NA	Bacteria	Bacteria	intra
SBS I vs. Control	Empedobacter brevis	Paraburkholderia tropica	-0.2895	0.004	0.892940731	NA	NA	NA	Bacteria	Bacteria	intra
SBS I vs. Control	Emticicia oligotrophica	Geomonas subterranea	-0.2665	0.001	0.892940731	NA	NA	NA	Bacteria	Bacteria	intra
SBS I vs. Control	Emticicia oligotrophica	Halolamina rubra	-0.2636	0.004	0.892940731	NA	NA	NA	Bacteria	Archaea	inter
SBS I vs. Control	Endocarpon pusillum	Mycolicibacterium sp. TUM20985	0.2826	0.002	0.892940731	NA	NA	NA	Fungi	Bacteria	inter
SBS I vs. Control	Endocarpon pusillum	Pseudomonas sessilinigenes	-0.3042	0.002	0.892940731	NA	NA	NA	Fungi	Bacteria	inter
SBS I vs. Control	Ensifer adhaerens	Methylobacter sp. YRD-M1	-0.3011	0.002	0.892940731	NA	NA	NA	Bacteria	Bacteria	intra
SBS I vs. Control	Enterobacter cloacae complex sp. FDA-CDC-AR_0164	Neisseria cinerea	0.3101	0.004	0.892940731	NA	NA	NA	Bacteria	Bacteria	intra
SBS I vs. Control	Enterobacter cloacae complex sp. R_G8	Streptosporangium sp. 'caverna'	-0.2696	0.003	0.892940731	NA	NA	NA	Bacteria	Bacteria	intra
SBS I vs. Control	Enterobacter kobei	Micromonospora sp. NBC_01796	0.3235	0.002	0.892940731	NA	NA	NA	Bacteria	Bacteria	intra
SBS I vs.	Enterobacter kobei	Plantibacter flavus	-0.253	0.004	0.892940731	NA	NA	NA	Bacteria	Bacteria	intra

Control											
SBS I vs. Control	Enterobacter kobei	Variovorax boronicumulans	0.3018	0.004	0.892940731	NA	NA	NA	Bacteria	Bacteria	intra
SBS I vs. Control	Enterobacter ludwigii	Streptomyces sp. f51	-0.2627	0.001	0.892940731	NA	NA	NA	Bacteria	Bacteria	intra
SBS I vs. Control	Enterobacter sp. AN-K1	Enterococcus sp. DIV1094	0.3555	0.004	0.892940731	NA	NA	NA	Bacteria	Bacteria	intra
SBS I vs. Control	Enterobacter sp. AN-K1	Microlunatus soli	-0.2892	0.004	0.892940731	NA	NA	NA	Bacteria	Bacteria	intra
SBS I vs. Control	Enterobacter sp. AN-K1	Paenibacillus sp. FSL R10-2734	0.3416	0.003	0.892940731	NA	NA	NA	Bacteria	Bacteria	intra
SBS I vs. Control	Enterobacter sp. Colony194	Faecalibacterium sp. I4-1-79	-0.2672	0.002	0.892940731	NA	NA	NA	Bacteria	Bacteria	intra
SBS I vs. Control	Enterobacter sp. Colony194	Fuerstiella marisgermanici	-0.2883	0.003	0.892940731	NA	NA	NA	Bacteria	Bacteria	intra
SBS I vs. Control	Enterobacter sp. Colony194	Helicobacter pylori	0.2676	0.002	0.892940731	NA	NA	NA	Bacteria	Bacteria	intra
SBS I vs. Control	Enterobacter sp. Colony194	Kribbella sp. NBC_01245	-0.2809	0.001	0.892940731	NA	NA	NA	Bacteria	Bacteria	intra
SBS I vs. Control	Enterobacter sp. Colony194	Mycobacterium sp. MS1601	-0.3091	0.001	0.892940731	NA	NA	NA	Bacteria	Bacteria	intra
SBS I vs. Control	Enterobacter sp. Colony194	Penicillium soppii	0.2543	0.003	0.892940731	NA	NA	NA	Bacteria	Bacteria	intra
SBS I vs. Control	Enterobacter sp. Colony194	Zhouia spongiae	-0.2902	0.003	0.892940731	NA	NA	NA	Bacteria	Bacteria	intra
SBS I vs. Control	Enterobacter sp. Crenshaw	Paenibacillus urinalis	-0.2646	0.001	0.892940731	NA	NA	NA	Bacteria	Bacteria	intra
SBS I vs. Control	Enterobacter sp. DSM 30060	Paeniglutamicibacter sp. Y32M11	0.2698	0.001	0.892940731	NA	NA	NA	Bacteria	Bacteria	intra
SBS I vs. Control	Enterobacter sp. JBIWA003	Ruminiclostridium cellulolyticum	0.2675	0.001	0.892940731	NA	NA	NA	Bacteria	Bacteria	intra
SBS I vs. Control	Enterobacter sp. JBIWA003	Streptococcus lactarius	-0.2529	0.003	0.892940731	NA	NA	NA	Bacteria	Bacteria	intra
SBS I vs. Control	Enterobacter sp. JJBC	Metabacillus sediminilitoris	-0.2567	0.002	0.892940731	NA	NA	NA	Bacteria	Bacteria	intra
SBS I vs. Control	Enterobacter sp. N18-03635	Skermanella pratensis	-0.2862	0.004	0.892940731	NA	NA	NA	Bacteria	Bacteria	intra
SBS I vs. Control	Enterobacter sp. R4-368	Hartmannibacter diazotrophicus	0.273	0.002	0.892940731	NA	NA	NA	Bacteria	Bacteria	intra
SBS I vs. Control	Enterobacter sp. R4-368	Microbacterium esteraromaticum	0.357	0.001	0.892940731	NA	NA	NA	Bacteria	Bacteria	intra

SBS I vs. Control	Enterobacter sp. R4-368	Shewanella polaris	0.2769	0.001	0.892940731	NA	NA	NA	Bacteria	Bacteria	intra
SBS I vs. Control	Enterocloster asparagiformis	Thioclava sp. GXIMD4216	0.2683	0.004	0.892940731	NA	NA	NA	Bacteria	Bacteria	intra
SBS I vs. Control	Enterococcus faecalis	Eubacterium sp. MSJ-33	-0.2621	0.003	0.892940731	NA	NA	NA	Bacteria	Bacteria	intra
SBS I vs. Control	Enterococcus faecalis	Microbacterium sp. PM5	0.2606	0.002	0.892940731	NA	NA	NA	Bacteria	Bacteria	intra
SBS I vs. Control	Enterococcus faecalis	Peptacetobacter hiranonis	-0.3121	0.004	0.892940731	NA	NA	NA	Bacteria	Bacteria	intra
SBS I vs. Control	Enterococcus faecalis	Sphingobium sp. TKS	-0.346	0.004	0.892940731	NA	NA	NA	Bacteria	Bacteria	intra
SBS I vs. Control	Enterococcus faecium	Nocardia puris	-0.2529	0.001	0.892940731	NA	NA	NA	Bacteria	Bacteria	intra
SBS I vs. Control	Enterococcus faecium	Streptococcus gwangjuense	0.2765	0.002	0.892940731	NA	NA	NA	Bacteria	Bacteria	intra
SBS I vs. Control	Enterococcus faecium	Streptomyces flavofungini	-0.2681	0.001	0.892940731	NA	NA	NA	Bacteria	Bacteria	intra
SBS I vs. Control	Enterococcus faecium	Streptomyces sp. RTd22	0.2826	0.001	0.892940731	NA	NA	NA	Bacteria	Bacteria	intra
SBS I vs. Control	Enterococcus gilvus	Leptolyngbya sp. O-77	-0.3133	0.001	0.892940731	NA	NA	NA	Bacteria	Bacteria	intra
SBS I vs. Control	Enterococcus gilvus	Streptomyces sp. NBC_01450	0.2833	0.002	0.892940731	NA	NA	NA	Bacteria	Bacteria	intra
SBS I vs. Control	Enterococcus mundtii	Microcella indica	-0.309	0.001	0.892940731	NA	NA	NA	Bacteria	Bacteria	intra
SBS I vs. Control	Enterococcus mundtii	Paracoccus zhejiangensis	0.2742	0.004	0.892940731	NA	NA	NA	Bacteria	Bacteria	intra
SBS I vs. Control	Enterococcus mundtii	Penicillium soppii	-0.3076	0.004	0.892940731	NA	NA	NA	Bacteria	Bacteria	intra
SBS I vs. Control	Enterococcus mundtii	Streptococcus koreensis	0.2889	0.003	0.892940731	NA	NA	NA	Bacteria	Bacteria	intra
SBS I vs. Control	Enterococcus mundtii	Streptomyces bathyalis	-0.2715	0.002	0.892940731	NA	NA	NA	Bacteria	Bacteria	intra
SBS I vs. Control	Enterococcus mundtii	Tumebacillus avium	-0.2617	0.001	0.892940731	NA	NA	NA	Bacteria	Bacteria	intra
SBS I vs. Control	Enterococcus rotai	Leptospira sp. severe_002	-0.3195	0.001	0.892940731	NA	NA	NA	Bacteria	Bacteria	intra
SBS I vs. Control	Enterococcus rotai	Leucobacter rhizosphaerae	-0.2929	0.003	0.892940731	NA	NA	NA	Bacteria	Bacteria	intra
SBS I vs.	Enterococcus rotai	Rhizobium gei	-0.2536	0.002	0.892940731	NA	NA	NA	Bacteria	Bacteria	intra

Control											
SBS I vs. Control	Enterococcus rotai	Salipiger profundus	0.3256	0.004	0.892940731	NA	NA	NA	Bacteria	Bacteria	intra
SBS I vs. Control	Enterococcus sp. DIV1094	Mongoliitalea daihaiensis	0.2796	0.003	0.892940731	NA	NA	NA	Bacteria	Bacteria	intra
SBS I vs. Control	Enterococcus sp. DIV1094	Streptomyces sp. NBC_00316	-0.4109	0.001	0.892940731	NA	NA	NA	Bacteria	Bacteria	intra
SBS I vs. Control	Enterococcus wangshanyuanii	Lactobacillus paragasseri	-0.2619	0.002	0.892940731	NA	NA	NA	Bacteria	Bacteria	intra
SBS I vs. Control	Enterococcus wangshanyuanii	Mucilaginibacter mali	-0.2686	0.002	0.892940731	NA	NA	NA	Bacteria	Bacteria	intra
SBS I vs. Control	Erwinia sp. QL-Z3	Massilia sp. YMA4	-0.2593	0.002	0.892940731	NA	NA	NA	Bacteria	Bacteria	intra
SBS I vs. Control	Erythrobacter litoralis	Kocuria varians	-0.3475	0.001	0.892940731	NA	NA	NA	Bacteria	Bacteria	intra
SBS I vs. Control	Erythrobacter sp. SCSIO 43205	Streptomyces sp. NBC_01304	-0.322	0.003	0.892940731	NA	NA	NA	Bacteria	Bacteria	intra
SBS I vs. Control	Escherichia coli	Lachnoanaerobaculum gingivalis	-0.2605	0.003	0.892940731	NA	NA	NA	Bacteria	Bacteria	intra
SBS I vs. Control	Escherichia coli	Streptomyces chartreusis	0.255	0.002	0.892940731	NA	NA	NA	Bacteria	Bacteria	intra
SBS I vs. Control	Escherichia fergusonii	Kocuria rosea	0.302	0.001	0.892940731	NA	NA	NA	Bacteria	Bacteria	intra
SBS I vs. Control	Escherichia fergusonii	Nitrospirillum amazonense	0.2562	0.003	0.892940731	NA	NA	NA	Bacteria	Bacteria	intra
SBS I vs. Control	Eubacterium maltosivorans	Streptococcus koreensis	-0.2712	0.004	0.892940731	NA	NA	NA	Bacteria	Bacteria	intra
SBS I vs. Control	Exserohilum turcicum	Mesorhizobium sp. M1D.F.Ca.ET.043.01.1.1	-0.2551	0.001	0.892940731	NA	NA	NA	Fungi	Bacteria	inter
SBS I vs. Control	Facklamia hominis	Methylocella silvestris	-0.2941	0.001	0.892940731	NA	NA	NA	Bacteria	Bacteria	intra
SBS I vs. Control	Facklamia hominis	Serratia liquefaciens	0.2582	0.003	0.892940731	NA	NA	NA	Bacteria	Bacteria	intra
SBS I vs. Control	Facklamia hominis	Thermaerobacter marianensis	0.3796	0.001	0.892940731	NA	NA	NA	Bacteria	Bacteria	intra
SBS I vs. Control	Faecalibacillus intestinalis	Pseudoxanthomonas sp. X-1	0.2759	0.001	0.892940731	NA	NA	NA	Bacteria	Bacteria	intra
SBS I vs. Control	Faecalibacillus intestinalis	Streptococcus oralis	-0.2539	0.002	0.892940731	NA	NA	NA	Bacteria	Bacteria	intra
SBS I vs. Control	Faecalibacterium duncaniae	Faecalibacterium prausnitzii	0.3113	0.001	0.892940731	NA	NA	NA	Bacteria	Bacteria	intra

SBS I vs. Control	Faecalibacterium duncaniae	Mycobacterium tuberculosis	-0.3073	0.003	0.892940731	NA	NA	NA	Bacteria	Bacteria	intra
SBS I vs. Control	Faecalibacterium duncaniae	Pasteurella skyensis	-0.2649	0.001	0.892940731	NA	NA	NA	Bacteria	Bacteria	intra
SBS I vs. Control	Faecalibacterium duncaniae	Sutcliffiella horikoshii	0.2784	0.003	0.892940731	NA	NA	NA	Bacteria	Bacteria	intra
SBS I vs. Control	Faecalibacterium prausnitzii	Thermomonospora curvata	-0.3507	0.001	0.892940731	NA	NA	NA	Bacteria	Bacteria	intra
SBS I vs. Control	Faecalibacterium sp. HTF-F	Lautropia mirabilis	-0.262	0.001	0.892940731	NA	NA	NA	Bacteria	Bacteria	intra
SBS I vs. Control	Faecalibacterium sp. HTF-F	Pseudomonas alcaligenes	0.2532	0.001	0.892940731	NA	NA	NA	Bacteria	Bacteria	intra
SBS I vs. Control	Faecalibacterium sp. HTF-F	Tannockella kyphosi	-0.2686	0.001	0.892940731	NA	NA	NA	Bacteria	Bacteria	intra
SBS I vs. Control	Faecalibacterium sp. I3-3-33	Maridesulfovibrio sp.	0.3144	0.001	0.892940731	NA	NA	NA	Bacteria	Bacteria	intra
SBS I vs. Control	Faecalibacterium sp. I3-3-33	Streptomyces sp. NBC_01727	0.3109	0.004	0.892940731	NA	NA	NA	Bacteria	Bacteria	intra
SBS I vs. Control	Faecalibacterium sp. I4-1-79	Protaetiibacter larvae	-0.4024	0.001	0.892940731	NA	NA	NA	Bacteria	Bacteria	intra
SBS I vs. Control	Faecalibacterium sp. IP-1-18	Uncinocarpus reesii	-0.2519	0.002	0.892940731	NA	NA	NA	Bacteria	Bacteria	intra
SBS I vs. Control	Ferribacterium limneticum	Leptotrichia sp. oral taxon 218	0.2802	0.001	0.892940731	NA	NA	NA	Bacteria	Bacteria	intra
SBS I vs. Control	Ferribacterium limneticum	Pseudomonas viridiflava	0.3989	0.002	0.892940731	NA	NA	NA	Bacteria	Bacteria	intra
SBS I vs. Control	Ferroglobus placidus	Haemophilus influenzae	-0.3221	0.004	0.892940731	NA	NA	NA	Archaea	Bacteria	inter
SBS I vs. Control	Ferroglobus placidus	Streptococcus phage PH15	0.2815	0.001	0.892940731	NA	NA	NA	Archaea	Bacteria	inter
SBS I vs. Control	Ferrovibrio terrae	Microcella humidisoli	0.2925	0.004	0.892940731	NA	NA	NA	Bacteria	Bacteria	intra
SBS I vs. Control	Ferrovibrio terrae	Nocardioides cynanchi	-0.2928	0.003	0.892940731	NA	NA	NA	Bacteria	Bacteria	intra
SBS I vs. Control	Flaviflexus salsibiostraticola	Jejubacter calystegiae	-0.2892	0.003	0.892940731	NA	NA	NA	Bacteria	Bacteria	intra
SBS I vs. Control	Flaviflexus salsibiostraticola	Kineothrix sp. MB12-C1	-0.2566	0.003	0.892940731	NA	NA	NA	Bacteria	Bacteria	intra
SBS I vs. Control	Flaviflexus salsibiostraticola	Sulfitobacter sp. S190	-0.3249	0.003	0.892940731	NA	NA	NA	Bacteria	Bacteria	intra
SBS I vs.	Flavobacterium	Parolsenella catena	0.3204	0.003	0.892940731	NA	NA	NA	Bacteria	Bacteria	intra

Control	azooxidireducens										
SBS I vs. Control	Flavobacterium ginsenosidimutans	Peribacillus sp. R9-11	0.2931	0.001	0.892940731	NA	NA	NA	Bacteria	Bacteria	intra
SBS I vs. Control	Flavobacterium johnsoniae	Nocardia huaxiensis	-0.4031	0.003	0.892940731	NA	NA	NA	Bacteria	Bacteria	intra
SBS I vs. Control	Flavobacterium johnsoniae	Nocardiopsis alba	-0.2633	0.003	0.892940731	NA	NA	NA	Bacteria	Bacteria	intra
SBS I vs. Control	Flavobacterium johnsoniae	Pseudarthrobacter sp. NBSH8	-0.2604	0.001	0.892940731	NA	NA	NA	Bacteria	Bacteria	intra
SBS I vs. Control	Flavobacterium kingsejongi	Pedobacter ginsengisoli	0.3252	0.003	0.892940731	NA	NA	NA	Bacteria	Bacteria	intra
SBS I vs. Control	Flavobacterium nitratireducens	Mogibacterium neglectum	-0.3154	0.002	0.892940731	NA	NA	NA	Bacteria	Bacteria	intra
SBS I vs. Control	Flavobacterium nitratireducens	Mycolicibacterium rhodesiae	-0.3189	0.004	0.892940731	NA	NA	NA	Bacteria	Bacteria	intra
SBS I vs. Control	Flavobacterium pallidum	Gleimia europaea	-0.2528	0.003	0.892940731	NA	NA	NA	Bacteria	Bacteria	intra
SBS I vs. Control	Flavobacterium pallidum	Klebsiella huaxiensis	-0.3311	0.003	0.892940731	NA	NA	NA	Bacteria	Bacteria	intra
SBS I vs. Control	Flavobacterium pallidum	Microbacterium lushaniae	0.3175	0.001	0.892940731	NA	NA	NA	Bacteria	Bacteria	intra
SBS I vs. Control	Flavobacterium pallidum	Nodularia sphaerocarpa	0.2547	0.002	0.892940731	NA	NA	NA	Bacteria	Bacteria	intra
SBS I vs. Control	Flavobacterium pallidum	[Ruminococcus] torques	-0.2941	0.002	0.892940731	NA	NA	NA	Bacteria	Bacteria	intra
SBS I vs. Control	Flavobacterium psychrophilum	Tumebacillus avium	0.3127	0.002	0.892940731	NA	NA	NA	Bacteria	Bacteria	intra
SBS I vs. Control	Flavobacterium sp. 20NA77.7	Labrys sp. KNU-23	0.3819	0.001	0.892940731	NA	NA	NA	Bacteria	Bacteria	intra
SBS I vs. Control	Flavobacterium sp. 20NA77.7	Parasphingorhabdus sp. SCSIO 66989	-0.3498	0.004	0.892940731	NA	NA	NA	Bacteria	Bacteria	intra
SBS I vs. Control	Flavobacterium sp. GSB-24	Protaetiibacter intestinalis	-0.3854	0.001	0.892940731	NA	NA	NA	Bacteria	Bacteria	intra
SBS I vs. Control	Flavobacterium sp. GSB-24	Streptococcus sp. zg-86	-0.3151	0.001	0.892940731	NA	NA	NA	Bacteria	Bacteria	intra
SBS I vs. Control	Flavobacterium sp. IMCC34852	Microbacterium sp. H1-D42	-0.2584	0.004	0.892940731	NA	NA	NA	Bacteria	Bacteria	intra
SBS I vs. Control	Flavobacterium sp. IMCC34852	Streptomyces sp. 891-h	-0.2996	0.001	0.892940731	NA	NA	NA	Bacteria	Bacteria	intra
SBS I vs. Control	Flavobacterium sp. KBS0721	Rossellomorea sp. y25	0.3793	0.004	0.892940731	NA	NA	NA	Bacteria	Bacteria	intra

SBS I vs. Control	Flavobacterium sp. N2038	Microbacterium sp. NIBRBAC000506063	0.2892	0.004	0.892940731	NA	NA	NA	Bacteria	Bacteria	intra
SBS I vs. Control	Flavobacterium sp. N2038	Microterricola viridarii	0.2735	0.003	0.892940731	NA	NA	NA	Bacteria	Bacteria	intra
SBS I vs. Control	Flavonifractor plautii	Schizosaccharomyces japonicus	-0.3544	0.002	0.892940731	NA	NA	NA	Bacteria	Bacteria	intra
SBS I vs. Control	Flintibacter sp. KGMB00164	Photobacterium leiognathi	0.2533	0.003	0.892940731	NA	NA	NA	Bacteria	Bacteria	intra
SBS I vs. Control	Flintibacter sp. KGMB00164	Scandinavium goeteborgense	-0.2824	0.004	0.892940731	NA	NA	NA	Bacteria	Bacteria	intra
SBS I vs. Control	Flintibacter sp. KGMB00164	Trichlorobacter lovleyi	-0.2615	0.003	0.892940731	NA	NA	NA	Bacteria	Bacteria	intra
SBS I vs. Control	Fluviicola taffensis	Paeniglutamicibacter sp. Y32M11	-0.2516	0.003	0.892940731	NA	NA	NA	Bacteria	Bacteria	intra
SBS I vs. Control	Fuerstiella marisgermanici	Paenibacillus elgii	-0.2949	0.001	0.892940731	NA	NA	NA	Bacteria	Bacteria	intra
SBS I vs. Control	Fuerstiella marisgermanici	Rhodocytophaga rosea	0.428	0.001	0.892940731	NA	NA	NA	Bacteria	Bacteria	intra
SBS I vs. Control	Fuerstiella marisgermanici	Streptococcus sp. DTU_2020_1001019_1_SI_AUS_MUR_006	0.3375	0.003	0.892940731	NA	NA	NA	Bacteria	Bacteria	intra
SBS I vs. Control	Fusarium vanettenii	Micromonospora sp. HUAS LYJ1	-0.2856	0.003	0.892940731	NA	NA	NA	Fungi	Bacteria	inter
SBS I vs. Control	Fusobacterium hwasookii	Gemella morbillorum	0.2839	0.003	0.892940731	NA	NA	NA	Bacteria	Bacteria	intra
SBS I vs. Control	Fusobacterium pseudoperiodonticum	Kosakonia cowanii	-0.3303	0.002	0.892940731	NA	NA	NA	Bacteria	Bacteria	intra
SBS I vs. Control	Fusobacterium pseudoperiodonticum	Paenibacillus naphthalenovorans	0.2673	0.002	0.892940731	NA	NA	NA	Bacteria	Bacteria	intra
SBS I vs. Control	Gallionella capsiferriformans	Trinickia caryophylli	-0.2832	0.002	0.892940731	NA	NA	NA	Bacteria	Bacteria	intra
SBS I vs. Control	Gemella morbillorum	Pseudomonas fuscovaginae	-0.3031	0.002	0.892940731	NA	NA	NA	Bacteria	Bacteria	intra
SBS I vs. Control	Gemella sanguinis	Paenibacillus sp. FSL H3-0469	0.2531	0.001	0.892940731	NA	NA	NA	Bacteria	Bacteria	intra
SBS I vs. Control	Geomonas subterranea	Lautropia mirabilis	-0.2566	0.002	0.892940731	NA	NA	NA	Bacteria	Bacteria	intra
SBS I vs. Control	Georgenia sp. M64	Streptococcus sp. oral taxon 061	0.2839	0.003	0.892940731	NA	NA	NA	Bacteria	Bacteria	intra
SBS I vs. Control	Georgenia sp. M64	Trueperella abortisuis	-0.3864	0.002	0.892940731	NA	NA	NA	Bacteria	Bacteria	intra

SBS I vs. Control	Georgenia wutianyii	Natrinema altunense	0.2951	0.003	0.892940731	NA	NA	NA	Bacteria	Bacteria	intra
SBS I vs. Control	Georgenia yuyongxinii	Lysobacter lycopersici	-0.3001	0.004	0.892940731	NA	NA	NA	Bacteria	Bacteria	intra
SBS I vs. Control	Georgenia yuyongxinii	Opitutus sp. GAS368	-0.2892	0.002	0.892940731	NA	NA	NA	Bacteria	Bacteria	intra
SBS I vs. Control	Georgenia yuyongxinii	Symbiopectobacterium purcellii	-0.3039	0.003	0.892940731	NA	NA	NA	Bacteria	Bacteria	intra
SBS I vs. Control	Geothrix sp. 21YS21S-2	Lachnoanaerobaculum umeaense	0.274	0.002	0.892940731	NA	NA	NA	Bacteria	Bacteria	intra
SBS I vs. Control	Geothrix sp. 21YS21S-2	Paucibacter sp. S2-9	0.3469	0.002	0.892940731	NA	NA	NA	Bacteria	Bacteria	intra
SBS I vs. Control	Geothrix sp. 21YS21S-2	Rhizosphaericola mali	-0.3573	0.001	0.892940731	NA	NA	NA	Bacteria	Bacteria	intra
SBS I vs. Control	Geothrix sp. PMB-07	Mycobacterium sp. ELW1	0.2532	0.001	0.892940731	NA	NA	NA	Bacteria	Bacteria	intra
SBS I vs. Control	Gimesia chilikensis	Mesorhizobium sp. L-2-11	0.251	0.003	0.892940731	NA	NA	NA	Bacteria	Bacteria	intra
SBS I vs. Control	Glaciihabitans sp. INWT7	Haladaptatus sp. DJG-WS-42	-0.3246	0.002	0.892940731	NA	NA	NA	Bacteria	Archaea	inter
SBS I vs. Control	Gluconobacter thailandicus	Microlunatus sagamiharensis	0.2611	0.002	0.892940731	NA	NA	NA	Bacteria	Bacteria	intra
SBS I vs. Control	Gluconobacter thailandicus	Schaalia sp. ZJ405	0.2831	0.002	0.892940731	NA	NA	NA	Bacteria	Bacteria	intra
SBS I vs. Control	Gluconobacter thailandicus	Streptococcus sp. Marseille-Q6470	-0.3129	0.002	0.892940731	NA	NA	NA	Bacteria	Bacteria	intra
SBS I vs. Control	Gluconobacter thailandicus	Streptomyces calvus	-0.3328	0.001	0.892940731	NA	NA	NA	Bacteria	Bacteria	intra
SBS I vs. Control	Glutamicibacter creatinolyticus	Nocardiopsis alba	-0.256	0.001	0.892940731	NA	NA	NA	Bacteria	Bacteria	intra
SBS I vs. Control	Glutamicibacter creatinolyticus	Streptomyces sp. ITFR-21	-0.2738	0.004	0.892940731	NA	NA	NA	Bacteria	Bacteria	intra
SBS I vs. Control	Glutamicibacter mishrai	Microbacterium fandaimingii	0.2622	0.003	0.892940731	NA	NA	NA	Bacteria	Bacteria	intra
SBS I vs. Control	Glutamicibacter mishrai	Vibrio vulnificus	-0.3514	0.004	0.892940731	NA	NA	NA	Bacteria	Bacteria	intra
SBS I vs. Control	Gordonia hydrophobica	Rhodococcus triatomae	-0.2634	0.001	0.892940731	NA	NA	NA	Bacteria	Bacteria	intra
SBS I vs. Control	Gordonia hydrophobica	Sporolactobacillus terrae	0.2954	0.003	0.892940731	NA	NA	NA	Bacteria	Bacteria	intra
SBS I vs.	Gordonia hydrophobica	Streptococcus oralis	-0.3155	0.001	0.892940731	NA	NA	NA	Bacteria	Bacteria	intra

Control											
SBS I vs. Control	Gordonia hydrophobica	Thermus aquaticus	0.3098	0.002	0.892940731	NA	NA	NA	Bacteria	Bacteria	intra
SBS I vs. Control	Gordonia sp. PP30	Heterobasidion irregulare	-0.445	0.003	0.892940731	NA	NA	NA	Bacteria	Bacteria	intra
SBS I vs. Control	Gordonia sp. PP30	Marispirochaeta sp.	0.2713	0.003	0.892940731	NA	NA	NA	Bacteria	Bacteria	intra
SBS I vs. Control	Gordonibacter pamelaeae	Streptomyces sp. NBC_01224	-0.2812	0.004	0.892940731	NA	NA	NA	Bacteria	Bacteria	intra
SBS I vs. Control	Gryllotalpicola protaetiae	Hyaloscypha bicolor	0.4005	0.004	0.892940731	NA	NA	NA	Bacteria	Bacteria	intra
SBS I vs. Control	Gryllotalpicola protaetiae	Nocardia puris	0.2644	0.002	0.892940731	NA	NA	NA	Bacteria	Bacteria	intra
SBS I vs. Control	Gudongella oleilytica	Janthinobacterium sp. J1-1	-0.2724	0.004	0.892940731	NA	NA	NA	Bacteria	Bacteria	intra
SBS I vs. Control	Gudongella oleilytica	Mycolicibacterium sp. MU0053	-0.2939	0.004	0.892940731	NA	NA	NA	Bacteria	Bacteria	intra
SBS I vs. Control	Gudongella oleilytica	Streptomyces tubbatahanensis	-0.2541	0.004	0.892940731	NA	NA	NA	Bacteria	Bacteria	intra
SBS I vs. Control	Gulosibacter sediminis	Piscinibacter gummiphilus	-0.3032	0.002	0.892940731	NA	NA	NA	Bacteria	Bacteria	intra
SBS I vs. Control	Gymnodinialimonas ceratoperidinii	Streptomyces chartreusis	-0.2658	0.001	0.892940731	NA	NA	NA	Bacteria	Bacteria	intra
SBS I vs. Control	Haematobacter massiliensis	Methylocystis rosea	-0.4394	0.002	0.892940731	NA	NA	NA	Bacteria	Bacteria	intra
SBS I vs. Control	Haemophilus parahaemolyticus	Mycolicibacterium fluoranthenivorans	0.2558	0.001	0.892940731	NA	NA	NA	Bacteria	Bacteria	intra
SBS I vs. Control	Haemophilus parahaemolyticus	Spirillospora sp. NBC_00431	0.2537	0.001	0.892940731	NA	NA	NA	Bacteria	Bacteria	intra
SBS I vs. Control	Haemophilus parainfluenzae	Klebsiella pasteurii	0.2561	0.003	0.892940731	NA	NA	NA	Bacteria	Bacteria	intra
SBS I vs. Control	Haemophilus parainfluenzae	Luteimonas sp. YGD11-2	-0.259	0.001	0.892940731	NA	NA	NA	Bacteria	Bacteria	intra
SBS I vs. Control	Haemophilus parainfluenzae	Marispirochaeta sp.	0.3191	0.002	0.892940731	NA	NA	NA	Bacteria	Bacteria	intra
SBS I vs. Control	Haemophilus parainfluenzae	Nitrospira japonica	-0.264	0.003	0.892940731	NA	NA	NA	Bacteria	Bacteria	intra
SBS I vs. Control	Haemophilus pittmaniae	Micromonospora sp. NBC_00330	0.3593	0.004	0.892940731	NA	NA	NA	Bacteria	Bacteria	intra
SBS I vs. Control	Haemophilus pittmaniae	Streptomyces sp. 891-h	0.2657	0.003	0.892940731	NA	NA	NA	Bacteria	Bacteria	intra

SBS I vs. Control	Haemophilus sp. oral taxon 036	Luteipulveratus mongoliensis	-0.3013	0.004	0.892940731	NA	NA	NA	Bacteria	Bacteria	intra
SBS I vs. Control	Haemophilus sp. oral taxon 036	Sphingobium sp. TKS	-0.3125	0.004	0.892940731	NA	NA	NA	Bacteria	Bacteria	intra
SBS I vs. Control	Haladaptatus sp. DJG-WS-42	Microbacterium schleiferi	0.2515	0.003	0.892940731	NA	NA	NA	Archaea	Bacteria	inter
SBS I vs. Control	Halarcobacter sp.	Peribacillus sp. R9-11	0.3947	0.0048	0.892940731	NA	NA	NA	Archaea	Bacteria	inter
SBS I vs. Control	Haliangium ochraceum	Kribbella qitaiheensis	-0.3229	0.004	0.892940731	NA	NA	NA	Archaea	Bacteria	inter
SBS I vs. Control	Haloarchaeobius sp. HME9146	Labrenzia sp. VG12	-0.2608	0.004	0.892940731	NA	NA	NA	Archaea	Bacteria	inter
SBS I vs. Control	Halobacteroides halobius	Pasteurella skyensis	0.2852	0.001	0.892940731	NA	NA	NA	Bacteria	Bacteria	intra
SBS I vs. Control	Halococcus qingdaonensis	Tannockella kyphosi	0.2579	0.003	0.892940731	NA	NA	NA	Archaea	Bacteria	inter
SBS I vs. Control	Halococcus sp. PRR34	Rathayibacter tritici	0.2641	0.002	0.892940731	NA	NA	NA	Archaea	Bacteria	inter
SBS I vs. Control	Halogeometricum sp. S1BR25-6	Luteolibacter luteus	-0.3723	0.001	0.892940731	NA	NA	NA	Archaea	Bacteria	inter
SBS I vs. Control	Halogeometricum sp. S1BR25-6	Mesoterricola silvestris	0.4001	0.003	0.892940731	NA	NA	NA	Archaea	Bacteria	inter
SBS I vs. Control	Halogeometricum sp. S1BR25-6	Micromonospora sp. WMMD882	-0.2601	0.002	0.892940731	NA	NA	NA	Archaea	Bacteria	inter
SBS I vs. Control	Halogranum amylolyticum	Streptococcus sp. oral taxon 431	-0.2731	0.004	0.892940731	NA	NA	NA	Archaea	Bacteria	inter
SBS I vs. Control	Halolamina rubra	Microbacterium sp. 1S1	-0.2908	0.001	0.892940731	NA	NA	NA	Archaea	Bacteria	inter
SBS I vs. Control	Halolamina rubra	Parafannyhessea umbonata	0.2991	0.002	0.892940731	NA	NA	NA	Archaea	Bacteria	inter
SBS I vs. Control	Halomonas alkalicola	Penicillium waksmanii	-0.2927	0.003	0.892940731	NA	NA	NA	Bacteria	Bacteria	intra
SBS I vs. Control	Halomonas alkalicola	Streptomyces actuosus	-0.3062	0.001	0.892940731	NA	NA	NA	Bacteria	Bacteria	intra
SBS I vs. Control	Halomonas alkalicola	Thermospira aquatica	-0.2507	0.004	0.892940731	NA	NA	NA	Bacteria	Bacteria	intra
SBS I vs. Control	Halomonas alkalicola	Umbelopsis ramanniana	-0.2788	0.004	0.892940731	NA	NA	NA	Bacteria	Bacteria	intra
SBS I vs. Control	Halomonas alkalicola	Vibrio anguillarum	0.2592	0.003	0.892940731	NA	NA	NA	Bacteria	Bacteria	intra
SBS I vs.	Halomonas sp. 3H	Nocardioides sp. JQ2195	-0.2564	0.004	0.892940731	NA	NA	NA	Bacteria	Bacteria	intra

Control											
SBS I vs. Control	Halomonas sp. 3H	Streptomyces sp. HUAS 2-6	0.3069	0.001	0.892940731	NA	NA	NA	Bacteria	Bacteria	intra
SBS I vs. Control	Halomonas sp. CKK8	Mycobacterium simiae	-0.3166	0.002	0.892940731	NA	NA	NA	Bacteria	Bacteria	intra
SBS I vs. Control	Halomonas sp. CKK8	Pelagerythrobacter marinus	-0.319	0.001	0.892940731	NA	NA	NA	Bacteria	Bacteria	intra
SBS I vs. Control	Halomonas sp. CKK8	Streptococcus phage PH15	0.2821	0.001	0.892940731	NA	NA	NA	Bacteria	Bacteria	intra
SBS I vs. Control	Halomonas sp. XH26	Heterobasidion irregulare	0.4352	0.001	0.892940731	NA	NA	NA	Bacteria	Bacteria	intra
SBS I vs. Control	Halomonas sp. XH26	Rathayibacter rathayi	-0.3706	0.001	0.892940731	NA	NA	NA	Bacteria	Bacteria	intra
SBS I vs. Control	Haloplanus sp. HW8-1	Sulfuritortus calidifontis	0.4455	0.004	0.892940731	NA	NA	NA	Archaea	Bacteria	inter
SBS I vs. Control	Halopseudomonas nanhaiensis	Rathayibacter tritici	0.2598	0.003	0.892940731	NA	NA	NA	Bacteria	Bacteria	intra
SBS I vs. Control	Halopseudomonas nanhaiensis	Sutcliffiella horikoshii	-0.3518	0.002	0.892940731	NA	NA	NA	Bacteria	Bacteria	intra
SBS I vs. Control	Halopseudomonas nanhaiensis	Variovorax paradoxus	0.2916	0.003	0.892940731	NA	NA	NA	Bacteria	Bacteria	intra
SBS I vs. Control	Halopseudomonas sp. SMJS2	Lysobacter capsici	0.3148	0.003	0.892940731	NA	NA	NA	Bacteria	Bacteria	intra
SBS I vs. Control	Halopseudomonas sp. SMJS2	Pedobacter riviphilus	0.3298	0.001	0.892940731	NA	NA	NA	Bacteria	Bacteria	intra
SBS I vs. Control	Halorubrum ezzemoulense	Microbacterium chengjingii	0.2615	0.003	0.892940731	NA	NA	NA	Archaea	Bacteria	inter
SBS I vs. Control	Haloterrigena sp. KLK7	Natronobiforma cellulositropha	0.336	0.003	0.892940731	NA	NA	NA	Archaea	Bacteria	inter
SBS I vs. Control	Halovivax ruber	Stieleria neptunia	-0.3094	0.002	0.892940731	NA	NA	NA	Bacteria	Bacteria	intra
SBS I vs. Control	Halovivax ruber	Streptococcus gordonii	0.2907	0.004	0.892940731	NA	NA	NA	Bacteria	Bacteria	intra
SBS I vs. Control	Hartmannibacter diazotrophicus	Pseudodesulfovibrio sp. zrk46	-0.3278	0.002	0.892940731	NA	NA	NA	Bacteria	Bacteria	intra
SBS I vs. Control	Hartmannibacter diazotrophicus	Tessaracoccus sp. MC1865	0.3103	0.002	0.892940731	NA	NA	NA	Bacteria	Bacteria	intra
SBS I vs. Control	Hartmannibacter diazotrophicus	uncultured Desulfosarcina sp	-0.2968	0.001	0.892940731	NA	NA	NA	Bacteria	Bacteria	intra
SBS I vs. Control	Helicobacter pylori	Herbaspirillum frisingense	-0.3635	0.002	0.892940731	NA	NA	NA	Bacteria	Bacteria	intra

SBS I vs. Control	Herbaspirillum frisingense	Leisingera thetidis	0.2942	0.004	0.892940731	NA	NA	NA	Bacteria	Bacteria	intra
SBS I vs. Control	Herbaspirillum frisingense	Streptomyces olivoreticuli	0.3098	0.004	0.892940731	NA	NA	NA	Bacteria	Bacteria	intra
SBS I vs. Control	Heterobasidion irregulare	Streptomyces sp. WZ-12	0.3153	0.002	0.892940731	NA	NA	NA	Bacteria	Bacteria	intra
SBS I vs. Control	Hirsutella rhossiliensis	Kineothrix sp. MB12-C1	-0.3239	0.002	0.892940731	NA	NA	NA	Bacteria	Bacteria	intra
SBS I vs. Control	Hirsutella rhossiliensis	Nocardia sp. NBC_01730	-0.3021	0.001	0.892940731	NA	NA	NA	Bacteria	Bacteria	intra
SBS I vs. Control	Hyaloscypha bicolor	Pedobacter ginsengisoli	-0.4298	0.001	0.892940731	NA	NA	NA	Bacteria	Bacteria	intra
SBS I vs. Control	Hydrogenibacillus sp. N12	Parvimonas micra	-0.2886	0.002	0.892940731	NA	NA	NA	Bacteria	Bacteria	intra
SBS I vs. Control	Hydrogenophaga sp. BPS33	Mycobacterium sp. MS1601	-0.2634	0.003	0.892940731	NA	NA	NA	Bacteria	Bacteria	intra
SBS I vs. Control	Hydrogenophaga sp. PAMC20947	Streptococcus phage PH10	-0.2977	0.003	0.892940731	NA	NA	NA	Bacteria	Bacteria	intra
SBS I vs. Control	Hymenobacter sp. 5317J-9	Jannaschia sp. GRR-S6-38	-0.4563	0.002	0.892940731	NA	NA	NA	Bacteria	Bacteria	intra
SBS I vs. Control	Hymenobacter sp. 5317J-9	Stenotrophomonas sp. Pemsol	0.2629	0.003	0.892940731	NA	NA	NA	Bacteria	Bacteria	intra
SBS I vs. Control	Intestinibacillus sp. NTUH-41-i26	Microbacterium sp. YJN-G	-0.2645	0.004	0.892940731	NA	NA	NA	Bacteria	Bacteria	intra
SBS I vs. Control	Intestinibacillus sp. NTUH-41-i26	Mycolicibacterium sp. TUM20985	-0.2945	0.002	0.892940731	NA	NA	NA	Bacteria	Bacteria	intra
SBS I vs. Control	Intestinimonas butyriciproducens	Lactobacillus crispatus	-0.2831	0.004	0.892940731	NA	NA	NA	Bacteria	Bacteria	intra
SBS I vs. Control	Intestinimonas butyriciproducens	Salinibacter ruber	-0.2814	0.001	0.892940731	NA	NA	NA	Bacteria	Bacteria	intra
SBS I vs. Control	Isoptericola variabilis	Rothia dentocariosa	0.2753	0.002	0.892940731	NA	NA	NA	Bacteria	Bacteria	intra
SBS I vs. Control	Janibacter cremeus	Lelliottia sp. WB101	-0.3521	0.002	0.892940731	NA	NA	NA	Bacteria	Bacteria	intra
SBS I vs. Control	Janibacter cremeus	Mycobacterium simiae	0.2508	0.004	0.892940731	NA	NA	NA	Bacteria	Bacteria	intra
SBS I vs. Control	Janibacter cremeus	Thermoanaerobacterium thermosaccharolyticum	-0.3391	0.001	0.892940731	NA	NA	NA	Bacteria	Bacteria	intra
SBS I vs. Control	Janibacter sp. A1S7	Variovorax sp. RKNM96	0.273	0.002	0.892940731	NA	NA	NA	Bacteria	Bacteria	intra
SBS I vs.	Janibacter sp. A1S7	Verminephrobacter eiseniae	0.3133	0.003	0.892940731	NA	NA	NA	Bacteria	Bacteria	intra

Control											
SBS I vs. Control	Janibacter terrae	Treponema vincentii	-0.3288	0.001	0.892940731	NA	NA	NA	Bacteria	Bacteria	intra
SBS I vs. Control	Jannaschia sp. GRR-S6-38	Klebsiella electrica	-0.2912	0.001	0.892940731	NA	NA	NA	Bacteria	Bacteria	intra
SBS I vs. Control	Jannaschia sp. GRR-S6-38	Penicillium soppii	-0.2602	0.003	0.892940731	NA	NA	NA	Bacteria	Bacteria	intra
SBS I vs. Control	Jannaschia sp. GRR-S6-38	Pseudorhizobium flavum	0.2504	0.004	0.892940731	NA	NA	NA	Bacteria	Bacteria	intra
SBS I vs. Control	Janthinobacterium sp. J1-1	Streptococcus sp. HN38	0.3678	0.002	0.892940731	NA	NA	NA	Bacteria	Bacteria	intra
SBS I vs. Control	Jeongeupia sp. USM3	Pseudomonas fluorescens	-0.2728	0.001	0.892940731	NA	NA	NA	Bacteria	Bacteria	intra
SBS I vs. Control	Jiangella alkaliphila	Salaquimonas pukyongi	-0.2926	0.003	0.892940731	NA	NA	NA	Bacteria	Bacteria	intra
SBS I vs. Control	Jiangella sp. DSM 45060	Pararhodospirillum photometricum	-0.2985	0.003	0.892940731	NA	NA	NA	Bacteria	Bacteria	intra
SBS I vs. Control	Kaustia mangrovi	Massilia endophytica	0.372	0.001	0.892940731	NA	NA	NA	Bacteria	Bacteria	intra
SBS I vs. Control	Kaustia mangrovi	Micromonospora sp. WMMA1998	0.4128	0.004	0.892940731	NA	NA	NA	Bacteria	Bacteria	intra
SBS I vs. Control	Kaustia mangrovi	Runella sp. SP2	0.3141	0.002	0.892940731	NA	NA	NA	Bacteria	Bacteria	intra
SBS I vs. Control	Kaustia mangrovi	Saccharopolyspora gloriosae	-0.2684	0.004	0.892940731	NA	NA	NA	Bacteria	Bacteria	intra
SBS I vs. Control	Kickxella alabastrina	Serratia ficaria	-0.2595	0.001	0.892940731	NA	NA	NA	Bacteria	Bacteria	intra
SBS I vs. Control	Kineococcus sp. NBC_00420	Proteus mirabilis	-0.3352	0.003	0.892940731	NA	NA	NA	Bacteria	Bacteria	intra
SBS I vs. Control	Kineococcus sp. NBC_00420	Thermococcus sp. Bubb.Bath	-0.3742	0.003	0.892940731	NA	NA	NA	Bacteria	Bacteria	intra
SBS I vs. Control	Kineothrix sp. MB12-C1	Streptomyces bathyalis	0.2859	0.002	0.892940731	NA	NA	NA	Bacteria	Bacteria	intra
SBS I vs. Control	Kingella denitrificans	Limosilactobacillus oris	-0.2857	0.004	0.892940731	NA	NA	NA	Bacteria	Bacteria	intra
SBS I vs. Control	Kitasatospora aureofaciens	Magnetospirillum sp. XM-1	-0.2783	0.002	0.892940731	NA	NA	NA	Bacteria	Bacteria	intra
SBS I vs. Control	Kitasatospora aureofaciens	Pseudoduganella flava	-0.3013	0.001	0.892940731	NA	NA	NA	Bacteria	Bacteria	intra
SBS I vs. Control	Kitasatospora fiedleri	Rothia dentocariosa	0.3334	0.001	0.892940731	NA	NA	NA	Bacteria	Bacteria	intra

SBS I vs. Control	Kitasatospora fiedleri	Salinimonas sediminis	-0.2813	0.003	0.892940731	NA	NA	NA	Bacteria	Bacteria	intra
SBS I vs. Control	Kitasatospora fiedleri	Verminephrobacter eiseniae	-0.272	0.003	0.892940731	NA	NA	NA	Bacteria	Bacteria	intra
SBS I vs. Control	Kitasatospora sp. HUAS 3-15	Streptococcus sp. FSL W8-0197	-0.27	0.002	0.892940731	NA	NA	NA	Bacteria	Bacteria	intra
SBS I vs. Control	Kitasatospora sp. HUAS 3-15	Streptomyces sp. NBC_01296	0.2755	0.001	0.892940731	NA	NA	NA	Bacteria	Bacteria	intra
SBS I vs. Control	Kitasatospora sp. YST-16	Rhodocytophaga rosea	-0.252	0.003	0.892940731	NA	NA	NA	Bacteria	Bacteria	intra
SBS I vs. Control	Kitasatospora sp. YST-16	Salinibacterium sp. ZJ70	0.2685	0.003	0.892940731	NA	NA	NA	Bacteria	Bacteria	intra
SBS I vs. Control	Klebsiella africana	Klebsiella electrica	0.313	0.002	0.892940731	NA	NA	NA	Bacteria	Bacteria	intra
SBS I vs. Control	Klebsiella africana	Paenibacillus macerans	-0.2731	0.001	0.892940731	NA	NA	NA	Bacteria	Bacteria	intra
SBS I vs. Control	Klebsiella africana	Streptococcus gwangjuense	0.2977	0.001	0.892940731	NA	NA	NA	Bacteria	Bacteria	intra
SBS I vs. Control	Klebsiella electrica	Microterricola viridarii	0.268	0.003	0.892940731	NA	NA	NA	Bacteria	Bacteria	intra
SBS I vs. Control	Klebsiella electrica	Sphingobium sp. TKS	0.2868	0.003	0.892940731	NA	NA	NA	Bacteria	Bacteria	intra
SBS I vs. Control	Klebsiella electrica	Streptomyces sp. NBC_01450	0.3985	0.001	0.892940731	NA	NA	NA	Bacteria	Bacteria	intra
SBS I vs. Control	Klebsiella huaxiensis	Leucobacter insecticola	-0.2834	0.002	0.892940731	NA	NA	NA	Bacteria	Bacteria	intra
SBS I vs. Control	Klebsiella huaxiensis	Parasphingorhabdus sp. SCSIO 66989	-0.2535	0.002	0.892940731	NA	NA	NA	Bacteria	Bacteria	intra
SBS I vs. Control	Klebsiella huaxiensis	Streptomyces olivoreticuli	0.3859	0.003	0.892940731	NA	NA	NA	Bacteria	Bacteria	intra
SBS I vs. Control	Klebsiella michiganensis	Trichoderma citrinoviride	-0.2659	0.002	0.892940731	NA	NA	NA	Bacteria	Bacteria	intra
SBS I vs. Control	Klebsiella oxytoca	Kosakonia oryzae	-0.3777	0.001	0.892940731	NA	NA	NA	Bacteria	Bacteria	intra
SBS I vs. Control	Klebsiella oxytoca	Planococcus sp. MB-3u-03	0.3492	0.003	0.892940731	NA	NA	NA	Bacteria	Bacteria	intra
SBS I vs. Control	Klebsiella sp. A52	Mucilaginibacter rubeus	0.4065	0.001	0.892940731	NA	NA	NA	Bacteria	Bacteria	intra
SBS I vs. Control	Klebsiella sp. A52	Pseudanabaena sp. ABRG5-3	-0.2579	0.002	0.892940731	NA	NA	NA	Bacteria	Bacteria	intra
SBS I vs.	Klebsiella sp. A52	Sporomusa termitida	-0.2672	0.002	0.892940731	NA	NA	NA	Bacteria	Bacteria	intra

Control											
SBS I vs. Control	Klebsiella sp. WP3-S18-ESBL-05	Microvirga sp. VF16	-0.2558	0.002	0.892940731	NA	NA	NA	Bacteria	Bacteria	intra
SBS I vs. Control	Klebsiella sp. WP3-S18-ESBL-05	Streptococcus sp. Marseille-Q6470	0.3044	0.001	0.892940731	NA	NA	NA	Bacteria	Bacteria	intra
SBS I vs. Control	Klebsiella sp. WP3-S18-ESBL-05	uncultured Methanobrevibacter sp	-0.2895	0.001	0.892940731	NA	NA	NA	Bacteria	Bacteria	intra
SBS I vs. Control	Kocuria flava	Pseudomonas fuscovaginae	0.2641	0.002	0.892940731	NA	NA	NA	Bacteria	Bacteria	intra
SBS I vs. Control	Kocuria palustris	Wenyingzhuangia fucanilytica	-0.3428	0.002	0.892940731	NA	NA	NA	Bacteria	Bacteria	intra
SBS I vs. Control	Kocuria rhizophila	Mycolicibacter terrae	0.2533	0.004	0.892940731	NA	NA	NA	Bacteria	Bacteria	intra
SBS I vs. Control	Kocuria rosea	Streptomyces sp. NBC_00536	0.2984	0.002	0.892940731	NA	NA	NA	Bacteria	Bacteria	intra
SBS I vs. Control	Kocuria rosea	Thermococcus sp. MAR1	0.3518	0.003	0.892940731	NA	NA	NA	Bacteria	Bacteria	intra
SBS I vs. Control	Kocuria rosea	[Ruminococcus] torques	-0.2943	0.002	0.892940731	NA	NA	NA	Bacteria	Bacteria	intra
SBS I vs. Control	Kocuria turfanensis	Microcella sp.	-0.3887	0.002	0.892940731	NA	NA	NA	Bacteria	Bacteria	intra
SBS I vs. Control	Kocuria turfanensis	Pseudomonas oryzihabitans	-0.3261	0.003	0.892940731	NA	NA	NA	Bacteria	Bacteria	intra
SBS I vs. Control	Kocuria turfanensis	Sporolactobacillus terrae	-0.2748	0.001	0.892940731	NA	NA	NA	Bacteria	Bacteria	intra
SBS I vs. Control	Kocuria turfanensis	Tessaracoccus sp. MC1865	0.254	0.003	0.892940731	NA	NA	NA	Bacteria	Bacteria	intra
SBS I vs. Control	Kocuria varians	Treponema socranskii	0.3082	0.002	0.892940731	NA	NA	NA	Bacteria	Bacteria	intra
SBS I vs. Control	Kosakonia cowanii	Peptoniphilus sp. SAHP1	-0.3967	0.002	0.892940731	NA	NA	NA	Bacteria	Bacteria	intra
SBS I vs. Control	Kosakonia cowanii	Pseudobutyrivibrio xylanivorans	-0.2652	0.004	0.892940731	NA	NA	NA	Bacteria	Bacteria	intra
SBS I vs. Control	Kosakonia cowanii	Pseudomonas gozinkensis	0.2555	0.004	0.892940731	NA	NA	NA	Bacteria	Bacteria	intra
SBS I vs. Control	Kosakonia cowanii	Salipiger profundus	0.2559	0.001	0.892940731	NA	NA	NA	Bacteria	Bacteria	intra
SBS I vs. Control	Kosakonia oryzae	Lacrimispora saccharolytica	0.3887	0.003	0.892940731	NA	NA	NA	Bacteria	Bacteria	intra
SBS I vs. Control	Kosakonia radicincitans	Mesorhizobium sp. M1D.F.Ca.ET.043.01.1.1	0.287	0.004	0.892940731	NA	NA	NA	Bacteria	Bacteria	intra

SBS I vs. Control	Kovacikia minuta	Sphingopyxis indica	0.3323	0.002	0.892940731	NA	NA	NA	Bacteria	Bacteria	intra
SBS I vs. Control	Kovacikia minuta	Streptococcus constellatus	-0.4198	0.001	0.892940731	NA	NA	NA	Bacteria	Bacteria	intra
SBS I vs. Control	Kribbella flavida	Microterricola viridarii	0.2804	0.004	0.892940731	NA	NA	NA	Bacteria	Bacteria	intra
SBS I vs. Control	Kribbella flavida	Pantoea vagans	-0.3033	0.001	0.892940731	NA	NA	NA	Bacteria	Bacteria	intra
SBS I vs. Control	Kribbella flavida	Pararhodospirillum photometricum	0.3828	0.002	0.892940731	NA	NA	NA	Bacteria	Bacteria	intra
SBS I vs. Control	Kribbella flavida	Streptomyces sp. NBC_00243	-0.3148	0.001	0.892940731	NA	NA	NA	Bacteria	Bacteria	intra
SBS I vs. Control	Kribbella sp. NBC_01245	Paenibacillus tritici	0.2553	0.004	0.892940731	NA	NA	NA	Bacteria	Bacteria	intra
SBS I vs. Control	Kytococcus sedentarius	Thermococcus sp. GR4	0.2676	0.004	0.892940731	NA	NA	NA	Bacteria	Bacteria	intra
SBS I vs. Control	Labrys sp. KNU-23	Streptomyces sp. NBC_01230	-0.3922	0.001	0.892940731	NA	NA	NA	Bacteria	Bacteria	intra
SBS I vs. Control	Lachnoanaerobaculum umeaense	Peterkaempfera bronchialis	-0.3462	0.002	0.892940731	NA	NA	NA	Bacteria	Bacteria	intra
SBS I vs. Control	Lachnoclostridium phocaeense	Streptococcus lactarius	0.2917	0.002	0.892940731	NA	NA	NA	Bacteria	Bacteria	intra
SBS I vs. Control	Lachnoclostridium phytofermentans	Neisseria subflava	-0.269	0.003	0.892940731	NA	NA	NA	Bacteria	Bacteria	intra
SBS I vs. Control	Lachnospira eligens	Rhodoferax ferrireducens	-0.2629	0.004	0.892940731	NA	NA	NA	Bacteria	Bacteria	intra
SBS I vs. Control	Lachnospira eligens	Streptomyces rimosus	-0.2977	0.003	0.892940731	NA	NA	NA	Bacteria	Bacteria	intra
SBS I vs. Control	Lacrimispora saccharolytica	Streptococcus sp. FSL W8-0197	0.2888	0.003	0.892940731	NA	NA	NA	Bacteria	Bacteria	intra
SBS I vs. Control	Lactobacillus johnsonii	Pseudocitrobacter corydidari	-0.3261	0.003	0.892940731	NA	NA	NA	Bacteria	Bacteria	intra
SBS I vs. Control	Lactobacillus johnsonii	Streptomyces aurantiacus	0.346	0.004	0.892940731	NA	NA	NA	Bacteria	Bacteria	intra
SBS I vs. Control	Lactobacillus paragasseri	Micromonospora maris	-0.3428	0.003	0.892940731	NA	NA	NA	Bacteria	Bacteria	intra
SBS I vs. Control	Lactobacillus paragasseri	Mycobacterium sp. ITM-2016-00318	-0.2601	0.001	0.892940731	NA	NA	NA	Bacteria	Bacteria	intra
SBS I vs. Control	Laribacter hongkongensis	Malassezia restricta	-0.2978	0.004	0.892940731	NA	NA	NA	Bacteria	Bacteria	intra
SBS I vs.	Lautropia mirabilis	Nodularia sphaerocarpa	-0.4775	0.002	0.892940731	NA	NA	NA	Bacteria	Bacteria	intra

Control											
SBS I vs. Control	Leclercia pneumoniae	Solobacterium moorei	0.2738	0.002	0.892940731	NA	NA	NA	Bacteria	Bacteria	intra
SBS I vs. Control	Leclercia sp. W6	Streptococcus pseudopneumoniae	0.3409	0.003	0.892940731	NA	NA	NA	Bacteria	Bacteria	intra
SBS I vs. Control	Leisingera sp. S132	[Ruminococcus] torques	-0.3053	0.002	0.892940731	NA	NA	NA	Bacteria	Bacteria	intra
SBS I vs. Control	Lelliottia amnigena	Sulfitobacter sp. S190	0.408	0.003	0.892940731	NA	NA	NA	Bacteria	Bacteria	intra
SBS I vs. Control	Lelliottia sp. WB101	Nitrogeniibacter mangrovi	-0.2623	0.001	0.892940731	NA	NA	NA	Bacteria	Bacteria	intra
SBS I vs. Control	Lentzea guizhouensis	Thermococcus sp. 21S7	-0.2827	0.004	0.892940731	NA	NA	NA	Bacteria	Bacteria	intra
SBS I vs. Control	Lentzea guizhouensis	Verminephrobacter eiseniae	-0.2555	0.002	0.892940731	NA	NA	NA	Bacteria	Bacteria	intra
SBS I vs. Control	Leptospira sp. severe_002	Propionibacterium freudenreichii	-0.256	0.002	0.892940731	NA	NA	NA	Bacteria	Bacteria	intra
SBS I vs. Control	Leptothrix cholodnii	Mesorhizobium sp. AR07	-0.2569	0.004	0.892940731	NA	NA	NA	Bacteria	Bacteria	intra
SBS I vs. Control	Leptotrichia sp. oral taxon 218	Microbacterium sp. M28	0.3619	0.001	0.892940731	NA	NA	NA	Bacteria	Bacteria	intra
SBS I vs. Control	Leptotrichia sp. oral taxon 218	Pseudorhizobium flavum	0.3242	0.003	0.892940731	NA	NA	NA	Bacteria	Bacteria	intra
SBS I vs. Control	Leptotrichia sp. oral taxon 218	Roseburia intestinalis	0.2597	0.002	0.892940731	NA	NA	NA	Bacteria	Bacteria	intra
SBS I vs. Control	Leucobacter insecticola	Niallia sp. Man26	-0.2921	0.002	0.892940731	NA	NA	NA	Bacteria	Bacteria	intra
SBS I vs. Control	Leucobacter insecticola	Streptococcus sp. oral taxon 061	-0.2668	0.004	0.892940731	NA	NA	NA	Bacteria	Bacteria	intra
SBS I vs. Control	Leucobacter insecticola	Streptomyces sp. NBC_01217	-0.3325	0.003	0.892940731	NA	NA	NA	Bacteria	Bacteria	intra
SBS I vs. Control	Leucobacter luti	uncultured Desulfobulbus sp	-0.258	0.002	0.892940731	NA	NA	NA	Bacteria	Bacteria	intra
SBS I vs. Control	Leucobacter muris	Paracoccus pantotrophus	-0.2682	0.003	0.892940731	NA	NA	NA	Bacteria	Bacteria	intra
SBS I vs. Control	Leucobacter rhizosphaerae	Tsuneonella sp. CC-YZS046	-0.4021	0.004	0.892940731	NA	NA	NA	Bacteria	Bacteria	intra
SBS I vs. Control	Leucobacter sp. Psy1	Methylococcus capsulatus	-0.2789	0.003	0.892940731	NA	NA	NA	Bacteria	Bacteria	intra
SBS I vs. Control	Leucobacter sp. Psy1	Variovorax sp. PBL-H6	-0.3419	0.002	0.892940731	NA	NA	NA	Bacteria	Bacteria	intra

SBS I vs. Control	Leucobacter triazinivorans	uncultured Acetobacteroides sp	-0.3264	0.003	0.892940731	NA	NA	NA	Bacteria	Bacteria	intra
SBS I vs. Control	Liberibacter crescens	Rouxiella chamberiensis	0.386	0.001	0.892940731	NA	NA	NA	Bacteria	Bacteria	intra
SBS I vs. Control	Limnochorda pilosa	Pseudoxanthomonas sp. X-1	0.28	0.004	0.892940731	NA	NA	NA	Bacteria	Bacteria	intra
SBS I vs. Control	Limosilactobacillus mucosae	Streptomyces actuosus	-0.3269	0.001	0.892940731	NA	NA	NA	Bacteria	Bacteria	intra
SBS I vs. Control	Limosilactobacillus mucosae	Thermomonospora curvata	-0.2537	0.002	0.892940731	NA	NA	NA	Bacteria	Bacteria	intra
SBS I vs. Control	Limosilactobacillus oris	Thermoanaerobacterium thermosaccharolyticum	-0.2645	0.001	0.892940731	NA	NA	NA	Bacteria	Bacteria	intra
SBS I vs. Control	Liquorilactobacillus hordei	Luteipulveratus mongoliensis	-0.3316	0.003	0.892940731	NA	NA	NA	Bacteria	Bacteria	intra
SBS I vs. Control	Loigolactobacillus coryniformis	Micrococcus terreus	0.2796	0.003	0.892940731	NA	NA	NA	Bacteria	Bacteria	intra
SBS I vs. Control	Loigolactobacillus coryniformis	Stenotrophomonas acidaminiphila	0.2598	0.003	0.892940731	NA	NA	NA	Bacteria	Bacteria	intra
SBS I vs. Control	Luteimonas chenhongjianii	Parvimonas micra	0.2512	0.001	0.892940731	NA	NA	NA	Bacteria	Bacteria	intra
SBS I vs. Control	Luteimonas fraxinea	Ruminococcus champanellensis	0.4154	0.002	0.892940731	NA	NA	NA	Bacteria	Bacteria	intra
SBS I vs. Control	Luteimonas sp. YGD11-2	Marinobacter sp. CP1	-0.3824	0.002	0.892940731	NA	NA	NA	Bacteria	Bacteria	intra
SBS I vs. Control	Luteipulveratus mongoliensis	Rossellomorea sp. y25	0.2998	0.003	0.892940731	NA	NA	NA	Bacteria	Bacteria	intra
SBS I vs. Control	Luteolibacter luteus	Paraburkholderia aromaticivorans	-0.3219	0.003	0.892940731	NA	NA	NA	Bacteria	Bacteria	intra
SBS I vs. Control	Luteolibacter luteus	Sanguibacter sp. HDW7	-0.3344	0.002	0.892940731	NA	NA	NA	Bacteria	Bacteria	intra
SBS I vs. Control	Lutibacter sp. A64	Paenibacillus mucilaginosus	-0.3247	0.004	0.892940731	NA	NA	NA	Bacteria	Bacteria	intra
SBS I vs. Control	Lutibacter sp. A64	Paenibacillus tianjinensis	0.33	0.004	0.892940731	NA	NA	NA	Bacteria	Bacteria	intra
SBS I vs. Control	Lysinibacillus fusiformis	Rothia terrae	-0.3246	0.002	0.892940731	NA	NA	NA	Bacteria	Bacteria	intra
SBS I vs. Control	Lysinibacillus sp. SGAir0095	Mycobacterium sp. MS1601	0.3687	0.001	0.892940731	NA	NA	NA	Bacteria	Bacteria	intra
SBS I vs. Control	Lysinibacillus sp. SGAir0095	Nesterenkonia pannonica	-0.369	0.003	0.892940731	NA	NA	NA	Bacteria	Bacteria	intra
SBS I vs.	Lysinibacillus sp. SGAir0095	Ralstonia sp. RRA.1	0.2533	0.003	0.892940731	NA	NA	NA	Bacteria	Bacteria	intra

Control											
SBS I vs. Control	Lysinibacillus sp. SGAir0095	Roseburia rectibacter	-0.2681	0.001	0.892940731	NA	NA	NA	Bacteria	Bacteria	intra
SBS I vs. Control	Lysinibacillus sp. SGAir0095	Serpula lacrymans	-0.2502	0.001	0.892940731	NA	NA	NA	Bacteria	Bacteria	intra
SBS I vs. Control	Lysinibacter sp. HNR	Pseudarthrobacter sp. NBSH8	0.3341	0.001	0.892940731	NA	NA	NA	Bacteria	Bacteria	intra
SBS I vs. Control	Lysobacter capsici	Salinibacter ruber	0.2594	0.004	0.892940731	NA	NA	NA	Bacteria	Bacteria	intra
SBS I vs. Control	Lysobacter lycopersici	Paenibacillus macerans	0.3887	0.001	0.892940731	NA	NA	NA	Bacteria	Bacteria	intra
SBS I vs. Control	Lysobacter lycopersici	Streptomyces phaeoluteigriseus	-0.3573	0.002	0.892940731	NA	NA	NA	Bacteria	Bacteria	intra
SBS I vs. Control	Lysobacter sp. S4-A87	Mesorhizobium sp. Pch-S	0.2526	0.003	0.892940731	NA	NA	NA	Bacteria	Bacteria	intra
SBS I vs. Control	Lysobacter sp. S4-A87	Microbacterium sp. ET2	0.2901	0.001	0.892940731	NA	NA	NA	Bacteria	Bacteria	intra
SBS I vs. Control	Lysobacter sp. S4-A87	Microlunatus sagamiharensis	0.253	0.001	0.892940731	NA	NA	NA	Bacteria	Bacteria	intra
SBS I vs. Control	Lysobacter sp. S4-A87	Sporosarcina ureae	-0.3128	0.003	0.892940731	NA	NA	NA	Bacteria	Bacteria	intra
SBS I vs. Control	Magnetococcus marinus	Pararhizobium sp. YC-54	-0.2562	0.001	0.892940731	NA	NA	NA	Bacteria	Bacteria	intra
SBS I vs. Control	Mahella australiensis	Psychrobacter sp. WB2	0.5166	0.0003	0.892940731	NA	NA	NA	Bacteria	Bacteria	intra
SBS I vs. Control	Malassezia globosa	Streptococcus sanguinis	-0.2705	0.003	0.892940731	NA	NA	NA	Bacteria	Bacteria	intra
SBS I vs. Control	Malassezia restricta	Microcella indica	0.2916	0.001	0.892940731	NA	NA	NA	Bacteria	Bacteria	intra
SBS I vs. Control	Malassezia restricta	Parazoarcus communis	0.327	0.001	0.892940731	NA	NA	NA	Bacteria	Bacteria	intra
SBS I vs. Control	Malassezia restricta	Sulfitobacter sp. S190	0.2718	0.002	0.892940731	NA	NA	NA	Bacteria	Bacteria	intra
SBS I vs. Control	Mammaliicoccus vitulinus	Rathayibacter sp. VKM Ac-2760	0.338	0.002	0.892940731	NA	NA	NA	Bacteria	Bacteria	intra
SBS I vs. Control	Mammaliicoccus vitulinus	Tatumella ptyseos	0.2963	0.001	0.892940731	NA	NA	NA	Bacteria	Bacteria	intra
SBS I vs. Control	Maricaulis maris	Pseudoduganella sp. SL102	-0.2731	0.002	0.892940731	NA	NA	NA	Bacteria	Bacteria	intra
SBS I vs. Control	Maridesulfovibrio sp.	Miniimonas sp. S16	0.3966	0.001	0.892940731	NA	NA	NA	Bacteria	Bacteria	intra

SBS I vs. Control	Maridesulfovibrio sp.	Nocardia huaxiensis	-0.3184	0.001	0.892940731	NA	NA	NA	Bacteria	Bacteria	intra
SBS I vs. Control	Maridesulfovibrio sp.	Syntrophotalea acetylenica	-0.2563	0.004	0.892940731	NA	NA	NA	Bacteria	Bacteria	intra
SBS I vs. Control	Marinobacter sp. CA1	Mesorhizobium sp. AR07	-0.287	0.002	0.892940731	NA	NA	NA	Bacteria	Bacteria	intra
SBS I vs. Control	Marinobacter sp. CA1	Micromonospora purpureochromogenes	0.3447	0.003	0.892940731	NA	NA	NA	Bacteria	Bacteria	intra
SBS I vs. Control	Marinobacter sp. CA1	Thermomonas brevis	-0.3168	0.004	0.892940731	NA	NA	NA	Bacteria	Bacteria	intra
SBS I vs. Control	Marinobacter sp. CP1	Microbacterium sp. zg-Y818	-0.3312	0.001	0.892940731	NA	NA	NA	Bacteria	Bacteria	intra
SBS I vs. Control	Marinobacter sp. NP-4(2019)	Qiania dongpingensis	-0.2702	0.003	0.892940731	NA	NA	NA	Bacteria	Bacteria	intra
SBS I vs. Control	Marispirochaeta sp.	Stenotrophomonas sp. ESTM1D_MKCIP4_1	-0.2885	0.004	0.892940731	NA	NA	NA	Bacteria	Bacteria	intra
SBS I vs. Control	Marivirga harenae	Mycobacterium colombiense	-0.3631	0.002	0.892940731	NA	NA	NA	Bacteria	Bacteria	intra
SBS I vs. Control	Marivirga harenae	Nocardia puris	-0.2728	0.003	0.892940731	NA	NA	NA	Bacteria	Bacteria	intra
SBS I vs. Control	Massilia endophytica	Streptococcus sp. FSL W8-0197	-0.3337	0.002	0.892940731	NA	NA	NA	Bacteria	Bacteria	intra
SBS I vs. Control	Massilia sp. YMA4	Niallia circulans	0.2965	0.002	0.892940731	NA	NA	NA	Bacteria	Bacteria	intra
SBS I vs. Control	Massilia sp. YMA4	Paenibacillus macerans	0.3355	0.003	0.892940731	NA	NA	NA	Bacteria	Bacteria	intra
SBS I vs. Control	Massilia violaceinigra	Paraburkholderia tropica	0.2971	0.003	0.892940731	NA	NA	NA	Bacteria	Bacteria	intra
SBS I vs. Control	Mediterraneibacter gnavus	Micromonospora sp. NBC_01796	-0.3125	0.004	0.892940731	NA	NA	NA	Bacteria	Bacteria	intra
SBS I vs. Control	Mediterraneibacter gnavus	Mycolicibacterium gadium	0.4757	0.001	0.892940731	NA	NA	NA	Bacteria	Bacteria	intra
SBS I vs. Control	Mediterraneibacter gnavus	Streptococcus sp. 29896	-0.3643	0.001	0.892940731	NA	NA	NA	Bacteria	Bacteria	intra
SBS I vs. Control	Megamonas hypermegale	Vibrio anguillarum	0.2932	0.002	0.892940731	NA	NA	NA	Bacteria	Bacteria	intra
SBS I vs. Control	Mesobacillus sp. AQ2	Metabacillus sediminilitoris	-0.2679	0.003	0.892940731	NA	NA	NA	Bacteria	Bacteria	intra
SBS I vs. Control	Mesorhizobium ciceri	Silicimonas algicola	-0.2926	0.003	0.892940731	NA	NA	NA	Bacteria	Bacteria	intra
SBS I vs.	Mesorhizobium sp. AR07	Streptomyces sp. ITFR-21	-0.3517	0.002	0.892940731	NA	NA	NA	Bacteria	Bacteria	intra

Control											
SBS I vs. Control	Mesorhizobium sp. INR15	Mycotypha africana	0.2597	0.003	0.892940731	NA	NA	NA	Bacteria	Bacteria	intra
SBS I vs. Control	Mesorhizobium sp. INR15	Pseudomonas viridiflava	0.2982	0.003	0.892940731	NA	NA	NA	Bacteria	Bacteria	intra
SBS I vs. Control	Mesorhizobium sp. L-2-11	Paenibacillus elgii	-0.379	0.002	0.892940731	NA	NA	NA	Bacteria	Bacteria	intra
SBS I vs. Control	Mesorhizobium sp. L-2-11	Pseudomonas sp. B21-019	0.355	0.001	0.892940731	NA	NA	NA	Bacteria	Bacteria	intra
SBS I vs. Control	Mesoterricola silvestris	Streptomyces durmitorensis	0.2532	0.004	0.892940731	NA	NA	NA	Bacteria	Bacteria	intra
SBS I vs. Control	Metabacillus sediminilitoris	Paracoccus zhejiangensis	-0.2893	0.003	0.892940731	NA	NA	NA	Bacteria	Bacteria	intra
SBS I vs. Control	Methylobacter sp. YRD-M1	Streptococcus sp. oral taxon 061	0.2542	0.001	0.892940731	NA	NA	NA	Bacteria	Bacteria	intra
SBS I vs. Control	Methylobacterium indicum	Oscillibacter sp. PEA192	0.261	0.004	0.892940731	NA	NA	NA	Bacteria	Bacteria	intra
SBS I vs. Control	Methylobacterium indicum	Pseudomonas oryzihabitans	-0.2706	0.001	0.892940731	NA	NA	NA	Bacteria	Bacteria	intra
SBS I vs. Control	Methylobacterium organophilum	Streptococcus lactarius	0.3061	0.001	0.892940731	NA	NA	NA	Bacteria	Bacteria	intra
SBS I vs. Control	Methylobacterium phyllosphaerae	Nocardioides piscis	-0.3974	0.001	0.892940731	NA	NA	NA	Bacteria	Bacteria	intra
SBS I vs. Control	Methylobacterium phyllosphaerae	Pseudomonas sp. LS44	-0.2617	0.004	0.892940731	NA	NA	NA	Bacteria	Bacteria	intra
SBS I vs. Control	Methylobacterium phyllosphaerae	Rouxiella chamberiensis	-0.266	0.003	0.892940731	NA	NA	NA	Bacteria	Bacteria	intra
SBS I vs. Control	Methylobacterium sp. C1	Ornithinimicrobium pratense	-0.2919	0.002	0.892940731	NA	NA	NA	Bacteria	Bacteria	intra
SBS I vs. Control	Methylocella silvestris	Paraburkholderia tropica	0.2912	0.002	0.892940731	NA	NA	NA	Bacteria	Bacteria	intra
SBS I vs. Control	Methylococcus capsulatus	Pseudarthrobacter sp. NIBRBAC000502772	0.2932	0.003	0.892940731	NA	NA	NA	Bacteria	Bacteria	intra
SBS I vs. Control	Methylococcus geothermalis	Propionibacterium freudenreichii	0.3053	0.002	0.892940731	NA	NA	NA	Bacteria	Bacteria	intra
SBS I vs. Control	Methylococcus geothermalis	Pseudoalteromonas arctica	-0.2583	0.001	0.892940731	NA	NA	NA	Bacteria	Bacteria	intra
SBS I vs. Control	Methylococcus geothermalis	Rhodococcus sp. DMU1	0.3539	0.001	0.892940731	NA	NA	NA	Bacteria	Bacteria	intra
SBS I vs. Control	Methylococcus geothermalis	Streptobacillus moniliformis	0.2683	0.001	0.892940731	NA	NA	NA	Bacteria	Bacteria	intra

SBS I vs. Control	Methylocystis bryophila	Mycobacterium sp. JS623	0.2576	0.002	0.892940731	NA	NA	NA	Bacteria	Bacteria	intra
SBS I vs. Control	Methylocystis bryophila	Thermococcus sp. GR4	-0.346	0.003	0.892940731	NA	NA	NA	Bacteria	Bacteria	intra
SBS I vs. Control	Methylocystis rosea	Mycobacterium sp. ELW1	-0.252	0.004	0.892940731	NA	NA	NA	Bacteria	Bacteria	intra
SBS I vs. Control	Metschnikowia bicuspidata	Vibrio anguillarum	0.2752	0.002	0.892940731	NA	NA	NA	Bacteria	Bacteria	intra
SBS I vs. Control	Microbacterium binotii	Microbacterium endophyticum	0.352	0.004	0.892940731	NA	NA	NA	Bacteria	Bacteria	intra
SBS I vs. Control	Microbacterium binotii	Paenibacillus andongensis	0.3684	0.001	0.892940731	NA	NA	NA	Bacteria	Bacteria	intra
SBS I vs. Control	Microbacterium binotii	Romboutsia hominis	-0.3543	0.001	0.892940731	NA	NA	NA	Bacteria	Bacteria	intra
SBS I vs. Control	Microbacterium binotii	Schaalia cardiffensis	0.305	0.001	0.892940731	NA	NA	NA	Bacteria	Bacteria	intra
SBS I vs. Control	Microbacterium binotii	uncultured Acetobacteroides sp	-0.2628	0.003	0.892940731	NA	NA	NA	Bacteria	Bacteria	intra
SBS I vs. Control	Microbacterium chengjingii	Mycobacterium simiae	0.2941	0.004	0.892940731	NA	NA	NA	Bacteria	Bacteria	intra
SBS I vs. Control	Microbacterium chengjingii	Rhodoluna limnophila	-0.2681	0.004	0.892940731	NA	NA	NA	Bacteria	Bacteria	intra
SBS I vs. Control	Microbacterium elymi	Streptomyces sp. HUAS YS2	-0.2945	0.004	0.892940731	NA	NA	NA	Bacteria	Bacteria	intra
SBS I vs. Control	Microbacterium elymi	Thermomonospora curvata	0.2705	0.003	0.892940731	NA	NA	NA	Bacteria	Bacteria	intra
SBS I vs. Control	Microbacterium endophyticum	Neisseria elongata	-0.2674	0.002	0.892940731	NA	NA	NA	Bacteria	Bacteria	intra
SBS I vs. Control	Microbacterium endophyticum	Pandoraea thiooxydans	0.2584	0.003	0.892940731	NA	NA	NA	Bacteria	Bacteria	intra
SBS I vs. Control	Microbacterium endophyticum	Pseudonocardia abyssalis	-0.2621	0.002	0.892940731	NA	NA	NA	Bacteria	Bacteria	intra
SBS I vs. Control	Microbacterium fandaimingii	Rouxiella chamberiensis	-0.3111	0.002	0.892940731	NA	NA	NA	Bacteria	Bacteria	intra
SBS I vs. Control	Microbacterium fandaimingii	Sulfuricurvum kujiense	0.2825	0.004	0.892940731	NA	NA	NA	Bacteria	Bacteria	intra
SBS I vs. Control	Microbacterium hominis	Stutzerimonas stutzeri	-0.3318	0.003	0.892940731	NA	NA	NA	Bacteria	Bacteria	intra
SBS I vs. Control	Microbacterium hominis	Thermomonospora amylolytica	-0.3469	0.002	0.892940731	NA	NA	NA	Bacteria	Bacteria	intra
SBS I vs.	Microbacterium	Neobacillus sp. Marseille-Q6967	-0.3503	0.004	0.892940731	NA	NA	NA	Bacteria	Bacteria	intra

Control	hydrocarbonoxydans										
SBS I vs. Control	Microbacterium hydrocarbonoxydans	Neorhizobium petrolearium	0.2501	0.004	0.892940731	NA	NA	NA	Bacteria	Bacteria	intra
SBS I vs. Control	Microbacterium hydrocarbonoxydans	Sphingorhabdus sp. SMR4y	0.3541	0.001	0.892940731	NA	NA	NA	Bacteria	Bacteria	intra
SBS I vs. Control	Microbacterium invictum	Polymorphospora rubra	-0.3397	0.002	0.892940731	NA	NA	NA	Bacteria	Bacteria	intra
SBS I vs. Control	Microbacterium invictum	Streptosporangium sp. 'caverna'	-0.4118	0.001	0.892940731	NA	NA	NA	Bacteria	Bacteria	intra
SBS I vs. Control	Microbacterium lushaniae	Pseudomonas promysalinigenes	-0.3557	0.001	0.892940731	NA	NA	NA	Bacteria	Bacteria	intra
SBS I vs. Control	Microbacterium sp. ABRD28	Streptococcus gwangjuense	-0.2521	0.001	0.892940731	NA	NA	NA	Bacteria	Bacteria	intra
SBS I vs. Control	Microbacterium sp. CBA3102	Mycobacterium colombiense	-0.3497	0.004	0.892940731	NA	NA	NA	Bacteria	Bacteria	intra
SBS I vs. Control	Microbacterium sp. CBA3102	Ornithinimicrobium flavum	-0.3223	0.001	0.892940731	NA	NA	NA	Bacteria	Bacteria	intra
SBS I vs. Control	Microbacterium sp. CBA3102	Rothia aeria	0.3211	0.001	0.892940731	NA	NA	NA	Bacteria	Bacteria	intra
SBS I vs. Control	Microbacterium sp. H1-D42	Roseivirga spongicola	0.291	0.001	0.892940731	NA	NA	NA	Bacteria	Bacteria	intra
SBS I vs. Control	Microbacterium sp. H1-D42	Streptococcus sp. oral taxon 431	-0.3866	0.004	0.892940731	NA	NA	NA	Bacteria	Bacteria	intra
SBS I vs. Control	Microbacterium sp. M28	Streptomyces sp. NBC_00287	0.2621	0.003	0.892940731	NA	NA	NA	Bacteria	Bacteria	intra
SBS I vs. Control	Microbacterium sp. NIBRBAC000506063	Shewanella polaris	0.2604	0.002	0.892940731	NA	NA	NA	Bacteria	Bacteria	intra
SBS I vs. Control	Microbacterium sp. Y-01	Pseudobutyrivibrio xylanivorans	-0.3332	0.002	0.892940731	NA	NA	NA	Bacteria	Bacteria	intra
SBS I vs. Control	Microbacterium sp. Y-01	Staphylococcus aureus	-0.3443	0.004	0.892940731	NA	NA	NA	Bacteria	Bacteria	intra
SBS I vs. Control	Microbacterium sp. YJN-G	Ruminiclostridium cellulolyticum	-0.3185	0.003	0.892940731	NA	NA	NA	Bacteria	Bacteria	intra
SBS I vs. Control	Microbacterium sp. nov. GSS16	Schaalia turicensis	0.2795	0.003	0.892940731	NA	NA	NA	Bacteria	Bacteria	intra
SBS I vs. Control	Microbacterium sp. zg-B185	Rothia mucilaginosa	-0.2803	0.004	0.892940731	NA	NA	NA	Bacteria	Bacteria	intra
SBS I vs. Control	Microbacterium sp. zg-Y818	Rhizophagus irregularis	-0.3177	0.002	0.892940731	NA	NA	NA	Bacteria	Bacteria	intra
SBS I vs. Control	Microbacterium suwonense	Pseudoalteromonas arctica	-0.29	0.003	0.892940731	NA	NA	NA	Bacteria	Bacteria	intra

SBS I vs. Control	Microcella humidisoli	Streptococcus sp. DTU_2020_1001019_1_SI_AUS _MUR_006	0.3616	0.001	0.892940731	NA	NA	NA	Bacteria	Bacteria	intra
SBS I vs. Control	Microcella sp.	Neoehrlichia mikurensis	-0.2806	0.003	0.892940731	NA	NA	NA	Bacteria	Bacteria	intra
SBS I vs. Control	Microcella sp.	Peterkaempfera bronchialis	-0.3206	0.001	0.892940731	NA	NA	NA	Bacteria	Bacteria	intra
SBS I vs. Control	Micrococcus luteus	Neisseria flavescens	-0.327	0.003	0.892940731	NA	NA	NA	Bacteria	Bacteria	intra
SBS I vs. Control	Micrococcus luteus	Polaribacter reichenbachii	-0.2541	0.004	0.892940731	NA	NA	NA	Bacteria	Bacteria	intra
SBS I vs. Control	Micrococcus yunnanensis	Paenibacillus sp. FSL W8-0194	-0.2717	0.003	0.892940731	NA	NA	NA	Bacteria	Bacteria	intra
SBS I vs. Control	Micrococcus yunnanensis	Silicimonas algicola	-0.2935	0.003	0.892940731	NA	NA	NA	Bacteria	Bacteria	intra
SBS I vs. Control	Microlunatus elymi	Micromonospora maris	-0.2882	0.001	0.892940731	NA	NA	NA	Bacteria	Bacteria	intra
SBS I vs. Control	Microlunatus sagamiharensis	Sporolactobacillus terrae	-0.2816	0.004	0.892940731	NA	NA	NA	Bacteria	Bacteria	intra
SBS I vs. Control	Microlunatus sagamiharensis	Streptosporangium sp. 'caverna'	0.3013	0.004	0.892940731	NA	NA	NA	Bacteria	Bacteria	intra
SBS I vs. Control	Microlunatus soli	uncultured Sphaerochaeta sp	0.3105	0.004	0.892940731	NA	NA	NA	Bacteria	Bacteria	intra
SBS I vs. Control	Micromonospora maris	Paroceanicella profunda	-0.2602	0.001	0.892940731	NA	NA	NA	Bacteria	Bacteria	intra
SBS I vs. Control	Micromonospora purpureochromogenes	Rhizobium sp. BG4	-0.2713	0.004	0.892940731	NA	NA	NA	Bacteria	Bacteria	intra
SBS I vs. Control	Micromonospora sp. HUAS LYJ1	Mycolicibacterium tokaiense	-0.3277	0.004	0.892940731	NA	NA	NA	Bacteria	Bacteria	intra
SBS I vs. Control	Micromonospora sp. WMMD882	Rathayibacter rathayi	-0.28	0.002	0.892940731	NA	NA	NA	Bacteria	Bacteria	intra
SBS I vs. Control	Micromonospora sp. WMMD882	Streptococcus sp. 1643	0.2504	0.001	0.892940731	NA	NA	NA	Bacteria	Bacteria	intra
SBS I vs. Control	Micromonospora sp. WMMD882	Thermococcus sp. 21S7	0.3354	0.004	0.892940731	NA	NA	NA	Bacteria	Bacteria	intra
SBS I vs. Control	Microterricola viridarii	uncultured Hyphomonas sp	0.2605	0.002	0.892940731	NA	NA	NA	Bacteria	Bacteria	intra
SBS I vs. Control	Microvirga sp. VF16	Parastagonospora nodorum	0.3387	0.003	0.892940731	NA	NA	NA	Bacteria	Bacteria	intra
SBS I vs. Control	Microvirga sp. VF16	Salipaludibacillus sp. LMS25	-0.4523	0.002	0.892940731	NA	NA	NA	Bacteria	Bacteria	intra

SBS I vs. Control	Miniimonas sp. S16	Protaetiibacter intestinalis	-0.2558	0.002	0.892940731	NA	NA	NA	Bacteria	Bacteria	intra
SBS I vs. Control	Mixta hanseatica	Sphingomonas ginsengisoli An et al. 2013	0.5285	0.004	0.892940731	NA	NA	NA	Bacteria	Bacteria	intra
SBS I vs. Control	Mixta hanseatica	Streptomyces bathyalis	0.2579	0.001	0.892940731	NA	NA	NA	Bacteria	Bacteria	intra
SBS I vs. Control	Mogibacterium neglectum	Sulfitobacter alexandrii	-0.2934	0.004	0.892940731	NA	NA	NA	Bacteria	Bacteria	intra
SBS I vs. Control	Mongoliitalea daihaiensis	Sphingorhabdus sp. SMR4y	0.2981	0.003	0.892940731	NA	NA	NA	Bacteria	Bacteria	intra
SBS I vs. Control	Mongoliitalea daihaiensis	Streptomyces sp. NBC_00243	-0.3078	0.002	0.892940731	NA	NA	NA	Bacteria	Bacteria	intra
SBS I vs. Control	Moraxella sp. FZFQ2102	Pasteurella skyensis	0.4705	0.001	0.892940731	NA	NA	NA	Bacteria	Bacteria	intra
SBS I vs. Control	Moritella sp. 24	Pelagerythrobacter marinus	-0.2756	0.004	0.892940731	NA	NA	NA	Bacteria	Bacteria	intra
SBS I vs. Control	Mucilaginibacter rubeus	Ralstonia insidiosa	0.3472	0.003	0.892940731	NA	NA	NA	Bacteria	Bacteria	intra
SBS I vs. Control	Mucilaginibacter rubeus	Streptomyces antibioticus	-0.2709	0.003	0.892940731	NA	NA	NA	Bacteria	Bacteria	intra
SBS I vs. Control	Mycetocola sp. JXN-3	Phaeoacremonium minimum	0.2835	0.004	0.892940731	NA	NA	NA	Bacteria	Bacteria	intra
SBS I vs. Control	Mycetocola zhujimingii	Streptococcus cristatus	0.3754	0.003	0.892940731	NA	NA	NA	Bacteria	Bacteria	intra
SBS I vs. Control	Mycobacterium colombiense	Qipengyuania spongiae	0.4087	0.001	0.892940731	NA	NA	NA	Bacteria	Bacteria	intra
SBS I vs. Control	Mycobacterium decipiens	Rhodococcus sovatensis	0.2612	0.003	0.892940731	NA	NA	NA	Bacteria	Bacteria	intra
SBS I vs. Control	Mycobacterium gordonae	Paenibacillus sp. RC67	0.2501	0.002	0.892940731	NA	NA	NA	Bacteria	Bacteria	intra
SBS I vs. Control	Mycobacterium gordonae	Ruminiclostridium cellulolyticum	-0.3049	0.003	0.892940731	NA	NA	NA	Bacteria	Bacteria	intra
SBS I vs. Control	Mycobacterium simiae	Parastagonospora nodorum	-0.3517	0.001	0.892940731	NA	NA	NA	Bacteria	Bacteria	intra
SBS I vs. Control	Mycobacterium sp. ELW1	Pseudomonas cavernae	0.2636	0.004	0.892940731	NA	NA	NA	Bacteria	Bacteria	intra
SBS I vs. Control	Mycobacterium sp. ITM-2016-00318	Streptomyces sp. NBC_00287	-0.3339	0.003	0.892940731	NA	NA	NA	Bacteria	Bacteria	intra
SBS I vs. Control	Mycobacterium stomatepiae	Vibrio rhizosphaerae	0.2568	0.002	0.892940731	NA	NA	NA	Bacteria	Bacteria	intra
SBS I vs.	Mycobacterium tuberculosis	Peptacetobacter hiranonis	0.2901	0.003	0.892940731	NA	NA	NA	Bacteria	Bacteria	intra

Control											
SBS I vs. Control	Mycobacterium tuberculosis	Tatumella ptyseos	0.3205	0.004	0.892940731	NA	NA	NA	Bacteria	Bacteria	intra
SBS I vs. Control	Mycobacterium vicinigordonae	Pseudomonas guguanensis	-0.2567	0.002	0.892940731	NA	NA	NA	Bacteria	Bacteria	intra
SBS I vs. Control	Mycolicibacterium aubagnense	Pseudoxanthomonas sp. X-1	-0.257	0.001	0.892940731	NA	NA	NA	Bacteria	Bacteria	intra
SBS I vs. Control	Mycolicibacterium aurum	Mycolicibacterium fluoranthenivorans	0.2585	0.004	0.892940731	NA	NA	NA	Bacteria	Bacteria	intra
SBS I vs. Control	Mycolicibacterium celeriflavum	Pedobacter riviphilus	-0.3397	0.003	0.892940731	NA	NA	NA	Bacteria	Bacteria	intra
SBS I vs. Control	Mycolicibacterium celeriflavum	Planctomyces sp. SH-PL14	0.2892	0.004	0.892940731	NA	NA	NA	Bacteria	Bacteria	intra
SBS I vs. Control	Mycolicibacterium chubuense	Protaetiibacter larvae	0.3596	0.001	0.892940731	NA	NA	NA	Bacteria	Bacteria	intra
SBS I vs. Control	Mycolicibacterium fluoranthenivorans	Pectobacterium aroidearum	-0.2612	0.002	0.892940731	NA	NA	NA	Bacteria	Bacteria	intra
SBS I vs. Control	Mycolicibacterium fortuitum	Mycolicibacterium rhodesiae	0.3052	0.002	0.892940731	NA	NA	NA	Bacteria	Bacteria	intra
SBS I vs. Control	Mycolicibacterium fortuitum	Rubrivivax gelatinosus	-0.2638	0.003	0.892940731	NA	NA	NA	Bacteria	Bacteria	intra
SBS I vs. Control	Mycolicibacterium fortuitum	Veillonella nakazawae	0.3448	0.003	0.892940731	NA	NA	NA	Bacteria	Bacteria	intra
SBS I vs. Control	Mycolicibacterium gadium	Paludicola sp. MB14-C6	0.2792	0.001	0.892940731	NA	NA	NA	Bacteria	Bacteria	intra
SBS I vs. Control	Mycolicibacterium goodii	Streptomyces sp. NBC_00316	-0.2888	0.003	0.892940731	NA	NA	NA	Bacteria	Bacteria	intra
SBS I vs. Control	Mycolicibacterium rutilum	Phaeoacremonium minimum	0.2512	0.002	0.892940731	NA	NA	NA	Bacteria	Bacteria	intra
SBS I vs. Control	Mycolicibacterium sp. MU0053	Streptomyces jietaisiensis	0.2862	0.002	0.892940731	NA	NA	NA	Bacteria	Bacteria	intra
SBS I vs. Control	Mycolicibacterium sp. TUM20985	Spirillospora sp. NBC_00431	-0.2717	0.003	0.892940731	NA	NA	NA	Bacteria	Bacteria	intra
SBS I vs. Control	Mycotypha africana	Sphingomonas sp. HDW15A	0.2638	0.004	0.892940731	NA	NA	NA	Bacteria	Bacteria	intra
SBS I vs. Control	Mytilinidion resinicola	Olivibacter sp. LS-1	0.2513	0.002	0.892940731	NA	NA	NA	Bacteria	Bacteria	intra
SBS I vs. Control	Mytilinidion resinicola	Streptomyces canus	-0.2795	0.002	0.892940731	NA	NA	NA	Bacteria	Bacteria	intra
SBS I vs. Control	Nakaseomyces glabratus	Staphylococcus aureus	-0.2691	0.001	0.892940731	NA	NA	NA	Bacteria	Bacteria	intra

SBS I vs. Control	Natrialba sp. PRR66	Silicimonas algicola	-0.3027	0.001	0.892940731	NA	NA	NA	Bacteria	Bacteria	intra
SBS I vs. Control	Natrialba swarupiae	Paracoccus zhejiangensis	-0.3222	0.003	0.892940731	NA	NA	NA	Bacteria	Bacteria	intra
SBS I vs. Control	Natrialba swarupiae	Phenylobacterium parvum	-0.3599	0.004	0.892940731	NA	NA	NA	Bacteria	Bacteria	intra
SBS I vs. Control	Natrinema salaciae	Rathayibacter rathayi	-0.3038	0.002	0.892940731	NA	NA	NA	Bacteria	Bacteria	intra
SBS I vs. Control	Natrinema salaciae	Selenomonas sp. oral taxon 920	-0.3183	0.003	0.892940731	NA	NA	NA	Bacteria	Bacteria	intra
SBS I vs. Control	Natronomonas halophila	Streptococcus phage SpSL1	0.2968	0.003	0.892940731	NA	NA	NA	Bacteria	Bacteria	intra
SBS I vs. Control	Neisseria sp. Marseille-Q6792	Nocardia farcinica	0.399	0.004	0.892940731	NA	NA	NA	Bacteria	Bacteria	intra
SBS I vs. Control	Neisseria sp. oral taxon 014	Nocardioides piscis	-0.2927	0.001	0.892940731	NA	NA	NA	Bacteria	Bacteria	intra
SBS I vs. Control	Neisseria sp. oral taxon 014	Ruminiclostridium cellulolyticum	0.2861	0.001	0.892940731	NA	NA	NA	Bacteria	Bacteria	intra
SBS I vs. Control	Neisseria sp. oral taxon 014	Thermococcus sp. Bubb.Bath	0.311	0.002	0.892940731	NA	NA	NA	Bacteria	Bacteria	intra
SBS I vs. Control	Neisseria subflava	Pannonibacter phragmitetus	0.3824	0.004	0.892940731	NA	NA	NA	Bacteria	Bacteria	intra
SBS I vs. Control	Neisseria yangbaofengii	Paenibacillus algicola	-0.2884	0.002	0.892940731	NA	NA	NA	Bacteria	Bacteria	intra
SBS I vs. Control	Neisseria yangbaofengii	[Pseudomonas] carboxydohydrogena	0.2623	0.004	0.892940731	NA	NA	NA	Bacteria	Bacteria	intra
SBS I vs. Control	Neobacillus sp. DY30	Streptomyces sp. NBC_00273	-0.2786	0.003	0.892940731	NA	NA	NA	Bacteria	Bacteria	intra
SBS I vs. Control	Neobacillus sp. DY30	Streptosporangium sp. 'caverna'	0.2998	0.004	0.892940731	NA	NA	NA	Bacteria	Bacteria	intra
SBS I vs. Control	Neobacillus sp. DY30	Sulfuritortus calidifontis	-0.3399	0.002	0.892940731	NA	NA	NA	Bacteria	Bacteria	intra
SBS I vs. Control	Neobacillus sp. DY30	Zhouia spongiae	-0.2805	0.004	0.892940731	NA	NA	NA	Bacteria	Bacteria	intra
SBS I vs. Control	Neobacillus sp. OS1-2	Streptococcus sp. 29896	-0.2726	0.004	0.892940731	NA	NA	NA	Bacteria	Bacteria	intra
SBS I vs. Control	Neobacillus sp. OS1-2	Vagococcus sp. B2T-5	0.3737	0.002	0.892940731	NA	NA	NA	Bacteria	Bacteria	intra
SBS I vs. Control	Neobacillus sp. YX16	Planococcus sp. N016	0.2886	0.004	0.892940731	NA	NA	NA	Bacteria	Bacteria	intra
SBS I vs.	Neobacillus sp. YX16	Priestia koreensis	0.308	0.003	0.892940731	NA	NA	NA	Bacteria	Bacteria	intra

Control											
SBS I vs. Control	Neobacillus sp. YX16	Raoultella planticola	0.2747	0.001	0.892940731	NA	NA	NA	Bacteria	Bacteria	intra
SBS I vs. Control	Neoehrlichia mikurensis	Trinickia caryophylli	0.3078	0.001	0.892940731	NA	NA	NA	Bacteria	Bacteria	intra
SBS I vs. Control	Neorhizobium petrolearium	Pseudarthrobacter defluvii	-0.2557	0.003	0.892940731	NA	NA	NA	Bacteria	Bacteria	intra
SBS I vs. Control	Neorhizobium petrolearium	Sphingobacterium sp. ML3W	0.277	0.003	0.892940731	NA	NA	NA	Bacteria	Bacteria	intra
SBS I vs. Control	Nesterenkonia pannonica	Olivibacter sp. LS-1	-0.2932	0.003	0.892940731	NA	NA	NA	Bacteria	Bacteria	intra
SBS I vs. Control	Nesterenkonia pannonica	Verminephrobacter eiseniae	0.2757	0.003	0.892940731	NA	NA	NA	Bacteria	Bacteria	intra
SBS I vs. Control	Niallia circulans	Nocardia yunnanensis	0.3047	0.004	0.892940731	NA	NA	NA	Bacteria	Bacteria	intra
SBS I vs. Control	Niallia circulans	Veillonella nakazawae	-0.2997	0.004	0.892940731	NA	NA	NA	Bacteria	Bacteria	intra
SBS I vs. Control	Niallia sp. Man26	Rossellomorea sp. y25	0.2628	0.003	0.892940731	NA	NA	NA	Bacteria	Bacteria	intra
SBS I vs. Control	Niallia sp. Man26	Streptomyces bathyalis	-0.2916	0.002	0.892940731	NA	NA	NA	Bacteria	Bacteria	intra
SBS I vs. Control	Nitrogeniibacter mangrovi	Parastagonospora nodorum	0.4181	0.003	0.892940731	NA	NA	NA	Bacteria	Bacteria	intra
SBS I vs. Control	Nitrospirillum amazonense	Sporisorium graminicola	0.2673	0.002	0.892940731	NA	NA	NA	Bacteria	Bacteria	intra
SBS I vs. Control	Niveibacterium microcysteis	Penicillium soppii	-0.344	0.002	0.892940731	NA	NA	NA	Bacteria	Bacteria	intra
SBS I vs. Control	Niveibacterium microcysteis	Streptomyces hygroscopicus	-0.2566	0.004	0.892940731	NA	NA	NA	Bacteria	Bacteria	intra
SBS I vs. Control	Niveibacterium microcysteis	Variovorax sp. PBS-H4	0.3463	0.003	0.892940731	NA	NA	NA	Bacteria	Bacteria	intra
SBS I vs. Control	Nocardia sp. NBC_01730	Schaalia sp. ZJ405	-0.2935	0.002	0.892940731	NA	NA	NA	Bacteria	Bacteria	intra
SBS I vs. Control	Nocardia yunnanensis	Phytobacter sp. SCO41	-0.2732	0.001	0.892940731	NA	NA	NA	Bacteria	Bacteria	intra
SBS I vs. Control	Nocardia yunnanensis	Pseudomonas fluorescens	0.3274	0.001	0.892940731	NA	NA	NA	Bacteria	Bacteria	intra
SBS I vs. Control	Nocardioides anomalus	Prochlorococcus marinus	0.2984	0.004	0.892940731	NA	NA	NA	Bacteria	Bacteria	intra
SBS I vs. Control	Nocardioides baekrokdamisoli	Pedobacter ginsengisoli	0.2687	0.001	0.892940731	NA	NA	NA	Bacteria	Bacteria	intra

SBS I vs. Control	Nocardioides cynanchi	Phytobacter sp. SCO41	0.2649	0.003	0.892940731	NA	NA	NA	Bacteria	Bacteria	intra
SBS I vs. Control	Nocardioides cynanchi	Trichlorobacter lovleyi	-0.3134	0.004	0.892940731	NA	NA	NA	Bacteria	Bacteria	intra
SBS I vs. Control	Nocardioides piscis	Pediococcus inopinatus	-0.2712	0.001	0.892940731	NA	NA	NA	Bacteria	Bacteria	intra
SBS I vs. Control	Nocardioides piscis	Pelagerythrobacter marinus	0.2761	0.002	0.892940731	NA	NA	NA	Bacteria	Bacteria	intra
SBS I vs. Control	Nocardioides sp. InS609-2	Paenarthrobacter ureafaciens	-0.2547	0.004	0.892940731	NA	NA	NA	Bacteria	Bacteria	intra
SBS I vs. Control	Nodularia sphaerocarpa	Veillonella sp. S12025-13	-0.3469	0.004	0.892940731	NA	NA	NA	Bacteria	Bacteria	intra
SBS I vs. Control	Nonomuraea glycinis	Streptomyces sp. SCA4-21	-0.3864	0.002	0.892940731	NA	NA	NA	Bacteria	Bacteria	intra
SBS I vs. Control	Novosphingopyxis iocasae	Paraburkholderia megapolitana	0.3133	0.001	0.892940731	NA	NA	NA	Bacteria	Bacteria	intra
SBS I vs. Control	Oceanisphaera sp. IT1-181	Zunongwangia sp. HGR-M22	-0.2564	0.003	0.892940731	NA	NA	NA	Bacteria	Bacteria	intra
SBS I vs. Control	Olsenella timonensis	Pediococcus inopinatus	-0.2739	0.004	0.892940731	NA	NA	NA	Bacteria	Bacteria	intra
SBS I vs. Control	Opitutus terrae	Rouxiella chamberiensis	0.2746	0.004	0.892940731	NA	NA	NA	Bacteria	Bacteria	intra
SBS I vs. Control	Ornithinimicrobium cryptoxanthini	Sorangium cellulosum	-0.3527	0.002	0.892940731	NA	NA	NA	Bacteria	Bacteria	intra
SBS I vs. Control	Ornithinimicrobium flavum	Penicillium cosmopolitanum	0.2769	0.003	0.892940731	NA	NA	NA	Bacteria	Bacteria	intra
SBS I vs. Control	Ornithobacterium rhinotracheale	Phialemonium atrogriseum	0.2695	0.002	0.892940731	NA	NA	NA	Bacteria	Bacteria	intra
SBS I vs. Control	Ornithobacterium rhinotracheale	Polymorphospora rubra	-0.2531	0.004	0.892940731	NA	NA	NA	Bacteria	Bacteria	intra
SBS I vs. Control	Oscillibacter sp. PEA192	Thermomonospora amylolytica	0.3296	0.002	0.892940731	NA	NA	NA	Bacteria	Bacteria	intra
SBS I vs. Control	Paenarthrobacter ureafaciens	Radiomyces spectabilis	0.2965	0.002	0.892940731	NA	NA	NA	Bacteria	Bacteria	intra
SBS I vs. Control	Paenarthrobacter ureafaciens	Rothia mucilaginosa	-0.259	0.003	0.892940731	NA	NA	NA	Bacteria	Bacteria	intra
SBS I vs. Control	Paenibacillus andongensis	Spirosoma endbachense	0.2699	0.003	0.892940731	NA	NA	NA	Bacteria	Bacteria	intra
SBS I vs. Control	Paenibacillus cellulositrophicus	Streptosporangium sp. 'caverna'	0.3183	0.003	0.892940731	NA	NA	NA	Bacteria	Bacteria	intra
SBS I vs.	Paenibacillus dendritiformis	Paenibacillus lycopersici	-0.2902	0.002	0.892940731	NA	NA	NA	Bacteria	Bacteria	intra

Control											
SBS I vs. Control	Paenibacillus elgii	Pusillibacter faecalis	0.3228	0.004	0.892940731	NA	NA	NA	Bacteria	Bacteria	intra
SBS I vs. Control	Paenibacillus lycopersici	Shewanella polaris	-0.29	0.001	0.892940731	NA	NA	NA	Bacteria	Bacteria	intra
SBS I vs. Control	Paenibacillus macerans	Pseudomonas fuscovaginae	-0.2975	0.004	0.892940731	NA	NA	NA	Bacteria	Bacteria	intra
SBS I vs. Control	Paenibacillus macerans	Sparassis crispa	0.3787	0.002	0.892940731	NA	NA	NA	Bacteria	Bacteria	intra
SBS I vs. Control	Paenibacillus mucilaginosus	uncultured Desulfosarcina sp	0.3025	0.001	0.892940731	NA	NA	NA	Bacteria	Bacteria	intra
SBS I vs. Control	Paenibacillus silvisoli	Pseudomonas sp. NIBR-H-19	-0.2954	0.002	0.892940731	NA	NA	NA	Bacteria	Bacteria	intra
SBS I vs. Control	Paenibacillus sp. FSL W8-0194	Streptomyces sp. Tue 6075	-0.3436	0.001	0.892940731	NA	NA	NA	Bacteria	Bacteria	intra
SBS I vs. Control	Paenibacillus sp. N3/727	Streptomyces pratensis	-0.2601	0.004	0.892940731	NA	NA	NA	Bacteria	Bacteria	intra
SBS I vs. Control	Paenibacillus sp. RC67	Stenotrophomonas sp. Pemsol	0.2518	0.003	0.892940731	NA	NA	NA	Bacteria	Bacteria	intra
SBS I vs. Control	Paenibacillus sp. URB8-2	Streptomyces sp. SCA4-21	0.2668	0.0038	0.892940731	NA	NA	NA	Bacteria	Bacteria	intra
SBS I vs. Control	Paenibacillus sp. URB8-2	Thermomonospora amylolytica	0.2995	0.004	0.892940731	NA	NA	NA	Bacteria	Bacteria	intra
SBS I vs. Control	Paenibacillus urinalis	Variovorax sp. PAMC26660	0.3247	0.001	0.892940731	NA	NA	NA	Bacteria	Bacteria	intra
SBS I vs. Control	Paenibacillus xylanilyticus	Streptococcus sp. oral taxon 064	-0.3405	0.001	0.892940731	NA	NA	NA	Bacteria	Bacteria	intra
SBS I vs. Control	Pandoraea thiooxydans	Pseudodesulfovibrio indicus	0.2926	0.002	0.892940731	NA	NA	NA	Bacteria	Bacteria	intra
SBS I vs. Control	Pandoraea thiooxydans	Sporolactobacillus terrae	0.2823	0.004	0.892940731	NA	NA	NA	Bacteria	Bacteria	intra
SBS I vs. Control	Pannonibacter phragmitetus	Streptococcus gordonii	-0.3116	0.004	0.892940731	NA	NA	NA	Bacteria	Bacteria	intra
SBS I vs. Control	Paraburkholderia caffeinilytica	Sporosarcina ureae	-0.2915	0.001	0.892940731	NA	NA	NA	Bacteria	Bacteria	intra
SBS I vs. Control	Paraclostridium sordellii	Pseudarthrobacter sp. MM222	0.295	0.004	0.892940731	NA	NA	NA	Bacteria	Bacteria	intra
SBS I vs. Control	Paraclostridium sordellii	uncultured Methanobrevibacter sp	0.2936	0.004	0.892940731	NA	NA	NA	Bacteria	Bacteria	intra
SBS I vs. Control	Paracoccus jeotgali	Thermococcus sp. MAR1	-0.3341	0.003	0.892940731	NA	NA	NA	Bacteria	Bacteria	intra

SBS I vs. Control	Paracoccus mutanolyticus	Paracoccus stylophorae	0.328	0.001	0.892940731	NA	NA	NA	Bacteria	Bacteria	intra
SBS I vs. Control	Paracoccus pantotrophus	Rhodococcus pseudokoreensis	-0.3161	0.003	0.892940731	NA	NA	NA	Bacteria	Bacteria	intra
SBS I vs. Control	Paracoccus stylophorae	Pseudomonas knackmussii	-0.2806	0.004	0.892940731	NA	NA	NA	Bacteria	Bacteria	intra
SBS I vs. Control	Paracoccus stylophorae	Pseudomonas sp. CCOS 191	0.2988	0.003	0.892940731	NA	NA	NA	Bacteria	Bacteria	intra
SBS I vs. Control	Paracoccus stylophorae	Sphingomonas sp. S2-65	0.2911	0.004	0.892940731	NA	NA	NA	Bacteria	Bacteria	intra
SBS I vs. Control	Paracoccus stylophorae	Streptomyces sp. NBC_00390	0.2631	0.002	0.892940731	NA	NA	NA	Bacteria	Bacteria	intra
SBS I vs. Control	Paracoccus stylophorae	Vibrio aerogenes	0.3262	0.002	0.892940731	NA	NA	NA	Bacteria	Bacteria	intra
SBS I vs. Control	Paracoccus zhejiangensis	Streptomyces sp. MA3_2.13	0.5085	0.001	0.892940731	NA	NA	NA	Bacteria	Bacteria	intra
SBS I vs. Control	Paraconexibacter antarcticus	Polaribacter tangerinus	0.3131	0.003	0.892940731	NA	NA	NA	Bacteria	Bacteria	intra
SBS I vs. Control	Paraconexibacter antarcticus	Streptococcus sp. FSL W8-0197	0.2534	0.003	0.892940731	NA	NA	NA	Bacteria	Bacteria	intra
SBS I vs. Control	Parafannyhessea umbonata	Rhizobium gei	-0.4097	0.001	0.892940731	NA	NA	NA	Bacteria	Bacteria	intra
SBS I vs. Control	Paraoerskovia sediminicola	Pseudomonas sp. GCEP-101	-0.344	0.004	0.892940731	NA	NA	NA	Bacteria	Bacteria	intra
SBS I vs. Control	Paraoerskovia sediminicola	Rhizorhabdus dicambivorans	-0.2918	0.004	0.892940731	NA	NA	NA	Bacteria	Bacteria	intra
SBS I vs. Control	Pararhizobium sp. YC-54	Parastagonospora nodorum	0.3332	0.004	0.892940731	NA	NA	NA	Bacteria	Bacteria	intra
SBS I vs. Control	Pararhizobium sp. YC-54	Pseudonocardia sp. DSM 110487	-0.3359	0.003	0.892940731	NA	NA	NA	Bacteria	Bacteria	intra
SBS I vs. Control	Pararhizobium sp. YC-54	Sphingobacterium sp. ML3W	-0.287	0.004	0.892940731	NA	NA	NA	Bacteria	Bacteria	intra
SBS I vs. Control	Pararhizobium sp. YC-54	Thyridium curvatum	-0.296	0.003	0.892940731	NA	NA	NA	Bacteria	Bacteria	intra
SBS I vs. Control	Parasphingorhabdus sp. SCSIO 66989	Shinella sp. XGS7	0.3025	0.004	0.892940731	NA	NA	NA	Bacteria	Bacteria	intra
SBS I vs. Control	Parastagonospora nodorum	Pseudoduganella lutea	0.255	0.003	0.892940731	NA	NA	NA	Bacteria	Bacteria	intra
SBS I vs. Control	Parastagonospora nodorum	Rhodanobacter sp. AS-Z3	0.3024	0.003	0.892940731	NA	NA	NA	Bacteria	Bacteria	intra
SBS I vs.	Parastagonospora nodorum	Sphingopyxis indica	0.2639	0.004	0.892940731	NA	NA	NA	Bacteria	Bacteria	intra

Control											
SBS I vs. Control	Pasteurella multocida	Pseudomonas toyotomiensis	-0.3073	0.001	0.892940731	NA	NA	NA	Bacteria	Bacteria	intra
SBS I vs. Control	Pasteurella skyensis	Polaromonas naphthalenivorans	-0.3969	0.004	0.892940731	NA	NA	NA	Bacteria	Bacteria	intra
SBS I vs. Control	Pasteurella skyensis	Thermococcus sp. Bubb.Bath	0.2991	0.002	0.892940731	NA	NA	NA	Bacteria	Bacteria	intra
SBS I vs. Control	Paucibacter sp. KCTC 42545	Streptomyces rimosus	0.3173	0.003	0.892940731	NA	NA	NA	Bacteria	Bacteria	intra
SBS I vs. Control	Paucibacter sp. S2-9	Streptococcus oralis	-0.2977	0.003	0.892940731	NA	NA	NA	Bacteria	Bacteria	intra
SBS I vs. Control	Pectobacterium polaris	Vaginimicrobium propionicum	0.3815	0.001	0.892940731	NA	NA	NA	Bacteria	Bacteria	intra
SBS I vs. Control	Pectobacterium wasabiae	Sphingopyxis sp. GC21	0.2502	0.001	0.892940731	NA	NA	NA	Bacteria	Bacteria	intra
SBS I vs. Control	Pediococcus claussenii	Streptomyces sp. NBC_00390	0.3589	0.001	0.892940731	NA	NA	NA	Bacteria	Bacteria	intra
SBS I vs. Control	Pediococcus inopinatus	Saccharopolyspora erythraea	0.2837	0.002	0.892940731	NA	NA	NA	Bacteria	Bacteria	intra
SBS I vs. Control	Pedobacter ginsengisoli	Ralstonia insidiosa	0.3728	0.003	0.892940731	NA	NA	NA	Bacteria	Bacteria	intra
SBS I vs. Control	Pedobacter ginsengisoli	Rhizobium sp. AB2/73	0.4014	0.003	0.892940731	NA	NA	NA	Bacteria	Bacteria	intra
SBS I vs. Control	Pelagerythrobacter marinus	Streptomyces tubbatahanensis	0.2579	0.001	0.892940731	NA	NA	NA	Bacteria	Bacteria	intra
SBS I vs. Control	Pelagerythrobacter marinus	Uncinocarpus reesii	-0.3269	0.004	0.892940731	NA	NA	NA	Bacteria	Bacteria	intra
SBS I vs. Control	Pelagovum pacificum	Streptococcus sp. 1643	-0.2776	0.004	0.892940731	NA	NA	NA	Bacteria	Bacteria	intra
SBS I vs. Control	Pelagovum pacificum	Streptomyces sp. NBC_01304	-0.2729	0.002	0.892940731	NA	NA	NA	Bacteria	Bacteria	intra
SBS I vs. Control	Pelobacter propionicus	Salinibacterium sp. M195	0.2613	0.002	0.892940731	NA	NA	NA	Bacteria	Bacteria	intra
SBS I vs. Control	Penicillium griseofulvum	Pseudanabaena sp. ABRG5-3	0.3828	0.003	0.892940731	NA	NA	NA	Bacteria	Bacteria	intra
SBS I vs. Control	Penicillium waksmanii	Xanthomonas phaseoli	0.4092	0.001	0.892940731	NA	NA	NA	Bacteria	Bacteria	intra
SBS I vs. Control	Peptacetobacter hiranonis	Thermococcus sp. MAR1	0.2757	0.003	0.892940731	NA	NA	NA	Bacteria	Bacteria	intra
SBS I vs. Control	Peribacillus frigoritolerans	Thermosipho melanesiensis	0.2938	0.004	0.892940731	NA	NA	NA	Bacteria	Bacteria	intra

SBS I vs. Control	Peribacillus sp. R9-11	Sinomonas atrocyanea	0.287	0.003	0.892940731	NA	NA	NA	Bacteria	Bacteria	intra
SBS I vs. Control	Peribacillus sp. R9-11	Sphingobacterium sp. DR205	0.3949	0.001	0.892940731	NA	NA	NA	Bacteria	Bacteria	intra
SBS I vs. Control	Peterkaempfera bronchialis	Streptococcus gordonii	0.3334	0.002	0.892940731	NA	NA	NA	Bacteria	Bacteria	intra
SBS I vs. Control	Peterkaempfera bronchialis	Thermococcus sp. MAR1	0.2563	0.003	0.892940731	NA	NA	NA	Bacteria	Bacteria	intra
SBS I vs. Control	Peteryoungia desertarenae	Saccharomycodes ludwigii	0.2644	0.004	0.892940731	NA	NA	NA	Bacteria	Bacteria	intra
SBS I vs. Control	Peteryoungia desertarenae	Streptomyces sp. NBC_01304	-0.2706	0.001	0.892940731	NA	NA	NA	Bacteria	Bacteria	intra
SBS I vs. Control	Phaeoacremonium minimum	Rhodococcus sovatensis	-0.2586	0.003	0.892940731	NA	NA	NA	Bacteria	Bacteria	intra
SBS I vs. Control	Phaeoacremonium minimum	Streptomyces flavofungini	0.306	0.004	0.892940731	NA	NA	NA	Bacteria	Bacteria	intra
SBS I vs. Control	Phaeoacremonium minimum	Streptomyces sp. NBC_01216	-0.2791	0.002	0.892940731	NA	NA	NA	Bacteria	Bacteria	intra
SBS I vs. Control	Phenylobacterium parvum	Streptomyces sp. MA3_2.13	0.3077	0.002	0.892940731	NA	NA	NA	Bacteria	Bacteria	intra
SBS I vs. Control	Photobacterium leiognathi	Rothia sp. SD9660Na	0.3341	0.001	0.892940731	NA	NA	NA	Bacteria	Bacteria	intra
SBS I vs. Control	Photobacterium leiognathi	Streptomyces rimosus	0.3235	0.002	0.892940731	NA	NA	NA	Bacteria	Bacteria	intra
SBS I vs. Control	Photobacterium sp. TY1-4	Streptococcus sp. 1643	-0.3365	0.004	0.892940731	NA	NA	NA	Bacteria	Bacteria	intra
SBS I vs. Control	Photobacterium sp. TY1-4	Streptosporangium sp. NBC_01495	-0.3067	0.001	0.892940731	NA	NA	NA	Bacteria	Bacteria	intra
SBS I vs. Control	Piscinibacter gummiphilus	Streptococcus rubneri	-0.2878	0.002	0.892940731	NA	NA	NA	Bacteria	Bacteria	intra
SBS I vs. Control	Planctopirus limnophila	Streptomyces sp. NBC_00335	-0.3385	0.002	0.892940731	NA	NA	NA	Bacteria	Bacteria	intra
SBS I vs. Control	Planococcus versutus	Rothia mucilaginosa	0.2526	0.003	0.892940731	NA	NA	NA	Bacteria	Bacteria	intra
SBS I vs. Control	Planococcus versutus	Sporomusa ovata	-0.2878	0.001	0.892940731	NA	NA	NA	Bacteria	Bacteria	intra
SBS I vs. Control	Planococcus versutus	Streptomyces sp. MA3_2.13	0.2962	0.003	0.892940731	NA	NA	NA	Bacteria	Bacteria	intra
SBS I vs. Control	Polaribacter tangerinus	Schizosaccharomyces japonicus	0.3903	0.004	0.892940731	NA	NA	NA	Bacteria	Bacteria	intra
SBS I vs.	Polaromonas	Wickerhamomyces ciferrii	0.3443	0.004	0.892940731	NA	NA	NA	Bacteria	Bacteria	intra

Control	naphthalenivorans										
SBS I vs. Control	Prevotella multiformis	Schaalia cardiffensis	-0.3126	0.001	0.892940731	NA	NA	NA	Bacteria	Bacteria	intra
SBS I vs. Control	Prevotella multiformis	Streptomyces sp. NBC_01296	0.3086	0.003	0.892940731	NA	NA	NA	Bacteria	Bacteria	intra
SBS I vs. Control	Pseudarthrobacter defluvii	Streptococcus sp. 116-D4	0.2646	0.002	0.892940731	NA	NA	NA	Bacteria	Bacteria	intra
SBS I vs. Control	Pseudarthrobacter sp. MM222	Streptomyces sp. NBC_01216	0.287	0.002	0.892940731	NA	NA	NA	Bacteria	Bacteria	intra
SBS I vs. Control	Pseudobutyrivibrio xylanivorans	Pseudomonas sp. DR48	0.3412	0.001	0.892940731	NA	NA	NA	Bacteria	Bacteria	intra
SBS I vs. Control	Pseudoduganella flava	Sulfitobacter sp. THAF37	-0.283	0.003	0.892940731	NA	NA	NA	Bacteria	Bacteria	intra
SBS I vs. Control	Pseudoduganella flava	uncultured Lichenicoccus sp	0.2793	0.003	0.892940731	NA	NA	NA	Bacteria	Bacteria	intra
SBS I vs. Control	Pseudomonas alcaligenes	Ruania suaedae	-0.3091	0.002	0.892940731	NA	NA	NA	Bacteria	Bacteria	intra
SBS I vs. Control	Pseudomonas arsenicoxydans	Sphingobacterium multivorum	-0.3323	0.001	0.892940731	NA	NA	NA	Bacteria	Bacteria	intra
SBS I vs. Control	Pseudomonas cavernae	Streptococcus phage PH15	-0.2551	0.001	0.892940731	NA	NA	NA	Bacteria	Bacteria	intra
SBS I vs. Control	Pseudomonas cavernae	Thermaerobacter marianensis	-0.2761	0.002	0.892940731	NA	NA	NA	Bacteria	Bacteria	intra
SBS I vs. Control	Pseudomonas knackmussii	Rathayibacter toxicus	0.2851	0.001	0.892940731	NA	NA	NA	Bacteria	Bacteria	intra
SBS I vs. Control	Pseudomonas laurylsulfatiphila	Rhizorhabdus dicambivorans	-0.2743	0.004	0.892940731	NA	NA	NA	Bacteria	Bacteria	intra
SBS I vs. Control	Pseudomonas orientalis	Rothia terrae	-0.2677	0.004	0.892940731	NA	NA	NA	Bacteria	Bacteria	intra
SBS I vs. Control	Pseudomonas orientalis	Streptomyces chartreusis	0.285	0.003	0.892940731	NA	NA	NA	Bacteria	Bacteria	intra
SBS I vs. Control	Pseudomonas sp. B21-019	Streptomyces rimosus	-0.331	0.004	0.892940731	NA	NA	NA	Bacteria	Bacteria	intra
SBS I vs. Control	Pseudomonas sp. DC1.2	Serinicoccus marinus	0.2644	0.003	0.892940731	NA	NA	NA	Bacteria	Bacteria	intra
SBS I vs. Control	Pseudomonas sp. GCEP-101	Variovorax paradoxus	-0.2619	0.003	0.892940731	NA	NA	NA	Bacteria	Bacteria	intra
SBS I vs. Control	Pseudomonas sp. LS44	Thermococcus sp. GR4	-0.2715	0.003	0.892940731	NA	NA	NA	Bacteria	Bacteria	intra
SBS I vs. Control	Pseudomonas sp. MPC6	Pseudomonas sp. NIBR-H-19	-0.3126	0.003	0.892940731	NA	NA	NA	Bacteria	Bacteria	intra

SBS I vs. Control	Pseudomonas toyotomiensis	Streptococcus toyakuensis	0.2602	0.004	0.892940731	NA	NA	NA	Bacteria	Bacteria	intra
SBS I vs. Control	Pseudomonas trivialis	Streptococcus gwangjuense	0.3292	0.002	0.892940731	NA	NA	NA	Bacteria	Bacteria	intra
SBS I vs. Control	Pseudomonas umsongensis	Rouxiella chamberiensis	-0.3013	0.002	0.892940731	NA	NA	NA	Bacteria	Bacteria	intra
SBS I vs. Control	Pseudomonas umsongensis	Streptococcus phage SpSL1	0.3337	0.003	0.892940731	NA	NA	NA	Bacteria	Bacteria	intra
SBS I vs. Control	Pseudomonas umsongensis	Streptomyces durmitorensis	-0.2526	0.002	0.892940731	NA	NA	NA	Bacteria	Bacteria	intra
SBS I vs. Control	Pseudomonas umsongensis	Thioclava sp. GXIMD4216	-0.3174	0.003	0.892940731	NA	NA	NA	Bacteria	Bacteria	intra
SBS I vs. Control	Pseudonocardia dioxanivorans	Symbiopectobacterium purcellii	-0.2521	0.002	0.892940731	NA	NA	NA	Bacteria	Bacteria	intra
SBS I vs. Control	Pseudonocardia sp. DSM 110487	Streptomyces capitiformicae	-0.2784	0.001	0.892940731	NA	NA	NA	Bacteria	Bacteria	intra
SBS I vs. Control	Pseudorhodoplanes sinuspersici	Roseivirga spongicola	0.298	0.002	0.892940731	NA	NA	NA	Bacteria	Bacteria	intra
SBS I vs. Control	Pseudoxanthomonas sp. X-1	Sphingobium sp. RAC03	0.2918	0.002	0.892940731	NA	NA	NA	Bacteria	Bacteria	intra
SBS I vs. Control	Pseudoxanthomonas sp. X-1	Synechococcus elongatus	-0.2598	0.004	0.892940731	NA	NA	NA	Bacteria	Bacteria	intra
SBS I vs. Control	Purpureocillium lilacinum	Schaalia sp. ZJ405	0.3101	0.002	0.892940731	NA	NA	NA	Bacteria	Bacteria	intra
SBS I vs. Control	Purpureocillium lilacinum	uncultured Enterococcus sp	0.2573	0.002	0.892940731	NA	NA	NA	Bacteria	Bacteria	intra
SBS I vs. Control	Qipengyuania spongiae	Spirosoma oryzicola	0.3148	0.002	0.892940731	NA	NA	NA	Bacteria	Bacteria	intra
SBS I vs. Control	Radiomyces spectabilis	Streptomyces sp. NBC_01450	-0.3231	0.001	0.892940731	NA	NA	NA	Bacteria	Bacteria	intra
SBS I vs. Control	Ralstonia insidiosa	Rhizobium lusitanum	-0.2719	0.001	0.892940731	NA	NA	NA	Bacteria	Bacteria	intra
SBS I vs. Control	Rhizobium leguminosarum	Streptococcus sp. DTU_2020_1001019_1_SI_AUS_MUR_006	-0.3457	0.001	0.892940731	NA	NA	NA	Bacteria	Bacteria	intra
SBS I vs. Control	Rhizobium sp. NXC24	Tatumella ptyseos	-0.2802	0.001	0.892940731	NA	NA	NA	Bacteria	Bacteria	intra
SBS I vs. Control	Rhizorhabdus dicambivorans	Saccharomyces eubayanus	-0.3264	0.001	0.892940731	NA	NA	NA	Bacteria	Bacteria	intra
SBS I vs. Control	Rhizorhabdus dicambivorans	Streptococcus pneumoniae	-0.2528	0.001	0.892940731	NA	NA	NA	Bacteria	Bacteria	intra

SBS I vs. Control	Rhizorhabdus wittichii	Streptococcus gordonii	0.2607	0.003	0.892940731	NA	NA	NA	Bacteria	Bacteria	intra
SBS I vs. Control	Rhodanobacter sp. AS-Z3	Schaalia radingae	0.3584	0.004	0.892940731	NA	NA	NA	Bacteria	Bacteria	intra
SBS I vs. Control	Rhodococcus triatomae	[Clostridium] hylemonae	0.4088	0.002	0.892940731	NA	NA	NA	Bacteria	Bacteria	intra
SBS I vs. Control	Rhodoferax ferrireducens	Streptomyces rimosus	0.3001	0.002	0.892940731	NA	NA	NA	Bacteria	Bacteria	intra
SBS I vs. Control	Rhodoluna limnophila	Streptococcus sp. D7B5	0.2899	0.002	0.892940731	NA	NA	NA	Bacteria	Bacteria	intra
SBS I vs. Control	Riemerella anatipestifer	Starkeya sp. ORNL1	0.3551	0.004	0.892940731	NA	NA	NA	Bacteria	Bacteria	intra
SBS I vs. Control	Romboutsia hominis	Runella sp. SP2	-0.2504	0.003	0.892940731	NA	NA	NA	Bacteria	Bacteria	intra
SBS I vs. Control	Roseitalea porphyridii	Trichlorobacter lovleyi	0.3098	0.003	0.892940731	NA	NA	NA	Bacteria	Bacteria	intra
SBS I vs. Control	Roseivivax marinus	Sulfuricurvum kujiense	0.3199	0.004	0.892940731	NA	NA	NA	Bacteria	Bacteria	intra
SBS I vs. Control	Rothia aeria	Rothia amarae	0.2907	0.001	0.892940731	NA	NA	NA	Bacteria	Bacteria	intra
SBS I vs. Control	Rothia aeria	Veillonella nakazawae	-0.3923	0.003	0.892940731	NA	NA	NA	Bacteria	Bacteria	intra
SBS I vs. Control	Rothia dentocariosa	Streptomyces griseus	0.2643	0.003	0.892940731	NA	NA	NA	Bacteria	Bacteria	intra
SBS I vs. Control	Rothia dentocariosa	Streptomyces sp. Tue 6075	-0.2926	0.001	0.892940731	NA	NA	NA	Bacteria	Bacteria	intra
SBS I vs. Control	Rothia mucilaginosa	Trueperella abortisuis	-0.2536	0.003	0.892940731	NA	NA	NA	Bacteria	Bacteria	intra
SBS I vs. Control	Ruania suaedae	Sphingomonas sp. KRR8	0.2862	0.003	0.892940731	NA	NA	NA	Bacteria	Bacteria	intra
SBS I vs. Control	Rubrobacter radiotolerans	Schaalia sp. 19OD2882	-0.2769	0.003	0.892940731	NA	NA	NA	Bacteria	Bacteria	intra
SBS I vs. Control	Ruminiclostridium cellulolyticum	Salaquimonas pukyongi	0.2835	0.003	0.892940731	NA	NA	NA	Bacteria	Bacteria	intra
SBS I vs. Control	Ruminococcus champanellensis	Thyridium curvatum	0.3238	0.001	0.892940731	NA	NA	NA	Bacteria	Bacteria	intra
SBS I vs. Control	Saccharomonospora glauca	Streptococcus toyakuensis	-0.2766	0.004	0.892940731	NA	NA	NA	Bacteria	Bacteria	intra
SBS I vs. Control	Saccharomonospora glauca	Streptomyces sp. NBC_01296	0.3585	0.002	0.892940731	NA	NA	NA	Bacteria	Bacteria	intra
SBS I vs.	Saccharomycodes ludwigii	Thiomonas sp. X19	0.302	0.004	0.892940731	NA	NA	NA	Bacteria	Bacteria	intra

Control											
SBS I vs. Control	Saccharomycodes ludwigii	Xanthomonas phaseoli	-0.3353	0.001	0.892940731	NA	NA	NA	Bacteria	Bacteria	intra
SBS I vs. Control	Saccharopolyspora gloriosae	Sphingomonas sp. KRR8	-0.2739	0.001	0.892940731	NA	NA	NA	Bacteria	Bacteria	intra
SBS I vs. Control	Salaquimonas pukyongi	Vibrio rhizosphaerae	-0.3048	0.001	0.892940731	NA	NA	NA	Bacteria	Bacteria	intra
SBS I vs. Control	Schaalia cardiffensis	Streptomyces sp. NBC_00287	-0.2586	0.002	0.892940731	NA	NA	NA	Bacteria	Bacteria	intra
SBS I vs. Control	Schaalia radingae	Trueperella bernardiae	-0.3543	0.001	0.892940731	NA	NA	NA	Bacteria	Bacteria	intra
SBS I vs. Control	Schaalia radingae	[Ruminococcus] torques	-0.3141	0.004	0.892940731	NA	NA	NA	Bacteria	Bacteria	intra
SBS I vs. Control	Schaalia sp. ZJ405	Streptococcus rubneri	-0.3239	0.001	0.892940731	NA	NA	NA	Bacteria	Bacteria	intra
SBS I vs. Control	Schaalia sp. ZJ405	Tannockella kyphosi	-0.3773	0.003	0.892940731	NA	NA	NA	Bacteria	Bacteria	intra
SBS I vs. Control	Schaalia sp. ZJ405	Tatumella ptyseos	-0.2662	0.003	0.892940731	NA	NA	NA	Bacteria	Bacteria	intra
SBS I vs. Control	Selenomonas sp. oral taxon 920	Stenotrophomonas pavanii	-0.2724	0.002	0.892940731	NA	NA	NA	Bacteria	Bacteria	intra
SBS I vs. Control	Serinicoccus profundi	Trueperella bernardiae	0.266	0.002	0.892940731	NA	NA	NA	Bacteria	Bacteria	intra
SBS I vs. Control	Serratia liquefaciens	Verminephrobacter eiseniae	-0.412	0.001	0.892940731	NA	NA	NA	Bacteria	Bacteria	intra
SBS I vs. Control	Shewanella polaris	Thermobispora bispora	0.2624	0.004	0.892940731	NA	NA	NA	Bacteria	Bacteria	intra
SBS I vs. Control	Shewanella polaris	Tumebacillus avium	-0.2875	0.002	0.892940731	NA	NA	NA	Bacteria	Bacteria	intra
SBS I vs. Control	Shinella sp. XGS7	Thermomonas brevis	0.3306	0.001	0.892940731	NA	NA	NA	Bacteria	Bacteria	intra
SBS I vs. Control	Simiaoa sunii	Variovorax sp. PBL-H6	-0.3366	0.003	0.892940731	NA	NA	NA	Bacteria	Bacteria	intra
SBS I vs. Control	Simiaoa sunii	Verminephrobacter eiseniae	0.3792	0.004	0.892940731	NA	NA	NA	Bacteria	Bacteria	intra
SBS I vs. Control	Simplicispira suum	uncultured Desulfuromonas sp	0.2773	0.004	0.892940731	NA	NA	NA	Bacteria	Bacteria	intra
SBS I vs. Control	Sinomonas atrocyanea	Streptomyces rectiverticillatus	-0.2611	0.002	0.892940731	NA	NA	NA	Bacteria	Bacteria	intra
SBS I vs. Control	Skermanella pratensis	Sphingobacterium multivorum	0.3142	0.002	0.892940731	NA	NA	NA	Bacteria	Bacteria	intra

SBS I vs. Control	Skermanella pratensis	Streptococcus australis	0.2939	0.002	0.892940731	NA	NA	NA	Bacteria	Bacteria	intra
SBS I vs. Control	Skermanella pratensis	Variovorax sp. 38R	-0.3132	0.004	0.892940731	NA	NA	NA	Bacteria	Bacteria	intra
SBS I vs. Control	Sphingobacterium multivorum	uncultured Lichenicoccus sp	-0.341	0.002	0.892940731	NA	NA	NA	Bacteria	Bacteria	intra
SBS I vs. Control	Sphingobacterium sp. DR205	Zymomonas mobilis	0.2655	0.001	0.892940731	NA	NA	NA	Bacteria	Bacteria	intra
SBS I vs. Control	Sphingobium cloacae	Streptomyces rapamycinicus	0.389	0.002	0.892940731	NA	NA	NA	Bacteria	Bacteria	intra
SBS I vs. Control	Sphingobium sp. RAC03	Streptomyces netropsis	-0.2699	0.002	0.892940731	NA	NA	NA	Bacteria	Bacteria	intra
SBS I vs. Control	Sphingomonas changnyeongensis	Streptococcus rubneri	-0.2546	0.004	0.892940731	NA	NA	NA	Bacteria	Bacteria	intra
SBS I vs. Control	Sphingopyxis sp. 113P3	Streptomyces sp. 891-h	-0.3751	0.001	0.892940731	NA	NA	NA	Bacteria	Bacteria	intra
SBS I vs. Control	Sphingorhabdus sp. SMR4y	Streptococcus sp. D7B5	0.3147	0.001	0.892940731	NA	NA	NA	Bacteria	Bacteria	intra
SBS I vs. Control	Spirosoma oryzicola	Streptomyces sp. HUAS 14-6	-0.2761	0.002	0.892940731	NA	NA	NA	Bacteria	Bacteria	intra
SBS I vs. Control	Spirosoma oryzicola	Treponema socranskii	0.337	0.001	0.892940731	NA	NA	NA	Bacteria	Bacteria	intra
SBS I vs. Control	Sporomusa termitida	Tichowtungia aerotolerans	0.4171	0.001	0.892940731	0.2804	0.001	0.910156812	Bacteria	Bacteria	intra
SBS I vs. Control	Stenotrophomonas sp. Pemsol	uncultured Hyphomonas sp	0.2791	0.003	0.892940731	NA	NA	NA	Bacteria	Bacteria	intra
SBS I vs. Control	Streptobacillus moniliformis	Streptomyces actuosus	-0.2831	0.001	0.892940731	NA	NA	NA	Bacteria	Bacteria	intra
SBS I vs. Control	Streptococcus anginosus	Stutzerimonas stutzeri	-0.2563	0.002	0.892940731	NA	NA	NA	Bacteria	Bacteria	intra
SBS I vs. Control	Streptococcus koreensis	Streptomyces durmitorensis	0.261	0.003	0.892940731	NA	NA	NA	Bacteria	Bacteria	intra
SBS I vs. Control	Streptococcus mitis	Streptomyces sp. Q6	-0.2958	0.002	0.892940731	NA	NA	NA	Bacteria	Bacteria	intra
SBS I vs. Control	Streptococcus mitis	Streptomyces sp. f51	-0.2691	0.002	0.892940731	NA	NA	NA	Bacteria	Bacteria	intra
SBS I vs. Control	Streptococcus mitis	Tessaracoccus sp. MC1865	0.2997	0.004	0.892940731	NA	NA	NA	Bacteria	Bacteria	intra
SBS I vs. Control	Streptococcus phage PH15	Streptomyces sp. HUAS 2-6	0.2542	0.003	0.892940731	NA	NA	NA	Bacteria	Bacteria	intra
SBS I vs.	Streptococcus phage SpSL1	Streptomyces chartreusis	-0.2726	0.004	0.892940731	NA	NA	NA	Bacteria	Bacteria	intra

Control											
SBS I vs. Control	Streptococcus phage SpSL1	Streptomyces tubbatahanensis	0.2863	0.001	0.892940731	NA	NA	NA	Bacteria	Bacteria	intra
SBS I vs. Control	Streptococcus sanguinis	Syntrophotalea acetylenica	0.3013	0.001	0.892940731	NA	NA	NA	Bacteria	Bacteria	intra
SBS I vs. Control	Streptococcus sp. D7B5	[Clostridium] hylemonae	-0.2741	0.001	0.892940731	NA	NA	NA	Bacteria	Bacteria	intra
SBS I vs. Control	Streptococcus sp. FSL W8-0197	Tepiditoga spiralis	-0.3196	0.003	0.892940731	NA	NA	NA	Bacteria	Bacteria	intra
SBS I vs. Control	Streptococcus sp. oral taxon 061	Vanderwaltozyma polyspora	0.2577	0.004	0.892940731	NA	NA	NA	Bacteria	Bacteria	intra
SBS I vs. Control	Streptococcus sp. oral taxon 061	uncultured Hyphomonas sp	-0.2563	0.003	0.892940731	NA	NA	NA	Bacteria	Bacteria	intra
SBS I vs. Control	Streptococcus sp. zg-86	Streptosporangium sp. NBC_01495	0.3108	0.001	0.892940731	NA	NA	NA	Bacteria	Bacteria	intra
SBS I vs. Control	Streptomyces atratus	Symbiopectobacterium purcellii	0.2713	0.003	0.892940731	NA	NA	NA	Bacteria	Bacteria	intra
SBS I vs. Control	Streptomyces canus	Streptomyces sp. NBC_00243	0.3325	0.003	0.892940731	NA	NA	NA	Bacteria	Bacteria	intra
SBS I vs. Control	Streptomyces cynarae	[Pseudomonas] carboxydohydrogena	-0.283	0.003	0.892940731	NA	NA	NA	Bacteria	Bacteria	intra
SBS I vs. Control	Streptomyces pratensis	Sutcliffiella horikoshii	-0.2768	0.004	0.892940731	NA	NA	NA	Bacteria	Bacteria	intra
SBS I vs. Control	Streptomyces rapamycinicus	Zhouia spongiae	0.3019	0.002	0.892940731	NA	NA	NA	Bacteria	Bacteria	intra
SBS I vs. Control	Streptomyces rectiverticillatus	Variovorax sp. 38R	-0.3135	0.004	0.892940731	NA	NA	NA	Bacteria	Bacteria	intra
SBS I vs. Control	Streptomyces rimosus	Streptomyces sp. HUAS YS2	-0.2669	0.002	0.892940731	NA	NA	NA	Bacteria	Bacteria	intra
SBS I vs. Control	Streptomyces sp. DSM 40750	Tepidibacter hydrothermalis	-0.269	0.004	0.892940731	NA	NA	NA	Bacteria	Bacteria	intra
SBS I vs. Control	Streptomyces sp. HUAS 2-6	Tepidibacter hydrothermalis	0.323	0.003	0.892940731	NA	NA	NA	Bacteria	Bacteria	intra
SBS I vs. Control	Streptomyces sp. HUAS YS2	Variovorax boronicumulans	-0.2925	0.001	0.892940731	NA	NA	NA	Bacteria	Bacteria	intra
SBS I vs. Control	Streptomyces sp. NBC_00490	Vibrio navarrensis	0.2912	0.002	0.892940731	NA	NA	NA	Bacteria	Bacteria	intra
SBS I vs. Control	Streptomyces sp. NBC_00536	Trichoderma citrinoviride	0.3035	0.002	0.892940731	NA	NA	NA	Bacteria	Bacteria	intra
SBS I vs. Control	Streptomyces sp. NBC_01217	Stutzerimonas stutzeri	0.3184	0.001	0.892940731	NA	NA	NA	Bacteria	Bacteria	intra

SBS I vs. Control	Streptomyces sp. NBC_01255	Yersinia frederiksenii	0.3387	0.001	0.892940731	NA	NA	NA	Bacteria	Bacteria	intra
SBS I vs. Control	Streptomyces sp. NBC_01304	Vanderwaltozyma polyspora	0.3197	0.003	0.892940731	NA	NA	NA	Bacteria	Bacteria	intra
SBS I vs. Control	Streptomyces sp. NBC_01450	Tsukamurella pulmonis	-0.3013	0.003	0.892940731	NA	NA	NA	Bacteria	Bacteria	intra
SBS I vs. Control	Sulfitobacter mediterraneus	uncultured Desulfobulbus sp	-0.3155	0.001	0.892940731	NA	NA	NA	Bacteria	Bacteria	intra
SBS I vs. Control	Tannockella kyphosi	Variovorax sp. RKNM96	0.253	0.001	0.892940731	NA	NA	NA	Bacteria	Bacteria	intra
SBS I vs. Control	Tepidibacter hydrothermalis	Thermococcus sp. GR4	0.2722	0.002	0.892940731	NA	NA	NA	Bacteria	Bacteria	intra
SBS I vs. Control	Thermococcus sp. MAR1	Tsukamurella pulmonis	0.2704	0.004	0.892940731	NA	NA	NA	Bacteria	Bacteria	intra
SBS I vs. Control	Thioclava sp. GXIMD4216	Trichoderma atroviride	-0.3533	0.003	0.892940731	NA	NA	NA	Bacteria	Bacteria	intra
SBS I vs. Control	Thioflavicoccus mobilis	Variovorax sp. PBS-H4	0.3536	0.003	0.892940731	NA	NA	NA	Bacteria	Bacteria	intra
SBS I vs. Control	Thioflavicoccus mobilis	uncultured Lichenicoccus sp	-0.2621	0.002	0.892940731	NA	NA	NA	Bacteria	Bacteria	intra
SBS I vs. Control	Actinomadura luteofluorescens	Vescimonas fastidiosa	NA	NA	NA	0.2578	0.001	0.910156812	Bacteria	Bacteria	intra
SBS I vs. Control	Arthrobacter sp. CDRTa11	Gluconobacter oxydans	NA	NA	NA	0.2501	0.001	0.910156812	Bacteria	Bacteria	intra
SBS I vs. Control	Mixta hanseatica	Streptococcus pneumoniae	NA	NA	NA	-0.2535	0.002	0.917547512	Bacteria	Bacteria	intra
SBS I vs. Control	Salipaludibacillus sp. LMS25	uncultured Desulfobacter sp.	NA	NA	NA	0.2517	0.004	0.917547512	Bacteria	Bacteria	intra
SBS I vs. Control	Streptococcus gordonii	Trueperella abortisuis	NA	NA	NA	0.2705	0.001	0.910156812	Bacteria	Bacteria	intra
SBS II vs. Control	Alicyclobacillus dauci	Candidatus Sulfuricurvum sp. RIFRC-1	-0.2073	0.002	0.551417125	NA	NA	NA	Bacteria	Bacteria	intra
SBS II vs. Control	Alicyclobacillus sp. ALC3	Oxalobacter formigenes	-0.2061	0.003	0.551417125	NA	NA	NA	Bacteria	Bacteria	intra
SBS II vs. Control	Alkalimarinus coralli	Paraliobacillus zengyii	-0.2084	0.004	0.551417125	NA	NA	NA	Bacteria	Bacteria	intra
SBS II vs. Control	Anaerococcus prevotii	Clostridium cadaveris	0.2037	0.002	0.551417125	NA	NA	NA	Bacteria	Bacteria	intra
SBS II vs. Control	Anaerococcus prevotii	Geobacillus thermodenitrificans	0.2354	0.001	0.551417125	NA	NA	NA	Bacteria	Bacteria	intra
SBS II vs.	Anaerostipes hadrus	Staphylococcus chromogenes	-0.2556	0.003	0.551417125	NA	NA	NA	Bacteria	Bacteria	intra

Control											
SBS II vs. Control	Anaerotignum sp. MB30-C6	Vibrio metschnikovii	0.241	0.003	0.551417125	NA	NA	NA	Bacteria	Bacteria	intra
SBS II vs. Control	Arthrobacter sp. StoSoilA2	Pontibacter pudoricolor	-0.2037	0.004	0.551417125	NA	NA	NA	Bacteria	Bacteria	intra
SBS II vs. Control	Aspergillus caelatus	Roseovarius faecimaris	-0.2276	0.004	0.551417125	NA	NA	NA	Fungi	Bacteria	inter
SBS II vs. Control	Aspergillus neoniger	Blautia hansenii	-0.2279	0.003	0.551417125	NA	NA	NA	Fungi	Bacteria	inter
SBS II vs. Control	Azospira sp. I09	Thomasclavelia spiroformis	0.2545	0.004	0.551417125	NA	NA	NA	Bacteria	Bacteria	intra
SBS II vs. Control	Bacillus sp. Y1	Streptococcus pasteurianus	0.2039	0.004	0.551417125	NA	NA	NA	Bacteria	Bacteria	intra
SBS II vs. Control	Bibersteinia trehalosi	Pontibacter pudoricolor	0.2006	0.003	0.551417125	NA	NA	NA	Bacteria	Bacteria	intra
SBS II vs. Control	Blautia producta	Streptococcus pasteurianus	-0.2215	0.004	0.551417125	NA	NA	NA	Bacteria	Bacteria	intra
SBS II vs. Control	Brevibacillus sp. WF146	Paenibacillus terrae	-0.2075	0.004	0.551417125	NA	NA	NA	Bacteria	Bacteria	intra
SBS II vs. Control	Butyrivibrio fibrisolvens	Hahella chejuensis	0.2892	0.002	0.551417125	NA	NA	NA	Bacteria	Bacteria	intra
SBS II vs. Control	Butyrivibrio proteoclasticus	Gracilibacillus salitolerans	-0.2163	0.003	0.551417125	NA	NA	NA	Bacteria	Bacteria	intra
SBS II vs. Control	Butyrivibrio proteoclasticus	Pseudonocardia autotrophica	-0.2155	0.003	0.551417125	NA	NA	NA	Bacteria	Bacteria	intra
SBS II vs. Control	Caldicellulosiruptor morganii	Talaromyces stipitatus	-0.2135	0.004	0.551417125	NA	NA	NA	Bacteria	Bacteria	intra
SBS II vs. Control	Candida jiufengensis	Paenibacillus sp. RC67	0.2387	0.002	0.551417125	NA	NA	NA	Fungi	Bacteria	inter
SBS II vs. Control	Candidatus Pelagibacter sp. FZCC0015	Lysinibacillus sp. 2017	0.2029	0.004	0.551417125	NA	NA	NA	Bacteria	Bacteria	intra
SBS II vs. Control	Capnocytophaga gingivalis	Geobacillus subterraneus	-0.257	0.004	0.551417125	NA	NA	NA	Bacteria	Bacteria	intra
SBS II vs. Control	Cardiobacterium sp. Marseille-Q4385	Haploplasma axanthum	0.2034	0.002	0.551417125	NA	NA	NA	Bacteria	Bacteria	intra
SBS II vs. Control	Cellvibrio sp. PSBB006	Limosilactobacillus gastricus	-0.2741	0.001	0.551417125	NA	NA	NA	Bacteria	Bacteria	intra
SBS II vs. Control	Christensenella minuta	Clostridium taeniosporum	-0.2041	0.002	0.551417125	NA	NA	NA	Bacteria	Bacteria	intra
SBS II vs. Control	Clostridium bornimense	Streptomyces sp. JB150	0.2877	0.002	0.551417125	NA	NA	NA	Bacteria	Bacteria	intra

SBS II vs. Control	Clostridium drakei	Jeotgalibaca arthritidis	0.2037	0.002	0.551417125	NA	NA	NA	Bacteria	Bacteria	intra
SBS II vs. Control	Clostridium scatologenes	Desulfitobacterium metallireducens	-0.2235	0.004	0.551417125	NA	NA	NA	Bacteria	Bacteria	intra
SBS II vs. Control	Clostridium septicum	Tissierella sp. MB52-C2	0.299	0.004	0.551417125	NA	NA	NA	Bacteria	Bacteria	intra
SBS II vs. Control	Clostridium sp. AWRP	Xenorhabdus doucetiae	-0.2531	0.0038	0.551417125	NA	NA	NA	Bacteria	Bacteria	intra
SBS II vs. Control	Cryptococcus amylolentus	Macrococcus armenti	0.2242	0.001	0.551417125	NA	NA	NA	Fungi	Bacteria	inter
SBS II vs. Control	Desulfitobacterium dichloroeliminans	Mammaliicoccus sciuri	-0.2311	0.004	0.551417125	NA	NA	NA	Bacteria	Bacteria	intra
SBS II vs. Control	Didymosphaeria variabile	Legionella sp. PC997	-0.2945	0.004	0.551417125	NA	NA	NA	Fungi	Bacteria	inter
SBS II vs. Control	Dorea longicatena	Lysinibacillus irui	-0.209	0.002	0.551417125	NA	NA	NA	Bacteria	Bacteria	intra
SBS II vs. Control	Enterocloster clostridioformis	Paenibacillus sp. FSL K6-3182	-0.2117	0.003	0.551417125	NA	NA	NA	Bacteria	Bacteria	intra
SBS II vs. Control	Faecalibacterium sp. IP-3-29	Pasteurella skyensis	0.2285	0.003	0.551417125	NA	NA	NA	Bacteria	Bacteria	intra
SBS II vs. Control	Hymenobacter sp. YIM 151500-1	Lactobacillus gasseri	0.2249	0.004	0.551417125	NA	NA	NA	Bacteria	Bacteria	intra
SBS II vs. Control	Janthinobacterium sp. HH102	Paenibacillus sabinae	-0.2438	0.001	0.551417125	NA	NA	NA	Bacteria	Bacteria	intra
SBS II vs. Control	Kocuria sp. KD4	Roseburia sp. 499	-0.2077	0.003	0.551417125	NA	NA	NA	Bacteria	Bacteria	intra
SBS II vs. Control	Lacimicrobium alkaliphilum	Streptomyces thermocarboxydus	-0.2555	0.003	0.551417125	NA	NA	NA	Bacteria	Bacteria	intra
SBS II vs. Control	Lactobacillus sp. 3B(2020)	uncultured Amphritea sp.	0.2348	0.004	0.551417125	NA	NA	NA	Bacteria	Bacteria	intra
SBS II vs. Control	Legionella micdadei	Thermomonas paludicola	-0.2556	0.002	0.551417125	NA	NA	NA	Bacteria	Bacteria	intra
SBS II vs. Control	Lysinibacillus parviboronicapiens	Paenisporosarcina antarctica	-0.3105	0.001	0.551417125	NA	NA	NA	Bacteria	Bacteria	intra
SBS II vs. Control	Mycoplasma mycoides	Paracrocinitomix mangrovi	0.2518	0.004	0.551417125	NA	NA	NA	Bacteria	Bacteria	intra
SBS II vs. Control	Paenibacillus typhae	Thermosediminibacter oceani	-0.2006	0.004	0.551417125	NA	NA	NA	Bacteria	Bacteria	intra
SBS II vs. Control	Pontibacter kalidii	Wohlfahrtiimonas chitiniclastica	-0.2519	0.004	0.551417125	NA	NA	NA	Bacteria	Bacteria	intra
SBS II vs.	Rosistilla carotiformis	Sphingosinicella flava	-0.2109	0.003	0.551417125	NA	NA	NA	Bacteria	Bacteria	intra

Control											
SBS II vs. Control	Sporosarcina sp. B2O-1	Wujia chipingensis	0.2251	0.004	0.551417125	NA	NA	NA	Bacteria	Bacteria	intra
SBS II vs. Control	Acetobacter tropicalis	Ignavibacterium sp.	NA	NA	NA	0.2297	0.001	0.900980392	Bacteria	Bacteria	intra
SBS II vs. Control	Acinetobacter equi	Deinococcus irradiatisoli	NA	NA	NA	0.2393	0.001	0.900980392	Bacteria	Bacteria	intra
SBS II vs. Control	Actinotignum schaalii	Corynebacterium faecale	NA	NA	NA	0.2691	0.004	0.900980392	Bacteria	Bacteria	intra
SBS II vs. Control	Aeromonas caviae	Anaerobutyricum hallii	NA	NA	NA	0.2139	0.001	0.900980392	Bacteria	Bacteria	intra
SBS II vs. Control	Agrobacterium sp. RAC06	Paenibacillus sp. MBLB1832	NA	NA	NA	0.2106	0.004	0.900980392	Bacteria	Bacteria	intra
SBS II vs. Control	Agrobacterium sp. RAC06	Ruminococcus bicirculans (ex Wegman et al. 2014)	NA	NA	NA	-0.2068	0.001	0.900980392	Bacteria	Bacteria	intra
SBS II vs. Control	Alkalicoccus halolimnae	Talaromyces stipitatus	NA	NA	NA	0.2195	0.004	0.900980392	Bacteria	Bacteria	intra
SBS II vs. Control	Anaerocolumna sp. AGMB13020	Lactococcus cremoris	NA	NA	NA	-0.215	0.002	0.900980392	Bacteria	Bacteria	intra
SBS II vs. Control	Arthrobacter sp. StoSoilB22	Ehrlichia ruminantium	NA	NA	NA	0.2046	0.001	0.900980392	Bacteria	Bacteria	intra
SBS II vs. Control	Aspergillus caelatus	Halomicroarcula limicola	NA	NA	NA	0.2052	0.001	0.900980392	Fungi	Archaea	inter
SBS II vs. Control	Bacteroides sp. A1C1	Dothidotthia symphoricarpi	NA	NA	NA	0.2074	0.001	0.900980392	Bacteria	Fungi	inter
SBS II vs. Control	Blautia sp. SC05B48	Herbinix luporum	NA	NA	NA	0.2149	0.001	0.900980392	Bacteria	Bacteria	intra
SBS II vs. Control	Brevibacterium sandarakinum	Streptomyces thermocarboxydus	NA	NA	NA	-0.212	0.001	0.900980392	Bacteria	Bacteria	intra
SBS II vs. Control	Caminicella sporogenes	Paraclostridium sordellii	NA	NA	NA	-0.2207	0.002	0.900980392	Bacteria	Bacteria	intra
SBS II vs. Control	Candidatus Mycosynbacter amalyticus	Pasteurella dagmatis	NA	NA	NA	0.2095	0.002	0.900980392	Bacteria	Bacteria	intra
SBS II vs. Control	Cellulophaga sp. L1A9	Spirosoma sp. KUDC1026	NA	NA	NA	-0.2391	0.002	0.900980392	Bacteria	Bacteria	intra
SBS II vs. Control	Chlorobium limicola	Staphylococcus rostri	NA	NA	NA	0.2242	0.002	0.900980392	Bacteria	Bacteria	intra
SBS II vs. Control	Chryseobacterium gambrini	[Ruminococcus] lactaris	NA	NA	NA	0.2234	0.001	0.900980392	Bacteria	Bacteria	intra
SBS II vs. Control	Clostridium argentinense	Thermosediminibacter oceani	NA	NA	NA	0.2072	0.004	0.900980392	Bacteria	Bacteria	intra

SBS II vs. Control	Clostridium cadaveris	Pectobacterium wasabiae	NA	NA	NA	0.2383	0.004	0.900980392	Bacteria	Bacteria	intra
SBS II vs. Control	Clostridium gelidum	Halothiobacillus neapolitanus	NA	NA	NA	-0.2116	0.001	0.900980392	Bacteria	Bacteria	intra
SBS II vs. Control	Clostridium septicum	Oceanirhabdus sp. W0125-5	NA	NA	NA	0.2683	0.001	0.900980392	Bacteria	Bacteria	intra
SBS II vs. Control	Corynebacterium endometrii	Wujia chipingensis	NA	NA	NA	0.2102	0.003	0.900980392	Bacteria	Bacteria	intra
SBS II vs. Control	Corynebacterium pseudotuberculosis	Prevotella sp. Rep29	NA	NA	NA	-0.211	0.003	0.900980392	Bacteria	Bacteria	intra
SBS II vs. Control	Crassaminicella indica	Mycolicibacter minnesotensis	NA	NA	NA	0.21	0.004	0.900980392	Bacteria	Bacteria	intra
SBS II vs. Control	Cytobacillus kochii	Virgibacillus necropolis	NA	NA	NA	-0.2162	0.002	0.900980392	Bacteria	Bacteria	intra
SBS II vs. Control	Dictyoglomus thermophilum	Mycolicibacterium pallens	NA	NA	NA	0.2044	0.002	0.900980392	Bacteria	Bacteria	intra
SBS II vs. Control	Erysipelothrix enhydrae	Fusarium keratoplasticum	NA	NA	NA	0.2193	0.004	0.900980392	Bacteria	Fungi	inter
SBS II vs. Control	Ferrigenium kumadai	Intestinibacter bartlettii	NA	NA	NA	-0.2002	0.001	0.900980392	Bacteria	Bacteria	intra
SBS II vs. Control	Fusarium keratoplasticum	Pseudodesulfovibrio sp. 5S69	NA	NA	NA	-0.2327	0.002	0.900980392	Fungi	Bacteria	inter
SBS II vs. Control	Halobacillus shinanisalinarum	Wujia chipingensis	NA	NA	NA	-0.2188	0.001	0.900980392	Bacteria	Bacteria	intra
SBS II vs. Control	Halobaculum sp. DT92	Segatella oris	NA	NA	NA	-0.2287	0.001	0.900980392	Archaea	Bacteria	inter
SBS II vs. Control	Hungatella hathewayi	Lysinibacillus louembei	NA	NA	NA	0.2124	0.001	0.900980392	Bacteria	Bacteria	intra
SBS II vs. Control	Lactobacillus johnsonii	Pantoea sp. At-9b	NA	NA	NA	0.2085	0.004	0.900980392	Bacteria	Bacteria	intra
SBS II vs. Control	Legionella spiritensis	Moellerella wisconsensis	NA	NA	NA	0.2347	0.002	0.900980392	Bacteria	Bacteria	intra
SBS II vs. Control	Limosilactobacillus panis	Thermosediminibacter oceani	NA	NA	NA	0.2437	0.003	0.900980392	Bacteria	Bacteria	intra
SBS II vs. Control	Macrococcoides caseolyticum	Pseudomonas tritici	NA	NA	NA	-0.2005	0.001	0.900980392	Bacteria	Bacteria	intra
SBS II vs. Control	Maridesulfovibrio hydrothermalis	Neokomagataea tanensis	NA	NA	NA	0.2316	0.002	0.900980392	Bacteria	Bacteria	intra
SBS II vs. Control	Methanosarcina thermophila	Pasteurella dagmatis	NA	NA	NA	0.2016	0.001	0.900980392	Bacteria	Bacteria	intra
SBS II vs.	Moellerella wisconsensis	Shewanella baltica	NA	NA	NA	0.2067	0.001	0.900980	Bacteria	Bacteria	intra

Control								392			
SBS II vs. Control	Paenibacillus sabinae	Pseudoleptotrichia goodfellowii	NA	NA	NA	-0.2188	0.001	0.900980392	Bacteria	Bacteria	intra
SBS II vs. Control	Paenibacillus sabinae	Streptomyces sp. JB150	NA	NA	NA	-0.2223	0.002	0.900980392	Bacteria	Bacteria	intra
SBS II vs. Control	Paludicola sp. MB14-C6	Streptomyces sp. WZ-12	NA	NA	NA	-0.2291	0.001	0.900980392	Bacteria	Bacteria	intra
SBS II vs. Control	Phycicoccus endophyticus	Roseobacter ponti	NA	NA	NA	-0.2037	0.003	0.900980392	Bacteria	Bacteria	intra
SBS II vs. Control	Pseudoalteromonas phenolica	Thermomonas paludicola	NA	NA	NA	0.2003	0.001	0.900980392	Bacteria	Bacteria	intra
SBS II vs. Control	Psychrobacillus glaciei	Roseburia sp. 499	NA	NA	NA	-0.2064	0.001	0.900980392	Bacteria	Bacteria	intra
SBS II vs. Control	Streptococcus didelphis	Vibrio metschnikovii	NA	NA	NA	0.2051	0.001	0.900980392	Bacteria	Bacteria	intra
SBS II vs. Control	Streptomyces argyrophyllae	Talaromyces stipitatus	NA	NA	NA	0.2022	0.001	0.900980392	Bacteria	Bacteria	intra
SBS III vs. Control	Agromyces flavus	Halalkaliarchaeum desulfuricum	0.2303	0.003	1	NA	NA	NA	Bacteria	Bacteria	intra
SBS III vs. Control	Alicyclobacillus mengziensis	Entomospira culicis	-0.2446	0.001	1	NA	NA	NA	Bacteria	Bacteria	intra
SBS III vs. Control	Arthrobacter sp. U41	Rossellomorea marisflavi	0.2027	0.002	1	NA	NA	NA	Bacteria	Bacteria	intra
SBS III vs. Control	Bacillus sp. KH172YL63	Sphingobium sp. YC-XJ3	0.2379	0.001	1	NA	NA	NA	Bacteria	Bacteria	intra
SBS III vs. Control	Chryseobacterium manosquense	Litorilituus sediminis	0.2478	0.001	1	NA	NA	NA	Bacteria	Bacteria	intra
SBS III vs. Control	Leptospira sp. GIMC2001	Nitrosomonas eutropha	0.2449	0.004	1	NA	NA	NA	Bacteria	Bacteria	intra
SBS III vs. Control	Luteolibacter sp. SL250	Pseudomonas sp. DTU12.3	-0.2357	0.003	1	NA	NA	NA	Bacteria	Bacteria	intra
SBS III vs. Control	Marinithermus hydrothermalis	Vibrio vulnificus	0.2356	0.003	1	NA	NA	NA	Bacteria	Bacteria	intra
SBS III vs. Control	Nitrogeniibacter aestuarii	Sanguibacter sp. 4.1	-0.2562	0.002	1	NA	NA	NA	Bacteria	Bacteria	intra
SBS III vs. Control	Pseudomonas asplenii	Pseudomonas oryzae	-0.2097	0.002	1	NA	NA	NA	Bacteria	Bacteria	intra
SBS III vs. Control	Aaosphaeria arxii	Bradyrhizobium sp. CCBAU 53421	NA	NA	NA	-0.2062	0.001	0.869165865	Bacteria	Bacteria	intra
SBS III vs. Control	Acidothermus cellulolyticus	Photorhabdus asymbiotica	NA	NA	NA	-0.2111	0.001	0.869165865	Bacteria	Bacteria	intra

SBS III vs. Control	Acinetobacter pseudolwoffii	Staphylococcus pasteuri	NA	NA	NA	0.2179	0.002	0.869165865	Bacteria	Bacteria	intra
SBS III vs. Control	Actinoplanes sp. N902-109	Streptomyces sp. NBC_00162	NA	NA	NA	0.242	0.001	0.869165865	Bacteria	Bacteria	intra
SBS III vs. Control	Actinoplanes sp. OR16	Tumebacillus avium	NA	NA	NA	-0.2026	0.002	0.869165865	Bacteria	Bacteria	intra
SBS III vs. Control	Aeribacillus sp. FSL W8-0870	Vibrio sp. B1FLJ16	NA	NA	NA	0.2376	0.003	0.869165865	Bacteria	Bacteria	intra
SBS III vs. Control	Alistipes senegalensis	Brucella anthropi	NA	NA	NA	0.2149	0.001	0.869165865	Bacteria	Bacteria	intra
SBS III vs. Control	Alkalimarinus coralli	Photobacterium swingsii	NA	NA	NA	0.2029	0.001	0.869165865	Bacteria	Bacteria	intra
SBS III vs. Control	Alkalimarinus coralli	uncultured Methanocorpusculum sp.	NA	NA	NA	0.219	0.001	0.869165865	Bacteria	Bacteria	intra
SBS III vs. Control	Alysiella filiformis	Methylomusa anaerophila	NA	NA	NA	-0.2101	0.001	0.869165865	Bacteria	Bacteria	intra
SBS III vs. Control	Ammonifex degensii	Pantoea sp. Z09	NA	NA	NA	0.2255	0.002	0.869165865	Bacteria	Bacteria	intra
SBS III vs. Control	Ammonifex degensii	Pseudomonas sp. AO-1	NA	NA	NA	0.2399	0.002	0.869165865	Bacteria	Bacteria	intra
SBS III vs. Control	Aquimarina sp. TRL1	Campylobacter avium	NA	NA	NA	-0.3108	0.002	0.869165865	Bacteria	Bacteria	intra
SBS III vs. Control	Arcanobacterium canis	Pseudomonas cedrina	NA	NA	NA	0.2004	0.001	0.869165865	Bacteria	Bacteria	intra
SBS III vs. Control	Arcobacter nitrofigilis	Candidatus Thioglobus sp. NP1	NA	NA	NA	0.2014	0.003	0.869165865	Bacteria	Bacteria	intra
SBS III vs. Control	Arcobacter peruensis	Paenibacillus tianjinensis	NA	NA	NA	-0.2038	0.003	0.869165865	Bacteria	Bacteria	intra
SBS III vs. Control	Aromatoleum petrolei	Desulfobaculum bizertense	NA	NA	NA	-0.2373	0.002	0.869165865	Bacteria	Bacteria	intra
SBS III vs. Control	Arthrobacter sp. D5-1	Suillus bovinus	NA	NA	NA	-0.2458	0.001	0.869165865	Bacteria	Bacteria	intra
SBS III vs. Control	Arthrobacter sp. PM3	Hydrogenibacillus sp. N12	NA	NA	NA	-0.2056	0.002	0.869165865	Bacteria	Bacteria	intra
SBS III vs. Control	Arthrobacter sunyaminii	Penicillium mononematosum	NA	NA	NA	-0.227	0.003	0.869165865	Bacteria	Bacteria	intra
SBS III vs. Control	Aspergillus aculeatus	Aspergillus uvarum	NA	NA	NA	0.2137	0.004	0.869165865	Bacteria	Bacteria	intra
SBS III vs. Control	Aspergillus eucalypticola	Candidatus Nanopusillus massiliensis	NA	NA	NA	-0.2191	0.003	0.869165865	Bacteria	Bacteria	intra
SBS III vs.	Aspergillus luchuensis	Candidatus Gullanella endobia	NA	NA	NA	-0.2241	0.003	0.869165	Bacteria	Bacteria	intra

Control								865			
SBS III vs. Control	Aspergillus niger	Ligilactobacillus animalis	NA	NA	NA	0.2115	0.004	0.869165 865	Bacteria	Bacteria	intra
SBS III vs. Control	Athalassotoga saccharophila	Cyanobacterium sp. Dongsha4	NA	NA	NA	0.2124	0.004	0.869165 865	Bacteria	Bacteria	intra
SBS III vs. Control	Athalassotoga saccharophila	Streptomyces aurantiacus	NA	NA	NA	-0.2148	0.001	0.869165 865	Bacteria	Bacteria	intra
SBS III vs. Control	Bacillus cereus	Brevibacillus composti	NA	NA	NA	-0.232	0.002	0.869165 865	Bacteria	Bacteria	intra
SBS III vs. Control	Bacillus cereus	Halorubrum aethiopicum	NA	NA	NA	0.203	0.002	0.869165 865	Bacteria	Bacteria	intra
SBS III vs. Control	Bacillus paralicheniformis	Sebaldella termitidis	NA	NA	NA	0.2819	0.004	0.869165 865	Bacteria	Bacteria	intra
SBS III vs. Control	Bacillus safensis	Streptomyces erythrochromogenes	NA	NA	NA	0.2113	0.002	0.869165 865	Bacteria	Bacteria	intra
SBS III vs. Control	Bacillus vallismortis	Bartonella sp. TP	NA	NA	NA	-0.2049	0.001	0.869165 865	Bacteria	Bacteria	intra
SBS III vs. Control	Bacillus vallismortis	Solitalea lacus	NA	NA	NA	0.2179	0.002	0.869165 865	Bacteria	Bacteria	intra
SBS III vs. Control	Bacillus velezensis	Uncinocarpus reesii	NA	NA	NA	-0.2082	0.003	0.869165 865	Bacteria	Bacteria	intra
SBS III vs. Control	Bacteroides sp. MSB163	Streptomyces sp. HUAS CB01	NA	NA	NA	-0.2096	0.002	0.869165 865	Bacteria	Bacteria	intra
SBS III vs. Control	Bifidobacterium eulemuris	Comamonas fluminis	NA	NA	NA	0.234	0.003	0.869165 865	Bacteria	Bacteria	intra
SBS III vs. Control	Bifidobacterium sp. ESL0682	Carnobacterium divergens	NA	NA	NA	-0.2085	0.003	0.869165 865	Bacteria	Bacteria	intra
SBS III vs. Control	Blautia wexlerae	Sporosarcina pasteurii	NA	NA	NA	0.2222	0.001	0.869165 865	Bacteria	Bacteria	intra
SBS III vs. Control	Blochmannia endosymbiont of Camponotus (Colobopsis) obliquus	Peribacillus simplex	NA	NA	NA	0.2024	0.001	0.869165 865	Bacteria	Bacteria	intra
SBS III vs. Control	Bombilactobacillus bombi	Streptomyces fradiae	NA	NA	NA	-0.2413	0.002	0.869165 865	Bacteria	Bacteria	intra
SBS III vs. Control	Borreliella burgdorferi	Nocardiopsis dassonvillei	NA	NA	NA	0.2286	0.004	0.869165 865	Bacteria	Bacteria	intra
SBS III vs. Control	Bosea vaviloviae	Winogradskyella sediminis	NA	NA	NA	0.2011	0.003	0.869165 865	Bacteria	Bacteria	intra
SBS III vs. Control	Bradyrhizobium arachidis	Staphylococcus debuckii	NA	NA	NA	0.2372	0.001	0.869165 865	Bacteria	Bacteria	intra
SBS III vs.	Bradyrhizobium ottawaense	Candidatus Desulfovibrio	NA	NA	NA	0.2288	0.004	0.869165	Bacteria	Bacteria	intra

Control		trichonymphae						865			
SBS III vs. Control	Brevibacillus brevis	Streptococcus thermophilus	NA	NA	NA	-0.2053	0.001	0.869165 865	Bacteria	Bacteria	intra
SBS III vs. Control	Brevibacillus composti	Cruoricaptor ignavus	NA	NA	NA	0.2455	0.002	0.869165 865	Bacteria	Bacteria	intra
SBS III vs. Control	Brevibacillus sp. WF146	Halorubrum aethiopicum	NA	NA	NA	-0.2106	0.002	0.869165 865	Bacteria	Bacteria	intra
SBS III vs. Control	Brucella pseudintermedia	Nostoc flagelliforme	NA	NA	NA	-0.2114	0.001	0.869165 865	Bacteria	Bacteria	intra
SBS III vs. Control	Burkholderia sp. IDO3	Paraliobacillus zengyii	NA	NA	NA	-0.2349	0.003	0.869165 865	Bacteria	Bacteria	intra
SBS III vs. Control	Butyricimonas virosa	Ureibacillus thermophilus	NA	NA	NA	0.2026	0.003	0.869165 865	Bacteria	Bacteria	intra
SBS III vs. Control	Caballeronia sp. SBC2	Halorubrum aethiopicum	NA	NA	NA	-0.223	0.002	0.869165 865	Bacteria	Bacteria	intra
SBS III vs. Control	Caldanaerobacter subterraneus	Clostridium cochlearium	NA	NA	NA	0.2171	0.003	0.869165 865	Bacteria	Bacteria	intra
SBS III vs. Control	Caldicellulosiruptor morganii	Comamonas odontotermitis	NA	NA	NA	-0.225	0.002	0.869165 865	Bacteria	Bacteria	intra
SBS III vs. Control	Caldicellulosiruptor morganii	Leptospira noguchii	NA	NA	NA	0.2715	0.003	0.869165 865	Bacteria	Bacteria	intra
SBS III vs. Control	Caldisphaera sp.	Hyaloscypha bicolor	NA	NA	NA	0.2093	0.001	0.869165 865	Bacteria	Bacteria	intra
SBS III vs. Control	Campylobacter curvus	Saccharomycodes ludwigii	NA	NA	NA	0.2066	0.002	0.869165 865	Bacteria	Bacteria	intra
SBS III vs. Control	Campylobacter ureolyticus	Peribacillus sp. R9-11	NA	NA	NA	-0.2332	0.003	0.869165 865	Bacteria	Bacteria	intra
SBS III vs. Control	Candidatus Nanopelagicus abundans	Cellulomonas wangleii	NA	NA	NA	-0.2004	0.001	0.869165 865	Bacteria	Bacteria	intra
SBS III vs. Control	Candidatus Planktophila limnetica	Clavispora lusitaniae	NA	NA	NA	0.2194	0.003	0.869165 865	Bacteria	Bacteria	intra
SBS III vs. Control	Candidatus Planktophila limnetica	Parabacteroides chongii	NA	NA	NA	-0.2283	0.001	0.869165 865	Bacteria	Bacteria	intra
SBS III vs. Control	Candidatus Profftia lariciata	Spirochaeta africana	NA	NA	NA	0.2021	0.002	0.869165 865	Bacteria	Bacteria	intra
SBS III vs. Control	Candidatus Schneideria nysicola	Cellvibrio sp. KY-GH-1	NA	NA	NA	-0.2038	0.002	0.869165 865	Bacteria	Bacteria	intra
SBS III vs. Control	Candidatus Sulfurimonas marisnigri	Paenibacillus lutimineralis	NA	NA	NA	0.2016	0.004	0.869165 865	Bacteria	Bacteria	intra
SBS III vs. Control	Caulobacter soli	Polaribacter sp. SA4-10	NA	NA	NA	0.2283	0.002	0.869165 865	Bacteria	Bacteria	intra

SBS III vs. Control	Cetobacterium somerae	Microbacterium sp. KUDC0406	NA	NA	NA	-0.2449	0.003	0.869165865	Bacteria	Bacteria	intra
SBS III vs. Control	Citrobacter sp. Colony219	Kosakonia sacchari	NA	NA	NA	-0.2201	0.001	0.869165865	Bacteria	Bacteria	intra
SBS III vs. Control	Clostridium drakei	Novacetimonas hansenii	NA	NA	NA	-0.21	0.003	0.869165865	Bacteria	Bacteria	intra
SBS III vs. Control	Clostridium estertheticum	Xenorhabdus hominickii	NA	NA	NA	-0.239	0.001	0.869165865	Bacteria	Bacteria	intra
SBS III vs. Control	Clostridium sp. CM028	Microbacterium sp. BH-3-3-3	NA	NA	NA	0.2122	0.001	0.869165865	Bacteria	Bacteria	intra
SBS III vs. Control	Clostridium thermarum	Desulfoferula mesophilus	NA	NA	NA	0.2145	0.002	0.869165865	Bacteria	Bacteria	intra
SBS III vs. Control	Colwellia sp. Arc7-D	Spiroplasma sp. SV19	NA	NA	NA	0.2036	0.003	0.869165865	Bacteria	Bacteria	intra
SBS III vs. Control	Corynebacterium genitalium	Staphylococcus debuckii	NA	NA	NA	-0.2028	0.001	0.869165865	Bacteria	Bacteria	intra
SBS III vs. Control	Corynebacterium sanguinis	Methanococcus voltae	NA	NA	NA	0.2158	0.003	0.869165865	Bacteria	Bacteria	intra
SBS III vs. Control	Cryptobacterium curtum	Exiguobacterium profundum	NA	NA	NA	0.2436	0.001	0.869165865	Bacteria	Bacteria	intra
SBS III vs. Control	Cryptococcus wingfieldii	Pseudoduganella sp. SL102	NA	NA	NA	-0.2018	0.001	0.869165865	Bacteria	Bacteria	intra
SBS III vs. Control	Curtobacterium sp. MCSS17_007	Gilliamella sp. ESL0441	NA	NA	NA	-0.2082	0.002	0.869165865	Bacteria	Bacteria	intra
SBS III vs. Control	Cyberlindnera jadinii	Paenibacillus tianjinensis	NA	NA	NA	0.2302	0.001	0.869165865	Bacteria	Bacteria	intra
SBS III vs. Control	Deinococcus actinosclerus	Polynucleobacter sp. JS-JIR-II-50	NA	NA	NA	0.2172	0.002	0.869165865	Bacteria	Bacteria	intra
SBS III vs. Control	Desulfobaculum bizertense	Microbacterium sp. ET2	NA	NA	NA	0.2262	0.001	0.869165865	Bacteria	Bacteria	intra
SBS III vs. Control	Desulforamulus reducens	Thermoanaerobacterium sp. RBIITD	NA	NA	NA	0.2004	0.001	0.869165865	Bacteria	Bacteria	intra
SBS III vs. Control	Emergencia sp. JLR.KK010	Fusobacterium vincentii	NA	NA	NA	0.2013	0.002	0.869165865	Bacteria	Bacteria	intra
SBS III vs. Control	Encephalitozoon cuniculi	Vulcanisaeta sp. JCM 16161	NA	NA	NA	-0.2207	0.003	0.869165865	Bacteria	Bacteria	intra
SBS III vs. Control	Enterobacteriaceae endosymbiont of Donacia vulgaris	Vibrio gazogenes	NA	NA	NA	-0.2105	0.003	0.869165865	Bacteria	Bacteria	intra
SBS III vs. Control	Eremothecium sinecaudum	Shewanella algae	NA	NA	NA	-0.2258	0.004	0.869165865	Bacteria	Bacteria	intra

SBS III vs. Control	Erwinia tasmaniensis	Sphingomonas qomolangmaensis	NA	NA	NA	-0.2047	0.002	0.869165865	Bacteria	Bacteria	intra
SBS III vs. Control	Erysipelothrix piscisicarius	Isosphaera pallida	NA	NA	NA	-0.2238	0.001	0.869165865	Bacteria	Bacteria	intra
SBS III vs. Control	Escherichia coli	Limosilactobacillus frumenti	NA	NA	NA	-0.2136	0.001	0.869165865	Bacteria	Bacteria	intra
SBS III vs. Control	Fluviibacter phosphoraccumulans	Mycolicibacter terrae	NA	NA	NA	-0.2043	0.001	0.869165865	Bacteria	Bacteria	intra
SBS III vs. Control	Fluviibacter phosphoraccumulans	Streptomyces sp. ST1015	NA	NA	NA	-0.2226	0.002	0.869165865	Bacteria	Bacteria	intra
SBS III vs. Control	Francisella philomiragia	Mediterraneibacter gnavus	NA	NA	NA	-0.2149	0.003	0.869165865	Bacteria	Bacteria	intra
SBS III vs. Control	Fusarium solani	Mariniplasma anaerobium	NA	NA	NA	0.206	0.001	0.869165865	Bacteria	Bacteria	intra
SBS III vs. Control	Fusobacterium sp. oral taxon 203	Ruficoccus sp. ZRK36	NA	NA	NA	-0.2589	0.003	0.869165865	Bacteria	Bacteria	intra
SBS III vs. Control	Fusobacterium vincentii	Methanocaldococcus fervens	NA	NA	NA	0.2011	0.003	0.869165865	Bacteria	Bacteria	intra
SBS III vs. Control	Gemella sp. oral taxon 928	Mucilaginibacter sabulilitoris	NA	NA	NA	-0.2141	0.003	0.869165865	Bacteria	Bacteria	intra
SBS III vs. Control	Geomonas sp. RF6	Kocuria sp. KD4	NA	NA	NA	0.2207	0.002	0.869165865	Bacteria	Bacteria	intra
SBS III vs. Control	Gilbertella persicaria	Vibrio natriegens	NA	NA	NA	-0.2222	0.004	0.869165865	Bacteria	Bacteria	intra
SBS III vs. Control	Gimesia fumaroli	Methanotorris formicicus	NA	NA	NA	-0.2175	0.001	0.869165865	Bacteria	Bacteria	intra
SBS III vs. Control	Gimesia panareensis	Pectobacterium wasabiae	NA	NA	NA	-0.248	0.001	0.869165865	Bacteria	Bacteria	intra
SBS III vs. Control	Gordonia amicalis	Synchytrium microbalum	NA	NA	NA	-0.2134	0.002	0.869165865	Bacteria	Bacteria	intra
SBS III vs. Control	Gramella sp. MAR_2010_147	Psychrobacter urativorans	NA	NA	NA	-0.205	0.002	0.869165865	Bacteria	Bacteria	intra
SBS III vs. Control	Halalkalicoccus tibetensis	Neokomagataea tanensis	NA	NA	NA	0.2004	0.004	0.869165865	Bacteria	Bacteria	intra
SBS III vs. Control	Halanaerobium hydrogeniformans	Haloplanus sp. GDY1	NA	NA	NA	0.2067	0.002	0.869165865	Bacteria	Bacteria	intra
SBS III vs. Control	Halococcus sediminicola	Weeksella virosa	NA	NA	NA	0.2115	0.001	0.869165865	Bacteria	Bacteria	intra
SBS III vs. Control	Halomarina sp. PSRA2	Natronolimnohabitans sp. A-GB9	NA	NA	NA	0.2541	0.001	0.869165865	Bacteria	Bacteria	intra
SBS III vs.	Halorhodospira halophila	Klebsiella sp. WP3-W18-ESBL-02	NA	NA	NA	0.2272	0.003	0.869165	Bacteria	Bacteria	intra

Control								865			
SBS III vs. Control	Hyaloscypha bicolor	Pseudazoarcus pumilus	NA	NA	NA	-0.2089	0.004	0.869165 865	Bacteria	Bacteria	intra
SBS III vs. Control	Hyphopichia burtonii	Solibacillus sp. FSL R5-0691	NA	NA	NA	0.2214	0.001	0.869165 865	Bacteria	Bacteria	intra
SBS III vs. Control	Ignavibacterium sp.	Limnohabitans sp. TEGF004	NA	NA	NA	-0.2308	0.001	0.869165 865	Bacteria	Bacteria	intra
SBS III vs. Control	Intestinibacter bartlettii	Vibrio natriegens	NA	NA	NA	-0.2222	0.003	0.869165 865	Bacteria	Bacteria	intra
SBS III vs. Control	Jeotgalibaca arthritidis	Maribacter dokdonensis	NA	NA	NA	0.2153	0.004	0.869165 865	Bacteria	Bacteria	intra
SBS III vs. Control	Kitasatospora aureofaciens	Parabacteroides chongii	NA	NA	NA	-0.2337	0.001	0.869165 865	Bacteria	Bacteria	intra
SBS III vs. Control	Klebsiella sp. WP3-W18-ESBL-02	Thiothrix winogradskyi	NA	NA	NA	-0.201	0.004	0.869165 865	Bacteria	Bacteria	intra
SBS III vs. Control	Kordia antarctica	Sphingobium sp. YBL2	NA	NA	NA	-0.2054	0.001	0.869165 865	Bacteria	Bacteria	intra
SBS III vs. Control	Kordiimonas sp. SCSIO 12603	Tissierella sp. Yu-01	NA	NA	NA	-0.2199	0.001	0.869165 865	Bacteria	Bacteria	intra
SBS III vs. Control	Lactiplantibacillus pentosus	Mesomycoplasma bovoculi	NA	NA	NA	0.2114	0.001	0.869165 865	Bacteria	Bacteria	intra
SBS III vs. Control	Lactiplantibacillus pentosus	Thalassospira indica	NA	NA	NA	-0.2218	0.002	0.869165 865	Bacteria	Bacteria	intra
SBS III vs. Control	Lactobacillus sp. ESL0677	Pseudomonas sp. ADAK18	NA	NA	NA	-0.2026	0.001	0.869165 865	Bacteria	Bacteria	intra
SBS III vs. Control	Lactococcus sp. LG606	Streptomyces sp. SM18	NA	NA	NA	0.2167	0.001	0.869165 865	Bacteria	Bacteria	intra
SBS III vs. Control	Letharia columbiana	Paenibacillus sp. FSL E2-0190	NA	NA	NA	0.2042	0.003	0.869165 865	Bacteria	Bacteria	intra
SBS III vs. Control	Leuconostoc mesenteroides	Paenibacillus psychroresistens	NA	NA	NA	-0.2088	0.001	0.869165 865	Bacteria	Bacteria	intra
SBS III vs. Control	Limnohabitans sp. MORI2	Pseudomonas sp. ADAK13	NA	NA	NA	0.2136	0.004	0.869165 865	Bacteria	Bacteria	intra
SBS III vs. Control	Limnohabitans sp. MORI2	Rasamsonia emersonii	NA	NA	NA	-0.2089	0.002	0.869165 865	Bacteria	Bacteria	intra
SBS III vs. Control	Litorilituus sediminis	Romboutsia ilealis	NA	NA	NA	0.2014	0.001	0.869165 865	Bacteria	Bacteria	intra
SBS III vs. Control	Lysinibacillus sp. 2017	Novacetimonas hansenii	NA	NA	NA	0.2213	0.001	0.869165 865	Bacteria	Bacteria	intra
SBS III vs. Control	Macrococcoides caseolyticum	Rhizobium gei	NA	NA	NA	0.2114	0.002	0.869165 865	Bacteria	Bacteria	intra

SBS III vs. Control	Macrococcus epidermidis	Natronococcus sp.	NA	NA	NA	0.2357	0.001	0.869165865	Bacteria	Bacteria	intra
SBS III vs. Control	Malassezia pachydermatis	Rhizobium sp. 11515TR	NA	NA	NA	-0.2076	0.002	0.869165865	Bacteria	Bacteria	intra
SBS III vs. Control	Mammaliicoccus sp. Marseille-Q6498	Polaribacter litorisediminis	NA	NA	NA	0.2195	0.001	0.869165865	Bacteria	Bacteria	intra
SBS III vs. Control	Marinobacter shengliensis	Moraxella haemolytica	NA	NA	NA	-0.2156	0.003	0.869165865	Bacteria	Bacteria	intra
SBS III vs. Control	Marinobacter shengliensis	Mycobacterium lentiflavum	NA	NA	NA	0.2096	0.001	0.869165865	Bacteria	Bacteria	intra
SBS III vs. Control	Marinomonas sp. FW-1	Methylocystis bryophila	NA	NA	NA	-0.2157	0.001	0.869165865	Bacteria	Bacteria	intra
SBS III vs. Control	Marivirga sp. ABR2-2	Streptomyces tanashiensis	NA	NA	NA	-0.201	0.001	0.869165865	Bacteria	Bacteria	intra
SBS III vs. Control	Massilia endophytica	Penicilliopsis zonata	NA	NA	NA	0.254	0.001	0.869165865	Bacteria	Bacteria	intra
SBS III vs. Control	Mesorhizobium opportunistum	Mesorhizobium sp. 131-3-5	NA	NA	NA	-0.2014	0.002	0.869165865	Bacteria	Bacteria	intra
SBS III vs. Control	Methanococcus vannielii	Sulfurimicrobium lacus	NA	NA	NA	-0.2144	0.001	0.869165865	Bacteria	Bacteria	intra
SBS III vs. Control	Methylophaga pinxianii	Rosistilla carotiformis	NA	NA	NA	0.2131	0.002	0.869165865	Bacteria	Bacteria	intra
SBS III vs. Control	Microbacterium aurum	Vibrio tarriae	NA	NA	NA	0.2129	0.001	0.869165865	Bacteria	Bacteria	intra
SBS III vs. Control	Moritella sp. 24	Polynucleobacter sp. AP-Nino-20-G2	NA	NA	NA	-0.2481	0.001	0.869165865	Bacteria	Bacteria	intra
SBS III vs. Control	Mycobacterium shigaense	Selenomonas sp. oral taxon 478	NA	NA	NA	-0.2031	0.001	0.869165865	Bacteria	Bacteria	intra
SBS III vs. Control	Mycoplasma sp. NEAQ87857	Ruegeria conchae	NA	NA	NA	-0.212	0.001	0.869165865	Bacteria	Bacteria	intra
SBS III vs. Control	Nitrososphaera sp. AFS	Pseudodesulfovibrio piezophilus	NA	NA	NA	0.239	0.004	0.869165865	Bacteria	Bacteria	intra
SBS III vs. Control	Nocardioides ungokensis	Peribacillus muralis	NA	NA	NA	0.2006	0.001	0.869165865	Bacteria	Bacteria	intra
SBS III vs. Control	Ornithinibacillus sp. FSL M8-0202	Paenibacillus sp. FSL R7-0273	NA	NA	NA	0.2046	0.001	0.869165865	Bacteria	Bacteria	intra
SBS III vs. Control	Paenibacillus tianjinensis	Tupanvirus deep ocean	NA	NA	NA	-0.2263	0.001	0.869165865	Bacteria	Bacteria	intra
SBS III vs. Control	Pelistega ratti	uncultured Acetobacterium sp.	NA	NA	NA	0.2353	0.001	0.869165865	Bacteria	Bacteria	intra
SBS III vs.	Pollutimonas thiosulfatoxidans	Pseudomonas sp. BW7P1	NA	NA	NA	-0.2037	0.004	0.869165	Bacteria	Bacteria	intra

Control								865			
SBS III vs. Control	Proteus sp. NMG38-2	uncultured Holophaga sp.	NA	NA	NA	0.2176	0.001	0.869165865	Bacteria	Bacteria	intra
SBS III vs. Control	Pseudarthrobacter phenanthrenivorans	Sphingomonas profundi	NA	NA	NA	0.2308	0.002	0.869165865	Bacteria	Bacteria	intra
SBS III vs. Control	Pseudomonas sp. AO-1	Radiobacillus deserti	NA	NA	NA	-0.21	0.004	0.869165865	Bacteria	Bacteria	intra
SBS III vs. Control	Rhizophagus irregularis	Vibrio ruber	NA	NA	NA	0.2892	0.002	0.869165865	Bacteria	Bacteria	intra
SBS III vs. Control	Salimicrobium jeotgali	Streptomyces violaceus	NA	NA	NA	-0.2047	0.003	0.869165865	Bacteria	Bacteria	intra
SBS III vs. Control	Salisediminibacterium selenitireducens	Sodiomyces alkalinus	NA	NA	NA	-0.2418	0.003	0.869165865	Bacteria	Bacteria	intra
SBS III vs. Control	Shewanella spartinae	Treponema putidum	NA	NA	NA	-0.2046	0.003	0.869165865	Bacteria	Bacteria	intra
SBS III vs. Control	Solibacillus sp. FSL R5-0691	[Candida] duobushaemulonis	NA	NA	NA	-0.2078	0.001	0.869165865	Bacteria	Bacteria	intra
SBS III vs. Control	Sphingopyxis sp. TUF1	Syntrophus aciditrophicus	NA	NA	NA	0.2236	0.001	0.869165865	Bacteria	Bacteria	intra
SBS III vs. Control	Sporosarcina sp. P37	Streptantibioticus cattleyicolor	NA	NA	NA	-0.204	0.001	0.869165865	Bacteria	Bacteria	intra
SBS III vs. Control	Sulfuricaulis limicola	Sulfurimonas marina	NA	NA	NA	0.2018	0.002	0.869165865	Bacteria	Bacteria	intra
SBS III vs. Control	Yersinia intermedia	Zhouia spongiae	NA	NA	NA	0.2326	0.002	0.869165865	Bacteria	Bacteria	intra

Supplementary Table 7. Differential functional pathways (level 3) between patients with SBS and controls

pathway	kegg_pathway	Log2FC_SBSI_vs_Control	Log2FC_SBSII_vs_Control	Log2FC_SBSIII_vs_Control	SBSI_vs_Control_pval	SBSII_vs_Control_pval	SBSIII_vs_Control_pval
ko02010	ABC transporters	0.614089269	0.471759381	0.461758258	0.001989072	0.000310161	0.000133173
ko03010	Ribosome	-0.949768147	-0.252953058	0.054073591	0.00596226	0.413562183	0.170419223
ko02020	Two-component system	1.094972721	0.627154927	0.150722283	2.09295E-06	0.00660017	0.986950105
ko02024	Quorum sensing	-0.249280526	0.187768771	0.471913105	0.563261241	0.383437381	0.004848756
ko00500	Starch and sucrose metabolism	-0.227119055	0.084097701	0.338672082	0.590592835	0.314073293	0.00430993
ko00520	Amino sugar and nucleotide sugar metabolism	0.142917886	0.073007418	0.465577009	0.164441532	0.276592401	0.017478649
ko02060	Phosphotransferase system (PTS)	1.162134876	1.475367319	1.329675781	0.063896396	0.00044706	0.017478649
ko00010	Glycolysis / Gluconeogenesis	-0.248056274	0.033656809	0.320861948	0.510431145	0.368880041	0.009478003
ko00270	Cysteine and methionine	-0.150056219	0.082177332	0.295535101	0.590592835	0.276592401	0.033385967

	metabolism						
ko00052	Galactose metabolism	0.000944979	0.124425763	0.379337669	0.920787076	0.20110221	0.015844007
ko00240	Pyrimidine metabolism	-0.259927401	-0.002766907	0.157190597	0.140669382	0.658865405	0.036418783
ko03070	Bacterial secretion system	1.19828925	0.64568639	-0.033781402	0.015309037	0.030218147	0.960863491
ko00620	Pyruvate metabolism	-0.219408431	0.044266604	0.244161516	0.705277491	0.413562183	0.039674448
ko00970	Aminoacyl-tRNA biosynthesis	-0.755311683	-0.355496859	0.066331614	0.003803328	0.067301313	0.440645766
ko00030	Pentose phosphate pathway	0.197196647	0.229598055	0.258150546	0.368236664	0.020899533	0.001791977
ko00250	Aspartate and glutamate metabolism	-0.69702275	-0.38388753	-0.018825194	0.005148984	0.026141658	0.706566584
ko00260	Serine and threonine metabolism	-0.055281856	0.00019014	0.461679392	0.390134524	0.58318255	0.017478649
ko00190	Oxidative phosphorylation	-0.700853061	-0.386383101	-0.075227534	4.95698E-06	0.042782548	0.908839187
ko00400	Tyrosine and tryptophan biosynthesis	-0.457474874	-0.338700911	-0.044702886	0.013491807	0.114086329	0.65844134
ko03030	DNA replication	0.23472552	-0.034960497	0.214302854	0.052521331	0.883087139	0.033385967

Note: The table presents the *p*-values and log2 fold changes (log2FC) of the top 20 KEGG differential pathways. Group differences were assessed using the Wilcoxon rank-sum test. Log2FC was calculated as log2 (mean abundance in SBS subtype / mean abundance in controls) for each pathway. SBS I: short bowel syndrome type 1; SBS II: short bowel syndrome type 2; SBS III: short bowel syndrome type 3; Control: healthy controls.

Supplementary Table 8. Differential KEGG orthology (KO) genes between patients with SBS and controls.

KO	Pathway level 3	Log2FC_SBS I_vs_Control	Log2FC_SBS II_vs_Control	Log2FC_SBS III_vs_Control	SBS I_vs_Control _qval	SBS II_vs_Control _qval	SBS III_vs_Control _qval	KO annotation
K02911	Ribosome	-0.941859011	NA	NA	0.037926962	NA	NA	RP-L32, MRPL32, rpmF; large subunit ribosomal protein L32
K10117	ABC transporters	-3.352180882	NA	NA	0.010221676	NA	NA	msmE; raffinose/stachyose/melibiose transport system substrate-binding protein
K16787	ABC transporters	-1.338589357	NA	NA	0.008590549	NA	NA	ecfA2; energy-coupling factor transport system ATP-binding protein [EC:7.-.-.-]
K16786	ABC transporters	-1.50460237	NA	NA	0.007947595	NA	NA	ecfA1; energy-coupling factor transport system ATP-binding protein [EC:7.-.-.-]
K02954	Ribosome	-0.724798538	NA	NA	0.015689107	NA	NA	RP-S14, MRPS14, rpsN; small subunit ribosomal protein S14
K02902	Ribosome	-0.537206644	NA	NA	0.020141132	NA	NA	RP-L28, MRPL28, rpmB; large subunit ribosomal protein L28
K02110	Oxidative phosphorylation	-1.913611382	NA	NA	0.011694749	NA	NA	ATPF0C, atpE; F-type H+-transporting ATPase subunit c

K03205	Bacterial secretion system	-4.324855764	NA	NA	0.004224672	NA	NA	virD4, lvhD4; type IV secretion system protein VirD4 [EC:7.4.2.8]
K01955	aspartate and glutamate metabolism	-1.550749051	NA	NA	0.014417768	NA	NA	carB, CPA2; carbamoyl-phosphate synthase large subunit [EC:6.3.5.5]
K02904	Ribosome	-1.66760722	NA	NA	0.041660057	NA	NA	RP-L29, rpmC; large subunit ribosomal protein L29
K01190	Other glycan degradation	NA	-0.952775546	NA	NA	0.023356754	NA	lacZ; beta-galactosidase [EC:3.2.1.23]
K15580	ABC transporters	NA	1.110894714	NA	NA	0.020134947	NA	oppA, mppA; oligopeptide transport system substrate-binding protein
K02760	Phosphotransferase system (PTS)	NA	2.659739639	NA	NA	0.013142515	NA	celA, chbB; cellobiose PTS system EIIB component [EC:2.7.1.196 2.7.1.205]
K02769	Phosphotransferase system (PTS)	NA	1.192100306	NA	NA	0.015570719	NA	fruAb; fructose PTS system EIIB component [EC:2.7.1.202]
K00053	Leucine and isoleucine biosynthesis; Pantothenate and CoA biosynthesis	NA	-0.827737998	NA	NA	0.042669466	NA	ilvC; ketol-acid reductoisomerase [EC:1.1.1.86]
K02822	Phosphotransferase system (PTS)	NA	1.563930394	NA	NA	0.014164915	NA	ulaB, sgaB; ascorbate PTS system EIIB component [EC:2.7.1.194]
K02073	ABC transporters	NA	1.218408721	NA	NA	0.018854048	NA	metQ; D-methionine transport system substrate-binding protein
K02793	Phosphotransferase system (PTS)	NA	2.04992978	NA	NA	0.014164915	NA	manXa; mannose PTS system EIIA component [EC:2.7.1.191]
K02072	ABC transporters	NA	1.312448663	NA	NA	0.00700412	NA	metI; D-methionine transport system permease protein
K02794	Phosphotransferase system (PTS)	NA	1.616264541	NA	NA	0.015335401	NA	manX; mannose PTS system EIIAB component [EC:2.7.1.191]
K01061	Chlorocyclohexane and chlorobenzene degradation	NA	NA	3.894673865	NA	NA	0.014440969	E3.1.1.45; carboxymethylenebutenolidase [EC:3.1.1.45]
K21071	Pentose phosphate pathway	NA	NA	-2.018218028	NA	NA	0.010391516	pfk, pfp; ATP-dependent phosphofructokinase / diphosphate-dependent phosphofructokinase [EC:2.7.1.11 2.7.1.90]
K06726	ABC transporters	NA	NA	2.088293537	NA	NA	0.032662075	rbsD; D-ribose pyranase [EC:5.4.99.62]
K19309	ABC transporters	NA	NA	-2.610254864	NA	NA	0.034243447	bcrA; bacitracin transport system ATP-binding protein
K09690	ABC transporters	NA	NA	-1.981287931	NA	NA	0.020010144	ABC-2.LPSE.P; lipopolysaccharide transport system permease protein
K01470	Arginine and proline metabolism	NA	NA	-0.229185373	NA	NA	0.043178357	E3.5.2.10; creatinine amidohydrolase [EC:3.5.2.10]
K16957	ABC transporters	NA	NA	1.901193255	NA	NA	0.041611585	tcyK; L-cystine transport system substrate-binding protein
K11601	ABC transporters	NA	NA	2.749397192	NA	NA	0.042793216	mntC;

								+A26+C30:I30+B30:I30+A26+C30:I30+A30:I30
K20492	ABC transporters	NA	NA	-1.929733656	NA	NA	0.028466646	nisG, spaG, cprC; lantibiotic transport system permease protein
K10202	ABC transporters	NA	NA	-2.298267038	NA	NA	0.024087817	ABC.NGC.P1; N-acetylglucosamine transport system permease protein

Note: The table presents the adjusted p-values (FDR/q-values) and log2 fold changes (log2FC) of the top 10 differential KEGG orthology (KO) genes for each SBS subtype (SBS I, SBS II, SBS III) extracted from the top 10 most significantly altered pathways. “KO annotation” indicates KO entries without functional annotation in the KEGG database. Group differences were assessed using the Wilcoxon rank-sum test. Log2FC was calculated as log2(mean abundance in each SBS subtype / mean abundance in controls) for each KO gene. SBS I: short bowel syndrome type 1; SBS II: short bowel syndrome type 2; SBS III: short bowel syndrome type 3; Control: healthy controls.

Supplementary Table 9. Spearman correlations between gestational age and α-diversity indices

**Index**	**r**	**P value**
Richness	-0.0216	0.9033
Chao1	-0.0002	0.9993
ACE	0.0172	0.9231
Shannon	-0.1518	0.3916
Simpson	-0.1731	0.3276
Inverse Simpson	-0.1731	0.3276
