# Supplementary material for: Multi-kingdom gut microbiota analyses identify biomarkers of different types of pediatric short bowel syndrome
Source: mSystems. 2026 Sep 1;11(9):e00316-26. doi: 10.1128/msystems.00316-26 (PMC13595922; doi:10.1128/msystems.00316-26)
Supplement: Supplementary file 2 [file msystems.00316-26-s0001.docx]

**Supplementary Figure 1: Gut microbiota composition of the control group and SBS group.** (a) Relative abundance of gut microbiota at the phylum level in the control group and different SBS subtypes (SBS I, SBS II, and SBS III). (b) Relative abundance of gut microbiota at the genus level in the control group and different SBS subtypes (SBS I, SBS II, and SBS III).

**Supplementary Figure 2: Impact of remaining small intestinal length on gut microbial diversity in pediatric SBS patients.** (a) Comparison of shannon diversity between SBS patients stratified by remaining small intestinal length. (b) Comparison of richness diversity between SBS patients stratified by remaining small intestinal length. (c) Principal coordinates analysis (PCoA) based on Bray–Curtis dissimilarity in SBS patients stratified by remaining small intestinal length (*PERMANOVA, P = 0.227*). (d) Correlation between remaining small intestinal length and gut microbial alpha diversity.

**Supplementary Figure 3: Gut microbial taxonomic composition in control and SBS patients without antibiotic exposure.** (a) Relative abundance at the phylum level. (b) Relative abundance at the family level. (c) Relative abundance at the genus level.
